# To explore the mechanism of acupoint application in the treatment of primary dysmenorrhea by 16S rDNA sequencing and metabolomics

**DOI:** 10.3389/fendo.2024.1397402

**Published:** 2024-05-30

**Authors:** Lin Wang, Tie Li, Wen-Xuan Cao, Jin-Ying Zhao, Xiao-Hong Xu, Jia-Peng Chai, Jia-Xun Zhang, Jia Liu, Fu-Chun Wang

**Affiliations:** ^1^Department of Acupuncture and Tuina, Changchun University of Chinese Medicine, Changchun, Jilin, China; ^2^Department of Pharmacy, Changchun University of Chinese Medicine, Changchun, Jilin, China

**Keywords:** primary dysmenorrhea, 16S r DNA sequencing, non-targeted metabolomics, graphene-based warm uterus acupoint paste, acupoint application

## Abstract

Graphene-based warm uterus acupoint paste (GWUAP) is an emerging non-drug alternative therapy for the treatment of primary dysmenorrhea (PD), but the underlying mechanism is still unclear. SD female rats were randomly divided into control group, model group and treatment group to explore the mechanism of GWUAP in the treatment of PD. Combined with 16S rDNA and fecal metabolomics, the diversity of microbiota and metabolites in each group was comprehensively evaluated. In this study, GWUAP reduced the torsion score of PD model rats, improved the pathological morphology of uterine tissue, reduced the pathological damage score of uterine tissue, and reversed the expression levels of inflammatory factors, pain factors and sex hormones. The 16 S rDNA sequencing of fecal samples showed that the abundance of Lactobacillus in the intestinal flora of the model group decreased and the abundance of Romboutsia increased, while the abundance of Lactobacillus in the intestinal flora of the treatment group increased and the abundance of Romboutsia decreased, which improved the imbalance of flora diversity in PD rats. In addition, 32 metabolites related to therapeutic effects were identified by metabolomics of fecal samples. Moreover, there is a close correlation between fecal microbiota and metabolites. Therefore, the mechanism of GWUAP in the treatment of PD remains to be further studied.

## Introduction

Primary dysmenorrhea (PD) refers to menstrual pain in the absence of pelvic lesions (1, 2). The clinical manifestations are recurrent spasmodic lower abdominal pain during menstruation or before and after menstruation, mostly accompanied by waist soreness, headache and dizziness. It is the most common cause of gynecological visits. Its high incidence affects 50% to 90% of women around the world, and half of them describe pain as moderate to severe, which seriously reduces the quality of life of patients. At present, NSAID drugs are the first-line drugs for clinical treatment of PD, which can relieve pain in time. However, the long-term efficacy is not ideal. After drug withdrawal, the disease often relapses, and long-term use is likely to cause adverse reactions such as nausea and vomiting. However, the disease often relapses after drug withdrawal, and long-term use is likely to cause adverse reactions such as nausea and vomiting (3, 4). Therefore, complementary and alternative therapies are urgently needed to intervene in PD.

Acupoint application is one of the external treatment methods of traditional Chinese medicine. Under the overall action of acupoints, meridians and drugs, it enters from the outside to the inside, and regulates the Zang-Fu organs by means of the meridian tropism of drugs (5). Its direct approach, rapid onset, low recurrence rate and simple and safe personalized diagnosis and treatment are more prominent advantages in clinical treatment (6, 7). The Graphene-based warm uterus acupoint paste (GWUAP) used in this experiment is a new generation product that combines traditional acupoint application materials containing traditional Chinese medicine ingredients with 1 ‰ graphene by using supercritical CO_2_ extraction technology and microcapsule production process.

The previous experimental results of the research group showed that GWUAP had the effects of promoting blood circulation and removing blood stasis, dispelling cold and relieving pain. Specifically, it can greatly improve the pain symptoms of PD rats, regulate serum inflammatory factors TNF-α, immune factor indicators IgA and IgG, and Th1/Th2-related cytokines (8, 9). However, as a dosage form attached to the skin surface, whether it can play a role in the treatment of PD by regulating microbial flora is still unclear. In recent years, researchers have gradually revealed the changes of PD metabolites by metabolomics technology, mainly focusing on the mechanism of traditional Chinese medicine, but few studies have reported its effect mechanism (10–13). Therefore, this study explores the biological mechanism of GWUAP in the treatment of PD from the perspective of microbial flora combined with metabolomics, in order to provide a scientific basis for further research and development of GWUAP.

## Materials and methods

### Animals, housing, and experimental design

Twelve SPF grade sexually mature female SD rats, healthy and unmated, 3 months old, body mass 180 g~220 g. The rats were housed in the Animal Experiment Centre of Changchun University of Chinese Medicine. Feeding conditions: room temperature 23°C~25°C, relative humidity 50%~60%, free feeding and drinking, and ordinary feed. After 1 week of adaptive rearing under fluorescent light with 12 h of alternating light and dark cycles, the animals were randomly divided into three groups, namely control group, model group and treatment group, with four animals in each group. Except for the control group, both the model and treatment groups used estradiol benzoate combined with oxytocin to replicate the PD rat model as described in the literature (14). The rats were subjected to abdominal hair removal (3 cm×3 cm) 24 h before the start of the experiment, followed by subcutaneous injection of oestradiol benzoate (Harbin Sanma Veterinary Pharmaceutical Co., Ltd., China. Approval No.: Veterinary Medicine 080232511) into the femur at the same time every day. To avoid subcutaneous hard nodules, the left side was injected on odd-numbered days, and the right side was injected on even-numbered days, once a day for 10 consecutive days, 0.25 mL/rat on the 1st and 10th days, and 0.1 mL/rat on the 2nd to 9th days. All study on animals were done in accordance with ARRIVE guidelines.

### Intervention methods

**Figure 1:**
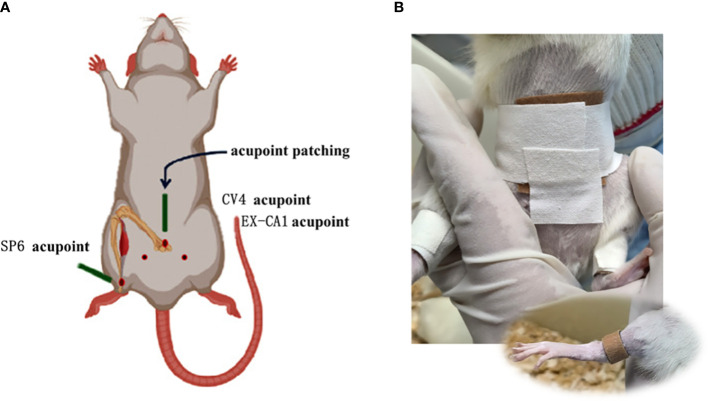
**(A, B)** are the position diagrams of CV4, EX-CA1 and SP6 acupoints in SD rats.

According to the experimental requirements, the original size of 2 cm × 2 cm acupoint paste was cut into 4 pieces of 1 cm × 1 cm size for acupoint coverage. Subsequently, a certain length of medical adhesive tape was cut and pasted on the acupoint, and then wrapped around the rats to fix them. For the positioning of the paste, we referred to the “commonly used animal acupoints and atlases” in Experimental Acupuncture and Moxibustion. The acupoints of ‘ Guanyuan ‘ (CV4, about 25 mm below the umbilicus), bilateral ‘ Zigong ‘ (EX-CA1, about 30 mm below the umbilicus, about 20 mm beside the midline of the abdomen) and bilateral ‘ Sanyinjiao ‘ (SP6, about 10 mm above the tip of the medial malleolus of the hind limb) were selected (15). The diagram is as follows (**Figures 1**). From the first day of modeling, the treatment group was given GWUAP (Jilin Ailuokang Pharmaceutical Technology Development Co., Ltd., China) application intervention and fixed with medical tape. The control group and the model group did not do the application intervention, but in order to maintain the same pressure, only the medical tape was wound around the corresponding acupoints for a total of 10 d, 1 time/d, 5 h/time.

### Behavior observation

On the 11th day, each rat was intraperitoneally injected with 2 U oxytocin (Jilin Huamu Animal Health Products Co., Ltd., China. Approval No.: Veterinary Medicine 070012778), and then the writhing reaction of rat uterine contraction was used as the standard for the success of PD model replication (14). The time from injection of oxytocin to writhing reaction (latent time) and the number of writhing within 30 min were recorded in each group. The writhing score was performed according to the behavioral scoring criteria: normal exploratory behavior, 0 point; the body trunk is inclined to one side, and the abdomen is depressed inward due to contraction, 1 point; hindlimb extension, hind claw dorsiflexion, trunk extension with pelvic rotation, 2 points; abdominal muscle contraction, hind limb extension, 3 points (16).

### Sample collection

At the same time of behavioral experiment observation, 4 fresh feces of rats were collected from each group, and 3-5 granules were taken from each. It was placed in a sterile cryopreservation tube and sealed, frozen in liquid nitrogen, and then transferred to a refrigerator at -80°C for subsequent 16S rDNA sequencing and metabolomics analysis. After the writhing reaction was recorded, 2% pentobarbital sodium was injected intraperitoneally at a dose of 40 mg/kg for anesthesia, supine fixation, and laparotomy. About 4 mL of blood was taken from the abdominal aorta with a disposable blood collection needle. The blood was placed at room temperature for 2 h, centrifuged for 15 min, and the isolated serum was frozen in the refrigerator at -20°C for the determination of estrogen (E2), prostaglandin F2α (PGF2α), β-endorphin (β-EP) and tumor necrosis factor (TNF-α). Subsequently, the rats were sacrificed by cervical dislocation, and the complete uterine tissue was dissected for pathomorphological detection.

### Enzyme-linked immunosorbent assay analysis

The serum samples that had been isolated from each group of rats were taken, and the absorbance value was measured at 450 nm according to the operation instructions of the corresponding kit. The contents of serum estrogen (E2), prostaglandin F2α (PGF2α), β-endorphin (β-EP) and tumor necrosis factor (TNF-α) were calculated according to the standard curve (All are purchased from Shanghai Jianglai Biotechnology Co., Ltd., China. batch numbers are: 102523007115251111, 102523007487221111, 102523007100851111, 102523007132021111).

### Pathological change

Hematoxylin-eosin (HE) staining was used to observe the pathological changes of uterine tissue in rats: The fresh uterine tissues of 4 rats in each group were washed and immersed in 4% paraformaldehyde solution for 48 h. After ethanol gradient dehydration and paraffin embedding, the sections (4 μm) were placed on glass slides, dewaxed with xylene, stained with hematoxylin and eosin staining for nucleus and cytoplasm, dehydrated, and sealed with neutral gum. The pathological changes of uterine tissue in each group were observed under a microscope.

The pathological damage of rat uterine tissue was scored: referring to the experiment of Wei et al., according to the morphological characteristics of uterine tissue under the microscope, the non-pathological uterus was recorded as 0 point; endometrial degeneration and necrosis was recorded as 1 point; the edema of lamina propria was recorded as 2 points; the increase of lamina propria gland was recorded as 3; inflammatory cell infiltration lamina propria score was recorded as 4 points; the myometrium inflammation score was recorded as 5 points (17).

### 16S rRNA gene profiling

According to the manufacturer ‘s instructions, use the Mag-bind soil DNA kit (Omega, China). Total microbial DNA was extracted from fecal samples by SDS method, and then the purity and concentration of DNA were monitored by 1% agarose gel electrophoresis (Solarbio, Beijing, China). DNA was diluted to a concentration of 1 ng/μl with sterile water according to the concentration. The V3-V4 hypervariable region of bacterial 16S was amplified by PCR using primers 341F (5 ‘ - CCTAYGGGRBGCASCAG-3 ‘) and 806R (5 ‘ - GGACTACNNGGGGTATCTAAT -3 ‘). All PCR reactions were carried out in 30μL reactions with 15μL of Phusion®High-Fidelity PCR Master Mix (New England Biolabs, USA); 0.2μM of forward and reverse primers, and about 10 ng template DNA. Thermal cycling consisted of initial denaturation at 98°C for 1 min, followed by 30 cycles of denaturation at 98°C for 10 s, annealing at 50°C for 30 s, and elongation at 72°Cfor 60 s. Finally 72°C for 5 min. Mix same volume of 1X loading buffer with PCR products and operate electrophoresis on 2% agarose gel for detection. Samples with bright main strip between 400-450bp were chosen for further experiments. PCR products was mixed in equidensity ratios. Then, mixture PCR products was purified with AxyPrepDNA Gel Extraction Kit (Axygen, USA). Sequencing libraries were generated using NEB Next®Ultra™DNA Library Prep Kit for Illumina (New England Biolabs, USA) following manufacturer’s recommendations and index codes were added. The library quality was assessed on the Qubit@ 2.0 Fluorometer (Thermo Scientific, USA) and Agilent 2100 bioanalyzer(Agilent, USA). At last, the library was sequenced on an Illumina NovaSeq 600 platform. Paired-end reads from the original DNA fragments were merged using FLASH, a very fast and accurate analysis tool Sequences analysis were performed by UPARSE software package using the UPARSE-OTU and UPARSE-OTUref algorithms. In-house Perl scripts were used to analyze alpha (within samples) and beta (among samples) diversity. Sequences with ≥97% similarity were assigned to the same OTUs. To confirm differences in the abundances of individual taxonomy between the two groups, STAMP software was utilized. LEfSe was used for the quantitative analysis of biomarkers within different groups.

### Metabolomics analysis

Fecal samples were naturally thawed at room temperature, and an appropriate amount of samples were added to a pre-cooled methanol/acetonitrile/water solution (2: 2: 1, v/v), extracted three times, 100 μL each time. After mixing, low-temperature ultrasound for 0.5 h, -20°C for 10 min, then centrifuged at 4000 rpm below 4°C for 20 min, and the supernatant was freeze-dried. The metabolites were re-dissolved in 150 μL of 50% cold methanol and centrifuged at 4000 rpm for 30 minutes. The supernatant was transferred to an automatic injection bottle for metabolic analysis. Quality control (QC) samples monitor the stability of the instrument by pooling each sample of the same volume.

Analysis was performed using an UHPLC (Vanquish UHPLC, Thermo) coupled to a Orbitrap in Shanghai Applied Protein Technology Co., Ltd. For HILIC separation, samples were analyzed using a 2.1 mm × 100 mm ACQUIY UPLC BEH Amide 1.7 µm column (waters, Ireland). In both ESI positive and negative modes, the mobile phase contained A=25 mM ammonium acetate and 25 mM ammonium hydroxide in water and B= acetonitrile. The gradient was 98% B for 1.5 min and was linearly reduced to 2% in 10.5 min, and then kept for 2 min, and then increased to 98% in 0.1 min, with a 3 min re-equilibration period employed. The ESI source conditions were set as follows: Ion Source Gas1 (Gas1) as 60, Ion Source Gas2 (Gas2) as 60, curtain gas (CUR) as 30, source temperature: 600°C, IonSpray Voltage Floating (ISVF) ± 5500 V. In MS only acquisition, the instrument was set to acquire over the m/z range 80-1200 Da, the resolution was set at 60000 and the accumulation time was set at 100ms. In auto MS/MS acquisition, the instrument was set to acquire over the m/z range 70-1200 Da, the resolution was set at 30000 and the accumulation time was set at 50ms, exclude time within 4 s. The raw MS data were converted to mzXML files using ProteoWizard MSConvert before importing into freely available XCMS software.

### Correlation analysis of 16S rDNA and metabolomics

Spearman statistical method was used to analyze the correlation coefficient between the significant difference flora and the significant difference metabolites screened in the experimental samples. Combined with R language and Cytoscape software, hierarchical clustering and correlation network analysis were carried out to explore the interaction between flora and metabolites from multiple perspectives.

### Statistical analysis

The data were analyzed by statistical software SPSS26.0, and the measurement data were expressed as means ± SD. One-way ANOVA was used for comparison between multiple groups, and Student ‘s unpaired t-test was used for comparison between groups. *P* < 0.05 was considered statistically significant.

## Results

### Comparison of torsion reaction, inflammatory factors, pathological morphology and score of uterine tissue in each group

**Figure 2:**
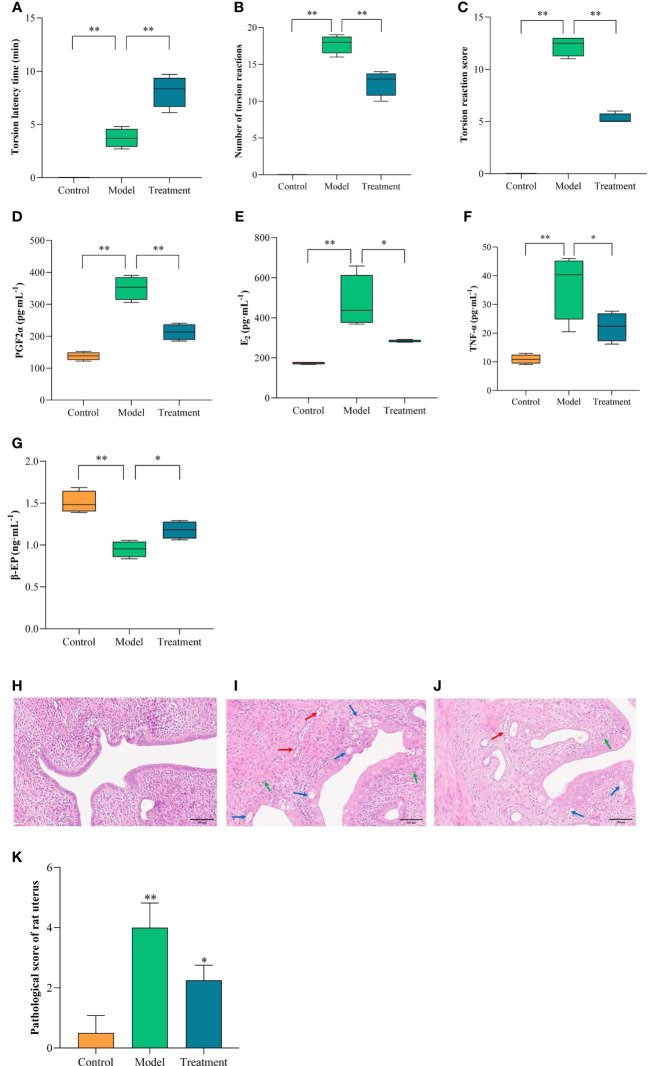
Comparison of torsion reaction, inflammatory factors, pathological morphology and score of uterine tissue in each group. The torsion reaction of rats in each group. **(A)** Torsion latency time; **(B)** Number of torsion reactions; **(C)** Torsion reaction score. The content changes of inflammatory factors in each group. **(D)** PGF2α; **(E)** E_2_; **(F)** TNF-α; **(G)** β-EP. Uterine pathological morphology and injury score. **(H)** Control; **(I)** Model; **(J)** Treatment; **(K)** Pathological score of rat uterus. Blue arrows showed vacuolar degeneration of epithelial cells, red arrows showed congestion, and green arrows showed neutrophil infiltration. Scale = 100μm. Compared with the control group, **p < 0.01; Compared with the model group, *p < 0.05, **p < 0.01.

The torsion of rats reflected the results (**Figures 2A–C**). no torsion reaction was seen in the control group within 30 min after oxytocin injection. Compared with the control group, the model group showed torsion reaction, and the latency time of torsion was shortened, and the number of torsion reactions and torsion scores within 30 min were significantly higher (*P*<0.01). compared with the model group, the latency time of torsion in the treated rats was prolonged, and the number of torsion reactions and torsion scores within 30 min were significantly lower (*P*<0.01).

ELISA index was used to detect the protein expression level of inflammatory factors in each group of rats. (**Figures 2D–G**). Compared with the control group, the serum levels of TNF-α, PGF2α and E_2_ in the model group were significantly increased, and the content of β-EP was significantly decreased (*P* < 0.01). Compared with the model group, the serum levels of TNF-α, PGF2α and E_2_ in the treatment group were significantly decreased, and the content of β-EP was significantly increased (*P* < 0.05, *P* < 0.01).

HE staining results of uterine tissue of rats in each group (**Figures 2H–J**). The structure of endometrial epithelial cells in the control group was clear, neatly arranged, and single-layer columnar. No congestion, edema and obvious inflammatory cell infiltration were found in the glandular cavity and endometrial stroma. The endometrial epithelial cells of the model group had a large number of vacuolar degeneration, showing a pseudostratified columnar. Gland cavity congestion, endometrial interstitial congestion with edema, and a large number of neutrophil infiltration. There was a small amount of vacuolar degeneration in the endometrial epithelium of the rats in the treatment group, which was high columnar. The degree of congestion and edema in the glandular cavity and endometrial stroma was mild, with only a small amount of neutrophil infiltration. It can be seen that GWUAP has a certain improvement effect on the uterine pathology of PD rats.

The pathological score was based on microscopic evaluation, and the scores that met the criteria were evaluated cumulatively (**Figure 2K**). Compared with the control group, the pathological damage score of uterine tissue in the model group was significantly increased (*P* < 0.01). Compared with the model group, the pathological score of the treatment group was significantly lower (*P* < 0.05).

### The effect of GWUAP on fecal intestinal flora in PD rats

**Figure 3:**
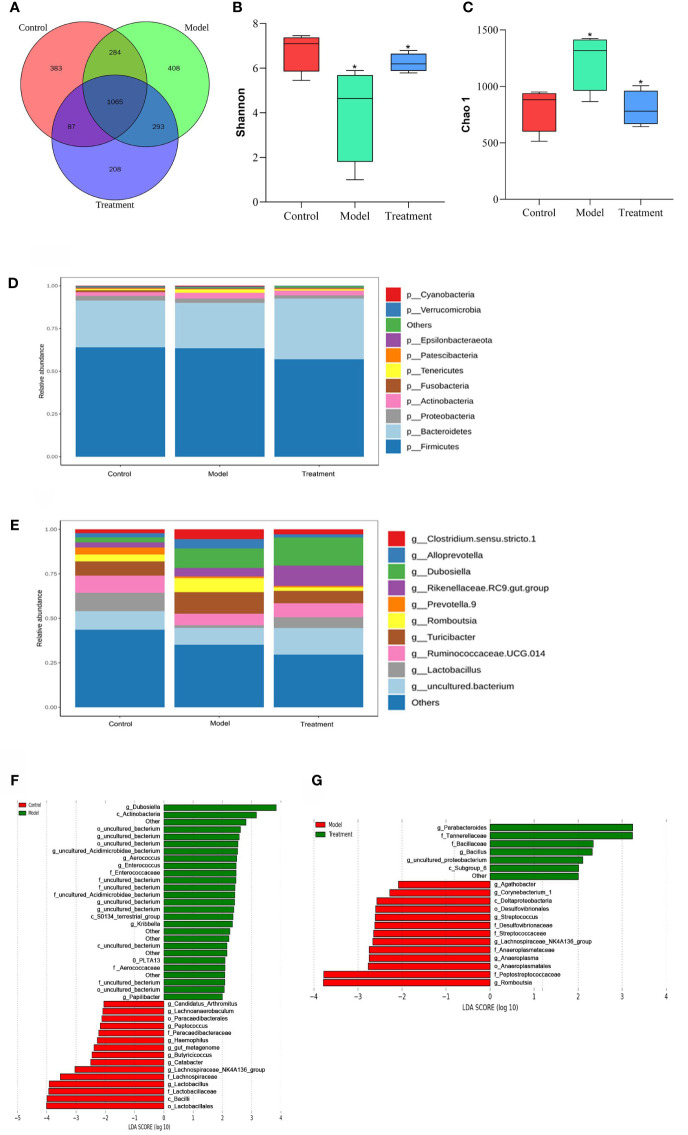
The effect of GWUAP on fecal intestinal flora in PD rats. **(A)** OTU distribution map between groups. The effect of GWUAP on the diversity of intestinal flora. **(B)** Shannon index; **(C)** Chao1 index. phylum, genus level distribution of rat intestinal flora by group. **(D)** Relative abundance of species at phylum level. **(E)** Relative abundance of species at genus level. The significantly enriched bacterial taxa in different groups as determined by LEfSe analysis. **(F)** Control group and model group; **(G)** Model group and treatment group. The symbol * indicates that Shannon index and Chao1 index are statistically significant compared with the control group and the model group, respectively.

Operational taxonomic units (OTUs) play an important role in the study of species diversity information. The composition similarity and overlap of OTUs between different treatment groups can be analyzed by Venn diagram. The number of OTUs common to the control, model and treatment groups was 1065, while the number of OTUs unique to these three groups was 383, 408 and 208, respectively. This shows that GWUAP can affect the OTU composition of intestinal flora in PD rats (**Figure 3A**).

The Shannon index and Chao1 index in Alpha diversity were used to analyze the diversity and abundance of intestinal communities in the samples (**Figures 3B, C**). Compared with the control group, the Shannon index of the model group was lower and the Chao1 index was higher (*P* < 0.05). Compared with the model group, the Shannon index of the treatment group was higher and the Chao1 index was lower (*P* < 0.05).

T-test analysis was performed between the two groups at the phylum and genus levels. The top 10 species in terms of maximum abundance were selected to generate a cumulative bar chart of species relative abundance (**Figures 3D, E**). At the phylum classification level, there was no significant difference among the three groups. The fecal microbiota of each group was mainly composed of Firmicutes, Bacteroidetes and Proteobacteria, of which Firmicutes accounted for the largest proportion. At the genus level, compared with the Control group, Lactobacillus in the Model group was significantly reduced (*P* < 0.05), and the abundance of Romboutsia was increased (*P* < 0.05). Compared with the Model group, the abundance of Lactobacillus in the Treatment group was significantly increased (*P* < 0.05), and the abundance of Romboutsia was decreased (*P* < 0.05).

According to the LDA effect size (LEfSe) analysis (LDA score > 2.0, *P* < 0.05), there were 42 groups with significant differences between the control group and the model group: 15 in the control group and 27 in the model group. The control group mainly enriched Candidatus _ Arthromitus, Lachnoanaerobaculum, Paracaedibacteraies, Peptococcus, Paracaedibacteraceae, Haemophilus, gut _ metagenome, Butyricicoccus, Catabacter, Bacilli, Lactobacillales, etc. There were 20 groups with significant differences between the model group and the treatment group: 13 in the model group and 7 in the treatment group. The treatment group mainly enriched Parabacteroides, Tannerellaceae, Bacillaceae, Bacillus, uncultured _ proteobacterium, Subgroup _ 6, etc (**Figures 3F, G**).

### Effect of GWUAP on fecal metabolomics in PD rats

**Figure 4:**
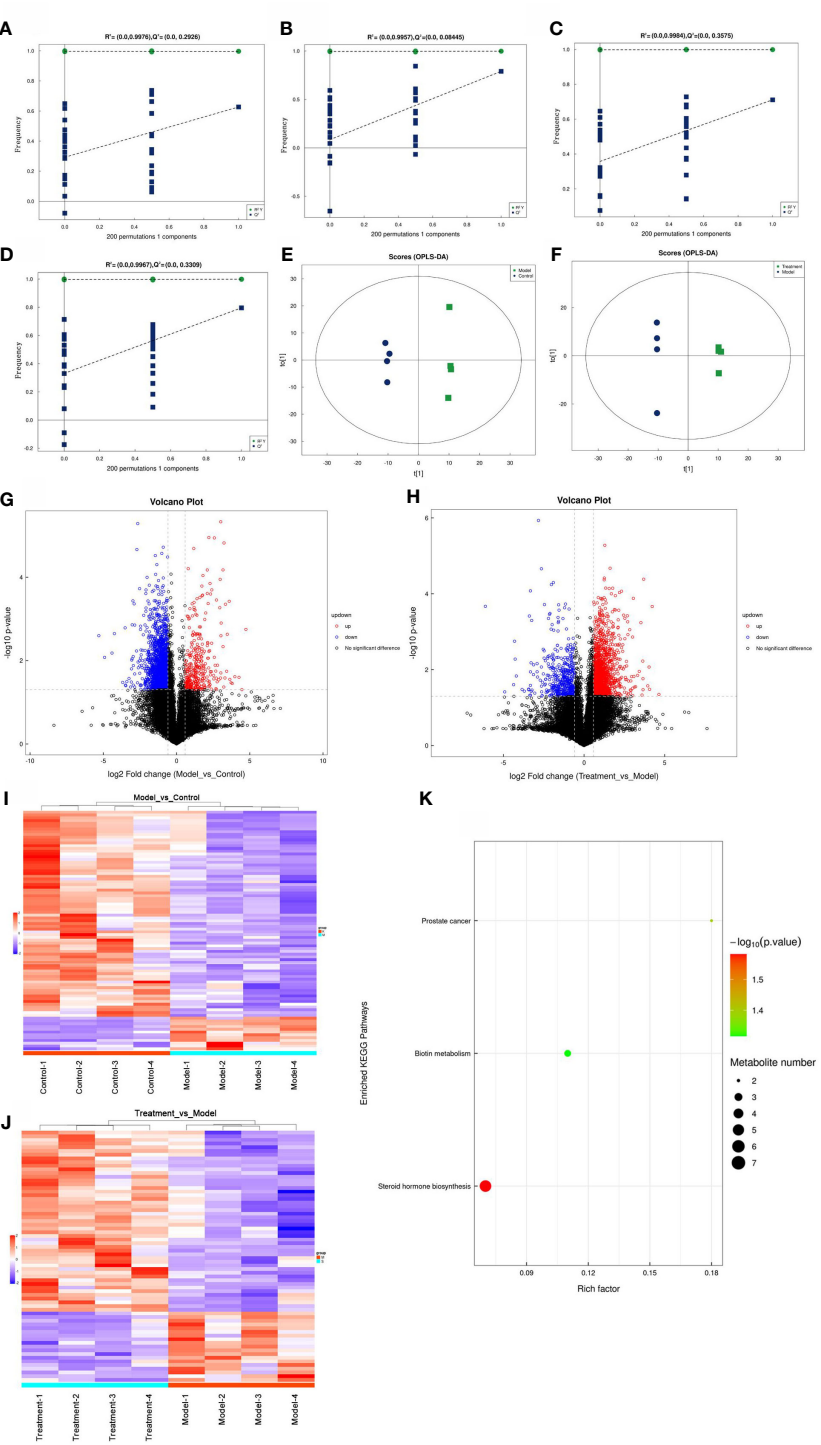
OPLS-DA substitution test graph. **(A)** Control group and model group in positive ion mode; **(B)** Control group and model group in negative ion mode; **(C)** Model group and treatment group in positive ion mode; **(D)** Model group and treatment group in negative ion mode. The OPLS-DA score graph. **(E)** Control group and model group; **(F)** Model group and treatment group. Differential metabolites volcano plot in the positive and negative ion co-mode. **(G)** Control group and model group; **(H)** Model group and treatment group. Red dots indicate significantly up-regulated differential metabolites, blue dots indicate significantly down-regulated differential metabolites, and black dots indicate non-significant differential metabolites. Differential metabolites heatmap in the positive and negative ion co-mode. **(I)** Control group and model group; **(J)** Model group and treatment group. **(K)** KEGG enrichment pathway bubble diagram of model group and treatment group.

Orthogonal partial least squares discriminant analysis (OPLS-DA) can filter out noise irrelevant to classification information and improve the analytical ability and effectiveness of the model. On the OPLS-DA score plot (**Figures 4E, F**), the maximum difference between groups is reflected in t [1], so the variation between groups can be directly distinguished from t [1], while the orthogonal principal component to [1] reflects the variation within the group. The model evaluation parameters (R^2^Y, Q^2^) obtained by 7-fold cross-validation are listed in **Supplementary Table S1**. Q^2^ > 0.5, indicating that the model is stable and reliable. There was a significant separation between the model group and the control group, the treatment group and the model group.

In order to avoid the over-fitting of the supervised model in the modeling process, the permutation test is used to test the model to ensure the validity of the model. With the decrease of permutation retention, the R2 and Q2 of the random model gradually decrease, indicating that there is no over-fitting phenomenon in the original model, and the robustness of the model is good. The results are reliable (**Figures 4A–D**).

In the OPLS-DA analysis, the VIP value (Variable importance of projection) was used to measure the metabolite expression profile of the two groups, and the related differential metabolites were evaluated. The significance of the differential metabolites between the two groups was analyzed by T-test (VIP > 1, P < 0.05). The p value, VIP and Fold change values were visually screened, and the results were represented by volcano plots (**Figures 4G, H**). A total of 667 metabolites were labeled in the model group and the control group. Among them, 152 differential metabolites were up-regulated and 515 differential metabolites were down-regulated in the model group (**Supplementary Table S2**). In contrast, a total of 589 metabolites were labeled in the treatment group and the model group. Among them, 428 differential metabolites were up-regulated and 161 differential metabolites were down-regulated in the treatment group (**Supplementary Table S3**).

In the differential metabolite heat map in the positive and negative ion co-mode, each row represents a differential metabolite (i.e., the ordinate is a metabolite with significant differential expression), and each column represents a group of samples (i.e., the abscissa is the sample information). The color blocks at different positions represent the relative expression of metabolites at the corresponding positions. Red represents a relatively high expression level, and blue represents a relatively low expression level. The color contrast of the difference between the groups was obvious, indicating that there were significant metabolic changes in the feces of the model group and the treatment group (**Figures 4I, J**).

Through the screening of fecal metabolites in the three groups of rats, a total of 52 differential metabolites were found, which were common potential biomarkers. The ionic strength of 52 biomarkers was compared and analyzed. Compared with the control group, 16 biomarkers were up-regulated (*P* < 0.05) and 36 biomarkers were down-regulated (*P* < 0.05) in the model group. Compared with the model group, 16 biomarkers were down-regulated (*P* < 0.05) and 36 biomarkers were up-regulated (*P* < 0.05) in the treatment group. The callback of biomarker expression may be evidence for GWUAP in the treatment of PD in terms of biological effects. See the results (**Supplementary Table S4**).

The results of metabolic pathway enrichment analysis were shown in a bubble diagram (**Figure 4K**). Each bubble in the bubble diagram represents a metabolic pathway. The abscissa and bubble size of the bubble represent the size of the impact factor of the pathway in the topological analysis. The larger the bubble, the larger the impact factor, the deeper the bubble color, the smaller the p value, and the more significant the enrichment degree. The Rich factor represents the proportion of the number of differential metabolites in the pathway to the number of metabolites annotated in the pathway. There were three metabolic pathways that were significantly different between the model group and the treatment group, namely Steroid hormone biosynthesis, Prostate cancer, and Biotin metabolism.

### Correlation analysis of 16S rDNA and metabolomics

**Figure 5:**
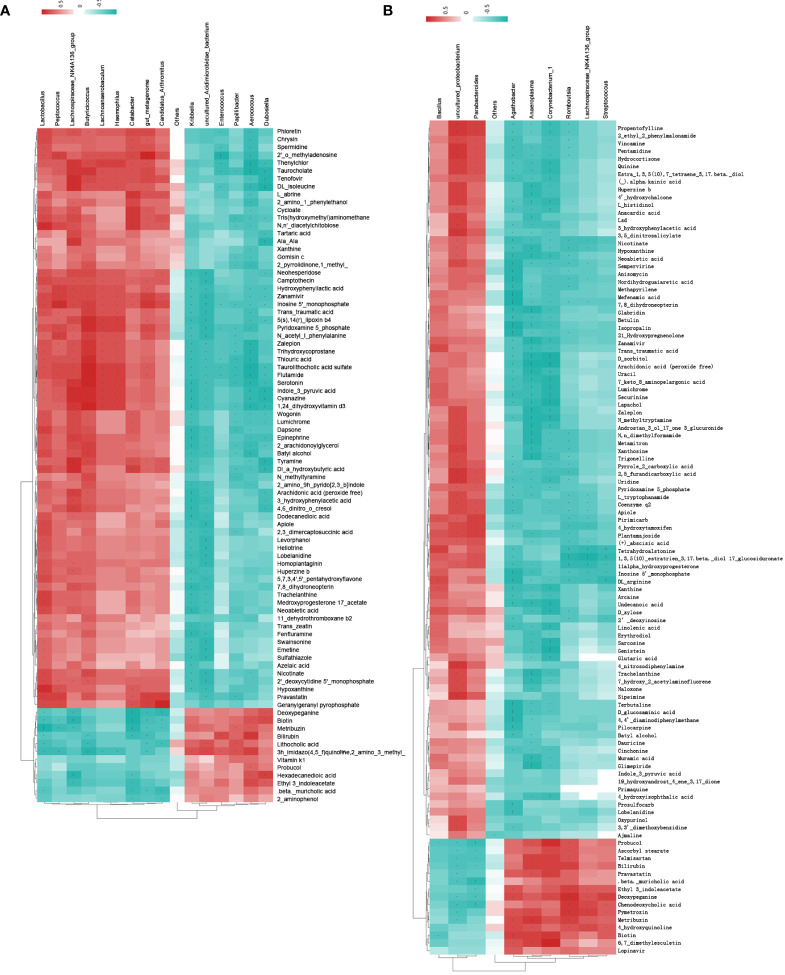
Cluster heat map of spearman correlation analysis between significant difference flora and significant difference metabolites. **(A)** model group and control group; **(B)** treatment group and model group. Red indicates positive correlation, blue indicates negative correlation, and the deeper the color, the stronger the correlation.

In the cluster heat map, each horizontal line represented the genus with significant difference, and each vertical column represented the metabolite with significant difference. The left branch represents the result of clustering the differential bacteria, and the upper branch represents the result of clustering the differential metabolites (**Figures 5A, B**). The samples of the model group and the control group were clearly distinguished at the metabolomics level and the diversity of the flora. A total of 16 significantly different floras and 86 significantly different metabolites were screened (**Supplementary Table S5**). The samples of the treatment group and the model group also had obvious differences in metabolomics and flora diversity. A total of 10 significantly different floras and 108 significantly different metabolites were screened (**Supplementary Table S6**).

## Discussion

PD is a common and challenging gynecological disease among women of childbearing age, which has a great negative impact on health-related quality of life and productivity (18). The clinical treatment of PD emphasizes the principle of starting as early as possible, adhering to the long-term, standardized and continuous treatment, and achieving and maintaining the best control state (19, 20). However, at present, the treatment of PD in modern medicine is mostly symptomatic treatment, and there is no specific drug. Western medicine analgesic treatment can only alleviate the clinical symptoms, cannot be completely cured, cannot achieve the desired therapeutic effect (21–24). The treatment of PD with traditional Chinese medicine has a rich theoretical system and clinical experience, and it is regarded as an advantageous disease in the treatment of traditional Chinese medicine (25, 26). ‘Traditional Chinese medicine gynecology ‘ divides dysmenorrhea into five types: qi stagnation and blood stasis, cold coagulation and blood stasis, damp heat and blood stasis, qi and blood deficiency, and liver and kidney loss (18). GWUAP was selected for the National Key Research and Development Program, and most of its drugs belong to the category of warming the heart, tonifying the blood and activating blood circulation and removing blood stasis, which have the efficacy of warming the uterus, dispersing cold, removing blood stasis and regulating menstruation to relieve pain. This study relies on the own advantages of acupoint patch therapy, using modern Chinese medicine purification technology, adding excipients with transdermal effect, so that the efficacy of the drug with acupoint stimulation to further achieve the therapeutic effect of drug-point unity and synergistic effect (27). And in the traditional acupuncture theory, the acupoints are used as reaction points for disease diagnosis and stimulation points for acupuncture treatment. “Guanyuan, Zigong and Sanyinjiao, as the intervention points of the patch, have certain specificity and are commonly used as empirical points for the clinical treatment of dysmenorrhea (28, 29).

The PD rat model was made by subcutaneous injection of estradiol benzoate combined with oxytocin. This method has a high utilization rate and success rate in animal experiments of dysmenorrhea. Based on the preliminary work of the experimental group, a lot of experience was obtained for the successful modeling. In this experiment, after copying the PD rat model, there was a 100% incidence of writhing and a more obvious writhing reaction (abdominal contraction concave, hip elevation, extension of hind limbs, body distortion), which was in line with the clinical characteristics of PD, and the model was successfully constructed. In the treatment group, the frequency of pain was significantly reduced after GWUAP intervention, the degree of pain was relieved, and the pain state was greatly improved. In addition, the pathological morphology of the uterine tissue of the rats in the treatment group was improved and the pathological injury score was significantly reduced, which also provided evidence support for the treatment plan of this experiment and provided help for the follow-up study of microbiota and metabolomics.

Studies have shown that a variety of pathogenesis are involved in the occurrence and development of primary dysmenorrhea. For example, the intervention of inflammatory mediators, low immune system, secretion of analgesic factors, secretion of hormones, etc. (30). PGF2α is a vasoconstrictor substance and a pain-causing substance. Its overexpression can stimulate the contraction of uterine muscles and blood vessels, reduce the blood supply of the uterus, and make the uterus in a state of hypoxia and ischemia, thereby aggravating dysmenorrhea symptoms (31). β-EP is a neuropeptide widely distributed in uterine cavity fluid and endometrium. It is an inhibitory transmitter that regulates the pain pathway and has morphine-like activity. The occurrence and outcome of dysmenorrhea are closely related to the rise and fall of β-EP (32, 33). In this experiment, the content of β-EP in PD rats decreased, so that the activity of β-EP was low, which would further aggravate the pain and form a positive feedback loop of pain. TNF-α is a common clinical inflammatory factor, and its excessive increase can increase the concentration of PGF2α in serum of patients with dysmenorrhea, thus mediating the occurrence of PD (34). As a sex hormone index, E2 can cause uterine muscle spasm, ischemia and pain when the secretion is abnormal. The decrease of its content can effectively alleviate uterine spasm and vasoconstriction, and play an analgesic effect (35). The results of this experiment showed that GWUAP had a good regulatory effect on inflammatory factors TNF-α, analgesic factors PGF2α and β-EP, and sex hormone index E2.

As the ‘ second genome ‘ of the human body, intestinal flora can resist pathogens, improve the intestinal immune system and human metabolism. Its metabolic potential explains its significance in host health and disease (36, 37). Studies have found that the occurrence and development of PD are related to the decrease of human immunity, the increase of inflammatory factors, the abnormal secretion of hormones and other factors, but there are relatively few studies on whether it is related to flora (38, 39). The results of this study showed that GWUAP could affect the OTU composition of intestinal flora in PD rats, regulate the diversity of flora, and screen out multiple differential flora related to treatment. Lactobacillus is a major probiotic that plays an important role in regulating body metabolism and immunity. It can inhibit smooth muscle contraction, reduce cholesterol, reduce oxidative stress, inflammation and cell death, and can also reduce histopathological damage caused by inflammation (40). Itoh, H (41). found that the tablets containing Lactobacillus gasseri OLL2809 are effective for endometriosis, especially for dysmenorrhea, which can improve NK cell activity, reduce VAS and VRS scores, improve pain intensity, and have a certain analgesic effect. In addition, the increased content of Lactobacillus in the treatment of atherosclerotic diseases can reduce the expression of inflammatory factors and improve the disorder of intestinal flora (42). Romboutsia is a short-chain fatty acid-producing bacterium that plays a role in anti-inflammatory immunity and regulation of metabolism. The abundance of this genus is often significantly negatively correlated with various host physiological dysfunctions (such as diabetes, obesity, and ulcerative colitis) (43). Studies have also shown that the abundance of Romboutsia in the intestinal flora is significantly reduced when Cepharanthine improves ulcerative colitis. Among them, the abundance of Romboutsia is positively correlated with the expression level of pro-inflammatory factors (44). Combined with the results of this experiment, it was proved that the abundance of Lactobacillus in the intestinal flora decreased, the abundance of Romboutsia increased, and the inflammatory factors in the serum increased significantly in the model group after estradiol benzoate modeling. After GWUAP treatment, the abundance of Lactobacillus in the intestinal flora increased, the abundance of Romboutsia decreased, and the inflammatory factors in the serum decreased significantly. Therefore, GWUAP can exert analgesic effect and play a therapeutic role in primary dysmenorrhea by regulating the abundance of bacteria, inhibiting uterine smooth muscle contraction, reducing inflammatory response and improving the activity of immune cells.

More and more evidence shows that there is a correlation between intestinal flora imbalance, estrogen increase and dysmenorrhea. Intestinal dysbacteriosis refers to disturbances in the diversity or number of gut microbiota, which may occur through diet, age, ethnicity, medication, smoking, and alcohol consumption (45). Studies have shown that supplementation of probiotics has a beneficial effect in reducing dysmenorrhea and symptom severity in patients with endometriosis. And in two randomized controlled trials, the effect of Lactobacillus on endometriosis symptoms, compared with placebo, Lactobacillus group menstrual pain significantly reduced (46, 47). Intestinal flora regulates estrogen metabolism through estrogen, which can treat gynecological diseases such as dysmenorrhea and endometriosis (48). In view of the potential of probiotic supplements in reversing intestinal flora imbalance and restoring estrogen homeostasis, increasing the abundance of Lactobacillus can be used to treat gynecological diseases, especially those with inflammatory origin and estrogen-driven diseases, such as primary dysmenorrhea (49). Different genera have certain positive effects on inflammation. For example, Lactobacillus with increased relative abundance and Romboutsia with decreased relative abundance can reduce the expression level of inflammatory marker TNF-α (50, 51). As a cytokine, TNF-α plays an important role in the function of endometrium, and the decrease of its expression level can significantly improve the pathological damage of uterine tissue (52, 53). In this study, after GWUAP treatment, Lactobacillus and Romboutsia can be adjusted, thereby reducing the expression of TNF-α, PGF2α, E2, increasing the expression of β-EP, and improving the pathological damage of rat uterine tissue, confirming the protective effect of GWUAP on PD rats.

Non-targeted metabolomics can detect all metabolites in the sample in an unbiased, large-scale and holistic manner, and comprehensively reflect the changes of metabolites in the organism. It is often used as an important means of basic research in modern medicine. It has unique advantages in the development and verification of disease diagnostic markers and the study of metabolic-related signaling pathway mechanisms (32, 54). Studies have shown that the occurrence and development of dysmenorrhea is closely related to non-targeted metabolomics (55). In this study, 32 biomarkers related to treatment were found and identified by OPLS-DA and T-test analysis, and three important metabolic pathways were enriched: Steroid hormone biosynthesis, Prostate cancer and Biotin metabolism. However, this experiment did not explore the related biomarkers and metabolic pathways in depth, which also provided a direction for subsequent research.

Intestinal flora is a microbial population that lives in the human intestinal tract. Under normal circumstances, it is beneficial and harmless to the human body. It is an ecological system that maintains a dynamic balance with the host and the external environment. If it is out of balance, it will induce various diseases. The decryption of intestinal flora must be inseparable from the topic of metabolomics. Because metabolites bear the life activities in cells and provide the terminal information of biology, they are the media for the interaction between many hosts and intestinal bacteria. The correlation analysis of intestinal flora and metabolites in this study will help us to reveal the possible mechanism of the association between intestinal bacteria and hosts (56, 57). The results showed that Lactobacillus and Romboutsia were significantly regulated by GWUAP. Correlation analysis showed that Lactobacillus was closely related to 12 differential metabolites (*P* < 0.05), and Romboutsia was closely related to 45 differential metabolites (*P* < 0.05). It is suggested that GWUAP may regulate the intestinal flora and change the metabolites related to the intestinal flora, so as to play an anti-inflammatory, analgesic and regulating hormone levels, and finally achieve the effect of treating PD.

In this study, modern advanced technology was used to explore the effect of GWUAP on PD rats by combining intestinal flora and non-targeted metabolomics. It was found that GWUAP could not only improve the imbalance of intestinal flora in PD rats, but also play an anti-inflammatory and analgesic role by regulating Steroid hormone biosynthesis, Prostate cancer and Biotin metabolism pathways. At the same time, the biomarkers on the three metabolic pathways were analyzed. After treatment, metabolites were significantly enriched in these three pathways. Cortisol, a hormone type on the Steroid hormone biosynthesis pathway, is significantly reduced in patients with dysmenorrhea and is negatively correlated with the duration of symptoms (58). Estradiol on the Prostate cancer pathway is generally used as an inducing factor for the manufacture of dysmenorrhea animal models, and its concentration is positively correlated with the risk of dysmenorrhea (59). Biotin on the Biotin metabolism pathway has a strong analgesic effect and is closely related to the occurrence of dysmenorrhea (60). Therefore, we speculate that GWUAP treatment of PD can have a therapeutic effect through the metabolites of Cortisol, Estradiol and Biotin in these three pathways. This also shows that GWUAP has a multi-target therapeutic effect, and the results can provide a theoretical basis for the further development and utilization of GWUAP.

## Data availability statement

The data presented in this study can be found in online repositories. The name of the repository and the accession number can be found here: https://www.ncbi.nlm.nih.gov/sra, accession number PRJNA1115957.

## Ethics statement

The animal study was approved by the Ethics Committee of Changchun University of Chinese Medicine (Approval No. 2022005). The study was conducted in accordance with the local legislation and institutional requirements.

## Author contributions

LW: Writing – original draft, Data curation. TL: Writing – original draft, Project administration, Methodology. W-XC: Writing – review & editing, Investigation. J-YZ: Writing – review & editing, Validation, Project administration. X-HX: Writing – review & editing, Resources, Funding acquisition. J-PC: Writing – review & editing, Validation, Formal analysis. J-XZ: Writing – review & editing, Software, Conceptualization. JL: Writing – review & editing, Visualization, Supervision. F-CW: Writing – review & editing, Visualization, Validation, Supervision, Resources, Funding acquisition.
