# Supplementary material for: To explore the mechanism of acupoint application in the treatment of primary dysmenorrhea by 16S rDNA sequencing and metabolomics
Source: Front Endocrinol (Lausanne). 2024 May 30;15:1397402. doi: 10.3389/fendo.2024.1397402 (PMC11169635; doi:10.3389/fendo.2024.1397402)
Supplement: Supplementary file 1 [file Table_1.docx]

Table S1: The model evaluation parameters (R^2^Y, Q^2^)

| **A** |  |  |  |  |
| --- | --- | --- | --- | --- |
| **sample grouping** | **A** | R2X（cum） | R2Y（cum） | Q2（cum） |
| control vs model | **1** | **0.621** | **0.997** | **0.627** |
| control vs treatment | **1** | **0.339** | **0.999** | **0.649** |
| model vs treatment | **1** | **0.612** | **0.999** | **0.711** |
| **B** |  |  |  |  |
| **sample grouping** | **A** | R2X（cum） | R2Y（cum） | Q2（cum） |
| control vs model | **1** | **0.684** | **0.998** | **0.79** |
| control vs treatment | **1** | **0.36** | **0.999** | **0.751** |
| model vs treatment | **1** | **0.637** | **0.999** | **0.796** |
| Table 1. (A)The evaluation parameters of positive ion mode OPLS-DA model;(B)Evaluation parameters of negative ion mode OPLS-DA model.A : represents the principal component fraction；R2X：Represents the explanatory rate of the model to the X variable.；R2Y：Representing the explanatory rate of the model to the Y variable.；Q2：Represents the predictive ability of the model。 | | | | |
|  |  |  |  |  |
|  |  |  |  |  |
|  |  |  |  |  |
|  |  |  |  |  |
|  |  |  |  |  |
