# Supplementary material for: To explore the mechanism of acupoint application in the treatment of primary dysmenorrhea by 16S rDNA sequencing and metabolomics
Source: Front Endocrinol (Lausanne). 2024 May 30;15:1397402. doi: 10.3389/fendo.2024.1397402 (PMC11169635; doi:10.3389/fendo.2024.1397402)
Supplement: Supplementary file 2 [file Table_2.docx]

Table S2: Model vs Control Volcano Plot

| **adduct** | **Name** | **VIP** | **Fold change** | **p-value** | **m/z** | **SuperClass** | **Control-1** | **Control-2** | **Control-3** | **Control-4** | **Model-1** | **Model-2** | **Model-3** | **Model-4** | **Treatment-1** | **Treatment-2** | **Treatment-3** | **Treatment-4** |
| --- | --- | --- | --- | --- | --- | --- | --- | --- | --- | --- | --- | --- | --- | --- | --- | --- | --- | --- |
| [M+H]+ | 6-oxirane boldenone | 2.078417931 | 5.990097399 | 1.1417E-05 | 315.19140 | Lipids and lipid-like molecules | 23481155.62 | 48803162.03 | 11607048.58 | 19783339.85 | 168460164.5 | 143771995 | 160027077.2 | 148762350.6 | 18370956.74 | 16971980.56 | 18393645.53 | 33723841.24 |
| [M+H]+ | Arachidonoylserotonin | 1.412240795 | 9.473413917 | 1.50824E-05 | 463.35332 | Organoheterocyclic compounds | 1879168.375 | 13180819.71 | 3192195.844 | 9907000.383 | 72597188.46 | 58334478.73 | 73937964.26 | 61893977.1 | 11771719.98 | 36168663.32 | 5702658.447 | 13961391.76 |
| [M+H]+ | Deoxypeganine | 2.700546829 | 7.89785277 | 0.00011268 | 173.09239 | Organoheterocyclic compounds | 32770075.34 | 46065484.39 | 21892339.44 | 27024003.61 | 232838342.7 | 301667042.6 | 280649622.2 | 193810711.7 | 25152825.94 | 25568759.26 | 25794273.19 | 24113762.58 |
| [M+H]+ | Metribuzin | 1.78811912 | 3.315878398 | 0.000163306 | 215.10280 | Organosulfur compounds | 46640526.28 | 47571373.51 | 40015136.02 | 33852478.81 | 170951088.1 | 129322724.2 | 118926178 | 138131241.3 | 44626551.48 | 50092707.12 | 50947228.88 | 40917196.96 |
| [M-H]- | 2,3-quinoxalinedione, 1,4-dihydro-6,7-dinitro- | 1.673791597 | 0.406047532 | 0.000166622 | 251.02325 | Organoheterocyclic compounds | 189451686.4 | 143707069 | 172022044 | 157612096.2 | 67822771.39 | 82906248.89 | 68196484.84 | 50199914.5 | 41037223.06 | 67601214.81 | 87764234 | 50301499.48 |
| [2M-H]- | 3-phosphonoalanine | 5.928300881 | 0.174227002 | 0.000187742 | 337.03882 | Organic acids and derivatives | 1698840495 | 1622339266 | 1246390226 | 1208218924 | 207496191.4 | 273000049.4 | 443068866.5 | 82733277.97 | 1192817560 | 891883330.4 | 3581562553 | 3106716905 |
| [M+H]+ | Biotin | 2.848674785 | 3.351353564 | 0.000278958 | 245.11328 |  | 105396353.6 | 158176512.3 | 75412878.94 | 76208614.8 | 405744793.3 | 367893647.8 | 333494428 | 284330227.7 | 94906308.83 | 73488665.62 | 88953957.75 | 83746705.08 |
| [M-H]- | Flutamide | 2.343709113 | 0.636397282 | 0.000324795 | 275.05953 | Benzenoids | 597150213.9 | 651754183.3 | 573928138.6 | 528852247.4 | 353901418.8 | 415116924.6 | 365434262.4 | 362153199.1 | 340359629.1 | 372449701.5 | 578596888.3 | 368842410.5 |
| [M-H]- | Camptothecin | 3.651927086 | 0.442434073 | 0.000378734 | 347.11684 |  | 924891599.4 | 801024183.6 | 887513281.6 | 865479655.2 | 435485660.2 | 505990341.8 | 385875191.8 | 211836559.8 | 352456942.9 | 582690976.7 | 656160354.7 | 503323436.3 |
| [2M-H]- | 3.alpha.-hydroxy-7-oxo-5.beta.-cholanic acid | 5.847416271 | 7.733198331 | 0.000538229 | 779.54657 | Lipids and lipid-like molecules | 87352538.08 | 331532878 | 153378432.8 | 68366666.56 | 1031474544 | 1259850908 | 1653506788 | 1009290592 | 605670493.2 | 690085179.1 | 304703069.5 | 423648372.6 |
| [M-H]- | Pravastatin | 5.203736236 | 0.36348105 | 0.000593737 | 423.25722 | Organic acids and derivatives | 1321025522 | 1719849607 | 1488662705 | 1562142390 | 742756798.6 | 731005498.3 | 518700753.1 | 221747273.4 | 14598416.61 | 24043346.73 | 3679309.286 | 72318003.37 |
| [M-H]- | Pyridoxamine 5-phosphate | 2.078380718 | 0.288972851 | 0.000595217 | 247.06462 | Organoheterocyclic compounds | 236901329 | 238205243.7 | 220618249.2 | 143270203.3 | 58991380.18 | 68888032.31 | 58331252.34 | 56236119.25 | 167190867.2 | 154972487.8 | 271147179.7 | 140557253.1 |
| [M-H]- | Donepezil | 1.285476293 | 0.388263012 | 0.000748856 | 378.20336 | Organoheterocyclic compounds | 111565350.2 | 75528867.62 | 109774475.6 | 87755268.93 | 27924468.73 | 39623174.49 | 43104766.63 | 38682848.19 | 39592437.99 | 31817618.69 | 78121133.23 | 63313563.54 |
| [M+H]+ | Cis-4,10,13,16-docosatetraenoic acid methyl ester | 1.842300643 | 0.422857573 | 0.001036953 | 347.30586 | Lipids and lipid-like molecules | 251268056.5 | 206194506.1 | 246692290.8 | 188382888.3 | 71889129.9 | 90682450.65 | 138904494.6 | 75940268.05 | 147643153.6 | 177320717.7 | 107511403 | 167964975.9 |
| [M-H]- | Taurocholate | 6.377719297 | 0.122774407 | 0.001385943 | 514.28437 | Lipids and lipid-like molecules | 1669762198 | 1988539273 | 1771359892 | 883515709.2 | 76747552.14 | 122046000.4 | 263268189.4 | 313034827 | 240599881 | 274778907.1 | 514231439.8 | 192985671.7 |
| [M+Na]+ | N,n'-diacetylchitobiose | 1.145019134 | 0.224888444 | 0.001395879 | 447.15894 | Organic oxygen compounds | 60811160.75 | 40104871.28 | 64370301.51 | 75192405.56 | 2186633.397 | 17217697.02 | 20238987.02 | 14437572.09 | 43679034.5 | 16333183.73 | 46416336.32 | 32148886.2 |
| [M+H-CH5O4P]+ | Tenofovir | 2.546500843 | 0.521582798 | 0.001575332 | 176.09189 | Organoheterocyclic compounds | 541178470.8 | 471944033.6 | 630257927.7 | 439781895.2 | 282069883.5 | 272910725.6 | 224072273.8 | 307488753.4 | 139117112.2 | 318856822.4 | 237286498.9 | 312721866.1 |
| [M+H-H2O]+ | Cholesta-4,6-dien-3-one | 3.36764044 | 0.64289282 | 0.001925167 | 365.31636 | Lipids and lipid-like molecules | 1377402330 | 1283153024 | 1143228902 | 1257041911 | 615602356.6 | 943551796 | 884419114.8 | 809995540 | 1000402495 | 1162656157 | 900701420.7 | 1213642190 |
| [M-H]- | 1,4-bis[(p-hydroxyphenethyl)amino]anthraquinone | 1.948102197 | 0.052915378 | 0.002251095 | 477.17266 | Benzenoids | 197537313.9 | 141278271.8 | 71891950.5 | 154211918.9 | 4736878.744 | 3371442.784 | 3698074.021 | 18086531 | 21468597.73 | 11654453.07 | 30603860.86 | 17856811.39 |
| [M+H]+ | 3,4-dimethoxychalcone | 3.639734545 | 11.02369904 | 0.002340878 | 269.11346 | Phenylpropanoids and polyketides | 35025219.71 | 80275685.91 | 26211253.02 | 29834514.07 | 337873588.3 | 593583964.4 | 640093740.1 | 317322858.6 | 25604393.09 | 23917275.29 | 26012041.8 | 19653329.17 |
| [2M+H]+ | Dapsone | 1.242033248 | 0.273549036 | 0.00245707 | 497.14824 | Benzenoids | 73455644.73 | 58732174.17 | 69224500.48 | 101074168.3 | 9394336.046 | 38388923.19 | 15293246.52 | 19668381.3 | 31244497.61 | 37427278.38 | 28401964.53 | 70553947.84 |
| [M+Li]+ | Pinanethromboxane a2 | 2.924364747 | 0.617035203 | 0.002723513 | 383.32692 | Lipids and lipid-like molecules | 1009118740 | 909084720.3 | 751298181.8 | 925715555.4 | 429399043.4 | 630008929.7 | 610866221.4 | 548101380.6 | 671994853.4 | 804211636.6 | 636499523.1 | 807984053.3 |
| [M-H-H2O]- | Mollicellin i | 1.230634382 | 0.450774367 | 0.003227233 | 351.14820 |  | 98686404.7 | 114314240.6 | 114103304.7 | 88781385.6 | 56921842.1 | 67357565.62 | 43717737.14 | 19473303.94 | 60062922.29 | 137799856.9 | 122818655.9 | 74915163.23 |
| [M+H]+ | Spermidine | 1.414810933 | 0.43619501 | 0.003239233 | 146.15398 | Organic nitrogen compounds | 185230317 | 124673874.6 | 113589536.8 | 129338658.6 | 65074308.93 | 60393481.41 | 66465530.1 | 49209407.83 | 36854184.21 | 119251078 | 102713345 | 50520360.53 |
| [M-H]- | Trans-traumatic acid | 3.105813894 | 0.386889579 | 0.00331902 | 227.12898 | Lipids and lipid-like molecules | 731668420.8 | 623438025.5 | 417124457.1 | 542561363.3 | 208996467.3 | 329224541.5 | 185954400 | 171393595.9 | 349473792.6 | 509677505.7 | 558366605 | 406043025 |
| [M+H]+ | L-propionylcarnitine | 1.217964681 | 0.527468048 | 0.003527731 | 218.13875 | Lipids and lipid-like molecules | 127164333.7 | 133183801 | 135336236.4 | 96960161.89 | 75166991.17 | 84363060.39 | 52916001.11 | 47408197.78 | 124002889.3 | 130562651.1 | 137250894.2 | 136897651.5 |
| [M-H-H2O]- | Neohesperidose | 3.298999135 | 0.368039266 | 0.003597999 | 307.08578 | Lipids and lipid-like molecules | 837447632.6 | 435514848.3 | 680648091.2 | 590094010.1 | 252502521.3 | 286729260.2 | 191481807.6 | 205469579.4 | 161837345.6 | 279550616.9 | 203641530.5 | 357548414 |
| [M+H]+ | 1,3-benzenediol, 5-methyl-4-[(1r,6r)-3-methyl-6-(1-methylethenyl)-2-cyclohexen-1-yl]- | 2.224489039 | 0.425652904 | 0.003699187 | 259.17662 | Lipids and lipid-like molecules | 348341412.4 | 271551980.1 | 451882039.4 | 337863594.6 | 132183500.6 | 98352790.46 | 209157771.6 | 160322882.8 | 56611147.99 | 68698666.32 | 290396072.8 | 169915088 |
| [M-H]- | Zanamivir | 2.975288875 | 0.415206512 | 0.003857883 | 331.12218 | Organic acids and derivatives | 641278475.4 | 583424571 | 687248807.7 | 408114513.1 | 287892391.1 | 304260227.2 | 253721776.6 | 117432270 | 317820847.8 | 402314824.1 | 476728009.9 | 508437244.7 |
| [M-H]- | Bilirubin | 1.795127236 | 3.38251593 | 0.004043964 | 583.25840 | Organoheterocyclic compounds | 46094824.46 | 24297495.94 | 57044757.86 | 43633705.7 | 137658362.5 | 205720955.5 | 104986038.2 | 130284295.8 | 50510334.32 | 89498200.6 | 50285281.42 | 45969319.18 |
| [M-H]- | Inosine 5'-monophosphate | 1.340998173 | 0.380835305 | 0.004083268 | 347.02623 | Nucleosides, nucleotides, and analogues | 97546369.78 | 121949179.2 | 125797240.9 | 83609739.52 | 44597246.51 | 66570627.7 | 37958286.92 | 14215064.28 | 92349264.02 | 133239424.7 | 91122071.37 | 97876985.63 |
| [M-H]- | Dl-Leu-dl-Val | 2.565310085 | 0.529389478 | 0.00461419 | 229.15587 | Organic acids and derivatives | 615969515.7 | 453381339.1 | 559043264.7 | 581607510.2 | 235848888.5 | 392803886.5 | 351622692.5 | 189676141.2 | 220020856 | 289020988.3 | 250969053 | 263995492.1 |
| [M-H-CH3]- | Wogonin | 2.27090374 | 0.453332969 | 0.004630344 | 268.04981 | Phenylpropanoids and polyketides | 410070525.1 | 240042684.9 | 334663039.1 | 413378299.2 | 131045115.5 | 173293305.6 | 135085750.2 | 194405381.5 | 132388139.2 | 164620071.8 | 300819364.1 | 134260080.6 |
| [M-2H]2- | Taurolithocholic acid sulfate | 4.352514936 | 0.566806083 | 0.005048645 | 280.62213 | Lipids and lipid-like molecules | 1855204173 | 2029293713 | 1768857087 | 1425914593 | 715669194.3 | 1231586126 | 1191729986 | 873587746.5 | 1269020119 | 1396430926 | 170802518.9 | 824090686 |
| [M-H-H2O]- | 5(s),14(r)-lipoxin b4 | 1.332633886 | 0.476832031 | 0.005176885 | 333.20727 | Lipids and lipid-like molecules | 166260751.7 | 125334012.6 | 112698884.5 | 104040422.6 | 56454770.09 | 81708651.15 | 50343486.76 | 53883059.88 | 83828069.81 | 187387410.3 | 100978640.9 | 80122708.74 |
| [M-H]- | Mef-glu | 2.408621947 | 0.443838241 | 0.005577729 | 369.13763 |  | 473521210.3 | 455375290.2 | 426947367.7 | 251587957.2 | 197428529.7 | 170817770.5 | 140937008.2 | 204256405.4 | 167566372.9 | 283334426.4 | 273344138.8 | 190943445.8 |
| [M+H-2H2O]+ | Trihydroxycoprostane | 2.247440632 | 0.668971549 | 0.005599158 | 385.34279 | Lipids and lipid-like molecules | 728688897.1 | 701587300.8 | 560637306.3 | 676339801.9 | 356367909.2 | 523665520 | 486669884.2 | 417613263.2 | 611552382.3 | 708932911.5 | 444835376.5 | 711140087.6 |
| [M+H-2H2O]+ | .beta.-muricholic acid | 3.310542563 | 2.255980064 | 0.005606569 | 373.27392 | Lipids and lipid-like molecules | 191015051.6 | 419478626.8 | 382137943.8 | 159258185.9 | 526486409.9 | 765383596.1 | 715582034.6 | 591188402.2 | 378238220 | 601125363.1 | 256244215.5 | 323071540.7 |
| [M+H]+ | Serotonin | 2.395497255 | 0.492352716 | 0.006186192 | 177.10162 | Organoheterocyclic compounds | 442007332.4 | 493484173.2 | 363023014.8 | 348821166.2 | 133218260 | 270427970.9 | 142524288 | 264899680.7 | 205866083.6 | 207126491 | 699185759.4 | 202330147.9 |
| [M-H]- | Phloretin | 1.980580796 | 0.265894438 | 0.006531508 | 273.08042 | Phenylpropanoids and polyketides | 287567598.8 | 123367328.6 | 212119508 | 187913028.3 | 38975338.16 | 57406661.38 | 90412658.88 | 28837079.61 | 61348992.05 | 93965694.91 | 161397814.7 | 105633471.5 |
| [M-H]- | Dl-a-hydroxybutyric acid | 2.404173727 | 0.320328025 | 0.006913757 | 103.03997 | Organic acids and derivatives | 282609454 | 209927498.3 | 429247739.2 | 334341815.3 | 39286921.04 | 115749720.3 | 83878466.36 | 163457415.7 | 70791762.25 | 142219448.1 | 271881595.4 | 182738573.6 |
| [M-H]- | 5-hydroxy-3,4-dihydro-2(1h)-quinolinone | 5.539804498 | 0.544422276 | 0.007570826 | 162.05606 | Organoheterocyclic compounds | 2970834153 | 1953986873 | 2344153108 | 2804011812 | 856657143.7 | 1638562167 | 1415339879 | 1573398743 | 1687486978 | 1781590222 | 1730436037 | 2765466665 |
| [M-H]- | Lumichrome | 3.13991601 | 0.383112019 | 0.007627829 | 241.07311 | Organoheterocyclic compounds | 707392451.6 | 341191919.6 | 640156153.5 | 733123775.5 | 152037124.1 | 245631596.5 | 239001616 | 291174986.1 | 325543025 | 446405450 | 533597552.6 | 350488067.1 |
| [M-H]- | 2,3-dimercaptosuccinic acid | 2.217460699 | 0.533215862 | 0.007784831 | 180.98125 | Lipids and lipid-like molecules | 459050329 | 447765677.7 | 329533982.3 | 464047385.7 | 202544938.9 | 329585642.8 | 232686393.6 | 141861876.8 | 304860016.6 | 252804672.3 | 392713324 | 428124523.2 |
| [M+H]+ | Fluticasone propionate | 1.147389008 | 0.033253143 | 0.008369977 | 501.16949 | Lipids and lipid-like molecules | 65610370.39 | 43519905.13 | 20167124.75 | 79935524.67 | 1845104.531 | 334256.3272 | 509783.0688 | 4268508.444 | 4697279.406 | 2923583.636 | 8360906.099 | 6079169.352 |
| [M+H-H2O]+ | 2-amino-1-phenylethanol | 2.038269258 | 0.565739549 | 0.008521477 | 120.08057 | Organic nitrogen compounds | 479823014.6 | 373152191.9 | 303313666.4 | 480391320.8 | 205109368.4 | 204015811.3 | 249007734.8 | 267801800.2 | 267063036.2 | 262640658.3 | 559666058.5 | 379534943.1 |
| [M-H]- | Trans-3'-hydroxycotinine o-.beta.-d-glucuronide | 1.953438482 | 0.181571127 | 0.00865941 | 367.10514 | Organic oxygen compounds | 108889991.5 | 259957312.7 | 114450942 | 192172142.6 | 20710501.75 | 40350496.29 | 26669341.48 | 34915580.02 | 126431256.9 | 49945928.73 | 245859942.6 | 186622606 |
| [M+H-C2H2O]+ | Zaleplon | 2.70007695 | 0.53002923 | 0.009489399 | 264.10914 | Organoheterocyclic compounds | 846262763.5 | 590199051.7 | 535152801.9 | 538928078.4 | 272928022.4 | 380239037.1 | 375128094.9 | 302365857.8 | 550406625.9 | 508220256 | 770787294.8 | 693262460.9 |
| [M-H]- | Undecanedioic acid | 1.522023727 | 0.572743876 | 0.009599626 | 215.12893 | Lipids and lipid-like molecules | 219850418.3 | 178337107.8 | 226495926 | 222508512.5 | 107302380.9 | 180917855.5 | 79624960.43 | 117378813 | 125674037.8 | 156076496 | 131806674.5 | 159004994.1 |
| [M+H]+ | 14-benzoylaconine | 1.374411068 | 0.345680942 | 0.009774284 | 604.32512 | Lipids and lipid-like molecules | 154983705.9 | 83312656.07 | 99640015.37 | 125662923.2 | 24568612.55 | 50723767.47 | 71243541.39 | 13721521.36 | 39728652.43 | 32661442.04 | 74584625.77 | 63642971.24 |
| [M+H]+ | Thenylchlor | 1.057255507 | 0.366857082 | 0.009844634 | 324.09576 | Benzenoids | 83929774.89 | 65874323.3 | 76279358.75 | 48659977.9 | 5231184.915 | 38399399.28 | 41706104.92 | 15454885.77 | 55591197.42 | 18432358.84 | 68086027.69 | 14134736.04 |
| [M-H-C4H5ON3]- | 2'-deoxycytidine 5'-monophosphate | 7.347665081 | 0.601894471 | 0.010234361 | 194.99689 | Nucleosides, nucleotides, and analogues | 6633338565 | 4768229824 | 5090511674 | 6002139467 | 3394229933 | 4542910375 | 3133123449 | 2468882615 | 4341821902 | 4251040420 | 4865013085 | 4515260028 |
| [M+H]+ | 2,2-bis[hydroxymethyl]-2,2',2''-nitrilotriethanol | 5.55854031 | 0.42580564 | 0.01035659 | 210.11256 | Organic nitrogen compounds | 2707494642 | 1986321103 | 1662678490 | 1837563201 | 737644197.2 | 1439885926 | 940231646.9 | 371314100.7 | 1984582051 | 845425531.5 | 2136197801 | 2529492370 |
| [M-H]- | Thiouric acid | 2.631789373 | 0.512448984 | 0.010400444 | 182.99688 | Organoheterocyclic compounds | 628374834.7 | 728572881.8 | 386386623.9 | 590776455.5 | 267763854.7 | 385454111.3 | 273420022.8 | 269474717.5 | 375432353 | 426084828.8 | 364293850 | 330454329.8 |
| [M-H]- | Leu-Ile | 1.001868392 | 0.633209476 | 0.010413846 | 243.16027 | Organic acids and derivatives | 136920465.8 | 117130979.6 | 102107277 | 157770071 | 70946051.95 | 86330948.91 | 90148115.36 | 77999465.76 | 132292284.9 | 96982966.44 | 100950406.3 | 132437310.2 |
| [M-H]- | 11.alpha.-hydroxyprogesterone .beta.-d-glucuronide | 1.978270513 | 0.580640415 | 0.010424658 | 505.26300 | Lipids and lipid-like molecules | 385135538 | 397731037 | 415336156 | 339150890.1 | 195027628.5 | 319138186.9 | 250998297.9 | 127485531.9 | 302626077 | 416647980 | 392836980.7 | 342554841.3 |
| [M-H-H2O]- | 11-hydroxy-.delta.9-tetrahydrocannabinol | 1.127248355 | 0.636435337 | 0.01080221 | 311.22294 | Organoheterocyclic compounds | 141653141.4 | 136327750.1 | 148470866.8 | 143203307.7 | 71523853.6 | 132044790.5 | 82749871.4 | 76230098.69 | 93826976.08 | 75612418.78 | 86476073.42 | 118372596 |
| [M-H]- | Artocaprin | 1.54540942 | 0.436032958 | 0.010878331 | 435.20550 | Phenylpropanoids and polyketides | 190958740.7 | 176585480.9 | 159737826.3 | 117544306.3 | 61690993.28 | 120977695 | 70938192.05 | 27558662.03 | 215925178.5 | 160576509.2 | 204999575.8 | 150919473.8 |
| [M+H-H2O]+ | Tris(hydroxymethyl)aminomethane | 1.645497736 | 0.409460572 | 0.010982128 | 104.07075 | Organic nitrogen compounds | 108370256.8 | 170967561 | 198475190 | 236031723.8 | 50358528.49 | 65466659.52 | 74009139.95 | 102456944 | 67687854.18 | 159631631.6 | 47704365.29 | 85121622.9 |
| [M+H]+ | Tyramine | 1.134820133 | 0.620740951 | 0.011141582 | 138.09144 | Benzenoids | 125391987 | 120388903 | 129222711.7 | 122565308.3 | 65555673.09 | 80981492.31 | 51021920.64 | 111302312.2 | 75383705.59 | 62654845.63 | 108871898.4 | 63530906.38 |
| [M-H]- | Nicotinate | 3.521067583 | 0.504777241 | 0.012623038 | 122.02478 | Organoheterocyclic compounds | 1426145884 | 925712949.1 | 751403582.1 | 1205573573 | 569058720.4 | 600710137.1 | 534893818.9 | 470339667.4 | 1089388586 | 757154947.5 | 626365846.3 | 1265064265 |
| [M-H]- | Tetradecanedioic acid | 1.164334588 | 0.335692873 | 0.012712768 | 257.17594 | Lipids and lipid-like molecules | 90845636.72 | 49656235.69 | 70668173.06 | 121018267.6 | 18238393.75 | 35975410.09 | 33040351.36 | 24259093.97 | 43556122.9 | 37334384.03 | 26878323.7 | 85814146.32 |
| [M+Na]+ | Ethyl 3-indoleacetate | 1.217620712 | 3.652567889 | 0.013043591 | 226.10882 | Organoheterocyclic compounds | 2925073.353 | 44388280.91 | 19272236.19 | 8834110.484 | 69331507.41 | 99271853.5 | 59030199.75 | 47842017.24 | 32536646.17 | 36066878.52 | 36206762.96 | 40702410.73 |
| [M-H]- | 15-hydroxy-5z,8z,11z,13e,17z-eicosapentaenoic acid | 1.562906863 | 0.477688451 | 0.013164679 | 317.19718 | Lipids and lipid-like molecules | 258425187.6 | 141797595.5 | 164141481.7 | 168338345.5 | 66202033.78 | 108059351 | 98911464.26 | 76830725.83 | 96471047.31 | 87873507.75 | 110980126.7 | 151258625.9 |
| [M+H]+ | N-methyl-l-isoleucine | 4.476158388 | 0.335447045 | 0.013617708 | 146.11756 | Organic acids and derivatives | 1543153788 | 685516538 | 1151290240 | 919890683.3 | 193388525.6 | 482575033.3 | 196620384.2 | 569788452.8 | 1311638609 | 1038855766 | 1245469969 | 1275945059 |
| [M-H]- | 4,2'-dihydroxy-3,4',6'-trimethoxychalcone | 4.955789131 | 1.644453147 | 0.014257095 | 329.10322 | Phenylpropanoids and polyketides | 1254950332 | 1697315220 | 1064183342 | 747756042.1 | 2111573483 | 2117154520 | 1938441234 | 1667342564 | 1147949436 | 1463191182 | 1509369020 | 1574267631 |
| [M-H]- | 1-palmitoyl-2-hydroxy-sn-glycero-3-phospho-(1'-rac-glycerol) | 4.14299155 | 2.454242682 | 0.01485544 | 483.27284 | Lipids and lipid-like molecules | 297857587.9 | 451760688.3 | 262249749.7 | 406811268.7 | 1155945961 | 926658746.9 | 936695013.4 | 462483555.8 | 878337710.5 | 415503058.1 | 362920967.7 | 1875947611 |
| [2M-H]- | N-acetyl-l-phenylalanine | 5.801652402 | 0.495104068 | 0.014977798 | 413.16400 | Organic acids and derivatives | 2641860845 | 3028730777 | 2973512212 | 2000610689 | 1358101196 | 2070379207 | 1339890384 | 501870676.7 | 1473348099 | 1674848921 | 1822302868 | 1723495052 |
| [M-H]- | (2s,3s)-2-(3,4-dihydroxyphenyl)-3,5,7-trihydroxy-6-methyl-2,3-dihydrochromen-4-one | 1.401593721 | 0.51301383 | 0.015318696 | 317.06690 | Phenylpropanoids and polyketides | 197405818.6 | 175941804.7 | 119576946.2 | 217572833.2 | 82442222.83 | 122161155.7 | 105708469.4 | 54183146.08 | 134253597.7 | 168153147.8 | 193251586 | 137644720 |
| [M+H]+ | DL-isoleucine | 4.856246555 | 0.370695045 | 0.015835527 | 132.10200 | Organic acids and derivatives | 1448452285 | 1573274865 | 1722252151 | 789073040.1 | 300608625.6 | 375882147.8 | 380904227.3 | 993680087.3 | 349197386.1 | 643319053.4 | 1608765037 | 876555349.8 |
| [M+H]+ | Trachelanthine | 2.968964525 | 0.510678727 | 0.016153281 | 302.19622 |  | 782325392 | 568926846.4 | 634626237.6 | 798430123.4 | 221501578 | 605686470.5 | 326501068.4 | 268198054.8 | 609705591.1 | 553306642.7 | 794004406.9 | 810518023.7 |
| [M-H]- | Menthyl salicylate | 2.714587069 | 0.429616733 | 0.017059785 | 275.18651 | Benzenoids | 606316950.2 | 303320030.4 | 449614941.9 | 640576012.1 | 124533684.2 | 318194593.7 | 200598049.1 | 215833217 | 199812819.9 | 255850465.3 | 300328465.5 | 251499500.2 |
| [M-H]- | 3',4'-methylenedioxyorobol | 1.453911641 | 0.570764723 | 0.017070754 | 297.02872 | Phenylpropanoids and polyketides | 28424500.56 | 16970147.01 | 23840151.61 | 20414209.77 | 11431352.45 | 17330970.49 | 9674847.43 | 12731321.42 | 19751500.53 | 18805247.8 | 60260992.88 | 32162900.1 |
| [M+H-H2O]+ | Epinephrine | 2.875127365 | 0.551720675 | 0.017098796 | 166.08636 | Benzenoids | 975842191.6 | 556092941.1 | 637164112.8 | 714746890.3 | 320605532.9 | 502705937.4 | 362900229.7 | 404865835.8 | 399509967.7 | 395246435.3 | 939914015.5 | 823425420 |
| [M-H]- | Dodecanedioic acid | 5.270687541 | 0.4977599 | 0.017574485 | 229.14463 | Lipids and lipid-like molecules | 2652077084 | 1365667947 | 2199353410 | 2706094549 | 955086868.6 | 1565863843 | 937744322.1 | 982912616.6 | 743243171.4 | 600503925.9 | 623395745.5 | 990032883.2 |
| [M+H]+ | Echimidine n-oxide | 1.147571209 | 0.343313471 | 0.01860634 | 414.19748 |  | 85743995.34 | 96244644.94 | 45546378.04 | 69188889.98 | 7894204.789 | 33837134.98 | 8917644.602 | 51220330.55 | 11288248.89 | 84203019.89 | 84459768.9 | 152895461.6 |
| [M-H]- | 3'-hydroxyrepaglinide | 7.751288844 | 2.572406225 | 0.018985044 | 467.27774 | Organoheterocyclic compounds | 1172630080 | 1463847589 | 863632093.3 | 1169051008 | 4195236185 | 3380007587 | 2929079558 | 1506654898 | 4731485071 | 2891246363 | 1737380202 | 4786673135 |
| [M+H-CH5N]+ | N-methyltyramine | 2.598466696 | 0.644473922 | 0.019612649 | 121.06494 | Benzenoids | 733750185.6 | 805432614 | 645704359.2 | 766224980.5 | 408942478.9 | 511308439.6 | 313746937.8 | 667916957 | 441897059.3 | 384171966.4 | 692870627.4 | 383609763.5 |
| [M-H]- | Gln-met | 1.698218818 | 0.271199029 | 0.019735081 | 276.08560 | Organic acids and derivatives | 159814424.9 | 102756340.1 | 259504242.8 | 126637385.6 | 43641254.59 | 84292718.97 | 33090041.29 | 14906156.12 | 558377815.8 | 125223995.5 | 325789561 | 302595025 |
| [M-H]- | Lithocholic acid | 17.56062882 | 2.61964385 | 0.019866823 | 375.29030 | Lipids and lipid-like molecules | 7727286773 | 5641793994 | 8073341673 | 6815676419 | 8953055566 | 18296625722 | 26218500699 | 20557972913 | 5535852018 | 18557918503 | 4643125815 | 10696326490 |
| [M-H]- | Ile-Leu | 1.516140066 | 0.668297692 | 0.019945059 | 243.17155 | Organic acids and derivatives | 314508496.1 | 294727259.1 | 323546409.6 | 360874648.3 | 200614219.2 | 291829885.6 | 230083427.3 | 142020330.9 | 112912803.4 | 207292660.9 | 140961933.3 | 164992778.9 |
| [M+H-2H2O]+ | Hexadecanedioic acid | 1.219716436 | 2.454927442 | 0.020143009 | 251.20072 | Lipids and lipid-like molecules | 24528796.28 | 37335903.12 | 26050421.14 | 34734806.6 | 98545764.7 | 86467555.48 | 80910931.34 | 35172420.38 | 39797517.95 | 94723138.44 | 68378371.56 | 59457788.38 |
| [M+H]+ | Heliotrine | 1.622565594 | 0.527888 | 0.020421832 | 314.20753 |  | 269907744.7 | 168235590.9 | 211926926.9 | 218534455.2 | 103044229.8 | 189089452 | 87934959.26 | 78457366.57 | 106741432.1 | 99032697 | 123305628.9 | 115445458 |
| [M+H]+ | (25s)-7-dafachronic acid | 1.065387338 | 0.452903671 | 0.020647469 | 415.31692 | Lipids and lipid-like molecules | 77901972.83 | 82590150.35 | 99468660.38 | 136884414.2 | 23524306.36 | 44514005.62 | 75733445.87 | 35960888.94 | 36377656.31 | 116401207.6 | 129074166.7 | 110452076.3 |
| [M-H]- | 5,7,3',4',5'-pentahydroxyflavone | 1.256282884 | 0.530076288 | 0.020821382 | 301.06007 | Phenylpropanoids and polyketides | 166891628.8 | 90404282.7 | 134151805 | 156541552.9 | 63245503.2 | 106473752 | 69048589.57 | 51708272.81 | 114351175 | 73386630.39 | 140729429.6 | 78483571.97 |
| [M+H]+ | Cycloate | 2.507391923 | 0.663647162 | 0.021148442 | 216.13835 | Organosulfur compounds | 931382141 | 657574097.3 | 1008976762 | 991150892.9 | 438399074.7 | 624374300.8 | 669348284 | 649763678.6 | 703015692.5 | 837579685.1 | 643001983.4 | 868343941.4 |
| [M+H]+ | Ethoxyquin | 1.825984284 | 0.619319106 | 0.021747769 | 218.13866 | Organoheterocyclic compounds | 491841523.6 | 275656077.1 | 405098635.1 | 349705043.2 | 214997916 | 269553647.5 | 229106579.5 | 229132123.9 | 615778705.8 | 599313118.5 | 548910631.6 | 536828015.2 |
| [M-H]- | L-abrine | 1.247508265 | 0.432818092 | 0.022154933 | 217.10821 | Organic acids and derivatives | 170302704.1 | 79987401.98 | 113099522 | 176161004.6 | 71983282.82 | 33406337.28 | 73265225.38 | 54872429.78 | 96669236.36 | 62370539.78 | 111142134.2 | 46356691.96 |
| [M-H]- | Probucol | 4.444081771 | 1.970484899 | 0.022351726 | 515.30490 | Benzenoids | 330418835.3 | 936901681.8 | 1000304860 | 402033692.1 | 1308345619 | 1554620681 | 1407718081 | 989838498.9 | 467646171.1 | 560246883.6 | 310723786.1 | 420865412.6 |
| [M+H-H5ON]+ | Dl-5-hydroxylysine | 1.080178982 | 0.512048143 | 0.024218548 | 128.08196 | Organic acids and derivatives | 114466172.8 | 69851567.33 | 135028954.1 | 106722602.4 | 64194248.99 | 80238122.11 | 39987540.1 | 33748080.93 | 93418965.57 | 84983280.88 | 97927280.2 | 86192063.54 |
| [M-H]- | 2'-o-methyladenosine | 5.206165701 | 0.362361155 | 0.024794093 | 280.12438 | Nucleosides, nucleotides, and analogues | 1266253971 | 2503752736 | 1858352391 | 1169415469 | 684586491.2 | 534716309.9 | 1090955550 | 152991089.9 | 387716586.8 | 421677715.6 | 927851140.3 | 536959508.5 |
| [M-H]- | Azelaic acid | 4.404110858 | 0.543915962 | 0.024927659 | 187.09763 | Lipids and lipid-like molecules | 2052849475 | 1029836062 | 1883211772 | 2166743950 | 757935466.8 | 1133765402 | 873692497.3 | 1114164069 | 973493462.1 | 906750907 | 1407713550 | 1247313783 |
| [M-H-CO2]- | N-(4-hydroxyphenyl)glycine | 3.288225866 | 0.530289235 | 0.025815627 | 122.03634 | Organic acids and derivatives | 1471678604 | 858182649 | 736828319.9 | 1212728808 | 600112913.6 | 622792897.5 | 568058203.8 | 478365483.2 | 93435622.67 | 704485906.2 | 618253471.9 | 120543448.4 |
| [M+H]+ | Fenfluramine | 1.162022219 | 0.552644824 | 0.027939877 | 232.11801 | Benzenoids | 127852202.4 | 116685310.3 | 113227746.7 | 123114761.6 | 42030056.19 | 119519172 | 41545802.34 | 62660824.24 | 91019038.97 | 83560320.37 | 100653342.6 | 150150458.8 |
| [M-H]- | Benzenepropanoic acid, 5-(3-carboxybenzoyl)-2-[[(5e)-6-(4-methoxyphenyl)-5-hexen-1-yl]oxy]- | 2.608924062 | 12.45911068 | 0.028199316 | 501.21637 | Benzenoids | 15509890.17 | 13253976.62 | 11704675.85 | 41467876.64 | 172601990.6 | 370290427.7 | 410355592.7 | 67606905.28 | 107976027.9 | 94378244.62 | 38679091.52 | 169215736 |
| [M-H]- | Pglu-glu-pro-amide | 1.055062794 | 0.544660746 | 0.028883749 | 353.12743 | Organic acids and derivatives | 134084632.5 | 97647050.65 | 75586502.02 | 97979381.96 | 48290462.85 | 86774607.03 | 45763321.88 | 39921283.57 | 62827176.48 | 55525925.81 | 79346670.52 | 84297791.03 |
| [M-H]- | Tartaric acid | 2.931576632 | 0.290457771 | 0.028984887 | 149.02732 | Organic oxygen compounds | 627523675.1 | 174057103.2 | 462773732.5 | 523251508.1 | 20929352.08 | 143297748.7 | 81899909.37 | 273097049.8 | 38307392.56 | 253442202.9 | 380294781.7 | 686864531.7 |
| [M+H]+ | Levorphanol | 4.574914684 | 0.652212526 | 0.029155442 | 258.20638 |  | 3174126828 | 2020121073 | 2492229942 | 2798705787 | 1614317373 | 2315936666 | 1503590008 | 1404724050 | 2153701179 | 2243849938 | 2065301432 | 2063317442 |
| [M+H]+ | 4-(1-piperazinyl)-1h-indole | 3.133653272 | 0.557971011 | 0.029973416 | 202.12235 | Organoheterocyclic compounds | 1161872367 | 720651295 | 790012045.7 | 1337298630 | 408589046.1 | 592301265.5 | 591292585.6 | 645188423.8 | 564424658 | 789080823.4 | 565876536 | 794121674.9 |
| [M-H]- | Glabrol | 2.583005538 | 0.665962626 | 0.029980759 | 391.17958 | Phenylpropanoids and polyketides | 749296813.7 | 1051189602 | 893522541.2 | 812191373.2 | 354177525.4 | 672589336.5 | 595433528.4 | 712797988.9 | 703659505.3 | 257098713.7 | 706753297 | 641461926.6 |
| [M-H]- | Val-Val | 1.159247429 | 0.490130608 | 0.030455579 | 215.14010 | Organic acids and derivatives | 140742584 | 83508190.1 | 95348137.48 | 171696911.6 | 39475795.24 | 71144313.3 | 77761785.37 | 52417226.56 | 64228421.73 | 66867471.01 | 68340062.77 | 87138086.26 |
| [M-H]- | Trp-Trp | 1.754153358 | 0.413419638 | 0.030984653 | 389.16388 | Organic acids and derivatives | 208657617.6 | 133640008.1 | 361289319.2 | 244017550.2 | 84802105.5 | 140911333.8 | 80147879.99 | 85896988.34 | 103202661.4 | 90706410.63 | 117781261.6 | 119164032.3 |
| [M-H-CO2]- | 3-hydroxyphenylacetic acid | 2.917142982 | 0.561967963 | 0.031044084 | 107.05012 | Benzenoids | 1040690346 | 500322209 | 872572294.4 | 792147578.4 | 408955930.7 | 600758202.7 | 346339994.7 | 445464792.8 | 687310233.6 | 543405606.3 | 625479826.4 | 772816542.9 |
| [M+H]+ | Ser-Gly-Ser | 1.413307968 | 0.575454279 | 0.031997856 | 250.11106 | Organic acids and derivatives | 217839881.1 | 133653018.1 | 179255248 | 210020079.3 | 66469271.47 | 164726162.2 | 91576025.93 | 103506786 | 208615618.5 | 182086675 | 196544394.5 | 176531282.7 |
| [M-H]- | Clausarin | 3.905031881 | 0.304761364 | 0.032988559 | 379.21256 | Phenylpropanoids and polyketides | 1324047762 | 520142291.2 | 609600730.6 | 1501395906 | 321170578.4 | 350822877.9 | 300721821.2 | 232672814.6 | 170753199 | 350323709.8 | 529244768.1 | 429905206.1 |
| [M+Na]+ | Medroxyprogesterone 17-acetate | 3.518944799 | 0.351163692 | 0.033009368 | 409.25872 | Lipids and lipid-like molecules | 983768937.4 | 273407705.9 | 825712396.1 | 1028293302 | 144297134.5 | 426017983.9 | 268278458.5 | 253940701.7 | 339325293.5 | 120124100.3 | 523803705.1 | 1187723743 |
| [M-H]- | Arachidonic acid (peroxide free) | 2.163894668 | 0.450873005 | 0.03372028 | 303.21784 | Lipids and lipid-like molecules | 449769122.1 | 188982347.3 | 331426684 | 300537725.8 | 56250057.14 | 219150026.5 | 129469702.3 | 168061700.4 | 244506119.8 | 265156824.5 | 285340081.7 | 344574271.9 |
| [M-H]- | Substance p (9-11) | 1.712659732 | 0.561353543 | 0.033927667 | 317.14058 | Organic acids and derivatives | 295009477.4 | 306653979.7 | 407628409.3 | 210739657.1 | 174533398.8 | 237166462.7 | 171378322.4 | 101790834.3 | 590188006.7 | 302850642.1 | 2310975234 | 218651081.5 |
| [M+H]+ | Huperzine b | 3.050167909 | 0.664270332 | 0.033954972 | 257.16087 |  | 1603228871 | 908038088.5 | 1182912770 | 1282095763 | 709056090.5 | 963286846.6 | 820844633.9 | 812404601.6 | 1332181218 | 1101969944 | 1354547287 | 1145843010 |
| [M+H-CH2O]+ | Apiole | 1.026874362 | 0.520289437 | 0.034457256 | 193.09766 |  | 109861571.2 | 54536717.79 | 105645186 | 132317556.7 | 50197263.08 | 71621466.36 | 42550253.01 | 44975212.2 | 127901850.1 | 90260083.5 | 94059578.74 | 85616804.94 |
| [M+H]+ | Trans-zeatin | 1.98091913 | 0.471405964 | 0.03483356 | 220.10013 | Organoheterocyclic compounds | 378500766.8 | 219748533.1 | 247757045.2 | 328455221.8 | 54980359.32 | 255624988.3 | 152668769.4 | 90374070.18 | 200585828 | 183258349.3 | 452943690.7 | 227789755.9 |
| [M-H]- | Lys-Asn | 2.265633663 | 0.578732464 | 0.034845765 | 259.15517 | Organic acids and derivatives | 621327055.4 | 331486797.8 | 611894782.7 | 482611164.1 | 195320797.3 | 294322105.1 | 298580630.1 | 396626899.9 | 306997430.9 | 258343821.6 | 134844418.8 | 400936495.1 |
| [M-H]- | 4,6-dinitro-o-cresol | 8.620119731 | 0.414248758 | 0.035143878 | 197.01255 | Benzenoids | 6153417444 | 3674483406 | 6485448337 | 3461341831 | 1022814505 | 4149978478 | 1433407799 | 1585440407 | 2660499753 | 1054366252 | 3600903033 | 1657145411 |
| [M-H]- | Hydroxyphenyllactic acid | 1.266979353 | 0.410166729 | 0.036171194 | 181.05069 | Phenylpropanoids and polyketides | 133531106.1 | 107208501.6 | 168824395.8 | 55945417.58 | 36482575.03 | 74977624.03 | 44768513.44 | 34707763.88 | 29556116.28 | 46860731.75 | 81399759.86 | 306687601 |
| [M-H]- | Cochlioquinone a | 4.566200778 | 2.508710974 | 0.036863403 | 531.29981 |  | 408005760.9 | 637246231.3 | 874374413.7 | 281497294.6 | 633748848.4 | 1996115753 | 1505402858 | 1386715722 | 501220543.5 | 286018517.3 | 675583793.3 | 178442534.7 |
| [M+H]+ | 2-amino-9h-pyrido[2,3-b]indole | 1.320457978 | 0.659673317 | 0.037446708 | 184.10817 | Organoheterocyclic compounds | 285646627.3 | 209069127.5 | 179880529.9 | 247305541 | 113623282.9 | 201682113.4 | 152492731.6 | 140355907.1 | 188294256.1 | 169271906.7 | 320627429 | 313181296.1 |
| [M+H]+ | 4-aminovaleric acid betaine | 9.886537767 | 0.226108732 | 0.037578471 | 160.13312 | Lipids and lipid-like molecules | 8502232391 | 3012831278 | 6056465581 | 2366419041 | 501552269.9 | 1269263235 | 887565334.7 | 1849763372 | 281709382.2 | 4946648785 | 1069792569 | 1241578336 |
| [M-H]- | Chrysin | 1.275532558 | 0.330177118 | 0.037625917 | 253.03895 | Phenylpropanoids and polyketides | 162408317.1 | 57780837.28 | 67878906.94 | 145987965.5 | 34599398.63 | 44744492 | 44815947.31 | 19155529.92 | 16040528.43 | 25178221.62 | 36078989.82 | 63586623.24 |
| [M-H]- | Homoplantaginin | 1.069564357 | 0.506663026 | 0.03816498 | 461.09725 | Phenylpropanoids and polyketides | 133552888.1 | 51831009.33 | 105173840.7 | 103692899.9 | 48609745.8 | 69706589.1 | 42666916.96 | 38768969.46 | 78115059.1 | 42733212.19 | 98560780.68 | 55928000.67 |
| [M+H]+ | Swainsonine | 1.864735933 | 0.549961412 | 0.038272496 | 174.11229 |  | 441115873.8 | 269422646.6 | 270541661.1 | 280438653.2 | 152648910.7 | 272583041.5 | 140031049 | 128523678.2 | 185571191.4 | 243983415.7 | 437376263.7 | 322446274.4 |
| [M+H-H2O]+ | N-mesitylbicyclo[2.2.1]heptane-2-carboxamide | 1.284652892 | 0.663754133 | 0.038317001 | 240.19560 | Lipids and lipid-like molecules | 266351982 | 165040779.8 | 212163173.5 | 234659483.3 | 138501017.3 | 198304870.7 | 128699457 | 117413769.1 | 189647832.5 | 185216579.1 | 171487436.8 | 172726509.6 |
| [M-H]- | 2-oxooctanoic acid | 1.803125619 | 0.383025305 | 0.038411769 | 157.08706 | Organic acids and derivatives | 330718464.6 | 108163977.8 | 166661229.9 | 260672727.7 | 68490127.78 | 96242025.69 | 60359598.41 | 106691049.3 | 85309803.08 | 109771286.2 | 112697887.2 | 115539080.5 |
| [M-H]- | Ala-Ala | 1.560462763 | 0.569950349 | 0.038520358 | 159.06628 | Organoheterocyclic compounds | 244558229.7 | 178877180.5 | 215687982.9 | 183851722.4 | 50176009.96 | 133088025.6 | 94918633.94 | 190872285.1 | 188986278.2 | 133498638.3 | 389721442.9 | 235219302.2 |
| [M-H]- | N-fructosyl pyroglutamate | 1.149044607 | 0.41252685 | 0.038547754 | 290.08835 | Organic acids and derivatives | 119824813.6 | 102977651.1 | 114267497.6 | 37799588.84 | 33757428.19 | 44638097.94 | 17596253.22 | 58651975.91 | 32505775.57 | 97566869.03 | 73357499.32 | 47412913.6 |
| [M-H-H2O]- | 11-dehydrothromboxane b2 | 1.084320479 | 0.494314718 | 0.038628013 | 349.17720 | Lipids and lipid-like molecules | 121741529 | 133534336.8 | 64514262.97 | 103198578.7 | 47729936.36 | 78631161.13 | 66282280.7 | 16446165.25 | 81004085.08 | 39292973.44 | 119368250 | 156796972.5 |
| [M-H-H2O]- | N-[tris(hydroxymethyl)methyl]-3-amino-2-hydroxypropanesulfonic acid | 2.669098937 | 2.576330158 | 0.038637954 | 240.05693 | Organic acids and derivatives | 84303946.34 | 259631091.2 | 249452711.2 | 143207853.5 | 213232931.3 | 693682269.3 | 438367828.5 | 552430435 | 188715172.7 | 178829888.6 | 139331128.6 | 299864921.4 |
| [M-H]- | Indole-3-pyruvic acid | 1.923522204 | 0.480205675 | 0.039096136 | 202.02949 | Organoheterocyclic compounds | 474175866.5 | 259109974.2 | 274539892.9 | 204264674.3 | 126038177.7 | 173329550.5 | 134636367.3 | 148048596.8 | 243587350.5 | 211437965.8 | 275231376 | 147372853.6 |
| [M+H]+ | 7,8-dihydroneopterin | 1.56901455 | 0.574678227 | 0.039131598 | 256.09298 | Organoheterocyclic compounds | 314368125.1 | 166263398.2 | 223425920.7 | 233018686.5 | 117735122.5 | 200769369.1 | 99764500.64 | 120248257.2 | 289336510.4 | 302371683.4 | 243889222.2 | 267599379.3 |
| [M-H]- | Neoabietic acid | 3.137192339 | 0.488764506 | 0.039627094 | 301.20218 | Lipids and lipid-like molecules | 1094822209 | 561695079 | 583294056.5 | 1118085517 | 323735862.6 | 569806884.1 | 351894129.2 | 395783924.6 | 770871492.3 | 687619439.4 | 664040265.9 | 963598136.6 |
| [M+H-C8H10O2]+ | Lobelanidine | 1.298088742 | 0.667427316 | 0.039751154 | 202.14307 | Organic nitrogen compounds | 273028846.3 | 171325571.7 | 224673152.5 | 255273649.9 | 149498400.7 | 209393815.5 | 123803692.3 | 134207974 | 231485850.2 | 225702384.7 | 212173627.3 | 184759533.1 |
| [M+H-C6H16O8]+ | Ginsenoside f1 | 1.802427527 | 0.54779623 | 0.039752357 | 423.36237 | Lipids and lipid-like molecules | 472771039.6 | 258346354.8 | 305650015.8 | 232998777.6 | 147609283.9 | 195281642 | 181783486.3 | 170898718.9 | 194857130.2 | 342633358.1 | 425003556.4 | 267906473.2 |
| [M+H]+ | 2-pyrrolidinone, 1-methyl- | 1.590931825 | 0.668816567 | 0.040079181 | 100.07602 | Organoheterocyclic compounds | 330160915.1 | 456641467.4 | 263987233.9 | 337355469.8 | 202390265.9 | 216269556.7 | 224910506 | 284844102 | 379246716.5 | 398088035.5 | 233066379.9 | 232556970.7 |
| [M-H]- | Methyl (1r,4as,9s,10s)-10-acetyloxy-5,9-dihydroxy-1,4a-dimethyl-2-oxo-7-propan-2-yl-10,10a-dihydro-9h-phenanthrene-1-carboxylate | 2.249528204 | 0.650059348 | 0.040536735 | 415.17966 | Lipids and lipid-like molecules | 2436481795 | 1997021820 | 1662197691 | 2740513930 | 1920482574 | 1486547584 | 1182173567 | 1154860588 | 1193177923 | 3355641831 | 3283375703 | 2823957312 |
| [M+H-2H2O]+ | Propofol .beta.-d-glucuronide | 3.53208745 | 0.54573154 | 0.04059945 | 319.15131 | Organic oxygen compounds | 1577919427 | 1083749077 | 1315480741 | 788017532.8 | 330447022.5 | 885314392.7 | 790437055 | 594303332.8 | 145366969 | 195338326.4 | 914448472.6 | 175492665.2 |
| [M-H]- | Dynasore | 2.44009143 | 0.388339195 | 0.041369691 | 321.10147 | Benzenoids | 721866347.5 | 304155338.1 | 357013768 | 334182572.8 | 217480794.3 | 199844786.2 | 145949857.2 | 103587628.6 | 318924743.1 | 216091668.7 | 459751363.9 | 217151012.4 |
| [M-H]- | Geranylgeranyl pyrophosphate | 2.054098973 | 0.495217304 | 0.041548595 | 449.18503 | Lipids and lipid-like molecules | 234796088.8 | 413777422.7 | 494617183.7 | 437630164.5 | 338751228.9 | 194528758.1 | 165067165.7 | 84502691.8 | 197580006.2 | 279431172.3 | 145853100.9 | 476839702.9 |
| [M-H]- | Sulfathiazole | 2.445755735 | 0.435382034 | 0.042171844 | 254.01292 | Benzenoids | 489849645.6 | 390512119 | 387030697 | 523133000.6 | 203985646.4 | 451428954 | 51985954.41 | 72162062.46 | 539435396.3 | 395881910.5 | 434205320.1 | 108202816.6 |
| [M-H]- | 15-cyclohexylpentanorprostaglandin f2.alpha. | 3.054982451 | 0.468304749 | 0.042330668 | 365.23650 | Lipids and lipid-like molecules | 682128545.4 | 895261186.5 | 1042334689 | 444050081.8 | 408744509.4 | 537658690.2 | 379825005.7 | 108551944.9 | 266505665.5 | 179510525.3 | 198509262.7 | 323807785.8 |
| [M+H-H2O]+ | Gomisin c | 2.318056372 | 0.113760658 | 0.042954645 | 519.18019 | Phenylpropanoids and polyketides | 230500935.1 | 310664356.3 | 45633664.4 | 488704639.4 | 8139385.783 | 18379853.41 | 21455575.62 | 74375181.89 | 43423104.84 | 44518926.87 | 53815042.18 | 43622614.86 |
| [M-H]- | Vitamin k1 | 3.346114743 | 2.721284722 | 0.04298491 | 449.32734 | Lipids and lipid-like molecules | 319007466.5 | 105059067.2 | 218388957.1 | 153628252.1 | 667814010.6 | 770004582.7 | 535240959.4 | 193310974.4 | 788810160.1 | 292700064.1 | 163349106.8 | 297980215.8 |
| [M-H]- | Mesoporphyrin ix | 2.376197138 | 2.512508212 | 0.043216894 | 565.28398 | Organoheterocyclic compounds | 92101128.32 | 155097671.1 | 104520971 | 108926962.7 | 411908829.1 | 365875909.8 | 269821282.4 | 109772678.4 | 175180027.9 | 112681950.9 | 351359720.9 | 237583151.3 |
| [M-H-C4H8]- | Neobavaisoflavone | 1.151025883 | 0.595384924 | 0.043347711 | 265.05666 | Phenylpropanoids and polyketides | 185052085.5 | 126612054.8 | 109182413.7 | 181178985.9 | 85601482.54 | 129393458.3 | 62259987.05 | 81182002.17 | 121448043.7 | 129017853.2 | 160764889.8 | 193311902.3 |
| [M-H-H2O]- | 17-phenoxytrinorprostaglandin f2.alpha. | 3.383828718 | 0.581623162 | 0.043941892 | 385.20524 | Lipids and lipid-like molecules | 992283994.3 | 1385418736 | 1498250824 | 1023345445 | 765960070.8 | 1027457408 | 775262263.4 | 280866034.9 | 794532462.2 | 1099603554 | 909704141.3 | 704740642.3 |
| [M-H]- | 2,2-dimethylglutaric acid | 1.733894341 | 0.35878114 | 0.04447134 | 159.06632 | Lipids and lipid-like molecules | 244466131.1 | 80247206.9 | 162705416.1 | 236038581.1 | 18906164.47 | 53478132.04 | 50513143.4 | 136665407.4 | 86065787.46 | 88082932.01 | 370116602.2 | 206473030.2 |
| [M+H]+ | Methyl (1-(cyclohexylmethyl)-1h-indole-3-carbonyl)-l-valinate | 1.423166244 | 0.444208366 | 0.044958217 | 371.22803 | Organic acids and derivatives | 109966359.9 | 203544989.7 | 225722659.8 | 86599096.01 | 64936725.26 | 61239105.42 | 76193785.13 | 75630685.3 | 1102373441 | 324158442.9 | 84470480.13 | 104312545.3 |
| [M-H-H2O]- | 3-methoxyprostaglandin f1.alpha. | 4.464658262 | 0.479135462 | 0.046143445 | 367.24883 | Lipids and lipid-like molecules | 2200397034 | 1304571749 | 1380460943 | 2794825499 | 504989888.3 | 1072608314 | 1340317196 | 761967233.7 | 1608690593 | 1267873076 | 1104522324 | 2356265403 |
| [M+H]+ | N-(2-hydroxyethyl)-14(15)-epoxy-5z,8z,11z-eicosatrienamide | 1.02790763 | 0.668279668 | 0.046333138 | 364.30430 | Organic nitrogen compounds | 173605860.1 | 134909300.4 | 113275884 | 144137970.8 | 64261065.66 | 131940147.8 | 91296806.41 | 90700834.71 | 108531430.5 | 129117185.4 | 99620453.92 | 128559203.7 |
| [M+H]+ | 4-ethoxycoumarin | 3.389349902 | 0.583252013 | 0.047675418 | 191.08145 |  | 1730768019 | 1238095375 | 811462058.1 | 1753390789 | 731545062.6 | 974363844.7 | 717575599.2 | 804066628.1 | 1435493432 | 1210804332 | 741934786.5 | 1366621675 |
| [M+H]+ | 2-aminophenol | 1.349000736 | 2.057029293 | 0.04783674 | 110.07133 | Benzenoids | 18602565.66 | 96359201.81 | 79762192.67 | 57705006.26 | 94327030.22 | 138460451.9 | 100818286.3 | 185648009.9 | 74264677.25 | 128312397.4 | 170937815.4 | 89277756.05 |
| [M+H]+ | 3h-imidazo(4,5-f)quinoline, 2-amino-3-methyl- | 1.700370096 | 3.082319265 | 0.047972292 | 199.09797 |  | 11573664.8 | 6285007.071 | 88821865.02 | 84758993.51 | 121080630.8 | 109994289 | 111747655.9 | 247255177 | 12219317.05 | 97975873.58 | 43976975.42 | 125735932.3 |
| [M-H]- | Xanthine | 4.177748538 | 0.480904704 | 0.049106031 | 151.02618 | Organoheterocyclic compounds | 1454450923 | 1375800687 | 1026498655 | 2483539283 | 518418831.6 | 713932297.2 | 738572891.6 | 1078151050 | 1638052103 | 2039051906 | 1950204194 | 1992220001 |
| [M-H]- | Hypoxanthine | 4.584275586 | 0.587879446 | 0.04994631 | 135.03125 | Organoheterocyclic compounds | 3244392870 | 2107205571 | 2132799194 | 3463536872 | 1782981217 | 1953285416 | 1949176385 | 750622649.8 | 3646286131 | 3187482159 | 2339738504 | 5257945577 |
|  |  | 1.423712404 | 8.095226856 | 4.68809E-06 | 567.29830 |  | 4302197.246 | 16304311.08 | 4589304.333 | 8939440.622 | 71156937.7 | 60997904.9 | 70849273.49 | 73328503.01 | 16231044.63 | 35118534.59 | 3008680.733 | 5479560.504 |
|  |  | 1.281934577 | 0.159396416 | 5.16976E-06 | 337.03874 |  | 67199212.34 | 66861221.69 | 60375208.25 | 63013130.85 | 4240576.084 | 11149176.6 | 18755937.94 | 6890721.11 | 45701746.63 | 59877156.15 | 89155351.01 | 75515813.66 |
|  |  | 1.913663379 | 0.153070238 | 2.16728E-05 | 337.03906 |  | 149125098.4 | 145225339.1 | 155096837.8 | 128876090 | 18890646.09 | 21536832.41 | 45041363.98 | 3055252.794 | 114416203.2 | 132040748.4 | 344054293.1 | 293107552.5 |
|  |  | 1.226082475 | 0.366397803 | 3.02741E-05 | 195.06634 |  | 82760700.94 | 85880489.78 | 83578413.91 | 74256209.3 | 35371387.63 | 37039184.88 | 26624066.98 | 20585381.38 | 143083820.6 | 49232713.37 | 66208066.67 | 65555389.27 |
|  |  | 1.337157612 | 9.010277395 | 6.68596E-05 | 500.34890 |  | 3304381.57 | 10009506.89 | 2588659.788 | 11102049.37 | 74641462.55 | 62247228.41 | 52323487.59 | 54106736.91 | 10498825.47 | 37849858.04 | 11124077.73 | 18020854.6 |
|  |  | 2.881890168 | 4.278367872 | 6.70136E-05 | 285.20729 |  | 43305046.13 | 114872515.3 | 70031605.12 | 73739815.14 | 367483550.8 | 295087101.3 | 347940925 | 281337244.9 | 105341726.9 | 326291080.3 | 220828336.3 | 202190404.6 |
|  |  | 1.447199971 | 0.372326244 | 8.54676E-05 | 409.17263 |  | 121925726.3 | 97956518.09 | 110707741.7 | 121994282.4 | 28003410.43 | 51503337.83 | 45797622.85 | 43204629.59 | 52062027.08 | 42096488.11 | 54587522.63 | 53902152.06 |
|  |  | 1.309304585 | 3.492518 | 9.09776E-05 | 407.28017 |  | 18663459.11 | 27053012.56 | 15074712.6 | 22100029.94 | 66037652.03 | 72703975.26 | 86308270.59 | 64449159.74 | 52035084.7 | 41285718.85 | 31715985.59 | 32920624.76 |
|  |  | 1.869866419 | 0.275922911 | 0.000155628 | 275.09611 |  | 191430480.4 | 167252043.8 | 165578902.4 | 148682787.6 | 37124783.22 | 47782527.34 | 77579597.52 | 23193818.17 | 50600120.38 | 60275645.11 | 129473359.3 | 102825764.4 |
|  |  | 4.227999383 | 0.533826887 | 0.00016054 | 379.29561 |  | 1550288665 | 1266059566 | 1378896680 | 1292956590 | 609978261 | 797908094.5 | 771759732.1 | 750103433 | 943834247.4 | 1092296645 | 982159543.4 | 938468934.7 |
|  |  | 3.509490958 | 0.235925454 | 0.00020176 | 377.09117 |  | 628084915 | 600135473.4 | 547510679.4 | 401703512.8 | 122185820.7 | 154567510.9 | 140126697.9 | 96832213.45 | 208416827.6 | 256630449.5 | 629875924.4 | 220400931.9 |
|  |  | 1.761113466 | 4.27794506 | 0.000246282 | 801.52849 |  | 12977360.17 | 47386277.68 | 41073533.26 | 15103071.29 | 113265408.4 | 107391899.7 | 144535923.1 | 133359523.2 | 70705509.95 | 99835140.08 | 42513609.42 | 66133256.61 |
|  |  | 1.862190585 | 5.013906448 | 0.000253112 | 171.07654 |  | 33626959.23 | 31458989.26 | 19700393.58 | 22364926.36 | 157744205.5 | 132342452.2 | 96895554.52 | 150264223.4 | 24755726.95 | 26784763.76 | 24003316.26 | 22366937.97 |
|  |  | 1.251251964 | 3.435248159 | 0.000268417 | 327.26853 |  | 10993766.96 | 31452678.2 | 24148484.24 | 12734623.29 | 58740440.53 | 72954153.57 | 76714642.29 | 64107463.51 | 31763670.77 | 82537163.32 | 23747473.89 | 21542740.32 |
|  |  | 1.074112951 | 3.020055164 | 0.000304647 | 432.31194 |  | 19829428.34 | 22083591.82 | 13477101.59 | 13241985.32 | 62733543.06 | 51776500.05 | 49712534.32 | 43050171.96 | 11381660.46 | 25427317.67 | 1471189.37 | 11954051.41 |
|  |  | 1.801230259 | 0.369324756 | 0.00036647 | 271.15525 |  | 208629582.6 | 146592749 | 192323851.2 | 159125638.6 | 53045573.4 | 80938976.11 | 62331621.26 | 64675227.53 | 73743415.01 | 116388855.4 | 164261144.3 | 106257281.2 |
|  |  | 1.267895384 | 12.09979997 | 0.000377643 | 86.61667 |  | 905935.9319 | 12605565.57 | 3980925.126 | 662218.0806 | 45922397.84 | 58846033.14 | 71469428 | 43429710.52 | 25010906.83 | 30914884.17 | 10047149.6 | 16232845.94 |
|  |  | 2.892386409 | 0.61451064 | 0.000395828 | 361.28501 |  | 838847858.3 | 854913403.8 | 759818137.7 | 750652218.4 | 392399606.4 | 534589524 | 501137981.3 | 540907310.3 | 558849456.3 | 547073245.3 | 535710197.6 | 613691324.8 |
|  |  | 1.472214171 | 0.134346645 | 0.000435501 | 489.23189 |  | 92176004.07 | 71950509.68 | 69385357.14 | 114225766.5 | 8026071.997 | 14894136.55 | 17930521.17 | 5866655.292 | 4750649.544 | 2358293.197 | 1783947.814 | 20777979.03 |
|  |  | 1.314211902 | 0.273293854 | 0.000435751 | 180.00518 |  | 87774834.69 | 61957913.06 | 86137596.25 | 95257324.01 | 19149158.8 | 34658121.84 | 24535549.05 | 12152326.75 | 59898701.45 | 43553842.71 | 66872765.05 | 42072796.79 |
|  |  | 1.66164621 | 10.13074778 | 0.000445572 | 272.00832 |  | 18362462.76 | 7530343.45 | 3764914.183 | 7166527.903 | 119671866.2 | 103806083.2 | 83496898.89 | 66082323.43 | 11836506.43 | 4292132.54 | 11875819.12 | 7532504.606 |
|  |  | 5.101978467 | 0.631128437 | 0.000520676 | 379.29554 |  | 2774612150 | 2702207712 | 2459639887 | 2479838307 | 1309656898 | 1804928472 | 1638806075 | 1820630463 | 1764427159 | 1807664861 | 1748746119 | 1894513530 |
|  |  | 7.659543489 | 0.424012956 | 0.000522446 | 434.18900 |  | 3958053565 | 3761669669 | 3177471353 | 3373201758 | 968313744.7 | 2117385498 | 1674576005 | 1290557689 | 3691245868 | 2528777246 | 1298912155 | 6530294463 |
|  |  | 3.626806326 | 0.236027594 | 0.000547889 | 307.12195 |  | 676723884.4 | 398949101.3 | 630238164.1 | 682893173.8 | 132598928.3 | 177839347.9 | 117196212.8 | 136189248 | 143281689.2 | 242967723 | 415741894.4 | 220930310.1 |
|  |  | 1.433716915 | 0.342613455 | 0.000564702 | 243.16028 |  | 116385532.4 | 80083386.01 | 124415905.7 | 129099901.3 | 36696174.52 | 38194771.09 | 39057568.06 | 40222307.96 | 7707731.065 | 22629935.21 | 53477181.53 | 26672409.83 |
|  |  | 2.34287229 | 1.883377941 | 0.000569602 | 879.38222 |  | 188959954.7 | 200092554.6 | 215549430.3 | 142624579.5 | 359990755.8 | 296979337.5 | 387806637.5 | 362533212 | 222828138.2 | 245828503.3 | 232442707.5 | 181844947.4 |
|  |  | 1.134577948 | 0.579219293 | 0.000651053 | 365.31658 |  | 115862177.2 | 123256840.4 | 117733226.1 | 112882961.1 | 51184909.46 | 82020210.03 | 78408521.76 | 60466051.84 | 86558522.1 | 214543743.6 | 67108366.58 | 103330715.9 |
|  |  | 2.528904042 | 0.23434996 | 0.00065793 | 403.14317 |  | 262314581.1 | 215742088.8 | 327259352.6 | 368793541.7 | 60459170.65 | 85924642.43 | 51340413.46 | 77428303.25 | 191901285.2 | 347854985.4 | 304897142.8 | 333375058.5 |
|  |  | 1.009568743 | 0.599681242 | 0.000762646 | 331.08576 |  | 105574642.2 | 99151704.24 | 100188415.4 | 96041636.21 | 65441846.21 | 74218700.44 | 54094808.54 | 46690675.53 | 73313824.64 | 87612336.61 | 94977131.98 | 63043159.08 |
|  |  | 3.823809875 | 0.295915155 | 0.000763467 | 377.09111 |  | 934369297.6 | 544671364.2 | 696697442.1 | 708909987.1 | 197797325.9 | 243940521 | 203928709.1 | 207944529.6 | 209815337.6 | 321900534 | 607579260.7 | 261843852.6 |
|  |  | 13.60007171 | 2.494485893 | 0.000772232 | 389.26967 |  | 2629132507 | 6191027113 | 3580033747 | 2608358430 | 10171441019 | 8823038129 | 9447694540 | 8996447049 | 8705828675 | 8621470113 | 4996936621 | 5712690071 |
|  |  | 1.744855246 | 0.423466541 | 0.00078107 | 363.11193 |  | 213525591.9 | 146766178.7 | 188780917.9 | 189900496.3 | 64081517.53 | 107446304.6 | 72931571.51 | 68471025.04 | 95563815.74 | 91351122.78 | 121680742.2 | 83698529.89 |
|  |  | 1.780498828 | 0.393816282 | 0.000795093 | 237.11334 |  | 160919861.9 | 205462399.3 | 194141274.5 | 169745050.6 | 42092268.9 | 111440592.6 | 68333957.62 | 65724840.18 | 134271708.9 | 117035949.1 | 110455196.3 | 128664849.1 |
|  |  | 3.673194253 | 0.211775266 | 0.000820449 | 188.00232 |  | 697569597.7 | 535346890.5 | 748338013.7 | 430112026.8 | 166712127.2 | 141025633.8 | 141269997.2 | 61660030.21 | 229198642.2 | 183489731.2 | 80218856.65 | 267083475.4 |
|  |  | 1.020924462 | 0.394694673 | 0.000841131 | 240.12322 |  | 61650942.41 | 68150254.85 | 52095297.16 | 65521612.54 | 23750676.22 | 36462018.56 | 25232667.07 | 12209246.86 | 30198916.6 | 35489713.06 | 51583955.31 | 62408624.6 |
|  |  | 2.502637446 | 0.437058565 | 0.000880916 | 363.11163 |  | 417259988.4 | 405966509.4 | 427100749.9 | 383091932.4 | 200866906 | 243135689.7 | 196069660.8 | 73827585.63 | 171518685.2 | 272870360.6 | 295587917 | 273564094.9 |
|  |  | 1.059898171 | 0.453431089 | 0.000953803 | 217.15868 |  | 90906818.12 | 83165358.92 | 74948971.59 | 67767672.97 | 50261762.8 | 36083503.11 | 30759319.54 | 26537314.87 | 49760008.99 | 55910472.98 | 56288633.52 | 35092171.7 |
|  |  | 8.735780716 | 1.845981606 | 0.000972974 | 217.00315 |  | 3041605343 | 3106325128 | 2428229445 | 2137562741 | 4744464864 | 4326658061 | 4971436212 | 5734775818 | 3932446563 | 5436592714 | 3708687110 | 2905600028 |
|  |  | 1.590055205 | 3.801998202 | 0.001031115 | 391.28452 |  | 22833648.06 | 40816493.92 | 29630403.08 | 20850547.15 | 79261319.9 | 95147656.88 | 128183598.9 | 131333631.8 | 63386137.47 | 68780162.28 | 34625181.99 | 45937444.76 |
|  |  | 1.934705232 | 0.27283509 | 0.001044926 | 341.10621 |  | 218751366.1 | 150569337.1 | 201964133.1 | 150085405.5 | 55038068.66 | 78009604.46 | 50618298.11 | 13149143.55 | 89876895.9 | 103921327.7 | 211537720.8 | 99044369.38 |
|  |  | 1.188494865 | 0.372374058 | 0.001208972 | 113.02451 |  | 83666731.4 | 90716161.49 | 56112777.56 | 88081748.51 | 25889543.45 | 33395788.96 | 22045039.65 | 37299594.18 | 58760707.62 | 53834001.81 | 76736106.64 | 83623974.2 |
|  |  | 4.293222063 | 8.26221629 | 0.001257769 | 780.55003 |  | 48282464.79 | 165673490.2 | 80306341.68 | 32624198.2 | 546054613.1 | 661355979.9 | 961546494.9 | 531849834.9 | 321192802.5 | 359520105.7 | 149431726.5 | 220298268.4 |
|  |  | 1.655434042 | 0.540691064 | 0.001284792 | 377.09104 |  | 253910362.9 | 211505446.5 | 268518458.4 | 201900594.4 | 132958220.5 | 146763506.2 | 126614454.1 | 99661366.95 | 100972375.1 | 167019779.3 | 319097115.8 | 132870998.2 |
|  |  | 2.687115691 | 0.374131491 | 0.001291396 | 162.11256 |  | 444880938.2 | 454961620.9 | 374741833.3 | 310830103.3 | 120750530.9 | 118676619.8 | 121616151.8 | 232110186.5 | 73930124.85 | 137589935.3 | 129866231.6 | 127526050.2 |
|  |  | 2.639441095 | 0.138131924 | 0.001407303 | 361.09610 |  | 358112748 | 310229292.9 | 292539671.2 | 161440636.4 | 36700895.65 | 45512475.47 | 61446191.34 | 11368982.53 | 79098415.58 | 160884800.3 | 119878513.8 | 89124642.49 |
|  |  | 1.029093979 | 0.35864397 | 0.001430471 | 282.04420 |  | 61885436.91 | 55776554.63 | 70547957.94 | 55048997.74 | 23366026.4 | 30087494.01 | 29472770.03 | 4317064.174 | 47434435.45 | 22014562.19 | 65668496.2 | 57607914.09 |
|  |  | 1.183193149 | 0.578957491 | 0.001473433 | 361.09611 |  | 126573978 | 121688113.4 | 138888179.1 | 112985959.8 | 73610979.25 | 72655385.26 | 52484839.82 | 90806412.97 | 61855096.03 | 76489805.68 | 104859570.4 | 75885069.35 |
|  |  | 2.737546111 | 0.087772337 | 0.001638636 | 361.09608 |  | 416252719.9 | 199588744.5 | 293786541.2 | 230989058.5 | 33276284.48 | 23443294.16 | 27614404.57 | 15780641.98 | 41898182.6 | 104921687.4 | 111939672.9 | 56382307.12 |
|  |  | 2.588356038 | 26.65984237 | 0.001785651 | 239.01551 |  | 13709562.49 | 4778122.091 | 9131827.994 | 5125355.987 | 331725662.2 | 183707170.2 | 205503941 | 152036260.9 | 179094138 | 312350282.2 | 135171948.1 | 4310745.738 |
|  |  | 2.106123972 | 0.417014821 | 0.002022452 | 391.17957 |  | 271923995.4 | 304481072.1 | 310194277.2 | 207966328.7 | 92919811.07 | 173372414.1 | 98416542.92 | 91741340.09 | 235066010.8 | 284510489.2 | 557866347.6 | 157499396.2 |
|  |  | 1.895637287 | 2.40524782 | 0.002274193 | 449.32744 |  | 56844857.88 | 76982111.22 | 69661207.35 | 109065531.5 | 172131035.1 | 196326398.3 | 235153191 | 148158500.4 | 123357000.3 | 394447314.8 | 72898306.75 | 101699084.4 |
|  |  | 1.301297107 | 2.572939464 | 0.002302459 | 498.32587 |  | 24877800.38 | 55006072.36 | 18060578.65 | 34314877.27 | 96332150.12 | 95513900.81 | 77359909.52 | 71089285.76 | 63278114.47 | 153605222.7 | 38570781.2 | 59949611.01 |
|  |  | 4.071209316 | 2.959039757 | 0.002326565 | 751.58804 |  | 302847784.7 | 246213515 | 290792909.2 | 215477218.3 | 633078437 | 1071935854 | 665724375.9 | 752028982.9 | 232803359.1 | 380052529.6 | 157480704.6 | 130348454.8 |
|  |  | 1.10819633 | 0.464883574 | 0.002411308 | 245.05174 |  | 6274108.349 | 4763492.794 | 5725077.96 | 3958181.265 | 2533198.577 | 2364316.617 | 1876661.725 | 2858610.697 | 5552478.153 | 5354573.921 | 4577661.334 | 1842280.493 |
|  |  | 2.008725643 | 0.613599171 | 0.002502164 | 363.30178 |  | 12979207.43 | 12856429.38 | 13557576.62 | 11635791.49 | 5897576.064 | 9300964.713 | 9434896.634 | 6677917.691 | 10380402.49 | 11555028.29 | 9931167.165 | 11998151.38 |
|  |  | 2.602873883 | 0.554544455 | 0.002522207 | 303.09086 |  | 538116289.9 | 531544155.4 | 734473921.6 | 621938262.8 | 346637101.8 | 305657659.8 | 286243380.4 | 406826981.4 | 254041909.7 | 332601119.2 | 357010068.9 | 232295675.3 |
|  |  | 1.540521873 | 0.02530716 | 0.002524603 | 479.18759 |  | 118832277.9 | 81347083.81 | 41416415.37 | 104384260.9 | 337541.4749 | 609665.8708 | 1114129.285 | 6694435.54 | 8622196.748 | 6315388.676 | 15764082.95 | 8454149.522 |
|  |  | 1.575642856 | 8.170318647 | 0.002531646 | 465.32221 |  | 9411534.544 | 8720478.695 | 11433475.65 | 14946156.99 | 116342238.1 | 120017461.6 | 56858895.59 | 70455735.11 | 43448320.34 | 59376979.49 | 15311759.24 | 23653319.42 |
|  |  | 2.566996189 | 0.480106606 | 0.002619009 | 193.01768 |  | 470405105.7 | 406137214.1 | 580073687.7 | 492864935.2 | 219397792.3 | 328881902.3 | 236800556.4 | 150878427.5 | 300428491.2 | 400071679.9 | 370126488.3 | 324692137.4 |
|  |  | 1.569995163 | 0.271990427 | 0.002658969 | 462.26416 |  | 92464361.21 | 157359620.5 | 139889893.9 | 95116550.78 | 22228285.26 | 36628769.98 | 53006993.22 | 20005186.48 | 41296459.2 | 30684211.5 | 60082648.05 | 85362283.9 |
|  |  | 1.050566214 | 0.264134534 | 0.002829213 | 357.27886 |  | 60369924.35 | 34931921.33 | 65084088.25 | 57022556.43 | 5221769.538 | 6980806.867 | 21257059.64 | 23965454.28 | 36811899.65 | 29679409.7 | 53456030.14 | 37240418.2 |
|  |  | 1.114101808 | 0.369306227 | 0.002867245 | 275.07771 |  | 78622054.55 | 69449950.9 | 67262913.1 | 60987969.95 | 20197765.86 | 49762163.74 | 13308812.27 | 18779021.45 | 48173752.55 | 49063243.69 | 50323830.07 | 53213500.08 |
|  |  | 1.022868488 | 0.474200982 | 0.002995755 | 187.10840 |  | 84811998.23 | 88628119.72 | 65542334.07 | 63569146.41 | 39560363.89 | 47383688.67 | 33778280.34 | 22747932.18 | 61504033.65 | 81086969.12 | 83713769.22 | 55734337.81 |
|  |  | 2.930487538 | 3.257648324 | 0.003003069 | 127.07138 |  | 84386495.75 | 174245856 | 139423708.8 | 64243461.26 | 430433241.1 | 406626319.6 | 435726641.8 | 233223060.3 | 112977920 | 240879539.7 | 130026225.5 | 361971954.8 |
|  |  | 2.14532041 | 0.486292159 | 0.003027048 | 163.06112 |  | 381111628.1 | 299187070.3 | 308051521.9 | 344294908.4 | 81983756.45 | 147400241.8 | 207698014.5 | 210972864.7 | 77244023.45 | 72294687.58 | 554606667.5 | 543807912.9 |
|  |  | 1.397756894 | 0.390639689 | 0.003313729 | 511.28954 |  | 121258111.6 | 136741818.7 | 98073546.53 | 109552087.1 | 35759648.83 | 82157155.71 | 39238924.07 | 24736096.7 | 38112937.1 | 24279910.32 | 29254462.52 | 101187253.9 |
|  |  | 2.057867547 | 0.416993014 | 0.003326705 | 277.07518 |  | 335858823.5 | 208958918.3 | 279844176.5 | 258599996.8 | 117063698.8 | 158176182.7 | 119631926 | 56840843.35 | 156311704.6 | 147963552.6 | 198817499.3 | 168949901.6 |
|  |  | 1.007654642 | 0.343538033 | 0.003354489 | 422.22983 |  | 56709866.92 | 43804302.78 | 63287584.33 | 51553766.39 | 2400841.992 | 16714113.79 | 22165953.07 | 32701903.13 | 7554223.167 | 12082592.79 | 66135821.96 | 40674703.49 |
|  |  | 1.099742966 | 0.455118823 | 0.003361295 | 268.01333 |  | 90714868.41 | 66028089.11 | 82538214.35 | 74567201.53 | 20928784 | 56305861.15 | 33541097.05 | 32062560.09 | 48307467.17 | 45296644.05 | 26810658.47 | 50423746.58 |
|  |  | 1.323243022 | 0.556602288 | 0.003406732 | 235.02835 |  | 173040976.4 | 118848085.7 | 156821433.2 | 173875450.2 | 86849317.15 | 106761739.6 | 74183502.58 | 78738202.47 | 113957703.7 | 131573559.7 | 147267071.3 | 117650315.3 |
|  |  | 2.251797411 | 0.467932133 | 0.003501849 | 222.99191 |  | 385883201.6 | 275941484.2 | 374798588 | 368651146.8 | 136994110.4 | 254001668.5 | 155011446.3 | 111565831.8 | 212419625.5 | 205400242.1 | 232779510.3 | 212269130.3 |
|  |  | 1.046977392 | 0.474576347 | 0.00351518 | 264.01852 |  | 94656410.36 | 60920964.54 | 84311830.77 | 65341179.08 | 26542955.31 | 40443327.39 | 36770815.7 | 41098022.45 | 45405816.57 | 42986475.05 | 65071493.34 | 27422148.45 |
|  |  | 1.533206739 | 0.507857137 | 0.003663058 | 347.11701 |  | 174845544.6 | 166707511.8 | 236573863.2 | 198545632.5 | 106914573.9 | 127510298.6 | 97620186.41 | 62393639.81 | 127465308.3 | 169021967.7 | 162415455.3 | 141701530.9 |
|  |  | 1.807981323 | 4.348831384 | 0.003688191 | 462.28625 |  | 436854.9072 | 89183349.54 | 4530961.297 | 32648988.02 | 151566576.9 | 143467209.6 | 106272708.9 | 150125992.7 | 34242665.49 | 72437448.81 | 12052286.02 | 30853605.3 |
|  |  | 1.639691605 | 0.42090198 | 0.003772032 | 336.20037 |  | 215168892 | 172404574.7 | 150397635.7 | 186257642.5 | 72508880.28 | 98384009.59 | 108249993.7 | 25686429.23 | 203854483.4 | 75150008.79 | 145431714.5 | 182260521.5 |
|  |  | 1.07225151 | 0.437317011 | 0.003806735 | 279.09078 |  | 98354031.65 | 53818231.53 | 81601567.81 | 79955563.49 | 37830689.68 | 40628829.34 | 30468271.89 | 28271410.22 | 27412322.8 | 56438798.08 | 61145996.33 | 77349063.02 |
|  |  | 2.155503123 | 0.442171815 | 0.003833115 | 393.19532 |  | 338976416.4 | 242435402.9 | 291396615.7 | 301531262.8 | 67967879.65 | 211548130.2 | 120482096.4 | 119261809.1 | 136673649.2 | 170725572.9 | 157788085.1 | 164093598.9 |
|  |  | 1.76841273 | 5.26700042 | 0.003833999 | 203.10264 |  | 15897082.18 | 26827670.18 | 37562348.52 | 9979566.428 | 142294527.6 | 155718366.1 | 112893315.7 | 64528365.24 | 18861320.76 | 25411947.67 | 23145117.14 | 14819425.25 |
|  |  | 2.834505949 | 0.35010791 | 0.003839649 | 409.16920 |  | 390459870.1 | 542879592.7 | 564252629.2 | 369320890.3 | 271104767.9 | 169564323.2 | 152829996.4 | 60121915.35 | 202851772.8 | 581280312 | 298168010 | 190270398 |
|  |  | 1.231023838 | 0.515530856 | 0.003857267 | 255.01847 |  | 138888702.9 | 97695300.38 | 122317591 | 126030888.3 | 55503508.84 | 84028791.34 | 70791373.13 | 39673984.36 | 88854119.04 | 79760316.93 | 128098480.4 | 102171469.2 |
|  |  | 2.518374719 | 0.31874297 | 0.003883402 | 269.05953 |  | 311291611.8 | 466824459.4 | 306193471.7 | 296645322.4 | 49883139.76 | 80006851.48 | 197713631.4 | 112566032.6 | 299016748.3 | 40578790.72 | 280825713.8 | 259435606.2 |
|  |  | 1.861299693 | 0.407209446 | 0.003982346 | 616.30471 |  | 280724505.9 | 156406220.7 | 196590566.6 | 206176560.6 | 62514381.64 | 107074214 | 84952980.55 | 87472763.73 | 72614375.58 | 85453574.29 | 84240290.45 | 78273762.78 |
|  |  | 2.401682707 | 2.103431245 | 0.004001688 | 553.32013 |  | 127802159.5 | 249389328.9 | 151799989.6 | 128272349.4 | 414448395.7 | 350695767.1 | 279030061.6 | 338335046 | 1282117362 | 192326140.9 | 527254783.5 | 533985284.8 |
|  |  | 1.663327146 | 2.675905538 | 0.004074292 | 431.31662 |  | 29090613.21 | 57686332.26 | 78195851.47 | 45884331.91 | 186099832.4 | 144365360.3 | 109015551.2 | 124753015 | 65034841.01 | 128548248 | 75155038.47 | 29708249.03 |
|  |  | 1.623002603 | 0.487416132 | 0.004103087 | 268.01339 |  | 218275639.3 | 141580332 | 182374357.8 | 215741848.5 | 75985213.55 | 127784991.4 | 79507802.56 | 86169859.77 | 89243497.33 | 81049045.73 | 100256889.5 | 98708082.95 |
|  |  | 1.388593737 | 0.396566494 | 0.004155156 | 215.04959 |  | 166028164.8 | 113993032.6 | 99465578.63 | 101258248.3 | 52988023.58 | 56363170.21 | 39387165.46 | 41909009.75 | 104437016.2 | 127211739.4 | 119415277.1 | 100666494.5 |
|  |  | 1.145806747 | 0.638807947 | 0.004177232 | 361.28515 |  | 164447731.5 | 136529184.2 | 138671705.1 | 120599430.8 | 72741164.32 | 100815928.5 | 84522850.14 | 99810964.95 | 99942544.94 | 111569432.3 | 102348077.9 | 94595240.47 |
|  |  | 1.243484799 | 0.407754032 | 0.004189328 | 449.18449 |  | 117991432.1 | 93507615.59 | 113187651 | 91247651.94 | 60033232.34 | 47545190.8 | 54644177.51 | 7376308.011 | 82442162.29 | 77014828.63 | 89115300.92 | 86443148.71 |
|  |  | 3.266761865 | 0.338218549 | 0.004345044 | 167.00195 |  | 794234634.1 | 549054633.4 | 394841707.3 | 655843640.1 | 164561642.4 | 282089445.6 | 204239970.9 | 158795562 | 342943303.5 | 399212331 | 533909645.6 | 400166315.7 |
|  |  | 1.105933081 | 0.345911349 | 0.004563808 | 308.08108 |  | 87269327.19 | 44492899 | 71601183.36 | 65772420.71 | 18870494.62 | 36722755.87 | 21841759.46 | 15662128.24 | 37385271.31 | 29930064.67 | 45317921.53 | 29421033.8 |
|  |  | 1.214603325 | 0.378659371 | 0.004914502 | 214.07115 |  | 120880257.2 | 73932155.86 | 80734355.31 | 102493146 | 31345089.38 | 39159579.79 | 56326636.19 | 16317050.73 | 29465081.2 | 79949126.1 | 31830479.81 | 80572794.4 |
|  |  | 1.735140141 | 2.394040873 | 0.005099052 | 161.09225 |  | 47546244.45 | 100601351.4 | 33488934 | 86191882.87 | 139751227.7 | 151967450.3 | 145155957 | 204317532.1 | 161214530.5 | 162039302.2 | 171808528 | 171508127.4 |
|  |  | 1.003202898 | 0.475771553 | 0.005185471 | 303.09066 |  | 86994500.24 | 83114833.39 | 53283791.63 | 80976151.91 | 24835517.38 | 45986586.63 | 44142275.39 | 29845864.37 | 42085764.45 | 50334699.17 | 52611448.32 | 34154479.24 |
|  |  | 1.038748761 | 3.334240999 | 0.005224884 | 333.06768 |  | 24363287.14 | 7486895.004 | 10414535.97 | 13393339.92 | 51219520.32 | 60398048.85 | 45119175.66 | 28840634.18 | 55708281.29 | 12792086.83 | 6972436.164 | 10626163.93 |
|  |  | 2.062265642 | 0.328735242 | 0.005240065 | 301.03881 |  | 184736015.8 | 266592709.8 | 298139346.2 | 189579118.9 | 66263801.26 | 137254725.5 | 82609607.68 | 22569770.84 | 166179598.4 | 195056320.9 | 222064170.4 | 209307683.6 |
|  |  | 1.861181302 | 0.350095612 | 0.005286695 | 409.25856 |  | 206982728.8 | 179110586.6 | 163299431.1 | 224219688.9 | 34405556.05 | 144003933 | 58849077.9 | 33579751.88 | 86398389.19 | 106066946.1 | 499052531.7 | 451142554.9 |
|  |  | 4.499554956 | 0.367804366 | 0.005322248 | 214.01827 |  | 1353352281 | 1652498967 | 873972232.3 | 998829148.5 | 540331257.2 | 475470405.6 | 431180544.1 | 347407529.9 | 310281658.3 | 948544232.3 | 984361883.1 | 746558029.8 |
|  |  | 2.540067435 | 0.534369372 | 0.005324819 | 291.12723 |  | 606039544.3 | 446581353.3 | 501427631.4 | 579530416 | 236971603 | 406743109.7 | 298203089.2 | 198201438.1 | 197403305.5 | 465979680.8 | 197178620.1 | 405175205.8 |
|  |  | 1.648757793 | 0.565240166 | 0.005384249 | 172.10848 |  | 273001450.6 | 192817277.2 | 241056743.7 | 219583497.1 | 111638920.9 | 177864143.1 | 104271578.1 | 129897178.8 | 275907385.5 | 248760486.6 | 242847711.7 | 261505679.8 |
|  |  | 1.515537855 | 0.322101016 | 0.005439647 | 319.06799 |  | 138339477.7 | 84849790.16 | 106748704.6 | 162128853.9 | 25844909.37 | 31341380.12 | 32567639.28 | 68741296.16 | 60628352.72 | 78116843.44 | 82129130.14 | 75643703.45 |
|  |  | 1.033584063 | 0.430300833 | 0.005450137 | 453.21611 |  | 83310891.56 | 56384586.87 | 77844811.1 | 63325364.84 | 32168257.54 | 48124667.39 | 26551553.16 | 14012246.9 | 64076787.3 | 61838777.41 | 70047035.66 | 47215460.42 |
|  |  | 1.578833615 | 0.272617666 | 0.005517823 | 215.03291 |  | 119579358.5 | 182363113.8 | 84338646.89 | 109215788.2 | 29587552.28 | 39050192.25 | 23499035.73 | 42944430.29 | 93281057.4 | 60282964.28 | 101013102.5 | 97412152.7 |
|  |  | 1.597454254 | 0.487024833 | 0.005570098 | 160.07526 |  | 197786120.3 | 214357773.4 | 156232653.5 | 153265961.7 | 58635692.46 | 115224537 | 62389912.52 | 115207680.5 | 86958708.12 | 88011025.43 | 311626295 | 88321580.57 |
|  |  | 2.381542874 | 2.963301617 | 0.005628206 | 373.27393 |  | 94101831.94 | 105907811.5 | 73540425.42 | 97896325.2 | 215364911.4 | 402109371.9 | 241080771.7 | 242152645.2 | 210249368.7 | 168884943 | 125338822.7 | 151583732.5 |
|  |  | 8.63037043 | 0.38710311 | 0.00565491 | 359.18967 |  | 5485913235 | 3518935614 | 5815378136 | 3725725436 | 919375576 | 2083159015 | 2455031304 | 1721629963 | 3099722033 | 3699091150 | 1168695230 | 2713644909 |
|  |  | 1.696500859 | 0.403134828 | 0.005655752 | 265.07530 |  | 222425236.2 | 145148122.3 | 140789377.3 | 230314113 | 70275281.74 | 104351296.2 | 63414645.2 | 59745140.96 | 50730492.1 | 56922572.48 | 61561443.47 | 76483095.72 |
|  |  | 1.047584175 | 0.200750543 | 0.005676303 | 371.08065 |  | 60765098.6 | 27200112.94 | 50895448.24 | 74090669.93 | 16095643.75 | 10423712.85 | 5539701.772 | 10691036.76 | 13690235.61 | 11457222.98 | 39815214.2 | 24600207.58 |
|  |  | 1.159421984 | 2.506526208 | 0.00570808 | 449.29083 |  | 17699280.13 | 36130294.52 | 24567018.56 | 29831938.8 | 71405948.55 | 73420920.98 | 83985600.62 | 42465181.83 | 68713736.94 | 58462896 | 34093068.94 | 28103131.97 |
|  |  | 1.050887726 | 0.409652071 | 0.005727966 | 448.20497 |  | 83174409.05 | 68136020.98 | 72158002.83 | 49883022.33 | 17282672.46 | 46316712.18 | 29479925.11 | 18899679.89 | 52870799.32 | 46706384.4 | 30130124.05 | 83370307.62 |
|  |  | 1.644760121 | 3.739470438 | 0.005973115 | 356.26703 |  | 39478677.14 | 13646280.43 | 10180139.51 | 62490667.21 | 83058989.53 | 152162877.8 | 139644953.1 | 95542721.35 | 142036565.3 | 37726181.61 | 78042505.16 | 38590049.87 |
|  |  | 2.813368848 | 0.415727353 | 0.005978956 | 305.10646 |  | 674299692.8 | 437268306.4 | 455094050.4 | 460807797.3 | 237784197.4 | 323428962.7 | 147288637.3 | 134372875 | 109301086.7 | 303822981.7 | 364635240.5 | 224904396.1 |
|  |  | 3.4922157 | 4.611529312 | 0.006037505 | 95.06048 |  | 31587075.6 | 142844745 | 232358921.5 | 45112219.83 | 322635712.2 | 554248288.8 | 469822223.1 | 737257531 | 359767060.3 | 335390193.1 | 471405206.8 | 4463672.897 |
|  |  | 1.372562517 | 0.558608163 | 0.00605318 | 365.10561 |  | 182326210.9 | 135258043.1 | 142187978.5 | 196581378.5 | 73447970.68 | 114509055.6 | 82190761.29 | 96496697.38 | 164695427.5 | 85403465.26 | 90392985.55 | 161182356.6 |
|  |  | 1.804400967 | 3.277542588 | 0.006063919 | 397.31042 |  | 24109861.2 | 88387724.69 | 54465261.27 | 28847169.03 | 156110861 | 209224049.9 | 101472376.8 | 174968379.5 | 35726784.17 | 90089652.38 | 33121164.94 | 15696067.92 |
|  |  | 1.316083624 | 0.263478048 | 0.006135358 | 223.09767 |  | 109681287.3 | 67924673.23 | 61679523.35 | 127769727.8 | 18762492.05 | 20417405.31 | 32924866.66 | 24606226.7 | 71461932.23 | 75475483.18 | 80302045.77 | 43969521.37 |
|  |  | 1.013007691 | 0.516256269 | 0.006165173 | 343.19799 |  | 79492182.43 | 59205550.76 | 88645044.83 | 80451179.76 | 24153456.81 | 49154889.63 | 33980826.64 | 51611387.04 | 117244425 | 45794668.83 | 65124020.59 | 94242906.32 |
|  |  | 3.11690709 | 0.507227192 | 0.006178757 | 411.14840 |  | 799672870.6 | 809784033 | 779629664.7 | 685306543 | 386667640.3 | 568777039.5 | 450238635.4 | 153732470.8 | 515145673.6 | 523089487.8 | 610876429 | 520757433.6 |
|  |  | 1.163511611 | 0.487576858 | 0.006184909 | 337.13269 |  | 98332124.83 | 94638739.38 | 113540646.7 | 101747818.4 | 49307412.43 | 80564317.58 | 47231222.59 | 21954848.54 | 66724239.41 | 67775453.24 | 110403658 | 86262026.64 |
|  |  | 1.202013876 | 0.5047612 | 0.006198867 | 146.06051 |  | 130467097.6 | 103538761 | 92760352.13 | 120713206.3 | 32459460.3 | 80475857.38 | 65295104.04 | 47639825.77 | 43936336.11 | 72374805 | 50136252.52 | 76408821.94 |
|  |  | 1.11186299 | 0.362118754 | 0.006209843 | 516.20480 |  | 88791521.34 | 52339988.3 | 67214748.41 | 83349479.01 | 30570808.42 | 45908184.78 | 18735007.73 | 10414496.05 | 52462141.99 | 28793358.5 | 21550857.7 | 64258153.06 |
|  |  | 1.594561143 | 0.441123271 | 0.006395051 | 100.05039 |  | 201600471.2 | 204867192.4 | 193227576.1 | 185913038.9 | 152227895.9 | 55814149.54 | 104943977.7 | 33564070.59 | 152385852.3 | 63523469.15 | 527339450.3 | 67231913.82 |
|  |  | 1.282742768 | 0.461514316 | 0.006407742 | 146.06012 |  | 144818219.7 | 97203603.85 | 114554699.9 | 119484565.8 | 36555900.35 | 76512703.24 | 75363632.21 | 31276772.37 | 53304009.07 | 46648073.5 | 178011607.6 | 50217038.71 |
|  |  | 3.926322437 | 0.57949282 | 0.006578644 | 140.00197 |  | 2014009622 | 1350160141 | 1603657943 | 1911717727 | 1143789588 | 979690792.4 | 1125314899 | 737851906.7 | 1668373539 | 1812487724 | 1787118985 | 914171995.9 |
|  |  | 1.610559403 | 0.047190197 | 0.006598445 | 244.90822 |  | 32645802.19 | 134374105.7 | 96636871.78 | 119309329.9 | 3056859.166 | 3276569.438 | 6289505.519 | 5449311.918 | 9574351.051 | 6946297.287 | 98609814.08 | 32342123.6 |
|  |  | 1.691016496 | 0.628959374 | 0.00660638 | 179.00188 |  | 390863367.5 | 275699622.3 | 387545394 | 300339988.7 | 198495368.1 | 200159443.8 | 217547065.5 | 235691122.9 | 380080241 | 248297129.6 | 313051557.7 | 398890380 |
|  |  | 1.138630294 | 0.662515702 | 0.006609222 | 225.00744 |  | 188995001.2 | 133110795.3 | 179907787.8 | 177746568.9 | 113623732.4 | 130520731.7 | 101409811.5 | 104797499.3 | 191751061 | 143627748.8 | 187821680.3 | 181719370.3 |
|  |  | 1.216707493 | 0.65837187 | 0.00665941 | 293.17596 |  | 171479767.3 | 182328914.6 | 223162602.1 | 184450273.4 | 117018308.6 | 158854102.8 | 117030009.2 | 108396113.9 | 90436267.84 | 126824995 | 135841687.6 | 87175959.51 |
|  |  | 4.186119359 | 0.378879509 | 0.006662731 | 335.19667 |  | 1320518130 | 911500324.5 | 788256840.1 | 1261064696 | 327965834.1 | 663901865.1 | 435794833.3 | 194449461.8 | 918718026.9 | 400717340 | 793772341.1 | 1162785297 |
|  |  | 2.213363516 | 0.55620433 | 0.006700239 | 405.28625 |  | 486899918.4 | 333371759.9 | 396842700.4 | 451195794 | 190985947.9 | 324493174.9 | 207079783 | 205362436.2 | 334333555.9 | 308444718.2 | 248715607.6 | 407514804.5 |
|  |  | 7.711420676 | 0.584877992 | 0.006732401 | 144.08086 |  | 5953433333 | 5731557483 | 5147697941 | 4745738435 | 2518167532 | 4358321274 | 2260140229 | 3484118131 | 3114247978 | 3146103712 | 7919487205 | 2819322965 |
|  |  | 1.104620452 | 2.781096333 | 0.006779827 | 269.21132 |  | 21159477.74 | 25217770.78 | 13713006.62 | 22005089.71 | 70082837.26 | 63436253.33 | 63414028.49 | 31381943.4 | 30216924.48 | 77560011.08 | 52885918.2 | 44470643.04 |
|  |  | 1.608695917 | 2.523263832 | 0.006783692 | 431.31587 |  | 45607451.98 | 41627711.57 | 50932045.21 | 76038469.2 | 160333401.2 | 80632628.05 | 157978463 | 141552947.7 | 62250444.23 | 169601567.9 | 33424517.36 | 30967053.05 |
|  |  | 6.268051206 | 0.481141357 | 0.006843964 | 209.01256 |  | 3449565854 | 1954728019 | 2710941487 | 3121787901 | 1073244698 | 1809038353 | 1172967737 | 1351345833 | 1704578370 | 1675041257 | 1785136691 | 1441830330 |
|  |  | 2.075749571 | 1.885754522 | 0.006921177 | 403.17951 |  | 118116543.6 | 101878333.5 | 139372761.1 | 232091336.7 | 237607073.8 | 284543053.9 | 306031582.6 | 287164726.3 | 997550145.3 | 1381937501 | 463319475.5 | 239201725.5 |
|  |  | 4.213268739 | 0.546072113 | 0.007163137 | 225.00749 |  | 1648221043 | 1502906479 | 1372915340 | 1454517485 | 444351735.5 | 1216088027 | 863418538.6 | 740866780.2 | 1140109003 | 1232219461 | 1370905414 | 1100510382 |
|  |  | 2.39978793 | 1.616203863 | 0.007168296 | 387.18457 |  | 310725811.5 | 266669352.8 | 253679985.1 | 316231030.5 | 524483839.5 | 449739247.8 | 351855966.8 | 528201625.4 | 950821987.7 | 2153215152 | 238957430.7 | 611474832.4 |
|  |  | 1.21499397 | 0.187608318 | 0.007248857 | 114.09144 |  | 89692966.44 | 81261670.02 | 67694991.55 | 31532765.74 | 3921434.495 | 15449412.77 | 25624321.41 | 5693295.83 | 35842256.23 | 5378312.302 | 44347326.5 | 23554543.68 |
|  |  | 1.527875863 | 0.498122202 | 0.007270284 | 278.06707 |  | 212726091.5 | 133097376.6 | 181923015.1 | 197121244.1 | 89479575.22 | 130476702.8 | 82912931.45 | 58203498.84 | 110429273.1 | 118913327 | 110314164.7 | 113461923.1 |
|  |  | 4.190690662 | 3.103542244 | 0.007356342 | 467.27788 |  | 308203588.1 | 172451474.6 | 202671320.9 | 321228143.7 | 873372164.4 | 1042880439 | 764312272.1 | 437112536.4 | 4741337921 | 899656574.2 | 545440573.3 | 1224469755 |
|  |  | 1.027438481 | 0.619843522 | 0.007359861 | 308.00588 |  | 121605009.3 | 129975938.5 | 111974853.1 | 130809583.2 | 56376768.39 | 95395232.1 | 95834978.45 | 58822201.68 | 115102466.3 | 172605149.4 | 164739290.1 | 173906869.4 |
|  |  | 1.039055366 | 1.844919807 | 0.007366432 | 215.10286 |  | 30834819.45 | 49837848.02 | 49333541.29 | 32622753.16 | 82248324.9 | 90017223.4 | 58750086.11 | 69021758.64 | 47399716.22 | 50344709.01 | 45360133.16 | 46959556.23 |
|  |  | 1.220707547 | 0.413903176 | 0.007374241 | 488.27670 |  | 111771250.1 | 68092117.02 | 90623211.01 | 92087923.86 | 8292324.64 | 44911288.03 | 43654004.48 | 53213120.72 | 101121114 | 52701193.09 | 79257814.47 | 15268670.39 |
|  |  | 1.308129316 | 0.629273096 | 0.007469437 | 233.06682 |  | 182619189 | 182039774.9 | 236961613.5 | 185990325 | 117279907.5 | 160765977.6 | 102727434.7 | 114849031 | 221374787 | 271991874.1 | 267985973.7 | 183245309.1 |
|  |  | 1.532077632 | 1.899812318 | 0.007555794 | 175.10755 |  | 96504005.6 | 62846423.5 | 96261806.96 | 69709205.68 | 165232319.4 | 191853799.5 | 114213900.5 | 146749663.1 | 78462014.92 | 98858217.55 | 122251454.1 | 131475575.3 |
|  |  | 1.081180893 | 1.993881005 | 0.00759434 | 461.18526 |  | 33088657.92 | 20201908.14 | 41546696.9 | 49877408.59 | 64464686.66 | 77903483.61 | 87622200.85 | 58553463.67 | 55258654.85 | 58506234.3 | 37658305.11 | 77035282.1 |
|  |  | 1.498259113 | 0.269613486 | 0.007723314 | 253.03891 |  | 146635637.3 | 99192258.79 | 71893926.35 | 156386835 | 39157383.17 | 48996508.22 | 5371591.281 | 34300605.29 | 32144988.9 | 59833701.39 | 60541330.29 | 45424989.4 |
|  |  | 1.562131787 | 0.641554431 | 0.007806951 | 359.32694 |  | 307044310 | 328785041.5 | 229399410.4 | 303686632.9 | 141621427.4 | 211770443.2 | 200555805.8 | 195975174.1 | 304916153.9 | 301230105.6 | 203759896.4 | 302513212.7 |
|  |  | 1.004641801 | 3.302949738 | 0.008221757 | 379.24889 |  | 12459211.11 | 7958001.131 | 9225973.962 | 25023315.44 | 59500444.71 | 54446265.58 | 37560796.74 | 29053200.21 | 34884305.49 | 23174061.41 | 7161269.803 | 11544005.71 |
|  |  | 2.906456779 | 1.90579707 | 0.008260745 | 431.21086 |  | 159052657.7 | 437536297.4 | 200372735.4 | 343808694.4 | 590242521.4 | 533506866.2 | 505846132.4 | 544481337.5 | 287309525.3 | 481271115.7 | 211443843.9 | 225863169.2 |
|  |  | 7.632191588 | 5.981125709 | 0.008326019 | 467.29940 |  | 730011167.6 | 11824233.53 | 8152653.046 | 685469294.2 | 2881833590 | 2483638874 | 2262312798 | 957865588.5 | 3108282852 | 1908236047 | 1264515199 | 3297737988 |
|  |  | 2.290703194 | 0.583710887 | 0.008389016 | 161.10739 |  | 541683718.3 | 509772326.8 | 465865708 | 405296523.6 | 228062234.8 | 386924657.5 | 197501671.9 | 309764655.8 | 268314276.2 | 274543831.3 | 660948543.1 | 240750639.9 |
|  |  | 1.708496979 | 0.481443075 | 0.008408878 | 159.07654 |  | 290006940.4 | 214578754.6 | 161544852.5 | 252830522.5 | 125747249.5 | 141728499.3 | 78945817.87 | 96005876.88 | 221009850.1 | 148215869.1 | 97505716.86 | 203310404.4 |
|  |  | 1.142571196 | 0.460053991 | 0.008526714 | 173.07100 |  | 87112229.36 | 94309797.88 | 65360129.05 | 105955573.7 | 24436973.69 | 50057936.87 | 26656264.06 | 61127225.78 | 81764568.62 | 53638775.56 | 54678279.23 | 42774352.92 |
|  |  | 2.999812383 | 0.513916411 | 0.008548892 | 73.02928 |  | 743439812.7 | 729693343 | 667750405.8 | 731736693.2 | 359206727.8 | 624536295.5 | 253364716.9 | 239178950.1 | 602623594.4 | 411829727.4 | 325208350.6 | 535606393.8 |
|  |  | 1.056181929 | 0.49071208 | 0.008630671 | 249.12693 |  | 109944223.9 | 65408977.76 | 80906723.99 | 98702165.62 | 47887870.82 | 57883795.79 | 41789240.17 | 26623279.24 | 78328373.73 | 76742684.5 | 58461848.89 | 80541338.92 |
|  |  | 1.78611738 | 0.316525072 | 0.008800082 | 291.09079 |  | 250754825.1 | 127243274.1 | 199286457.7 | 131127901.4 | 70573326.56 | 80260372.55 | 39029101.42 | 34367503.75 | 149274589.6 | 63669324.09 | 143624598.6 | 56522904.35 |
|  |  | 1.300094571 | 0.551412011 | 0.008884127 | 172.10866 |  | 189621973.6 | 158342611.2 | 131474597.7 | 124563724.7 | 66141720.83 | 108201853.3 | 89977435.43 | 68733448.25 | 164658097.6 | 133812803 | 282058004.5 | 292860591.2 |
|  |  | 3.552120214 | 0.122403795 | 0.009018699 | 696.31184 |  | 632935227.2 | 255096709.2 | 523148963.2 | 886027403.9 | 65963655.15 | 41163915.72 | 137476723.6 | 36582719.17 | 399745572.4 | 53995623.24 | 126640735.9 | 103317383.4 |
|  |  | 1.345901355 | 0.604451173 | 0.009249127 | 227.02318 |  | 204023254.4 | 191591216.5 | 184680467 | 204275969.8 | 139249744.2 | 162428315.6 | 99605958.32 | 72950787 | 167282406.7 | 165143116.6 | 205844958.7 | 134973874.9 |
|  |  | 3.75578492 | 2.661594823 | 0.009284795 | 555.29983 |  | 248972215.6 | 385186055.6 | 208203491.3 | 317068058.8 | 998643295.1 | 753513929.5 | 439030293.6 | 894744891.6 | 283829578.6 | 557473003.5 | 238117918.7 | 88037637.53 |
|  |  | 2.123407919 | 0.292707494 | 0.009345254 | 363.21767 |  | 341119259.2 | 141189752.7 | 259849401.3 | 208880309.6 | 39944588.06 | 94544827.89 | 95380380.25 | 48506365.13 | 109983929.9 | 63443903.17 | 126052489.7 | 203797907.5 |
|  |  | 2.033904911 | 0.630729596 | 0.009393109 | 142.03463 |  | 554497884.8 | 489816172.9 | 422364654.3 | 386948359.3 | 221909986.9 | 349368426 | 302392549.2 | 295466492.2 | 549590043.7 | 549378552.6 | 369594979.4 | 230617344.4 |
|  |  | 4.999185003 | 0.519424247 | 0.009399607 | 454.29309 |  | 2299067353 | 2131432587 | 1842424214 | 1830838699 | 707396246.7 | 1660281815 | 1169799879 | 671812978 | 3041219064 | 1408641174 | 1783130404 | 1929268427 |
|  |  | 1.432518739 | 0.574561456 | 0.009859095 | 580.32814 |  | 243655918.7 | 184213433.7 | 209130550.3 | 226329147.7 | 75244749.1 | 139312667.2 | 174001232 | 107476947.7 | 81437634.57 | 116994991.3 | 132323117 | 116030469.8 |
|  |  | 1.039092579 | 0.586230226 | 0.010088855 | 163.04155 |  | 131926862 | 78654368.97 | 103194624.7 | 118953976.2 | 58377523.64 | 60065263.17 | 60275269.79 | 74961250.4 | 65180344.45 | 91986173.9 | 64497983.27 | 63194217.29 |
|  |  | 1.154725029 | 0.447477724 | 0.010171242 | 330.17670 |  | 78730086.32 | 93640246.81 | 74076454.66 | 83971632.66 | 20974382.24 | 35965998.32 | 20754659.87 | 70159842.41 | 116494919.2 | 59412403.99 | 84873223.44 | 88947940.88 |
|  |  | 1.745152415 | 3.253017011 | 0.010177577 | 233.14973 |  | 65031813.2 | 36549308.24 | 25952567.53 | 44243856.13 | 140305325.9 | 209775079.1 | 106655685.2 | 102059186.1 | 31872929.48 | 22645283.53 | 29219752.8 | 34326640.36 |
|  |  | 1.668934959 | 3.309242986 | 0.010177749 | 138.10265 |  | 10579543.88 | 77219275.69 | 51512211.48 | 17935434.66 | 133144203.4 | 76014107.49 | 164236082.5 | 146972370.3 | 113669438.2 | 76119108.94 | 74176277.66 | 80755570.72 |
|  |  | 2.74616654 | 0.448056352 | 0.010303577 | 87.04504 |  | 666384249 | 456821971.6 | 426423191 | 762542694.4 | 306345241.7 | 304977173.5 | 181913182.8 | 242747800.6 | 663065152.2 | 418429564 | 380393935 | 498368389.6 |
|  |  | 1.972351519 | 2.879234032 | 0.010357492 | 465.32201 |  | 68753369.65 | 95837371.18 | 65745578.17 | 58202469.04 | 193276998.5 | 283472994.9 | 115119669.6 | 238901035 | 68371364.41 | 99941531.45 | 72435031.24 | 78173289.91 |
|  |  | 1.476559172 | 0.456651489 | 0.010387797 | 330.26401 |  | 157162504 | 127403422.7 | 187465135.6 | 148774764.1 | 57744868.38 | 124641806.5 | 67422442.57 | 33682787.66 | 147133385.2 | 47989673.12 | 119523654.5 | 154361585.2 |
|  |  | 5.584477165 | 0.590803614 | 0.010446489 | 225.00750 |  | 3402654663 | 2477746612 | 2780953320 | 3334142404 | 1465737750 | 2514810798 | 1604986454 | 1501447973 | 2750632115 | 2863546898 | 3144062462 | 2651386135 |
|  |  | 8.329886532 | 0.50257097 | 0.010553641 | 209.01255 |  | 5632914710 | 4755132641 | 4017963922 | 6704529677 | 1869785279 | 2912026403 | 2098900236 | 3728833131 | 2519021177 | 4418145678 | 2638433081 | 3783005608 |
|  |  | 1.177849285 | 0.442207937 | 0.010710732 | 432.17461 |  | 95058995.76 | 135920864.6 | 80967577.17 | 84086932.3 | 35583827.97 | 57537263.04 | 57581217.9 | 24427232.64 | 119994461.3 | 71884347.27 | 36785531.4 | 209919293.4 |
|  |  | 1.420693611 | 0.545902912 | 0.01071384 | 196.02857 |  | 211826209.5 | 158156124.8 | 151156156.9 | 231604334.6 | 100802388.8 | 136416342.5 | 97341853.73 | 76363915.85 | 117570112.1 | 104482976.7 | 152722115.6 | 263386341.1 |
|  |  | 1.247115483 | 2.560453389 | 0.010772901 | 857.50159 |  | 33530486.02 | 30990911.56 | 31635563.64 | 29594552.84 | 54193253.49 | 81074360.44 | 69451312.57 | 117261963.9 | 77190520.9 | 57848330.52 | 41823076.74 | 24636981.67 |
|  |  | 1.367642414 | 0.579915132 | 0.010798748 | 286.31069 |  | 253540181.7 | 172052735 | 202680355.8 | 172202211.7 | 131947775.7 | 126216348.5 | 128200081.6 | 77843640.35 | 199976204 | 97181596.41 | 180734520.9 | 234952506.9 |
|  |  | 1.136524538 | 0.162160823 | 0.010819433 | 245.01269 |  | 60447284.11 | 69254988.35 | 78542828.27 | 19750013.6 | 4403403.409 | 10632341.32 | 7874157.442 | 14061973.12 | 3093586.192 | 4049701.381 | 12265228.32 | 6135339.487 |
|  |  | 4.319712085 | 0.624288972 | 0.01169688 | 163.00703 |  | 2277125929 | 1649744634 | 1916872929 | 2005471409 | 1090713559 | 1702338854 | 1005516543 | 1101609343 | 1456264364 | 1668270660 | 1862739555 | 1550128013 |
|  |  | 1.32175196 | 3.91886821 | 0.011826481 | 487.30726 |  | 20412259.74 | 7527987.006 | 4890975.299 | 44481757.89 | 103592695.6 | 91942223.01 | 56212046.39 | 51232414.33 | 29780832.39 | 92450117.36 | 11244619.72 | 33791773.22 |
|  |  | 1.973488777 | 1.958865153 | 0.011830721 | 567.26382 |  | 106688601.9 | 208129065.7 | 90215883.02 | 115755905.6 | 306421220.3 | 278386908.2 | 205615213.1 | 229732976.3 | 148549652 | 214206782.1 | 76749747.14 | 70130042.13 |
|  |  | 1.414535356 | 1.861672355 | 0.012109609 | 467.24733 |  | 63266259.72 | 96678494.26 | 90297362.62 | 43924193.13 | 130603498.4 | 155334044.8 | 158938331.4 | 102765411.9 | 80026103.46 | 54525574.97 | 46686262.76 | 22650757.2 |
|  |  | 1.128034202 | 0.322562633 | 0.012199449 | 512.27361 |  | 85605345.73 | 49156293.44 | 92863846.32 | 49194273.62 | 8795584.936 | 22640318.55 | 18885815.33 | 38969991.48 | 68031870.13 | 42409489.14 | 58855770.94 | 28335638.1 |
|  |  | 1.836703521 | 0.504243931 | 0.01223785 | 289.16582 |  | 306023398.2 | 199149012.7 | 259953382.2 | 375930317.4 | 141137794.1 | 181705178.2 | 112143183.5 | 140384463 | 176832833.2 | 214848634.5 | 229390838.3 | 272359842 |
|  |  | 1.83699141 | 0.393330962 | 0.012306175 | 389.25434 |  | 148844402.3 | 187971026.3 | 230900058.2 | 311254981.6 | 67351151.26 | 97655518.12 | 58589338.59 | 122130291.7 | 161550731.9 | 205246266.4 | 85059266.98 | 151170007.5 |
|  |  | 1.7243893 | 0.667967075 | 0.012335107 | 194.99674 |  | 453669708.2 | 357614878.8 | 378360902 | 443674851.8 | 285300345.7 | 345791843.5 | 259532537.5 | 200379484.5 | 392972591 | 301755266.5 | 461427305.5 | 454463796.2 |
|  |  | 3.416385022 | 0.388091184 | 0.012367078 | 298.09705 |  | 840896053.8 | 418146736.9 | 715015907.2 | 893451151.7 | 196484593.5 | 231702177.4 | 214985079.5 | 469683443.6 | 916993879.3 | 780151445.4 | 269325362.8 | 1313478410 |
|  |  | 1.086618939 | 0.304847755 | 0.012446547 | 303.13766 |  | 42438749.86 | 50251002.7 | 20821257.33 | 35275414.54 | 12088935.31 | 4177763.604 | 7346614.297 | 21743894.2 | 30557437.79 | 31964104.66 | 21339373.24 | 45570095.71 |
|  |  | 2.118338583 | 0.621017376 | 0.012759646 | 216.10198 |  | 551244616.7 | 437488079.5 | 432558435.4 | 573010081.4 | 265586526.6 | 422524845 | 301903415.1 | 248480919 | 363897065.8 | 335336337.4 | 380231432 | 398237789.7 |
|  |  | 2.168830913 | 0.428933603 | 0.01278918 | 238.98679 |  | 320891170 | 213352150.6 | 466353420 | 345322243.4 | 114921676.3 | 193094533.5 | 121597274.7 | 147696395 | 109976851.7 | 130204406.2 | 102437202.4 | 102606118.9 |
|  |  | 1.02188133 | 0.527992483 | 0.012838352 | 252.15963 |  | 69737265.4 | 78249767.52 | 78612466.92 | 83267837.86 | 30600229.52 | 36673504.4 | 25970145.21 | 70363745.75 | 59136670.83 | 74742023.06 | 61319747.09 | 54830906.57 |
|  |  | 3.879764831 | 0.519937818 | 0.01287703 | 235.06459 |  | 1429190940 | 864577361 | 1323139645 | 1250504304 | 513630037.8 | 961314538.5 | 595580940.4 | 460226188.8 | 1015530980 | 853349297.7 | 1104106625 | 930400908.6 |
|  |  | 1.04029712 | 0.411561121 | 0.012890026 | 393.21055 |  | 49225492.34 | 78193256.12 | 107090253.2 | 62457681.42 | 27478297.55 | 30446749.85 | 32333871.67 | 31961021.76 | 447900350.7 | 135464629.9 | 47745833.33 | 48052709.5 |
|  |  | 2.163219515 | 0.418266628 | 0.012923824 | 181.01763 |  | 430314469.8 | 282418278 | 373289126.5 | 242730131.8 | 207962774.8 | 191477176.7 | 79370152.37 | 76962517.75 | 246488003.6 | 161847871.2 | 220049152.6 | 197793555.1 |
|  |  | 1.199508801 | 6.948085202 | 0.012935308 | 530.31492 |  | 2166901.202 | 9480710.817 | 225602.2968 | 19471041.14 | 59615494.45 | 86828237.52 | 43669700.43 | 27669125.13 | 35111387.32 | 42391661.62 | 1307419.364 | 10234342.22 |
|  |  | 1.899963165 | 6.223109441 | 0.012971934 | 485.25750 |  | 11635087.62 | 25196993.58 | 20027451.67 | 34729976.37 | 179067063.4 | 46030921.81 | 198604779.5 | 146268775 | 32916489.62 | 100945007.7 | 28804132.39 | 31627385.6 |
|  |  | 1.927356761 | 0.502510705 | 0.012976394 | 271.10112 |  | 324716002.8 | 215243762.9 | 377616264.3 | 295171730.2 | 126882337.3 | 226078084.8 | 154294268.5 | 102164041.3 | 308634066 | 23175881.92 | 317670783.7 | 283444148.7 |
|  |  | 1.01470939 | 3.352620487 | 0.012989093 | 407.27781 |  | 23695488.19 | 7774696.08 | 1401531.522 | 22806021.28 | 38408412.66 | 42921273.5 | 36336234.32 | 69000401.49 | 46711824.52 | 39679398.62 | 34223303.31 | 21593713.37 |
|  |  | 1.265853747 | 0.573884817 | 0.013060855 | 427.14278 |  | 152383686.9 | 135863769.8 | 178972734.5 | 159999257 | 59041284.06 | 133324152.5 | 100204455.4 | 67381826.48 | 108954665.1 | 217164961.6 | 126073049.9 | 119034894.1 |
|  |  | 1.005974737 | 0.385661555 | 0.013205415 | 378.19945 |  | 68040368.37 | 36337220.6 | 88071024.92 | 65631737.86 | 17286722.93 | 34231621.45 | 18951566.79 | 29061758.65 | 70639742.29 | 32532834.94 | 20775396.93 | 57128313.46 |
|  |  | 1.461342065 | 1.513628776 | 0.013264556 | 326.30557 |  | 84135776.39 | 136904720.7 | 116608525.1 | 156740958.9 | 170895533.3 | 207072618.5 | 170006562.4 | 200348187.7 | 266226360 | 232967944 | 168347832.4 | 207220092.7 |
|  |  | 1.24063556 | 0.569021867 | 0.013285854 | 230.13848 |  | 158193034.4 | 155850620.5 | 115850359.1 | 165495161.3 | 68914240.41 | 126397122.1 | 83411033.28 | 60067064.57 | 130112790.4 | 158450475.1 | 157161137.7 | 246294589.2 |
|  |  | 2.163157175 | 0.17585616 | 0.013401775 | 355.08560 |  | 332431624.5 | 112620259.9 | 256240583.3 | 154989373.3 | 31294971.39 | 59602869.61 | 51209072.71 | 8475522.411 | 71601720.12 | 81225806.39 | 172478434.3 | 121858753.9 |
|  |  | 1.420996506 | 0.565623522 | 0.013497072 | 315.12398 |  | 229779222.8 | 184292882 | 151583317.5 | 259627328.5 | 110589943.2 | 144511070.4 | 114209626.2 | 97488696.11 | 73905028.48 | 110056357.8 | 100924716.5 | 230320064.5 |
|  |  | 4.509725918 | 2.808577127 | 0.013576587 | 445.19002 |  | 211557139.8 | 305723605 | 600509833.4 | 414550483.9 | 584584419.9 | 1087518628 | 1169661928 | 1461933082 | 384715627.3 | 985194686.1 | 422267398.5 | 705488725.4 |
|  |  | 2.630074321 | 0.518862208 | 0.013748098 | 430.16221 |  | 555848016 | 723329540.2 | 556674584.7 | 448324173.5 | 238315905.9 | 463152807.3 | 224666000 | 259038053.2 | 208152367.6 | 364515608.3 | 646409926 | 335328964.4 |
|  |  | 1.369374828 | 2.530196249 | 0.013764258 | 480.31464 |  | 8791870.593 | 62760806.17 | 14738952.63 | 68208618.01 | 115610372.6 | 100294083.2 | 93997468.96 | 81014021.78 | 74891150.59 | 134472905.5 | 10076486.15 | 28739999 |
|  |  | 1.224813716 | 0.50150608 | 0.013775158 | 316.21201 |  | 137072313.5 | 93791966.46 | 101519662.7 | 113446235.1 | 35291604.18 | 94288505.43 | 47164928.84 | 46841506.43 | 104498904.1 | 102900970.1 | 125290001.9 | 138886637.3 |
|  |  | 3.545370465 | 0.331115986 | 0.013902248 | 365.10551 |  | 958468213 | 495154031.3 | 529134103.1 | 826961765 | 81955334.91 | 434690737.3 | 260312043.4 | 153384467 | 225578877.8 | 269631861.9 | 269637794.8 | 431999645.1 |
|  |  | 1.25411447 | 0.613566207 | 0.014007962 | 171.06635 |  | 205343304.8 | 165870922.2 | 135322049.5 | 191771110.4 | 70587821.98 | 118934319.3 | 121688314.2 | 117247359.2 | 108791837 | 111868930.1 | 159581295.4 | 115828192.9 |
|  |  | 3.652491537 | 0.442421988 | 0.014182254 | 345.22828 |  | 1310186941 | 738117068.2 | 930718462.7 | 722067571.2 | 250195977 | 528060328.7 | 495462814.2 | 363724495.7 | 554201778.6 | 427850073.3 | 568326563.2 | 690037686.9 |
|  |  | 1.305362003 | 0.536707981 | 0.014288392 | 261.11334 |  | 189003683.8 | 119775342.3 | 136230600 | 158754067.5 | 90197491.68 | 116493703.9 | 57466705.09 | 59886892.26 | 112443148.7 | 107215889.5 | 91381783.72 | 124327321 |
|  |  | 1.432950584 | 0.470019569 | 0.014347125 | 213.00754 |  | 154143484.9 | 111916381 | 225945764.9 | 169625078.9 | 79366994.28 | 103006847 | 52790853.8 | 75814686.2 | 99826735.85 | 186391915.4 | 119347097.4 | 50883665.76 |
|  |  | 1.013562712 | 0.535211079 | 0.014434565 | 142.06201 |  | 110260157.1 | 74678662.13 | 107709253.7 | 64783936.86 | 40143056.76 | 58484195.04 | 43430686.44 | 49243633.37 | 108088210.7 | 69487407.36 | 84363960.26 | 66039702.4 |
|  |  | 1.019849294 | 0.400493381 | 0.014454112 | 246.04642 |  | 2068867.887 | 3530289.907 | 1844391.939 | 1998128.431 | 675058.3861 | 1021201.74 | 790557.65 | 1294511.839 | 2016192.121 | 2784097.27 | 2046304.334 | 2114207.432 |
|  |  | 2.828286851 | 1.99390717 | 0.014478873 | 355.26336 |  | 198506168.6 | 342070141.8 | 299880901.6 | 290814205.6 | 347582566.6 | 669458315.3 | 678152136.8 | 560457171.7 | 297585285.8 | 714147448.5 | 350669424.8 | 168685946.1 |
|  |  | 3.737699308 | 2.950619219 | 0.014499069 | 96.05597 |  | 130323205.6 | 315563007.1 | 243752662.7 | 240890378.8 | 680351781.4 | 549360532.8 | 468844992.4 | 1047080194 | 45797842.48 | 265916084.3 | 322391665.1 | 482236791.9 |
|  |  | 1.670539362 | 0.36892094 | 0.014633288 | 251.02322 |  | 158641804.7 | 163234722.2 | 220914392.5 | 150178659.4 | 7135891.669 | 133725443.2 | 81516597.61 | 33273055.67 | 117798422.1 | 119485303.3 | 132979248.2 | 163287715 |
|  |  | 2.573130561 | 0.48135983 | 0.014790949 | 361.20606 |  | 378680377 | 704775482 | 450092098.6 | 471588944.7 | 191331377.3 | 195870584.1 | 318927516.6 | 259062881 | 298890105 | 445101058.6 | 211264628.1 | 730060040.8 |
|  |  | 1.094095081 | 0.571921685 | 0.014845295 | 194.99694 |  | 126265510.1 | 77022914.66 | 121481463.9 | 107320507.3 | 49296546.19 | 81551984.38 | 48226288.39 | 68047048.37 | 67739468.04 | 74757256.39 | 155784261.9 | 56036848.72 |
|  |  | 2.809523393 | 2.995120431 | 0.014857677 | 316.13459 |  | 33394604.69 | 306903046.6 | 117115647.3 | 67923215.78 | 502149894.7 | 320240149.3 | 306030182.5 | 445025900.9 | 420593297.7 | 35783051.72 | 4212384.259 | 168624712 |
|  |  | 1.014174165 | 0.511304971 | 0.014890707 | 375.11175 |  | 88693248.5 | 88463223.2 | 86338255.07 | 85636224.36 | 60987320.09 | 63772062.87 | 44789839.91 | 8963167.908 | 57131399.17 | 62288581.31 | 98254077.63 | 80687088.23 |
|  |  | 2.189250775 | 0.295179736 | 0.014995584 | 258.09501 |  | 380124070.5 | 280908821.8 | 123178377.2 | 325332468.7 | 49755523.84 | 115047010.7 | 109177835.7 | 53534457.98 | 193298024.2 | 51276373.87 | 164567849.9 | 259112834.6 |
|  |  | 1.42463676 | 0.540881178 | 0.015348958 | 373.09596 |  | 220211436.5 | 125927483.5 | 206416586 | 189576095.9 | 103961863.5 | 136764238.5 | 94370666.08 | 66308247.39 | 94339437.12 | 118976987.8 | 129480581.2 | 107532371 |
|  |  | 2.611328274 | 0.635607117 | 0.015463267 | 191.00200 |  | 853020212.1 | 626134363.1 | 796993914.4 | 775479861.4 | 416336478.2 | 685855224 | 438220001.6 | 399224993.8 | 721678894.2 | 676559210.9 | 823278158 | 741413201.3 |
|  |  | 1.209392192 | 0.613519252 | 0.015573514 | 124.03946 |  | 201646982.1 | 155319512.3 | 149962772.6 | 198476064.1 | 122561961.7 | 133405073.3 | 111834966.2 | 64977749.88 | 188529416.6 | 133283414.2 | 116760974 | 266372087.3 |
|  |  | 1.610728195 | 3.19346184 | 0.015723976 | 269.21126 |  | 45180022.29 | 51883123.45 | 23797260.68 | 24124860.42 | 156846690.6 | 132085744.9 | 123036758.9 | 51035722.67 | 55313759.27 | 80652304.4 | 67450731.03 | 100212795.5 |
|  |  | 1.179148506 | 0.118804644 | 0.015792355 | 505.22769 |  | 53173476.83 | 29908013.63 | 110382577.6 | 66745647.83 | 3649716.921 | 10951503.71 | 14980670.91 | 1332231.198 | 14332192.81 | 32447468.22 | 56457435.29 | 36010415.28 |
|  |  | 1.05145593 | 0.611210317 | 0.015821392 | 260.14938 |  | 125252289.1 | 98225585.14 | 104956820.3 | 102086959.3 | 51520673.52 | 91190519.6 | 43662067.71 | 76766015.55 | 195814546.2 | 102405107.8 | 117517586.8 | 120425112.8 |
|  |  | 2.339872246 | 0.362562872 | 0.015900225 | 235.06486 |  | 497158289.2 | 237575565.5 | 244351761.8 | 454112681.6 | 143288586.2 | 140647806.9 | 119439340.1 | 116248758.3 | 106540487.9 | 137255830.7 | 196874230.6 | 161340139.8 |
|  |  | 2.346504574 | 0.517616834 | 0.015951898 | 273.17086 |  | 511582194.2 | 296848076.3 | 415696477.4 | 420628876 | 137107241.7 | 233343046.1 | 163569623.1 | 317333287.2 | 169148216 | 159824279 | 257192668.9 | 196035826.7 |
|  |  | 4.093804646 | 0.539423998 | 0.016027636 | 80.96500 |  | 1460292183 | 1410564500 | 1494397243 | 1326590333 | 388048683.6 | 1309621476 | 708165390.2 | 664481836.2 | 779879430.4 | 1404318523 | 1896971408 | 675854035.1 |
|  |  | 1.156342897 | 0.571697372 | 0.016377387 | 361.20577 |  | 125110879.8 | 136443625.3 | 121724943.6 | 146251655.4 | 27098956.55 | 89032169.87 | 94835705.1 | 91764709.03 | 122044725.3 | 252900168 | 111112200.6 | 69155269.55 |
|  |  | 11.50393778 | 0.614708393 | 0.016439612 | 225.00753 |  | 16853059393 | 10226237801 | 13783690245 | 15897245359 | 7646729110 | 10973035078 | 7839721058 | 8431506233 | 9265217828 | 9391985432 | 11596684448 | 9711595156 |
|  |  | 1.148118373 | 0.54148689 | 0.016521507 | 431.04412 |  | 129718359.4 | 98258274.31 | 143624387.6 | 118319083.7 | 55611527.26 | 95523664.27 | 82025016 | 32125106.31 | 87239971.59 | 74260052.22 | 110081874.3 | 81882429.69 |
|  |  | 1.596786714 | 0.51798324 | 0.016643574 | 489.30748 |  | 247538613.2 | 153790816.9 | 181692120.2 | 209495763.3 | 70475140.03 | 164416459.1 | 84428109.07 | 91190977.5 | 108677207.5 | 91424738.13 | 110564200.5 | 132429086.5 |
|  |  | 1.011045975 | 0.553211485 | 0.016701581 | 210.07731 |  | 122707838.8 | 76450082.16 | 74389190.25 | 99184156.68 | 48831478.58 | 67790846.44 | 41395132.89 | 48181760.25 | 71922412.12 | 61570580.2 | 78537639.55 | 64616876.3 |
|  |  | 1.279836265 | 0.544947084 | 0.016770825 | 188.00236 |  | 159162523.1 | 197424882.8 | 123810339.1 | 114148758.2 | 62427289.62 | 101500644.9 | 76084092.51 | 83984356.28 | 87744836.38 | 169383985.9 | 189366094.9 | 68239935.93 |
|  |  | 1.085942497 | 0.569177639 | 0.016802073 | 222.97412 |  | 122251487.5 | 95288568.69 | 117842231.4 | 102667748.5 | 56948580.27 | 98599634.65 | 56359581.73 | 37420488.51 | 88675416.26 | 94146202.87 | 149112795.4 | 108795770 |
|  |  | 1.178217399 | 0.310021521 | 0.016831905 | 273.19221 |  | 84505877.44 | 46531813.87 | 132309988.8 | 91131663.3 | 21414709.29 | 16444653.61 | 45062200.82 | 26974661.45 | 17669943.38 | 11758514.2 | 55106173.6 | 26878449.66 |
|  |  | 1.149857924 | 0.642933944 | 0.016881235 | 180.06665 |  | 191211288.3 | 144735468 | 152861738.3 | 219783707.8 | 100065014.8 | 132947792.7 | 122383177.9 | 100181994 | 165010646.5 | 127279888.7 | 327967173.9 | 161886719.6 |
|  |  | 1.460869081 | 0.374378071 | 0.016885574 | 208.13330 |  | 154697980.5 | 80792233.76 | 109033219.5 | 133018936.8 | 19405350.29 | 49502990.61 | 20790523.8 | 89082526.92 | 108377141.8 | 75073102.5 | 136360554.1 | 212268826.5 |
|  |  | 2.147762127 | 0.573017053 | 0.016970275 | 124.02394 |  | 652167515.5 | 431267475.4 | 550384503.2 | 366914126 | 331914723.7 | 282651032.9 | 261367621.4 | 270521104 | 455680473.8 | 603414068.8 | 615134518.8 | 197788454.2 |
|  |  | 1.58441848 | 2.880578357 | 0.016977962 | 471.27324 |  | 50676472.41 | 33152855.89 | 37634176.09 | 64606135.81 | 170235772 | 113390924.1 | 70898720.94 | 181462761.6 | 40082009.59 | 40634245.99 | 34076189.95 | 27022645.54 |
|  |  | 1.526682233 | 0.621923343 | 0.017098932 | 256.05945 |  | 280103836.1 | 219563545.8 | 231617167.1 | 326314125 | 119848498.7 | 154289019.1 | 176296106.3 | 207311678.7 | 314586872.9 | 197692144.1 | 179489435.6 | 293923778.5 |
|  |  | 1.045119347 | 0.422858751 | 0.017105149 | 98.06010 |  | 96837671.87 | 59058690.42 | 118615744.3 | 65268711.06 | 51355967.74 | 36549763.02 | 26739321.66 | 29034239.89 | 59008749.21 | 58732511.57 | 47015339.68 | 66259230.31 |
|  |  | 1.384344236 | 0.47607617 | 0.017177907 | 99.04515 |  | 175648350 | 115307737.9 | 161769060.4 | 124715473.2 | 75483202.76 | 116978120.8 | 46087311.59 | 36357084.66 | 59488418.55 | 65734348.01 | 138790064.7 | 88371253 |
|  |  | 1.37092345 | 0.374778298 | 0.017186947 | 222.99195 |  | 110200910.5 | 93354465.34 | 198211019.3 | 115459699.4 | 38023369.23 | 63744831.05 | 60453546.46 | 31623368.57 | 56250364.48 | 115719013.7 | 137677183.9 | 43891662.2 |
|  |  | 3.230000624 | 0.424683952 | 0.017323552 | 263.05960 |  | 669156030.8 | 518213484.2 | 989085574.6 | 927546994.5 | 330402254.4 | 542286793.3 | 297679900.4 | 147850924.4 | 541762798.1 | 808679661.1 | 696754568.8 | 601205509.1 |
|  |  | 5.441062242 | 0.583773907 | 0.017482227 | 206.99695 |  | 3036605176 | 2699469440 | 2752712837 | 3180834250 | 1470342570 | 2721060637 | 1554485043 | 1066532410 | 2566258797 | 2605173827 | 3853178848 | 3401211876 |
|  |  | 3.902693419 | 0.295031098 | 0.017842887 | 512.26865 |  | 685836339.4 | 1276887405 | 756431710.6 | 501860719.1 | 86000136.37 | 246268372.6 | 256157934.6 | 361873494.8 | 421250413.2 | 109134379.3 | 120337981.9 | 96729118.24 |
|  |  | 1.091237893 | 3.185923645 | 0.017979228 | 221.05685 |  | 19777352.12 | 8552673.015 | 30717672.59 | 17088586 | 55748039.52 | 91267395.05 | 32754129.96 | 62794822.03 | 16024228.4 | 15983262.21 | 13534455.59 | 8216185.336 |
|  |  | 2.185309454 | 2.080703698 | 0.017980796 | 531.29991 |  | 37544513.6 | 217209683.2 | 108119682.6 | 205683135.2 | 271637572.5 | 295838364.6 | 355469729.8 | 260053015.8 | 170653168.7 | 217367358.5 | 106009915.5 | 104826517.1 |
|  |  | 1.027499395 | 0.490296163 | 0.018091456 | 347.22271 |  | 104853068.7 | 94597689.83 | 87186998.09 | 121223634.4 | 88957895.83 | 45419771.36 | 45832554.78 | 19762653.31 | 25722204.03 | 48592062.9 | 97189177.69 | 22589170.08 |
|  |  | 1.860097348 | 3.903670872 | 0.018145139 | 469.28336 |  | 43965557.1 | 40715112.2 | 27608690.15 | 32932411.44 | 170027931.9 | 185443696.9 | 166514272.8 | 44912095.33 | 175098498.9 | 125074878.6 | 160143339.9 | 167963988.4 |
|  |  | 1.035405271 | 0.662908819 | 0.018259332 | 196.09610 |  | 149454031.2 | 153156554.1 | 122771546.7 | 153366995.8 | 99911677.41 | 130709813.5 | 84674415.22 | 68361994.33 | 151106803.2 | 117317960.6 | 135534706.3 | 111801849.4 |
|  |  | 2.860411729 | 0.624236338 | 0.018262596 | 209.01260 |  | 1024010197 | 724030460.7 | 858683778.7 | 1060912768 | 495302740.5 | 791991047 | 520096177.2 | 482082451.8 | 743300349 | 712765989.8 | 857812488.7 | 723371086 |
|  |  | 1.17755169 | 4.956435124 | 0.018297168 | 287.22184 |  | 6588122.253 | 13924339.95 | 12238719.74 | 10073577.98 | 72894947.15 | 61986795.83 | 62868009.65 | 14508391.61 | 24308659.72 | 41427240.38 | 29907835.07 | 48063802.76 |
|  |  | 2.087946286 | 0.356020229 | 0.018529787 | 444.20794 |  | 290443246.9 | 256436961.7 | 152770945.4 | 382775488.3 | 34945002.63 | 157593394.4 | 74750782.12 | 118076601.7 | 30832782.89 | 182799782 | 210489826.9 | 247128944.9 |
|  |  | 1.129565851 | 0.604656785 | 0.018699534 | 252.01849 |  | 175093979.5 | 137290186.7 | 107641457.8 | 135687027.1 | 79865685 | 113044466 | 77634170.68 | 65471102.95 | 135702915.4 | 120160379.1 | 101519267.4 | 122122632.8 |
|  |  | 1.212170665 | 0.575525519 | 0.018781191 | 206.13875 |  | 218639389.5 | 120307000.9 | 159012053.1 | 155631569 | 85774180.01 | 82072980.04 | 115564396.3 | 92746174.74 | 181795744.7 | 243761121.5 | 259464848.4 | 215110946.3 |
|  |  | 6.968436642 | 0.60459926 | 0.018782979 | 225.00747 |  | 6169703358 | 3851492664 | 5083866560 | 6490544922 | 2967854383 | 4141427618 | 3147808553 | 2799597771 | 4294268818 | 3923947752 | 4955769622 | 4515192060 |
|  |  | 2.60443841 | 2.440345448 | 0.018785045 | 751.58799 |  | 176056865.9 | 103958680.5 | 174085501.7 | 135383791 | 312490390.2 | 539213245.8 | 349350771.4 | 237492236.5 | 307899091.4 | 208966515.9 | 85615547.29 | 51241662.99 |
|  |  | 1.070078493 | 0.605160836 | 0.01898343 | 402.34112 |  | 156286490.6 | 149213890.1 | 99389806.53 | 111811346.4 | 63604146.3 | 98021623.19 | 83718034.74 | 67343727.96 | 81756226.44 | 106265903.2 | 79317629.28 | 111001793.8 |
|  |  | 2.383646116 | 0.58283274 | 0.019119885 | 139.05033 |  | 842842139.7 | 560084332.6 | 475088054.8 | 748200663.8 | 424997085 | 363323972.4 | 345153884.5 | 397169254.8 | 577727176 | 580226262.1 | 369604544.4 | 672087266.8 |
|  |  | 2.539022014 | 0.412734123 | 0.019603417 | 295.22799 |  | 547393354.4 | 233343087.5 | 642882210 | 551343123.3 | 206754541.1 | 216301468.4 | 244009042 | 148069065.6 | 269117810.4 | 259368606.5 | 198125353.1 | 216231395.8 |
|  |  | 1.806187746 | 0.334647462 | 0.019889996 | 317.10649 |  | 142169394.3 | 250230294.5 | 274137515.6 | 110470675.8 | 70421358.07 | 82963432.22 | 66889085.95 | 39749839.22 | 52906687.87 | 83959367.07 | 142300170.2 | 78640510.85 |
|  |  | 5.185343311 | 0.527435571 | 0.019993207 | 209.01261 |  | 2668275255 | 2217564697 | 2869310582 | 1706018304 | 1320904858 | 1901322370 | 979540480 | 788389281.8 | 1225313692 | 2342622640 | 2568334115 | 1203278728 |
|  |  | 5.118039522 | 0.350480333 | 0.02000575 | 417.19533 |  | 1558305623 | 840829318 | 2355657055 | 1807308322 | 343082913.9 | 915981825.2 | 424960752.5 | 615861614.7 | 793169823.2 | 816232716.2 | 2188488905 | 1728010441 |
|  |  | 1.180180728 | 0.411975457 | 0.020069002 | 211.04162 |  | 120397393.2 | 52236281.08 | 79831089.73 | 114409852.3 | 29661525.17 | 49135930.05 | 23869766.47 | 48476116.01 | 63258066.51 | 49628550.56 | 56909998.28 | 68396417.99 |
|  |  | 1.023159872 | 0.484184758 | 0.020087185 | 267.01810 |  | 90323719.28 | 72207057.28 | 76676619.18 | 133620876.8 | 49917336.64 | 60285989.59 | 37057465.42 | 33256975.34 | 65087316.16 | 62551737.24 | 98804258.31 | 63998399.39 |
|  |  | 1.795731543 | 0.561940231 | 0.020115911 | 282.02902 |  | 235690241.3 | 379417747.9 | 344583121.2 | 241443385 | 131215761.3 | 219267974.9 | 137949164.9 | 186532895.3 | 150034446 | 122679805.6 | 292005241.7 | 211023695.3 |
|  |  | 1.313005565 | 0.388840479 | 0.020163407 | 401.18500 |  | 177302192.3 | 91895465.38 | 80288427.13 | 105110186.1 | 42077569.26 | 55454656.96 | 40542576.01 | 38690629.36 | 111352191.4 | 47046145.09 | 63503789.14 | 108609889 |
|  |  | 1.438247552 | 0.659559119 | 0.020190433 | 175.10781 |  | 261085587.2 | 192300399.3 | 237978360.6 | 273907742.5 | 123407783.3 | 199857060.3 | 129989366.2 | 183399799 | 146303583 | 125158937.9 | 93864143.64 | 161334087.1 |
|  |  | 1.617829505 | 0.657954828 | 0.020198713 | 225.00749 |  | 392658162.6 | 296038066.7 | 422292196.3 | 373520021.2 | 276560587.5 | 312202796.2 | 204873321.4 | 183102794 | 136614842.3 | 425901159.6 | 723666120.8 | 376237794 |
|  |  | 1.245109176 | 0.536638814 | 0.020240235 | 379.12224 |  | 112906784.9 | 155223003.1 | 154603434.7 | 148651317.2 | 86043722.57 | 106251960.6 | 91877001.07 | 22454437.41 | 123068306.9 | 202985552.5 | 98491759.58 | 185779357.7 |
|  |  | 1.044181658 | 3.43779123 | 0.020326478 | 553.28501 |  | 10730332.96 | 28385743.28 | 14065178.47 | 11172877.36 | 84320035.26 | 59113189.45 | 27036951.4 | 50765894.76 | 6163046.122 | 11523514.89 | 7581064.136 | 4102592.523 |
|  |  | 1.814175055 | 0.294342675 | 0.020351658 | 383.15326 |  | 243643034.5 | 173922570.9 | 84033782.64 | 280539554.2 | 40053233.55 | 81401306.24 | 53796899.82 | 54965428.91 | 25989342.61 | 113257500 | 248344483.1 | 97177360.2 |
|  |  | 1.668194547 | 0.497806549 | 0.020630698 | 200.12823 |  | 265054926.8 | 125843209.2 | 261700393.3 | 253734149.4 | 84072470.34 | 151432414 | 110405492.9 | 105267965.6 | 211126165.5 | 197268023.5 | 256463603.2 | 252005994.2 |
|  |  | 1.331410247 | 0.461067539 | 0.020813231 | 391.17945 |  | 164976411.3 | 106451596 | 154757110.1 | 103881552.2 | 38081740.01 | 97027030.49 | 80826071.39 | 28461693.05 | 104751148.4 | 90271182.58 | 145998990.4 | 122524433.3 |
|  |  | 1.022593201 | 0.587230759 | 0.020932893 | 335.22302 |  | 85614530.26 | 141074422.5 | 82407891.24 | 121078115.5 | 64458694.38 | 64043227.97 | 58541860.54 | 65568184.99 | 59270353.37 | 83692801.11 | 63349515.84 | 53732930.04 |
|  |  | 1.703172433 | 0.596031071 | 0.020974978 | 393.19536 |  | 392655337.8 | 294019175.1 | 238426509.7 | 267354951.2 | 139624190.8 | 169802262.1 | 168885841.2 | 232428516.9 | 163309142.3 | 230125645.9 | 237577767.1 | 354090493.7 |
|  |  | 1.086676735 | 0.385021868 | 0.020976318 | 320.08937 |  | 81519639.94 | 55541990.5 | 64843034.06 | 107326183.4 | 19749471.9 | 60394766.42 | 22106762.82 | 16809637.48 | 43469078.67 | 44323026.92 | 70652214.63 | 64169342.1 |
|  |  | 2.090096807 | 0.63628257 | 0.021025163 | 291.10197 |  | 590579704.9 | 392633493.1 | 530902557.9 | 581126070 | 310368968.7 | 451322114.9 | 310688678.4 | 260786092.3 | 295628365.6 | 415714221 | 415431205.9 | 357954318.4 |
|  |  | 1.236815875 | 0.585618788 | 0.021302944 | 267.16029 |  | 200842395.9 | 156217809.7 | 131848027.8 | 128543732.8 | 85142564.56 | 126378091.8 | 75445003 | 74625812.59 | 124950598.2 | 95690699.49 | 167415032.1 | 154052642.8 |
|  |  | 1.135473622 | 0.40236623 | 0.021469477 | 359.19480 |  | 108831135.3 | 51708712.13 | 79588851.81 | 89065960.15 | 28909929.96 | 63882950.48 | 24081473.39 | 15582460.23 | 29969356.64 | 38995540.13 | 43648776.17 | 60890555.62 |
|  |  | 1.069067845 | 0.38059037 | 0.021521137 | 357.17875 |  | 124581805.4 | 70923037.68 | 64916814.54 | 85964011.25 | 64574881.29 | 31904770.28 | 17150942.45 | 18200455.79 | 74735136.54 | 28323299.12 | 77241332.74 | 61806495.37 |
|  |  | 5.406353836 | 3.13497403 | 0.021798925 | 467.27789 |  | 333158626.2 | 570152406.6 | 290221157.4 | 623183560.1 | 2257710062 | 1492586780 | 1005636310 | 939423544.4 | 1599146832 | 1261382330 | 580317490.1 | 1357219095 |
|  |  | 1.690992645 | 0.288721089 | 0.02185708 | 361.12570 |  | 261476098.2 | 161007467.5 | 135926427 | 84827196.32 | 44090587.87 | 54732987.16 | 45184332.64 | 41708234.15 | 108409245.3 | 64283050.26 | 153216123.5 | 229076448.7 |
|  |  | 1.018251754 | 0.396994717 | 0.021936601 | 377.08563 |  | 51477013.23 | 78319711.41 | 109472330.2 | 49276985.74 | 25569898.68 | 28392931.78 | 32772092.9 | 27816330.42 | 29382428.49 | 27797297.87 | 31718236.02 | 34194019.1 |
|  |  | 1.492143599 | 0.470752392 | 0.022030853 | 202.11859 |  | 256672814.9 | 203352892.3 | 201047138.4 | 170683297.1 | 188432021 | 87697275.49 | 50998381.7 | 64423515.24 | 78953117.5 | 222578210.4 | 219658864.1 | 194485725.3 |
|  |  | 1.103339703 | 0.534804147 | 0.022211341 | 436.28287 |  | 133419116.7 | 108701654.9 | 95210265.94 | 88237849.78 | 36963614.01 | 89733621.6 | 64398639.55 | 36500130.61 | 149813750.3 | 51957498.76 | 85658239.35 | 101015955.9 |
|  |  | 1.202804866 | 0.531936331 | 0.022334068 | 125.09720 |  | 146357365 | 73843846.03 | 138649589.5 | 155456127.5 | 52553571.07 | 78290932.35 | 62680529.18 | 80053507.55 | 69359310.84 | 63476160.17 | 99201539.26 | 92619301.14 |
|  |  | 1.221944674 | 0.398765078 | 0.02243653 | 279.16026 |  | 156568998.2 | 55605595.76 | 108227027.7 | 107663344.8 | 50678762.04 | 43803548.07 | 45972962.75 | 30242087 | 46560897.18 | 68302571.93 | 85600860.93 | 81531779 |
|  |  | 1.028180617 | 0.397117838 | 0.022521087 | 264.13552 |  | 87974608.97 | 41924263.41 | 62350320.52 | 109818898.2 | 26560241.84 | 34515919.59 | 27452359.89 | 31428105.8 | 36251091.67 | 41947157.09 | 62492638.7 | 56505361.07 |
|  |  | 1.640220132 | 5.765080171 | 0.022572735 | 283.09260 |  | 15546130.77 | 30227281.59 | 22607967.59 | 14866822.24 | 66670084.31 | 143734448.3 | 200741241 | 68786786.1 | 18526278.93 | 20668591.05 | 18125104.92 | 22892989.69 |
|  |  | 1.174398112 | 0.639794784 | 0.022692863 | 221.01267 |  | 162998640.6 | 202490316.4 | 144003794 | 208581143.4 | 111756655 | 152172475 | 113897364.7 | 81593437.29 | 161241451.8 | 110982731.1 | 157293753.1 | 108379059.8 |
|  |  | 1.066303409 | 0.364183381 | 0.022697808 | 375.12707 |  | 28045917.61 | 88199236.34 | 85803767.31 | 87437978.04 | 21352266.46 | 31671715.07 | 31982744.71 | 20419591.46 | 75038341.81 | 87262102.61 | 101330565.9 | 51777125.69 |
|  |  | 4.718016848 | 0.337021691 | 0.022883054 | 363.22090 |  | 898497892.6 | 1944045714 | 1390124537 | 993612983.5 | 357881728.4 | 890909476.5 | 357981721.1 | 154597179.2 | 155745286.8 | 67640734.31 | 25005309.4 | 250375942 |
|  |  | 2.212206474 | 0.444085072 | 0.023030929 | 343.19779 |  | 451404343.1 | 208925069.8 | 298026640.8 | 376670774 | 104480107.6 | 241828739.7 | 108001416 | 138555221.3 | 125924410.3 | 100928576.3 | 141889212.4 | 172256284.9 |
|  |  | 3.150280486 | 3.492895557 | 0.023153465 | 497.29443 |  | 52173405.99 | 151187430.5 | 83517590.09 | 204057465.7 | 640573721 | 425071001.6 | 469451456.9 | 179691617.4 | 98614481.25 | 269065962 | 79954733.13 | 139662403.3 |
|  |  | 1.028054733 | 2.090317228 | 0.023204465 | 378.29992 |  | 42910525.25 | 41043623.81 | 33050994.47 | 27120588.14 | 45402613.02 | 77867542.82 | 106109630.7 | 71888713.44 | 35849713.65 | 37178287.22 | 39402506.41 | 31063362.65 |
|  |  | 1.683594521 | 0.56631544 | 0.02326396 | 243.01813 |  | 344111149 | 185119527.6 | 292507417.9 | 244323539.5 | 140147695 | 201583110.8 | 120155661.2 | 141840696 | 165945198.5 | 178210142.4 | 216996696.6 | 167917258.7 |
|  |  | 2.931530736 | 0.408846046 | 0.023446452 | 162.11250 |  | 717470520.3 | 388135746.1 | 482968256 | 469846867.3 | 54267773.01 | 220829327.8 | 167816971.5 | 398663374.8 | 373321321.6 | 153086860.6 | 505091385.5 | 202448674.9 |
|  |  | 1.663516416 | 0.34590794 | 0.023572588 | 307.08589 |  | 228510575.2 | 82900905.3 | 150871066.5 | 179655208 | 42509090.63 | 106171602.5 | 37889119.02 | 35481554.16 | 48207051.2 | 68062088.36 | 62643991.03 | 71590108.26 |
|  |  | 1.451695623 | 0.432071559 | 0.023587777 | 411.22023 |  | 146101963.6 | 188445516.7 | 158559660.8 | 218359268.8 | 52932049.57 | 78045604.7 | 157195348.5 | 19231398.46 | 79193759.57 | 112625444 | 91817228.19 | 94667142.19 |
|  |  | 2.320308678 | 1.868117498 | 0.023596876 | 279.23205 |  | 178676870.7 | 327521431.9 | 203059310.1 | 106376948.9 | 395201292.2 | 472430073.9 | 358097190.8 | 297972639.7 | 187240326.7 | 502283166.9 | 400030093.5 | 330227355.8 |
|  |  | 3.162041323 | 2.288010757 | 0.02360209 | 373.27473 |  | 338215842.8 | 207755898.8 | 250518746.9 | 272198913.5 | 561152567.3 | 484333449.2 | 937870365 | 461816465.9 | 490457668.4 | 607412445.1 | 176235732.5 | 304505432.1 |
|  |  | 5.000828872 | 0.554012631 | 0.023873484 | 165.05097 |  | 3537837119 | 2223536628 | 3006540456 | 1839014908 | 1743063486 | 1431312286 | 1343080130 | 1358916798 | 2406716110 | 2211289746 | 3237748326 | 1041397221 |
|  |  | 1.262607916 | 0.455515867 | 0.023905013 | 286.17617 |  | 167757640.4 | 70461449.61 | 136638495 | 131927881 | 70731699.71 | 55167657.5 | 28751994.97 | 76197468.97 | 77559710.7 | 47409800.37 | 35067060.21 | 88031123.89 |
|  |  | 2.191521859 | 0.637971106 | 0.024214586 | 393.19521 |  | 652923189.6 | 505229182.6 | 642081371.4 | 728987396.7 | 264137191.6 | 516617774.7 | 492600432.2 | 340214609.3 | 409711769.4 | 443385856.2 | 285772326.7 | 361305802.4 |
|  |  | 3.640920066 | 1.802184052 | 0.024229594 | 429.25843 |  | 543122524.2 | 643396976.6 | 511829487.9 | 505053611.1 | 763882826.1 | 1200485610 | 726168932.7 | 1280399657 | 649534722.2 | 643693005 | 409862951.1 | 360404747.2 |
|  |  | 1.07655443 | 15.77972189 | 0.024350988 | 562.45824 |  | 1805741.13 | 2957331.696 | 1373498.293 | 3933404.924 | 47024567.86 | 67998451.87 | 35543063.97 | 8335337.681 | 7379596.572 | 20605181.47 | 5786392.918 | 11804548.42 |
|  |  | 1.111818151 | 0.633532049 | 0.024361446 | 315.09086 |  | 177830442.9 | 136432453.5 | 167217976.2 | 188745151.1 | 130451399.1 | 131678535 | 104248345.1 | 58231386.94 | 139097164.3 | 138385060.1 | 161218869 | 129230728.8 |
|  |  | 1.058293578 | 0.613795782 | 0.024371292 | 411.14811 |  | 125583309.3 | 146709516.8 | 178405660.2 | 132267160.2 | 70935973.47 | 102512485.1 | 124380619.5 | 59992776.99 | 227287455.5 | 178653214.1 | 104964558.4 | 117678731.8 |
|  |  | 2.575712997 | 0.517525324 | 0.024589481 | 695.30823 |  | 471054684.5 | 790868292.8 | 465940737.2 | 695686427.5 | 258840648.8 | 218406640 | 466061406.2 | 310939878 | 393582285.8 | 270602588.7 | 540399260.1 | 606095981.5 |
|  |  | 1.990754879 | 2.253539271 | 0.024622967 | 421.30668 |  | 83575140.08 | 121173187.7 | 47775595.84 | 171211301.9 | 314042205.1 | 284047560.3 | 163532843.4 | 193281362.2 | 180649184.6 | 193886076.8 | 94441814.3 | 156364076.4 |
|  |  | 1.147380758 | 4.234378378 | 0.024771941 | 209.13962 |  | 6461099.108 | 23291013.87 | 12258853.17 | 19627842.18 | 72121318.04 | 103225359 | 24098954.42 | 61556405.76 | 15005978.49 | 20947327.8 | 19516550.26 | 21178687.94 |
|  |  | 3.908167257 | 19.21197398 | 0.025196281 | 212.12818 |  | 37603637.06 | 19478630.62 | 31281442.76 | 28089050.09 | 806182141.6 | 28131751.45 | 683547748.6 | 719425763.5 | 23594567.69 | 29741414 | 24730783.81 | 30214891 |
|  |  | 1.005891497 | 0.597483056 | 0.02519941 | 104.07051 |  | 121356288.7 | 78567474.88 | 141679413 | 117718325.3 | 59349782.98 | 55278535.47 | 67342435.63 | 92466060.38 | 116940192 | 111853120.9 | 135716332.3 | 145189666 |
|  |  | 1.15822745 | 0.62305343 | 0.025215692 | 193.01773 |  | 186500611.7 | 115299699.9 | 145709277.9 | 186895859.5 | 96202131.69 | 128370919.9 | 81803882.46 | 88891556.87 | 296387298.3 | 182340658.8 | 169406685.7 | 298775461.6 |
|  |  | 1.617806083 | 0.419121231 | 0.025306885 | 388.25447 |  | 138261572.1 | 234687012.2 | 254145564.3 | 107097734.6 | 71127102.96 | 74073231.8 | 80045563.48 | 82469507.81 | 1209686123 | 384252926.7 | 95867610.42 | 120616015.9 |
|  |  | 1.316115798 | 0.628340451 | 0.025359983 | 443.17455 |  | 247692805.1 | 158154086.1 | 203101670.9 | 242231351.4 | 105114418 | 170321555.3 | 154456168.5 | 104938628.8 | 121380431 | 64713585.46 | 78396021.71 | 98519420.95 |
|  |  | 2.866110561 | 2.598616118 | 0.025448607 | 481.26263 |  | 69044836.44 | 236616826.5 | 329757633.9 | 103788933.9 | 453160980 | 711907108 | 356189516.7 | 399660818.1 | 158160464.6 | 135329578.4 | 340227426.8 | 84871537.36 |
|  |  | 2.94931463 | 0.425896775 | 0.025890632 | 132.10187 |  | 831472837.6 | 714045047.4 | 582159873.8 | 421475934 | 359269735 | 458694313.5 | 57124183.41 | 210588104.8 | 106111034 | 387594053.8 | 302901694.7 | 226196466.7 |
|  |  | 1.229147853 | 0.527689706 | 0.025938129 | 315.03929 |  | 149109193.3 | 93970976.43 | 125625646.1 | 173440942.3 | 51257941.23 | 109763528.5 | 72695201.32 | 52368592.2 | 98407376.21 | 88297411.92 | 128104601 | 127395845.9 |
|  |  | 1.234984411 | 0.370468575 | 0.026034013 | 99.04515 |  | 106020535.9 | 53172030.35 | 147122595.1 | 96918739.69 | 32119655.25 | 66251233.26 | 24666050.22 | 26348550.04 | 47094924.27 | 39940575.57 | 61531690.06 | 42294874.1 |
|  |  | 1.780905293 | 9.886042153 | 0.02609804 | 98.09650 |  | 8698090.987 | 12849769.54 | 13330538.71 | 9213958.109 | 148734716.7 | 131812239.9 | 145799395.8 | 9552551.022 | 13776704.52 | 11446240.36 | 11126318.98 | 7082276.913 |
|  |  | 1.23561737 | 1.83292884 | 0.026174875 | 419.29159 |  | 49284797.77 | 74560955.01 | 32777289.36 | 90311660.38 | 123838877.2 | 134905903.9 | 79623370.65 | 114245586.1 | 89099027.59 | 84698469.2 | 57379212.96 | 66826603.6 |
|  |  | 5.242372144 | 0.338380286 | 0.026183243 | 393.26372 |  | 1813524716 | 864683570.1 | 1691817686 | 2628913037 | 164951438 | 903101289.3 | 749644686.4 | 550605572.4 | 1283578815 | 933028374.8 | 1229000464 | 2027036499 |
|  |  | 1.716577278 | 8.011843231 | 0.026196514 | 417.33297 |  | 7913746.881 | 9179693.073 | 7772511.033 | 27515535.13 | 142310862.4 | 154718583.4 | 104944885.9 | 17697923.22 | 6494719.303 | 23113612.33 | 5597138.462 | 157147970 |
|  |  | 1.459431704 | 3.751576116 | 0.026216383 | 209.08113 |  | 17287739.04 | 48258135.04 | 27376191.92 | 11304213.55 | 122800815.5 | 30913014.29 | 104198695.3 | 133100295.9 | 93081139.11 | 88637164.08 | 21726258.45 | 22817201.91 |
|  |  | 1.102281272 | 6.55976829 | 0.026330482 | 487.23719 |  | 1020279.414 | 16977810.09 | 667319.9037 | 9991201.053 | 56894683.26 | 76613157.13 | 39052858.15 | 15420026.07 | 20484233.18 | 34727106.46 | 28413060.52 | 25211093.71 |
|  |  | 1.488716498 | 2.76738482 | 0.026736125 | 285.20728 |  | 24607069.37 | 53034363.55 | 26371328.53 | 47754733.7 | 141457623.7 | 115723002.6 | 121088244.6 | 41730191.34 | 53321509.04 | 143880909.5 | 96335392.25 | 91565337.72 |
|  |  | 1.951845448 | 0.105627702 | 0.026858679 | 495.18327 |  | 154297946.8 | 236175766.4 | 35349745.71 | 276378646.5 | 6902839.608 | 10295735.63 | 12692534.87 | 44280884.64 | 27288684.79 | 32091080.4 | 37983531.77 | 29308248.73 |
|  |  | 1.367047627 | 0.360226902 | 0.026968699 | 280.19087 |  | 76858620.18 | 125507004.4 | 129631752.5 | 85844029.47 | 31887707.59 | 11537145.46 | 15321376.98 | 91771485.53 | 56116759.04 | 6286989.864 | 88935965.95 | 8175063.396 |
|  |  | 1.527912065 | 0.431330724 | 0.027321461 | 353.20721 |  | 224062568.8 | 102930715.1 | 197913195.2 | 205578098.3 | 54221108.23 | 106130177.5 | 128359673.5 | 26369482.47 | 198020262.2 | 60990145.33 | 94340772.4 | 240735690.4 |
|  |  | 1.332852945 | 0.602363742 | 0.027350354 | 267.16031 |  | 223117071.9 | 129233903.8 | 178134459.9 | 224964286.7 | 99108944.78 | 148198832 | 91500419.11 | 116247326.1 | 148974352.9 | 130168201.7 | 165582415.2 | 161879511.2 |
|  |  | 1.853846838 | 0.511916248 | 0.027354686 | 371.15344 |  | 295008536.5 | 313650591.9 | 359039474.1 | 216053633.7 | 176595691.5 | 234142565.7 | 151650633.7 | 43593112.64 | 372433714.8 | 294911685 | 310432312.2 | 242160282.1 |
|  |  | 1.286261016 | 5.844404446 | 0.02743273 | 765.56706 |  | 12978284.81 | 23018054.61 | 6813845.343 | 5903492.114 | 51443711.31 | 130182342.4 | 60117654.62 | 42958721.3 | 50664041.28 | 38554903.95 | 10276715.45 | 20975672.99 |
|  |  | 3.016442616 | 0.451510709 | 0.027502719 | 237.08041 |  | 785222624.2 | 663855984 | 691986800.9 | 727725964 | 491208056.3 | 99801240.29 | 93788184.75 | 610492544.2 | 509233513.8 | 686465094.4 | 610985951.6 | 549608688.1 |
|  |  | 1.245390614 | 0.656565401 | 0.027593508 | 229.01778 |  | 17052169.06 | 13098813.86 | 14753885.36 | 12547703.88 | 8891991.967 | 12778392.7 | 9918740.628 | 6132245.799 | 10735278.22 | 12738654.02 | 17915631.91 | 4606168.797 |
|  |  | 1.610427129 | 3.377768532 | 0.0276063 | 439.18697 |  | 58472996.66 | 52758902.19 | 25661838.54 | 13036805.35 | 176516025 | 48386119.24 | 162809451.2 | 118719073.8 | 9041903.53 | 21458813.54 | 106709742.8 | 31309031.12 |
|  |  | 1.42791596 | 0.413761695 | 0.027739574 | 397.30638 |  | 200388706.5 | 169603929.4 | 97698481.76 | 96810564.45 | 31672615.92 | 87350964.47 | 49687931.36 | 64857661.12 | 45734755.27 | 77085140.04 | 83044072.78 | 57599606.06 |
|  |  | 2.905308461 | 0.254417723 | 0.027905358 | 117.05575 |  | 176285107.1 | 486357216.8 | 642749002 | 423010494.9 | 131141292.4 | 18455877.7 | 31933262.25 | 258205623.7 | 26982352.98 | 70459418.02 | 442834050.2 | 61385515 |
|  |  | 2.133269561 | 1.633977872 | 0.027924214 | 116.02554 |  | 130635309.3 | 251485563.4 | 256789680.7 | 253442280.8 | 397744578.1 | 303273240.6 | 300405936.1 | 456661030.1 | 378567649.4 | 331531451.9 | 388578380.4 | 255849640.3 |
|  |  | 2.323527547 | 0.454107303 | 0.028050895 | 176.98631 |  | 615964880.1 | 454326359.6 | 303110635.4 | 287874644.1 | 145693797.5 | 230153731.7 | 179928405.7 | 198621865.3 | 285341484.2 | 428953010.3 | 357569211.9 | 421524249.3 |
|  |  | 1.41291873 | 0.612072187 | 0.028113193 | 369.13730 |  | 183380764.2 | 186793179.2 | 238285427.6 | 237835418 | 76640895.8 | 177957679.3 | 101763198.8 | 161631728.5 | 37070740.98 | 263345967.7 | 245496550.1 | 181116556.7 |
|  |  | 2.852177123 | 0.370776663 | 0.028347353 | 280.62603 |  | 396522679.2 | 774777699.7 | 574865091.9 | 362441865.4 | 207200950 | 160172344.7 | 366283025.7 | 48166072.24 | 119820680.8 | 121584288.3 | 294615716.6 | 148744943.1 |
|  |  | 4.936212516 | 0.665702686 | 0.028426732 | 179.00194 |  | 3522929122 | 2726004365 | 2701715202 | 3179072830 | 1701590190 | 2830904505 | 1498221783 | 2044071720 | 1869627430 | 2920168375 | 3278628265 | 2778542545 |
|  |  | 1.131656778 | 0.648795849 | 0.028442305 | 80.96508 |  | 183518512.9 | 112134883.5 | 149083754.6 | 182619766.9 | 78810401.57 | 121940710.1 | 98202382.97 | 108073069.6 | 93790938.61 | 139819503.7 | 149451634.3 | 140317289.2 |
|  |  | 1.239023393 | 0.550884364 | 0.028601173 | 232.10046 |  | 156855605.9 | 87425999.01 | 146837323 | 139161651.3 | 43506643.48 | 109604071.9 | 63490022.12 | 75522542.32 | 140186880.2 | 120661873.2 | 152331566.2 | 130459867.8 |
|  |  | 1.052947926 | 0.429517465 | 0.0286881 | 379.15951 |  | 101872794.4 | 89150432.66 | 69820475.15 | 70629433.37 | 18800962.13 | 60296900.41 | 60165400.95 | 3110237.371 | 22701720.51 | 7294243.397 | 8785143.963 | 89647723.66 |
|  |  | 1.0941895 | 0.5200603 | 0.0287063 | 445.29496 |  | 131203959.5 | 70790483.48 | 97992971.69 | 134318930.2 | 59639344.81 | 84165057.37 | 43085422.02 | 38975663.66 | 65961063.42 | 83519813.27 | 64401901.83 | 65614662.29 |
|  |  | 1.046937948 | 0.498774327 | 0.028726834 | 311.16130 |  | 100655739.8 | 48231724.16 | 116630467.3 | 107019313.2 | 31719730.84 | 54238293.59 | 43884914.36 | 55969074.76 | 35118119.88 | 54367294.56 | 52914377.62 | 40685505.14 |
|  |  | 1.607345928 | 0.448913834 | 0.028778922 | 359.11669 |  | 210113835.9 | 296327151.2 | 162471586.7 | 152309799.3 | 82531543.74 | 88782393.69 | 151735911.2 | 45608235.21 | 94171222.88 | 167780402.3 | 169093105 | 212528843.5 |
|  |  | 1.248162548 | 0.517662683 | 0.029213844 | 211.13404 |  | 162036256.1 | 82428407.55 | 125699790.2 | 164908785.5 | 58960308.73 | 104740073.7 | 55928863.74 | 57358202.54 | 33593430.4 | 30489068.07 | 28297760.48 | 49332830.47 |
|  |  | 1.017486891 | 0.437990141 | 0.029304465 | 150.09148 |  | 95906845.95 | 46811050.54 | 73152759 | 60095500.4 | 17644524.23 | 23683880.36 | 23493035.46 | 56049015.44 | 60854770.61 | 49355173.93 | 38952316.32 | 30975439.23 |
|  |  | 1.083454229 | 0.601841428 | 0.029374826 | 213.04040 |  | 130936051.2 | 98560174.01 | 120163343.8 | 142494953.6 | 57671697.33 | 117383731.3 | 60146677.29 | 60996874.53 | 114583299.7 | 131332857.6 | 152367731.2 | 151287628.9 |
|  |  | 1.102477045 | 0.388005425 | 0.029588775 | 211.01470 |  | 106230987.5 | 44845200.28 | 63749282.58 | 115585450.5 | 28011785.04 | 35509853.05 | 19149543.63 | 45530048.02 | 52224048.36 | 42187740.51 | 53351369.44 | 56114266.5 |
|  |  | 1.46892679 | 1.778483257 | 0.029627475 | 187.10778 |  | 107421339.2 | 82870168.04 | 73201285.81 | 88065921.53 | 152115792.8 | 222243281.7 | 119133009 | 131749204.2 | 73867132.51 | 68765195.29 | 80699912.11 | 83387261.42 |
|  |  | 1.987394863 | 0.436343066 | 0.029739582 | 210.99182 |  | 417103756.8 | 295155341.9 | 200731667.7 | 234328661.2 | 91953648.78 | 209747186.7 | 132794365.4 | 66129676.28 | 193522865.3 | 120202239.7 | 69942416.73 | 220055663.3 |
|  |  | 1.049389826 | 0.639117072 | 0.029843835 | 237.04395 |  | 187189837.8 | 121156054.3 | 123477686.8 | 129795171.5 | 92343435.98 | 111159918.7 | 85652273.48 | 69784503.34 | 149283502.9 | 105184778.1 | 199632218.7 | 155797169.5 |
|  |  | 1.052125376 | 0.520402738 | 0.030072118 | 291.19572 |  | 114740972.8 | 65118166.58 | 102885792.8 | 77750237.48 | 34690393.95 | 77521590.62 | 38567099.09 | 36823589.75 | 95063279.69 | 103304832.2 | 100362525.3 | 101982444.3 |
|  |  | 8.159587687 | 0.638561823 | 0.030198274 | 249.06167 |  | 9197169371 | 7294737841 | 6638017864 | 10192089244 | 4804967970 | 7080735911 | 3876239953 | 5516222365 | 7711708994 | 8405468258 | 9247224944 | 8076446456 |
|  |  | 1.199704089 | 0.494495857 | 0.030257916 | 301.20221 |  | 136383834.4 | 72091949.36 | 92218793.62 | 124603580.4 | 30903702.94 | 72945347.06 | 32520985.4 | 73938141.69 | 98848366.73 | 108850480.2 | 108107540.6 | 82075954.26 |
|  |  | 1.769432971 | 3.351769438 | 0.030284362 | 390.16723 |  | 41467332.4 | 38403257.96 | 44043104.47 | 38835186.78 | 164063387.5 | 192440916.4 | 150891447.3 | 38100976.26 | 26372794.15 | 31955512.7 | 20679414.34 | 26472654.53 |
|  |  | 2.038211399 | 0.388270784 | 0.03031144 | 335.18972 |  | 298420217.2 | 310789787 | 343466588.7 | 142746927.9 | 90707368.68 | 220828763.3 | 62900055.61 | 50884761.3 | 29727617.85 | 14956488.26 | 47996433.63 | 25325776.91 |
|  |  | 1.463024905 | 1.836790652 | 0.030550449 | 451.30616 |  | 112519408.9 | 66858987.06 | 43848923.65 | 115666523.1 | 101904644.5 | 173116748.9 | 178400056.9 | 169055592 | 133089462 | 97571136.08 | 93399888.95 | 69568154.39 |
|  |  | 1.319216802 | 3.687209984 | 0.03069955 | 102.06704 |  | 25159452.33 | 12733616.45 | 21241476.65 | 30645220.86 | 83024035.3 | 60460984.85 | 141799998.8 | 45751831.6 | 65356280.19 | 25455348.49 | 26039135.07 | 99844334.73 |
|  |  | 1.694664115 | 0.346898369 | 0.030711019 | 286.12876 |  | 240770356.9 | 61224875.66 | 209416429.9 | 192745444.6 | 49882694.27 | 84594333.08 | 38279546.94 | 71514377.41 | 80862523.7 | 80267721.72 | 114111193 | 77904440.82 |
|  |  | 1.840396783 | 0.472578935 | 0.030825033 | 239.02321 |  | 303050064.7 | 147117241.4 | 304530467.3 | 393485871 | 118169193.8 | 186115163.4 | 117240019.2 | 121083027.1 | 363263614.9 | 168353568.6 | 134731891.2 | 136793208.5 |
|  |  | 3.289628273 | 0.566064068 | 0.030847942 | 160.04041 |  | 1478537914 | 721179900.2 | 997301721.9 | 1244182113 | 510613503.9 | 571452536.1 | 676055532.2 | 755883100.6 | 1056582992 | 690204567.4 | 681787018.4 | 1078051994 |
|  |  | 1.641376105 | 3.018783816 | 0.031209671 | 251.20071 |  | 38522568.19 | 60530122.17 | 34447204.53 | 29809250.46 | 150798929.4 | 155959887.6 | 148821832.5 | 37414355.53 | 38031922.11 | 162421254 | 120566876.8 | 115950115.1 |
|  |  | 2.034796511 | 4.690076058 | 0.031300226 | 99.04515 |  | 48547862.82 | 21377400.23 | 31430889.39 | 37591077.31 | 196210389.9 | 200806490.1 | 226682978.6 | 27973217.02 | 22846298.66 | 177936802.1 | 33459493.27 | 36302572.29 |
|  |  | 1.050586428 | 2.715752562 | 0.031368804 | 491.27602 |  | 16336667.19 | 23479182.36 | 11281042 | 28637530.74 | 82043214.98 | 49855632.96 | 58816370.29 | 25823743.34 | 66380004.56 | 37351083.09 | 17758794.73 | 118523345.4 |
|  |  | 1.958528552 | 0.425405971 | 0.031401516 | 405.15872 |  | 264501103.9 | 202004439.9 | 378704420.4 | 468243941.5 | 182476368.9 | 189697354.6 | 137612077.5 | 48965333.68 | 175621008.9 | 284664043.2 | 208918051.1 | 216973088.5 |
|  |  | 1.21566181 | 0.560821826 | 0.03153297 | 145.09917 |  | 162844706.1 | 92572434.21 | 127461608.5 | 158480247.9 | 45021981.5 | 111806466.2 | 74751457.44 | 72026035.69 | 130877078.7 | 100494283.2 | 104211673.3 | 113648974.4 |
|  |  | 1.194791195 | 0.562703784 | 0.031566731 | 225.00750 |  | 144892137.3 | 87861599.32 | 147652548.8 | 119674926.1 | 44278966.85 | 95076981.92 | 48678223.64 | 93363417.62 | 103547534.8 | 68691756.23 | 170681678.1 | 125222211.9 |
|  |  | 1.76395716 | 0.095288555 | 0.031898122 | 196.99464 |  | 10689498.84 | 143872211.3 | 209635198.4 | 201755589.8 | 11514596.64 | 15593534.61 | 13992332.76 | 12828331.48 | 28535446.13 | 144663650.8 | 15287821.28 | 153975398.4 |
|  |  | 1.600305895 | 0.545768485 | 0.032062454 | 345.22830 |  | 305677572.3 | 179969745.9 | 176250694.7 | 297674480.1 | 96188527.91 | 174187831.5 | 122473130.6 | 130854936.1 | 157487881.2 | 148169997.3 | 158785805.7 | 131732406.2 |
|  |  | 4.726349636 | 0.56141821 | 0.032130193 | 80.96510 |  | 2429496929 | 1685929850 | 2151344937 | 3292605481 | 1194114638 | 1845045151 | 1198011559 | 1129637085 | 1570136107 | 1858935644 | 4219884230 | 2976984044 |
|  |  | 1.05726645 | 0.613543305 | 0.032210535 | 211.09767 |  | 174715936.8 | 95545851.57 | 110785371.4 | 147845874.3 | 76441539.12 | 93781292.98 | 76614758.43 | 77661189.52 | 98247039.73 | 91543690.71 | 124978858 | 107553877.7 |
|  |  | 1.149323781 | 6.843518138 | 0.032289278 | 413.23674 |  | 5342860.678 | 4735630.007 | 5595378.027 | 13188546.95 | 86363339.91 | 49817702.31 | 48810051.1 | 12529371.73 | 7617360.341 | 8819789.093 | 6305344.615 | 15974455.54 |
|  |  | 2.990142861 | 0.617555189 | 0.032408047 | 257.16090 |  | 1263337464 | 666434329.5 | 1129402388 | 961190463 | 501323505.5 | 753869543.8 | 604112482.7 | 623491515 | 1147439097 | 804217625.5 | 892436435.2 | 847421585.1 |
|  |  | 1.671367665 | 0.554538292 | 0.032439141 | 319.12213 |  | 250560777.4 | 331377330.6 | 264732748.7 | 215286256.1 | 179963988.7 | 220537500 | 131109835.8 | 57284559.4 | 180570303.2 | 253765831.5 | 314353416.9 | 201777581.7 |
|  |  | 1.025626889 | 0.436154081 | 0.032551141 | 245.16503 |  | 96492214.12 | 40106940.13 | 62823964.43 | 94539594.75 | 19410634.86 | 47690172.07 | 24153171.68 | 36959058.55 | 114954044.8 | 72732722.56 | 42089595.29 | 45381203.39 |
|  |  | 1.917896128 | 0.325075364 | 0.03256191 | 105.06985 |  | 372452242 | 247012157.4 | 166448750.8 | 135544423.2 | 85647844.93 | 107442160.3 | 86684388.48 | 19768762.59 | 145311706.2 | 71380631.6 | 533971233.6 | 108059502.5 |
|  |  | 2.775328965 | 0.590927933 | 0.032738006 | 409.18998 |  | 966758765 | 582772538.2 | 945741116.9 | 870578592.2 | 374523942.2 | 694100598.1 | 593195012.4 | 327155827.5 | 691033892.6 | 457238285.5 | 726749886.1 | 522928297.3 |
|  |  | 1.107133216 | 1.856686345 | 0.032859457 | 162.11258 |  | 66235351.73 | 51675875.66 | 46883862.29 | 23100113.24 | 78838776.07 | 121176699.9 | 78747553.91 | 70099427.73 | 13347129.46 | 9856617.114 | 161205003.6 | 151864154.9 |
|  |  | 1.060071811 | 0.391044928 | 0.032890493 | 425.10247 |  | 105678673.4 | 32975226.15 | 73550913.14 | 98324487.71 | 29017820.43 | 44537523.39 | 26365387.6 | 21510176.35 | 25673509.66 | 35933504.28 | 31136237.97 | 36303179.02 |
|  |  | 1.803759388 | 0.249080825 | 0.0330705 | 131.07141 |  | 217885052 | 52777769.94 | 275890438.4 | 165148801.2 | 20037877.28 | 62912362.85 | 35945054.84 | 58376041.99 | 4080275.302 | 150824907 | 1480245470 | 282264936.5 |
|  |  | 1.069155486 | 0.637972854 | 0.033122237 | 219.13412 |  | 164674823.1 | 95271238.5 | 119685704.3 | 148228283.7 | 66625067.54 | 91448317.73 | 75822663.46 | 102864333.7 | 110623651.8 | 86660760.89 | 81847405.47 | 130644377.1 |
|  |  | 1.806410519 | 0.420801065 | 0.033138767 | 339.26856 |  | 148564289.2 | 299152115.5 | 325250633.9 | 170205796.3 | 62385664.29 | 160864882.4 | 94859850.59 | 78777735.74 | 108159334 | 69995194.63 | 101589630.2 | 50272059.12 |
|  |  | 1.210078968 | 0.370629027 | 0.033259704 | 257.16611 |  | 168331859.2 | 89472107.83 | 114264119.1 | 65798582.26 | 43638403.27 | 24215688.2 | 73366462.31 | 21065543.42 | 45546183.09 | 41717640.36 | 103045717 | 50684736.5 |
|  |  | 1.435111152 | 0.466345455 | 0.033267738 | 367.20960 |  | 140356926.4 | 153692353.2 | 104415816.8 | 207811446.8 | 28944333.52 | 68016170.59 | 60289157.01 | 125484649.3 | 66144511.99 | 109461628.5 | 70162314.36 | 139777571.4 |
|  |  | 4.725470715 | 0.078267498 | 0.033341266 | 443.24707 |  | 1192494466 | 963906888.9 | 1726323176 | 117014462.2 | 95021890.67 | 102410777.8 | 87558044.34 | 28058852.38 | 207408916.9 | 144661031.4 | 75831982.16 | 91705670.93 |
|  |  | 1.123390286 | 0.489216666 | 0.033553631 | 202.07217 |  | 93673221.37 | 125620362 | 139820318.7 | 64848852.65 | 44950812.04 | 81558803.94 | 47749820.49 | 33150209.07 | 122009432.4 | 61562191.46 | 49956578.98 | 115561740.8 |
|  |  | 6.367101506 | 1.737952168 | 0.033643169 | 427.26190 |  | 1879820196 | 2325642297 | 1703649404 | 1685008911 | 2462627069 | 4049781985 | 2448679112 | 4237130556 | 2344667697 | 2268402748 | 1368085177 | 1195811445 |
|  |  | 1.047878708 | 0.488847698 | 0.033723868 | 341.17077 |  | 94907750 | 69875494.77 | 89435893.01 | 81352578.5 | 27064520.66 | 84206366.53 | 19474447.77 | 33298126.22 | 81266772.27 | 61522146.4 | 88077271.87 | 44183306.55 |
|  |  | 1.284051076 | 0.534794589 | 0.033823044 | 391.17967 |  | 191757939.3 | 105592543.7 | 120288811.4 | 161962132.3 | 51915477.94 | 117706205.2 | 81512803.05 | 58833220.7 | 78368916.78 | 56856888.33 | 63071003.84 | 74645977.74 |
|  |  | 2.718111578 | 13.22012128 | 0.033981271 | 182.97713 |  | 22051384.26 | 26784469.52 | 16787704.1 | 34012359.41 | 13491686.47 | 533253455.9 | 406918147.6 | 363535620.9 | 22119782.34 | 7518595.005 | 33638967.57 | 28508676.96 |
|  |  | 1.249643716 | 0.644512131 | 0.034075031 | 293.17596 |  | 178886675.3 | 238922429.1 | 190049361.2 | 241474965 | 103335411.2 | 194919467.3 | 148958826.2 | 100191994.5 | 92775273.51 | 167869864.3 | 129980088.1 | 71202298.87 |
|  |  | 1.032199748 | 0.396070818 | 0.034275127 | 323.18921 |  | 97753061.17 | 60814161.88 | 101797041.3 | 43069345.38 | 23115073.09 | 44507200.63 | 44238466.79 | 8320457.38 | 22772439.93 | 14720984.56 | 15797730.93 | 13847384.69 |
|  |  | 1.248529346 | 0.497770323 | 0.034521392 | 458.35458 |  | 191624104.4 | 70007448.49 | 153790720.1 | 155713645.3 | 60520059.78 | 66366815.01 | 85932989.89 | 71474645.94 | 93728350.55 | 93630964.58 | 105252119.2 | 103958204.4 |
|  |  | 2.700546829 | 0.589671434 | 0.034596243 | 173.04562 |  | 94374222.35 | 50287093.53 | 59774414.04 | 87516395.89 | 37255056.27 | 49755981.84 | 39712422.18 | 45432368.42 | 37758745.76 | 48197742.25 | 64413414.93 | 70662491.67 |
|  |  | 1.7266525 | 0.525536813 | 0.034678753 | 114.05609 |  | 367014771.3 | 184681490.3 | 200482816.6 | 305570943.2 | 121949382.4 | 179038664.4 | 108458213.3 | 146440314.9 | 213096285.7 | 181392384 | 234490797.9 | 213896775.5 |
|  |  | 1.599893367 | 0.503316975 | 0.034756624 | 403.14344 |  | 270223520.5 | 177464510.2 | 214628657 | 349294507.5 | 135761216.9 | 170892855.4 | 153992829.1 | 48514185.52 | 217011059.5 | 271761514.1 | 288549761 | 261700539.3 |
|  |  | 1.063940433 | 1.646938729 | 0.034897691 | 551.30479 |  | 44782388.3 | 82657813.04 | 54645711.86 | 52825323.19 | 99812096.15 | 127115056.7 | 75605827.92 | 84351432.3 | 49415510.79 | 77258738.84 | 36468468.66 | 52584287.83 |
|  |  | 2.15847883 | 0.544984118 | 0.034921545 | 379.29566 |  | 558875181.8 | 484670305.8 | 379103209.8 | 377271380.3 | 276781903.9 | 343794368.1 | 293649220.1 | 66702363.25 | 353572457.6 | 329831266.5 | 444299549.2 | 432256279.4 |
|  |  | 1.541522191 | 0.495734035 | 0.03494387 | 293.07004 |  | 173734356 | 162053701.7 | 327949961.4 | 291131066.2 | 134434322.5 | 149881785.1 | 108982186.8 | 80062809.89 | 144563308.7 | 148092783.3 | 167343476.9 | 143618471.3 |
|  |  | 1.741696323 | 0.223869727 | 0.035064719 | 564.35961 |  | 101563283.9 | 218659726.2 | 241506260.4 | 59644006.78 | 25635839.73 | 39401783.85 | 28743612.39 | 45325430.3 | 1275061393 | 383090791.6 | 76967447.91 | 109733564.1 |
|  |  | 1.661962706 | 0.480348644 | 0.035112432 | 373.25961 |  | 287184156.2 | 111299888.3 | 215803919.3 | 240505324.5 | 68190885.06 | 97653406.13 | 94517204.54 | 150237301 | 157206857.1 | 170464253.4 | 75544666.54 | 84284931.82 |
|  |  | 1.703171495 | 0.442822697 | 0.035327323 | 333.10143 |  | 332642098.3 | 161612068.8 | 287645499.7 | 171444434.7 | 104554661.7 | 118475655.9 | 158681712.2 | 40450375.96 | 147499984.5 | 98994673.05 | 255775810.8 | 219017829.3 |
|  |  | 2.032677253 | 0.25062154 | 0.035332079 | 160.13335 |  | 300430624.4 | 146584798 | 325377996.7 | 102196602.8 | 23833048.35 | 41756141.08 | 26341048.56 | 127260859.9 | 121833869.9 | 98763490.83 | 274541796.9 | 168699091.4 |
|  |  | 1.707473346 | 13.97086685 | 0.035343477 | 264.14975 |  | 7408591.109 | 9188103.966 | 6339082.332 | 14983984.46 | 2714095.056 | 187923435.3 | 201889623.3 | 137244790.4 | 4394744.433 | 6817350.857 | 6596378.472 | 333891305.5 |
|  |  | 1.16270345 | 0.659339443 | 0.035425565 | 202.10767 |  | 204167736.5 | 158399992.4 | 184679203.6 | 178298310.2 | 127121278.2 | 164661514.6 | 122725612.1 | 63872191.36 | 176972827.6 | 200982327 | 177535077.2 | 175661146.9 |
|  |  | 1.462769684 | 0.461575564 | 0.035706653 | 117.05566 |  | 224873361.2 | 86549149.65 | 142278934.5 | 193458018.1 | 64063204.99 | 94467358.28 | 48340703.4 | 91841727.35 | 121814183.4 | 91011518.19 | 106884302 | 132684386 |
|  |  | 1.367829714 | 0.645075728 | 0.035748218 | 312.19193 |  | 290043217.5 | 170967772.5 | 230408844.2 | 277073939.8 | 151333999.2 | 205513694.9 | 137509553.3 | 130394578.5 | 234521529.8 | 217625214 | 236924200.7 | 255726304.8 |
|  |  | 1.460055701 | 0.534358673 | 0.03583591 | 273.12354 |  | 227290687.5 | 112058449.9 | 229267102.6 | 167699183.3 | 63036817.68 | 120113845 | 83183793.93 | 127122075.9 | 111622975.7 | 112226257.7 | 129528161.9 | 102851345.8 |
|  |  | 1.252560149 | 0.359391575 | 0.036145067 | 292.10393 |  | 137254114.7 | 89454575.6 | 59890189.39 | 138012300 | 20319429.98 | 74606053.52 | 55837639.7 | 1838557.629 | 104999490 | 106776493.9 | 109268397.9 | 138864091.3 |
|  |  | 1.478697924 | 0.508883794 | 0.036357345 | 427.17956 |  | 293809278.4 | 179194909.7 | 135283940.6 | 224906349.9 | 109368064 | 134267255.2 | 124530759.6 | 55833088.81 | 138605339.1 | 225083760.2 | 196592939.9 | 383034873.9 |
|  |  | 1.144296366 | 0.553733086 | 0.036601183 | 377.20015 |  | 146003606.3 | 83428539.03 | 108762579.3 | 150009877.4 | 50583032.8 | 105405382.6 | 58065387.2 | 56281238.43 | 72729221.41 | 67231164.51 | 71837676.7 | 96478150.62 |
|  |  | 4.120518389 | 0.353789766 | 0.036753702 | 176.12795 |  | 1691124448 | 573874848.6 | 850361345 | 1007690680 | 148193762.1 | 483781148.2 | 418289118.5 | 408429334.7 | 343840004.1 | 688971884.3 | 1086665563 | 478216420 |
|  |  | 1.234012377 | 0.337925236 | 0.036889296 | 331.01304 |  | 129905380.5 | 33759520.13 | 142210265.7 | 94890299.35 | 20681677.89 | 33316922.48 | 44818165.26 | 36611999.09 | 131165299.4 | 88243165.41 | 38345618.05 | 31209485.48 |
|  |  | 1.923267478 | 0.394494483 | 0.037138304 | 275.12385 |  | 371722779.6 | 311506803.1 | 149018690.2 | 183312711.7 | 39278445.46 | 143606631.1 | 87426825.36 | 130321303.5 | 71241250.52 | 147839795.5 | 225474628 | 363146831.5 |
|  |  | 2.088365677 | 0.224144707 | 0.037168013 | 476.30688 |  | 147314854.2 | 307425949.1 | 370039129.9 | 87777674.97 | 44064831.09 | 44490138.2 | 59804758.15 | 56185230.2 | 1914439915 | 519339982.5 | 92367227.56 | 140687174.7 |
|  |  | 1.029689801 | 0.597697724 | 0.03722169 | 443.25452 |  | 150406029.3 | 112159591.7 | 138729050.2 | 77850411 | 57179593.21 | 92078286.73 | 77555619.11 | 59570426.22 | 32707985.81 | 89429175.51 | 66354378.76 | 108279447.1 |
|  |  | 1.56949419 | 0.406289808 | 0.037317736 | 165.05574 |  | 146288960.5 | 202590796.3 | 230965274.7 | 144384432.4 | 136483636 | 127876802.9 | 26055570.63 | 3831040.546 | 23850844.43 | 97425060.69 | 239378287.3 | 176492905.7 |
|  |  | 1.29339062 | 4.876831408 | 0.037751629 | 141.07642 |  | 13861014.13 | 15810974.99 | 16723968.63 | 16215109.85 | 44374468.73 | 142315682.9 | 71745289.86 | 46908179.42 | 19983321.01 | 29841627.5 | 54707539.61 | 39422679.87 |
|  |  | 1.111232476 | 0.501258169 | 0.037880335 | 237.00760 |  | 116133340.7 | 84461072.52 | 100114140.3 | 181266529.8 | 52560208.42 | 82852725.61 | 58528469.03 | 47652544.79 | 79748330.17 | 79958963.79 | 92636768.99 | 91754686.54 |
|  |  | 1.216645049 | 0.470009081 | 0.037962763 | 351.11185 |  | 156707143.7 | 90921757.06 | 76172908.98 | 154500282.4 | 37841878.37 | 88993579.85 | 53250275.76 | 44720592.58 | 37743524.3 | 35999052.7 | 69659598.96 | 60212814.27 |
|  |  | 3.294739709 | 3.610721122 | 0.038083619 | 602.44604 |  | 87384408.14 | 100912500 | 82707240.92 | 318583420.6 | 421437066.4 | 206412321.4 | 762399068.1 | 738587834.9 | 301541659.6 | 802585822.6 | 449330913.1 | 504562509.2 |
|  |  | 3.214923362 | 0.514208837 | 0.03834349 | 211.02818 |  | 1260645590 | 544514915.3 | 907584895.7 | 932925248.1 | 517613285.5 | 675542371.7 | 312722629.3 | 368757777.2 | 921967406.2 | 643205012.1 | 1202109913 | 863147294.1 |
|  |  | 2.435030281 | 0.45293934 | 0.038396774 | 388.25438 |  | 350513481.7 | 565913056.9 | 687147730.7 | 269369452.8 | 197823681.9 | 187703294.3 | 218744318.9 | 244058597.9 | 3231957364 | 949086108.8 | 265472866.6 | 313251527.5 |
|  |  | 9.880024925 | 1.570424707 | 0.038453564 | 375.29033 |  | 5249179482 | 4081190825 | 4992157934 | 5504364983 | 7561536092 | 10364603107 | 7851393353 | 5359110428 | 7159895293 | 6299000398 | 3889794343 | 3618533848 |
|  |  | 3.21438138 | 1.727575785 | 0.038480719 | 428.26534 |  | 487197077.2 | 615293144.5 | 438275605.3 | 441295014 | 635099224.5 | 1036185029 | 630609012.3 | 1122267048 | 597401900.7 | 593200366.6 | 346509949.5 | 308647924.8 |
|  |  | 1.371107875 | 0.370991223 | 0.038511494 | 236.11301 |  | 181159513.9 | 124623644.5 | 54364603.73 | 182989931.1 | 23770473.42 | 72192242.93 | 65384043.84 | 40152557.04 | 183723874.6 | 37123052.6 | 98070969.89 | 191205853.6 |
|  |  | 1.317996043 | 0.089260448 | 0.038697466 | 497.19811 |  | 68471394.71 | 102574555.5 | 13233556.93 | 148696690.7 | 2476630.649 | 4939723.091 | 4704720.116 | 17600530.89 | 11406209.82 | 14243138.21 | 17684061.42 | 15566917.88 |
|  |  | 2.605346778 | 0.12907108 | 0.038829354 | 188.05655 |  | 103822704.8 | 419335103.7 | 543011227 | 178216782.7 | 31855400.32 | 57913913.24 | 21594471.27 | 49250436.97 | 34128028.9 | 79425174.71 | 52434667.21 | 51688409.98 |
|  |  | 1.327475645 | 0.333344169 | 0.038841278 | 504.23537 |  | 99019907.84 | 57022472.48 | 150503837.1 | 162958667 | 31120795.06 | 86943851.02 | 31286231.37 | 7155837.992 | 126704727.1 | 191203257.7 | 222753764.7 | 204719141.1 |
|  |  | 1.14642876 | 0.189782927 | 0.039043641 | 273.07997 |  | 37555990.62 | 118333939.2 | 52726062.18 | 41486560.84 | 7016610.398 | 23066570.39 | 7039351.285 | 10342662.53 | 39923229.29 | 19403251.09 | 39489421.53 | 14940793.24 |
|  |  | 1.553234245 | 0.510305715 | 0.039231329 | 225.02220 |  | 262048181.6 | 112906232.6 | 200352721 | 259084366.1 | 88226448.07 | 157773073 | 91105584 | 88689646.72 | 128304503.4 | 89353456.07 | 148655599.2 | 127282002.5 |
|  |  | 1.142924605 | 2.523903292 | 0.039311235 | 877.36620 |  | 22011864.82 | 30039223.5 | 42763215.28 | 15360899.75 | 108975786.9 | 59864669.17 | 71297588.17 | 37933514.2 | 46707433.59 | 42809759.39 | 28958252.12 | 36552201.63 |
|  |  | 1.16388495 | 0.583094972 | 0.039545503 | 224.99312 |  | 169769354.7 | 84575002.13 | 146273447.9 | 148431009.3 | 70393325.89 | 115434073.8 | 65549419 | 68770784.07 | 88963312.12 | 72250224.5 | 120604535.1 | 92211720.18 |
|  |  | 1.223220233 | 0.502032914 | 0.039641064 | 181.05231 |  | 173216672.5 | 72353567.13 | 135155323.8 | 168962896.4 | 55338987.06 | 90770273.36 | 87107056.6 | 42745382.16 | 112274212.7 | 119870159.7 | 105940247.8 | 101380889.8 |
|  |  | 1.458992377 | 0.090719857 | 0.04017218 | 192.06556 |  | 177485654 | 111750752.2 | 4426714.505 | 121206409.9 | 9655535.746 | 7542743.286 | 8922829.073 | 11515796.52 | 18720676.44 | 9079856.326 | 8380769.623 | 3506178.026 |
|  |  | 2.262312954 | 0.473016831 | 0.04017554 | 379.21273 |  | 527785691.4 | 304614790.4 | 377842368.6 | 724577997.7 | 248807353.4 | 298382358 | 216039634 | 151973480 | 185169162 | 382199930.3 | 486809012.7 | 367315161.4 |
|  |  | 1.030745652 | 0.627741491 | 0.040386615 | 245.04907 |  | 151066684 | 108601507.4 | 144971719.5 | 138672922.8 | 74046716.01 | 116574128.7 | 107988705.9 | 42450457.4 | 114231158.4 | 135616490.4 | 135727944 | 120377279.2 |
|  |  | 1.137579704 | 0.555833256 | 0.040407532 | 227.12902 |  | 157796318.1 | 78604255.92 | 97848244.54 | 126167765.7 | 43648340.23 | 78976872.92 | 52578729.78 | 80710906.2 | 69114411.81 | 63580834.26 | 161935224.3 | 88001129.21 |
|  |  | 1.492910117 | 0.537579686 | 0.040560244 | 149.04554 |  | 269695375.3 | 179487623.8 | 174941415.1 | 348223285 | 141736675.5 | 152249830.4 | 94022229.63 | 134705635.5 | 289612943.5 | 278634930 | 66332139.69 | 253335596.8 |
|  |  | 1.318999107 | 0.23697654 | 0.040567627 | 289.06039 |  | 145888627.1 | 59200784.92 | 140486785.2 | 44405348.48 | 2157602.383 | 8230293.332 | 45133547.01 | 36895034.53 | 49202355.76 | 59458617.55 | 142991854.4 | 43701736.26 |
|  |  | 2.302583871 | 0.655057506 | 0.040581681 | 151.00682 |  | 773676868.4 | 525841680.5 | 674232868.3 | 700266537.5 | 403826662.1 | 648899380.4 | 365307958.6 | 333601531.3 | 552359635.4 | 548926840.8 | 623791977.3 | 509629308.2 |
|  |  | 2.806029392 | 4.767957908 | 0.040620592 | 149.08176 |  | 25377693.15 | 115670008.4 | 122090232.1 | 28570685.02 | 317112849 | 83987423.55 | 418444421.8 | 571309720.8 | 261654843.2 | 112556347.8 | 176901475.9 | 31008298.12 |
|  |  | 1.101542195 | 0.338117998 | 0.040665734 | 122.09649 |  | 129596930.8 | 89485233.37 | 59077325.73 | 43487676.54 | 34605562.1 | 36529009.49 | 33404543.36 | 4215581.173 | 56298579.79 | 28195366.55 | 182819425.4 | 39744667.13 |
|  |  | 1.415911017 | 0.450189321 | 0.040743867 | 158.16514 |  | 150119422.9 | 137719601.9 | 139136374.2 | 167230816.9 | 35668762.77 | 158925440.1 | 36138840.69 | 36772249.12 | 140763495 | 102353086.2 | 93361985.54 | 354925573.4 |
|  |  | 1.985628995 | 1.910912045 | 0.04077285 | 403.29605 |  | 144511047.8 | 245644088.3 | 107290226.8 | 162718900.8 | 296930222.8 | 410842253.4 | 183315838.1 | 370427529.1 | 234280363.5 | 258334596.8 | 235750073 | 238982445.5 |
|  |  | 1.188335481 | 0.64025899 | 0.040885574 | 407.17433 |  | 191322545.8 | 161028142.1 | 204392362.3 | 158660720.4 | 121197975.4 | 163129440.3 | 117684704.7 | 56031575.3 | 136215691.2 | 141436310.4 | 174311362.5 | 167203972.2 |
|  |  | 1.281059198 | 0.622485136 | 0.040970934 | 190.10759 |  | 252279984.6 | 179807462.4 | 161388431.9 | 212923846.5 | 130831386.4 | 174456912.4 | 126959259.2 | 69724284.57 | 162690149.2 | 125074011.5 | 22494425.02 | 167678451.4 |
|  |  | 1.780356176 | 0.340767423 | 0.041056806 | 176.12799 |  | 298422071.1 | 163619119.9 | 217565579.9 | 87838857.78 | 39470027.43 | 58949966.76 | 38620612.13 | 124479862.7 | 73332869.29 | 65339228.7 | 105794159.6 | 54254271.94 |
|  |  | 1.935279757 | 0.239283372 | 0.041092575 | 347.16331 |  | 294567283.7 | 82952570.89 | 243852795.6 | 122775358.4 | 13806487.93 | 100002430.7 | 3058600.081 | 61194725.88 | 17376061.4 | 124546064.8 | 30752361.82 | 110515372 |
|  |  | 1.06131068 | 0.54357108 | 0.041164249 | 249.04502 |  | 140148229.3 | 71405531.95 | 90994898.03 | 119978450.9 | 53526579.88 | 86855569.49 | 53719920.89 | 35571447.42 | 92008991.93 | 99870098 | 112965607.5 | 87989791.22 |
|  |  | 2.056732061 | 1.935633828 | 0.041261977 | 469.29312 |  | 121083474.3 | 143968286.3 | 94506607.09 | 205283114.3 | 395843017 | 236924137.2 | 277754753.4 | 182804372.5 | 351075952.7 | 286278609.1 | 162142652.9 | 495720250.3 |
|  |  | 1.38035417 | 1.678621299 | 0.041579814 | 583.29481 |  | 138497840.1 | 104149249.8 | 63299047.6 | 62857973.25 | 175675687 | 135158588.5 | 187547978.9 | 120700181.2 | 120481829.9 | 134138241.2 | 123432138.2 | 122613484.8 |
|  |  | 2.600472589 | 4.529036049 | 0.041834997 | 588.46643 |  | 47468628.84 | 31876604.6 | 47944432.95 | 152515302.8 | 232597749.7 | 98751911.16 | 481690725.2 | 454206405.9 | 111450463.2 | 272726538.4 | 251701153.2 | 204531736.4 |
|  |  | 1.49538672 | 0.503388886 | 0.041873041 | 117.05568 |  | 216021761.8 | 91362592.18 | 236653762.3 | 211451805.9 | 60903897.99 | 129416479.7 | 79215132.77 | 110769719.8 | 69907396.94 | 169674568.4 | 169328114.5 | 26012137.69 |
|  |  | 2.038272859 | 1.513158472 | 0.042100147 | 439.31782 |  | 277932699.3 | 327693376 | 176748360.6 | 307014070.9 | 503596256.3 | 427868015.9 | 296969906.7 | 419983269.8 | 288957296.2 | 339285586 | 257094179.1 | 274811570.1 |
|  |  | 6.44760371 | 0.47672849 | 0.042147601 | 407.17439 |  | 4336961099 | 2645496114 | 2489896318 | 4904162245 | 1556029337 | 2833149801 | 1607058214 | 857457300.3 | 1650577008 | 2733375733 | 3335523172 | 1209354443 |
|  |  | 2.636704726 | 0.601199488 | 0.042389694 | 196.01073 |  | 1030011885 | 829496094.9 | 761952020.1 | 661115222.1 | 500457646.2 | 627958296.6 | 639022167.1 | 206044432.7 | 1597611426 | 841893169.2 | 1384639018 | 1886378280 |
|  |  | 1.067773099 | 0.472002591 | 0.043312147 | 289.08618 |  | 85835308.59 | 87875145.66 | 150115253.5 | 65119144.18 | 23473408.8 | 62046232.83 | 45560089.53 | 52503246.8 | 21699698.58 | 83741057.57 | 51930336.79 | 90206991.63 |
|  |  | 1.125029439 | 0.598543099 | 0.043605101 | 277.12179 |  | 209911154.3 | 110768001.4 | 130471719.9 | 129143051.6 | 86186708.84 | 103043739.2 | 83361746.56 | 74738730.91 | 118689147.3 | 126647705 | 164258572.2 | 139335376.5 |
|  |  | 1.480742132 | 0.455162593 | 0.043864455 | 144.08030 |  | 184353810.2 | 220578896.2 | 144179004.2 | 112773169.2 | 21638073.29 | 137760637.1 | 42370538.58 | 99495989.41 | 22337769.76 | 90177009.94 | 275024984.8 | 86461019.64 |
|  |  | 1.967196279 | 0.647414604 | 0.043931247 | 182.99684 |  | 565053593.5 | 359262805.6 | 501480664.2 | 438745866.4 | 259649560.5 | 443259850.7 | 262381108.7 | 241841802.5 | 481260680.9 | 434648542.9 | 480774918.7 | 431452325.6 |
|  |  | 1.422678341 | 0.582483552 | 0.043996564 | 238.07562 |  | 256054271.3 | 140869070 | 217647421 | 178933925.1 | 94769607.77 | 178579112.2 | 100145391.5 | 88709317.39 | 191161542.5 | 160370911.1 | 167624975.6 | 176286318.4 |
|  |  | 1.669938783 | 0.499543935 | 0.044209132 | 459.34729 |  | 306122702.5 | 113916257.7 | 261247955.4 | 288134766.2 | 94698688 | 173274754.8 | 126013072 | 90282206.82 | 97973446.28 | 157101470.8 | 182458213.2 | 152747346.3 |
|  |  | 3.094458595 | 3.822334058 | 0.044383903 | 616.46154 |  | 76682267.17 | 87951278.27 | 74671304.74 | 261143878.7 | 450674275.6 | 195864921.6 | 832629802.5 | 433713221 | 330248722.7 | 402273372.4 | 333730802 | 401970338.4 |
|  |  | 2.310681688 | 2.167502747 | 0.044403514 | 449.24415 |  | 146047993.4 | 183884741.6 | 146629277.3 | 143625318.4 | 252476016.4 | 471114817.6 | 439840138.3 | 180826770.8 | 282001307.1 | 210406359.7 | 184097991 | 159465158.4 |
|  |  | 1.159389407 | 3.685913506 | 0.044786357 | 423.20568 |  | 21695533.67 | 25979403.8 | 14800440.05 | 20772515.31 | 11209694.75 | 96420621.93 | 93473980.8 | 105740235 | 100853562.4 | 8033369.445 | 27660908.52 | 23866042.12 |
|  |  | 1.337011876 | 0.556411705 | 0.044840242 | 498.32294 |  | 233284027.9 | 155864666 | 139606822.5 | 159724650.9 | 42172230.28 | 143748355 | 115088155.3 | 82069683.37 | 106938855.9 | 90045036.24 | 71008884.21 | 60839366.82 |
|  |  | 1.295320156 | 1.542962962 | 0.044998809 | 435.31176 |  | 101359854 | 102506740.2 | 86982961.27 | 102464236.5 | 205681699.9 | 163195345.2 | 116967160.7 | 121024407.8 | 78306167.29 | 86563391.81 | 75106732.4 | 46833737.06 |
|  |  | 1.984278049 | 0.231529486 | 0.045046344 | 520.33335 |  | 120871616.9 | 286730069.3 | 349298155.2 | 84949263.04 | 36551779.77 | 41590399.69 | 55489514.35 | 61281196.72 | 1889878938 | 479948337.7 | 79785349.58 | 145686446.9 |
|  |  | 1.293150297 | 0.458644612 | 0.045110457 | 252.15957 |  | 186879441 | 99283501.71 | 83097430.12 | 123529425.7 | 36658356.4 | 58899474.95 | 36955866.38 | 93501688 | 100764318.3 | 133656179.4 | 80317508.97 | 127524031.3 |
|  |  | 1.086618939 | 0.375212078 | 0.045338667 | 303.17037 |  | 57909848.22 | 63267203.27 | 78247685.62 | 145908506.4 | 14498056.08 | 37375885.2 | 31145995.93 | 46553266.53 | 41816615.27 | 28634716.16 | 43110708.32 | 67458754.6 |
|  |  | 1.434237831 | 0.552305522 | 0.045490007 | 347.24394 |  | 214962727 | 154271843.8 | 148472291.2 | 216538221.2 | 43602441.32 | 169219121.6 | 78441737.34 | 114264313.6 | 161783076.3 | 181727114.5 | 182403209.2 | 227767490.6 |
|  |  | 1.633822678 | 2.957639647 | 0.045570776 | 588.43051 |  | 28613499.58 | 35499110.77 | 38979618.45 | 98066196.28 | 124836309.9 | 68542910.57 | 238617340.5 | 162957572.4 | 131910834.5 | 376838629.7 | 214497547.3 | 252727974.2 |
|  |  | 1.618624157 | 0.627150666 | 0.045798074 | 248.02353 |  | 375158320 | 299848917.1 | 357941879.5 | 230253094.1 | 176971381.5 | 286698910.4 | 202451991.6 | 126095823.8 | 246602379.1 | 409741942.2 | 544841496.1 | 463255186.8 |
|  |  | 1.140228149 | 0.535713175 | 0.045939135 | 419.13782 |  | 131810244.3 | 121149262.2 | 115541752.5 | 188300844.2 | 93143114.19 | 107956840.1 | 78587037.84 | 18599230.65 | 87046982.36 | 107590254.7 | 113000509.3 | 104582610.2 |
|  |  | 1.944410025 | 0.474578408 | 0.046183559 | 230.99697 |  | 508440189.8 | 192062584.5 | 357239051.8 | 270282793.5 | 145843453 | 118679925.1 | 176494610.9 | 189233820.7 | 301278880 | 282051134.9 | 348390363.9 | 291792142.4 |
|  |  | 1.032081171 | 0.313266759 | 0.046576192 | 427.17969 |  | 90645761.26 | 34034787.96 | 45746949.12 | 116968264.7 | 20054879.82 | 33423554.61 | 21095225.24 | 15457879.65 | 32123724.26 | 77004033.03 | 98298552.11 | 73748823.02 |
|  |  | 1.530255321 | 0.624618991 | 0.046578574 | 165.02220 |  | 339607981.1 | 184642432.5 | 290992763.4 | 306260048.5 | 166076051 | 247736061.2 | 158383846.8 | 128316253.8 | 197317084.8 | 202942610.7 | 235541004.3 | 210165050.3 |
|  |  | 2.771364847 | 1.806802324 | 0.046674947 | 355.26336 |  | 224928399.1 | 588871924.7 | 382463567.9 | 239892041 | 467615963.6 | 730267760 | 823890313.5 | 573075839.4 | 334085405.8 | 929777278.2 | 276279048.2 | 286928864.8 |
|  |  | 1.25261304 | 0.535330249 | 0.046711729 | 229.01761 |  | 131384427 | 205543783 | 151168615.3 | 95955377.49 | 80092664.53 | 112339250.1 | 79421234.44 | 40807662.31 | 113516112.2 | 112288005.2 | 129903176.9 | 128864461.6 |
|  |  | 1.095541328 | 0.52679858 | 0.046763713 | 231.16026 |  | 135223039.3 | 67364744.45 | 104810944.5 | 118551490.3 | 50426222.19 | 94140706.81 | 53725917.84 | 26097123.57 | 26475999.48 | 16512524.1 | 13350842.48 | 19237547.61 |
|  |  | 1.926660513 | 2.002671641 | 0.04677296 | 213.12365 |  | 162367611.5 | 108331551.8 | 143718053 | 142959338.6 | 285049710.1 | 354331127.4 | 122092699 | 354768683.1 | 90779713.95 | 355032491.4 | 82880304.13 | 96296399.18 |
|  |  | 1.680222618 | 1.847438609 | 0.04694082 | 87.02019 |  | 94792479.63 | 224147109.9 | 133321180 | 78141361.57 | 176131631 | 311604679.4 | 211337178.1 | 280811886.6 | 154564860.5 | 122349915.7 | 152221003 | 651937973.5 |
|  |  | 2.027234378 | 0.571611084 | 0.047048202 | 239.02318 |  | 468457056.4 | 245611958 | 454941099.5 | 411736220 | 174730262.3 | 352479758.2 | 154127779.4 | 222234324.9 | 380891739.3 | 357078185.3 | 293009240.6 | 268452299.6 |
|  |  | 1.918888534 | 0.4402922 | 0.04716061 | 363.21685 |  | 271985942.1 | 184493640.2 | 225794623.9 | 419365850.6 | 52477434.5 | 105249940.9 | 109141256 | 218174892.5 | 77636508.75 | 107176340 | 118127274.3 | 64010760.79 |
|  |  | 1.008028694 | 0.288629088 | 0.047261363 | 185.02222 |  | 99174580.56 | 63360993.59 | 33780143.33 | 39980820.58 | 12430273.54 | 7363105.088 | 7264281.758 | 41144393.82 | 22809967.94 | 19596775.2 | 40874728.81 | 14735155.68 |
|  |  | 2.192735106 | 3.343977221 | 0.047858332 | 253.00236 |  | 46669858.24 | 37671460.02 | 88564360.86 | 122740966.4 | 248284537.4 | 59383132.93 | 365636483.4 | 315331494.2 | 103442485.8 | 69587594.76 | 106143374.9 | 100117910.6 |
|  |  | 1.181597248 | 0.528832055 | 0.047907107 | 362.20758 |  | 154610345 | 74817124.08 | 133742710.3 | 187273734.8 | 61998305.33 | 105691752 | 66915550.38 | 56486778.23 | 101872498.1 | 62283518.65 | 99568511.39 | 110924971.8 |
|  |  | 1.947277631 | 0.295577349 | 0.047937341 | 432.28072 |  | 142415958.2 | 307993337.2 | 357775796.6 | 92069417.5 | 67049085.9 | 57831798.85 | 72017976.77 | 69195979.82 | 1812241858 | 501324495.6 | 94842873.45 | 133822104.5 |
|  |  | 1.441534914 | 0.524970356 | 0.048143441 | 391.17962 |  | 217968583.7 | 154890502.5 | 168627764.9 | 320010994.1 | 98248116.99 | 165474555.1 | 98534830.74 | 90003327.83 | 132782751.8 | 97676705.88 | 125038989.3 | 152789835.7 |
|  |  | 2.004374485 | 0.28772553 | 0.048183237 | 564.35963 |  | 127096854.9 | 301333732.2 | 399331811.6 | 123997603.5 | 48440557.23 | 58311392.48 | 81247726.54 | 85845975.24 | 2224222881 | 685025568.3 | 89246465.53 | 201644423.4 |
|  |  | 1.5679871 | 0.543277417 | 0.048776323 | 289.07514 |  | 303290253.1 | 176194339.6 | 234837767 | 187625067.1 | 107956481.7 | 196316358.4 | 134833034.8 | 50901793.29 | 215845603.5 | 166225585.4 | 270197231.5 | 210142216.3 |
|  |  | 2.589157352 | 2.810688447 | 0.048919909 | 495.27854 |  | 42989400.47 | 161718841.4 | 177614912.7 | 108886202.5 | 439013466.6 | 198684633.2 | 537409903.2 | 205528461.8 | 1052929554 | 188781389 | 115041584.3 | 112400424.4 |
|  |  | 1.07606886 | 0.595581735 | 0.048920301 | 426.26199 |  | 139427380.1 | 127044761.7 | 172567445.7 | 218948204.4 | 105399527.6 | 112984752.2 | 126505436.7 | 46995793.92 | 192314928.2 | 170506965.8 | 217838289.2 | 277187336 |
|  |  | 1.967384111 | 3.555502271 | 0.049125565 | 89.01445 |  | 42180930.56 | 84868869.71 | 86614403.45 | 23667312.44 | 278238264.1 | 148174091.8 | 78037612.1 | 339382776.6 | 88832762.5 | 100789505.9 | 174560684.4 | 137654047.1 |
|  |  | 1.709189253 | 2.348624583 | 0.049129172 | 431.31668 |  | 67279603.47 | 66542369.75 | 117138197.9 | 71911976.61 | 147131365.2 | 173841212.3 | 313465741.1 | 123867144.8 | 138705804 | 175529416.8 | 80672636.02 | 65037263.72 |
|  |  | 1.177748371 | 6.626376185 | 0.049481894 | 434.29136 |  | 7203453.123 | 17082070.87 | 10812238.3 | 6767540.431 | 29225507.31 | 54081557.67 | 138649579.7 | 55458600.26 | 41300762.77 | 30645427.09 | 25834458.05 | 14364364.66 |
|  |  | 1.83398648 | 0.215890599 | 0.049519087 | 303.09093 |  | 90495681.3 | 187692104.4 | 143536406.2 | 384185567 | 44276182.13 | 60747354.15 | 31022037.75 | 37942766.95 | 53669129.52 | 65547922.19 | 64140578.04 | 62874802.71 |
|  |  | 2.310182672 | 2.495357454 | 0.049593794 | 405.26448 |  | 44162393.69 | 219178802.8 | 114073478 | 64697148.76 | 388745574.3 | 229046807.7 | 340255986.9 | 145178664.5 | 159866627.5 | 201838293.5 | 59981529.8 | 70886026.25 |
|  |  | 2.323419395 | 0.554337914 | 0.049617912 | 160.09699 |  | 689037095.8 | 384534359.1 | 401215809.3 | 428206845.7 | 169577453.9 | 318650608.7 | 195572063.1 | 371101659 | 448607126.1 | 287181796.8 | 345627154.4 | 346950224.6 |
|  |  | 4.226046066 | 22.02900997 | 0.049673953 | 273.98547 |  | 43672464.58 | 21888527.38 | 37560953.07 | 38251485.51 | 937555859.9 | 1483706784 | 25045525.17 | 668008541.3 | 51154965.72 | 19374922.16 | 42297393.46 | 39903881.3 |
|  |  | 2.161959342 | 0.668659588 | 0.049739435 | 205.01767 |  | 731551950.2 | 454026526.5 | 621876134.6 | 671105472.8 | 357498558.5 | 590955680.3 | 356676882.8 | 352181842.6 | 525703948.5 | 554221137.8 | 591998890.7 | 1072605827 |
|  |  | 1.45437892 | 2.5966782 | 0.049766197 | 131.03687 |  | 32813511.49 | 33068015.27 | 88926274.93 | 31437713.58 | 99799720.91 | 83902686.43 | 99220932.46 | 200696329.6 | 22808227.4 | 126667672.5 | 103134218 | 191907804.6 |
