# Supplementary material for: To explore the mechanism of acupoint application in the treatment of primary dysmenorrhea by 16S rDNA sequencing and metabolomics
Source: Front Endocrinol (Lausanne). 2024 May 30;15:1397402. doi: 10.3389/fendo.2024.1397402 (PMC11169635; doi:10.3389/fendo.2024.1397402)
Supplement: Supplementary file 3 [file Table_3.docx]

Table S3: Treatment vs Model Volcano Plot

| **adduct** | **Name** | **VIP** | **Fold change** | **p-value** | **SuperClass** | **Control-1** | **Control-2** | **Control-3** | **Control-4** | **Model-1** | **Model-2** | **Model-3** | **Model-4** | **Treatment-1** | **Treatment-2** | **Treatment-3** | **Treatment-4** |
| --- | --- | --- | --- | --- | --- | --- | --- | --- | --- | --- | --- | --- | --- | --- | --- | --- | --- |
| [M+H]+ | 6-oxirane boldenone | 2.093878247 | 0.140833146 | 1.16887E-06 | Lipids and lipid-like molecules | 23481155.62 | 48803162.03 | 11607048.58 | 19783339.85 | 168460164.5 | 143771995 | 160027077.2 | 148762350.6 | 18370956.74 | 16971980.56 | 18393645.53 | 33723841.24 |
| [M+H]+ | Ethoxyquin | 3.283471884 | 2.440447841 | 5.28412E-06 | Organoheterocyclic compounds | 491841523.6 | 275656077.1 | 405098635.1 | 349705043.2 | 214997916 | 269553647.5 | 229106579.5 | 229132123.9 | 615778705.8 | 599313118.5 | 548910631.6 | 536828015.2 |
| [M+H]+ | Biotin | 2.927063914 | 0.245134519 | 5.72717E-05 |  | 105396353.6 | 158176512.3 | 75412878.94 | 76208614.8 | 405744793.3 | 367893647.8 | 333494428 | 284330227.7 | 94906308.83 | 73488665.62 | 88953957.75 | 83746705.08 |
| [M+H]+ | Deoxypeganine | 2.729132966 | 0.099735421 | 8.36381E-05 | Organoheterocyclic compounds | 32770075.34 | 46065484.39 | 21892339.44 | 27024003.61 | 232838342.7 | 301667042.6 | 280649622.2 | 193810711.7 | 25152825.94 | 25568759.26 | 25794273.19 | 24113762.58 |
| [M+H]+ | L-tryptophanamide | 2.869257952 | 4.580565933 | 9.2713E-05 | Organoheterocyclic compounds | 79426434.62 | 32583366.35 | 101411900.4 | 83285289.7 | 41159838.76 | 116922857.8 | 96502561.71 | 37492512.21 | 395060848.3 | 315952230.9 | 317030868.6 | 309837537.5 |
| [M+H]+ | Metribuzin | 1.713189806 | 0.334780601 | 0.000194574 | Organosulfur compounds | 46640526.28 | 47571373.51 | 40015136.02 | 33852478.81 | 170951088.1 | 129322724.2 | 118926178 | 138131241.3 | 44626551.48 | 50092707.12 | 50947228.88 | 40917196.96 |
| [M-H]- | Xanthine | 6.032048348 | 2.498963793 | 0.000243027 | Organoheterocyclic compounds | 1454450923 | 1375800687 | 1026498655 | 2483539283 | 518418831.6 | 713932297.2 | 738572891.6 | 1078151050 | 1638052103 | 2039051906 | 1950204194 | 1992220001 |
| [M+H]+ | (+)-fluprostenol methyl ester | 1.849733572 | 3.698754637 | 0.000259773 | Lipids and lipid-like molecules | 138362958.7 | 30775983.72 | 133187195.6 | 174011304.6 | 34410771.09 | 70581316.69 | 30177177.73 | 28406183.3 | 122653121.3 | 151520271.3 | 159071755 | 171780302.1 |
| [M+H]+ | N-methyl-l-isoleucine | 5.278463508 | 3.377705659 | 0.000298967 | Organic acids and derivatives | 1543153788 | 685516538 | 1151290240 | 919890683.3 | 193388525.6 | 482575033.3 | 196620384.2 | 569788452.8 | 1311638609 | 1038855766 | 1245469969 | 1275945059 |
| [M+H]+ | Undecanoic acid | 1.68228807 | 5.469846112 | 0.000365482 | Lipids and lipid-like molecules | 10424037.4 | 29424700.22 | 33436875.52 | 11820837.1 | 8966111.587 | 21269703.78 | 20912878.92 | 30417672.03 | 92489022.44 | 104001197.3 | 103361261.6 | 146303990.4 |
| [M+H]+ | L-propionylcarnitine | 1.417653447 | 2.034656294 | 0.000368488 | Lipids and lipid-like molecules | 127164333.7 | 133183801 | 135336236.4 | 96960161.89 | 75166991.17 | 84363060.39 | 52916001.11 | 47408197.78 | 124002889.3 | 130562651.1 | 137250894.2 | 136897651.5 |
| [M+H]+ | 11alpha-hydroxyprogesterone | 1.402123211 | 2.827911159 | 0.00039503 | Lipids and lipid-like molecules | 43513866.26 | 67690743.81 | 26967269.99 | 111486194.4 | 45921302.9 | 43265799.36 | 29468412.4 | 24402771.23 | 109710263.5 | 98079615.25 | 81149256.22 | 115616988.1 |
| [M-H]- | 21-Hydroxypregnenolone | 11.01266555 | 4.344073654 | 0.000484838 | Lipids and lipid-like molecules | 1575906549 | 1328056571 | 1060655956 | 900147715.7 | 408003259.3 | 1581502875 | 729005850.7 | 1869071524 | 5346933697 | 5735887602 | 3705530727 | 5140448629 |
| [M-H]- | Probucol | 5.345438329 | 0.334469081 | 0.000531648 | Benzenoids | 330418835.3 | 936901681.8 | 1000304860 | 402033692.1 | 1308345619 | 1554620681 | 1407718081 | 989838498.9 | 467646171.1 | 560246883.6 | 310723786.1 | 420865412.6 |
| [M+H]+ | Arachidonoylserotonin | 1.276217104 | 0.253424498 | 0.000649928 | Organoheterocyclic compounds | 1879168.375 | 13180819.71 | 3192195.844 | 9907000.383 | 72597188.46 | 58334478.73 | 73937964.26 | 61893977.1 | 11771719.98 | 36168663.32 | 5702658.447 | 13961391.76 |
| [M-H]- | N-methylleucine | 1.10676618 | 3.40839462 | 0.001213506 | Organic acids and derivatives | 87788400.62 | 26212050.32 | 50694588.91 | 44120302.08 | 6693308.413 | 18205783.41 | 5892636.751 | 31857874.1 | 58561610.87 | 46878795.67 | 55326566.68 | 52767595.48 |
| [M+H-CH3ON]+ | DL-arginine | 2.582326795 | 1.795230493 | 0.001568908 | Organic acids and derivatives | 732105658 | 292619225.4 | 362919616.2 | 535270156.3 | 362017745.6 | 361659410.6 | 200146576.5 | 261687424.4 | 536206897.5 | 560395357.1 | 484536933 | 547126592 |
| [M+H]+ | 7,8-dihydroneopterin | 2.062005413 | 2.048582095 | 0.001590268 | Organoheterocyclic compounds | 314368125.1 | 166263398.2 | 223425920.7 | 233018686.5 | 117735122.5 | 200769369.1 | 99764500.64 | 120248257.2 | 289336510.4 | 302371683.4 | 243889222.2 | 267599379.3 |
| [M+H]+ | 3,4-dimethoxychalcone | 3.721582752 | 0.050393532 | 0.001780301 | Phenylpropanoids and polyketides | 35025219.71 | 80275685.91 | 26211253.02 | 29834514.07 | 337873588.3 | 593583964.4 | 640093740.1 | 317322858.6 | 25604393.09 | 23917275.29 | 26012041.8 | 19653329.17 |
| [M+H-H2O]+ | Hydrocortisone | 1.292273281 | 2.604597708 | 0.001997502 | Lipids and lipid-like molecules | 40767464.13 | 44906637.41 | 50967302.26 | 30177238.57 | 21858218.9 | 49450044.81 | 19100195.17 | 42376474.82 | 103262224 | 70135466.5 | 90115087.02 | 82338556.46 |
| [M+H]+ | Cys-Ile | 1.61299901 | 4.583120816 | 0.002121518 | Organic acids and derivatives | 50376743.56 | 25140539.46 | 38876582.2 | 32056773.52 | 22635632.79 | 30338565.29 | 24581246.66 | 20377993.39 | 149504521.6 | 85254933.3 | 132147025 | 81934298.98 |
| [M+H]+ | Metamitron | 1.971646961 | 4.418515071 | 0.002273666 | Organoheterocyclic compounds | 113107153.6 | 52570330.28 | 46503153.05 | 62137327.03 | 33575478.59 | 44759100.09 | 48751141.48 | 25388387.51 | 166859992.7 | 101361432.2 | 183878432.3 | 221609285.4 |
| [M+H]+ | 16-phenoxytetranorprostaglandin f2.alpha. cyclopropyl methyl amide | 1.064471847 | 5.018107735 | 0.002631269 | Lipids and lipid-like molecules | 20608043.8 | 8674031.746 | 24661750.94 | 19287278.73 | 10981432.11 | 10108894.86 | 6225222.812 | 10367280.91 | 63977997.57 | 27066099.17 | 49143605.35 | 48908802.13 |
| [M+H]+ | Mefenamic acid | 2.135910641 | 1.565647632 | 0.003047129 | Benzenoids | 432455945.7 | 244018956.4 | 361088188.9 | 413934277.1 | 235380049.4 | 324552170.3 | 197121634.2 | 304773438.6 | 432458667.6 | 433069501.5 | 402153722.4 | 394765494.5 |
| [M-H]- | Uracil | 6.832867772 | 1.524949923 | 0.003099134 | Organoheterocyclic compounds | 4552578472 | 5149063596 | 3302116647 | 5451591064 | 2604453327 | 3947536316 | 3636933089 | 2631607222 | 4690742283 | 4968423707 | 5031070918 | 4860429256 |
| [M-H-CO2]- | Telmisartan | 3.285012526 | 0.17995677 | 0.003182423 | Benzenoids | 99725182.85 | 1302260706 | 353656417.4 | 178087309 | 478652527.6 | 583900030.9 | 287700268.3 | 365046645.2 | 52414083.12 | 183740027.8 | 27906607.73 | 44619033.61 |
| [M+H]+ | N-methyltryptamine | 2.614208425 | 2.513847043 | 0.003349162 | Organoheterocyclic compounds | 162549679.7 | 215145377.7 | 197255460.6 | 153928143.9 | 40087596.04 | 231666450.9 | 133403107.1 | 171815203.1 | 363715003.8 | 296278545.2 | 388896777.2 | 401529927.5 |
| [M+H-C2H4O2]+ | Oleana-1,9(11)-dien-28-oic acid, 2-cyano-3,12-dioxo-, methyl ester | 1.420093664 | 2.74261329 | 0.003422015 | Organic oxygen compounds | 38326936.53 | 37285245.24 | 73343657.44 | 38278880.18 | 43486828.04 | 44383457.69 | 22040870.33 | 49423663.33 | 146336234.3 | 93704083.34 | 111827146 | 85126329.59 |
| [M+H]+ | N-phenylmorpholine | 1.550523262 | 1.700763889 | 0.003497905 | Organoheterocyclic compounds | 204737734.6 | 67651508.53 | 192583954.6 | 85163970.19 | 92474952.82 | 150511439.8 | 139973791.9 | 107707270 | 197450089.5 | 231859116.9 | 226585079.5 | 178615202 |
| [M+H]+ | Methapyrilene | 2.484015373 | 3.023513688 | 0.003949348 | Organic nitrogen compounds | 135143784.8 | 82909940.3 | 100304446 | 243494037.4 | 23893527.38 | 130004909.3 | 105528274.1 | 149110520.3 | 388796253.7 | 340176373 | 219889326.6 | 286355956.9 |
| [M-H]- | Artocaprin | 1.820587872 | 2.60494487 | 0.004140065 | Phenylpropanoids and polyketides | 190958740.7 | 176585480.9 | 159737826.3 | 117544306.3 | 61690993.28 | 120977695 | 70938192.05 | 27558662.03 | 215925178.5 | 160576509.2 | 204999575.8 | 150919473.8 |
| [M+H-C2H2O]+ | Zaleplon | 2.896826461 | 1.895807132 | 0.004276449 | Organoheterocyclic compounds | 846262763.5 | 590199051.7 | 535152801.9 | 538928078.4 | 272928022.4 | 380239037.1 | 375128094.9 | 302365857.8 | 550406625.9 | 508220256 | 770787294.8 | 693262460.9 |
| [M+H]+ | Pirimicarb | 2.428723633 | 2.85126379 | 0.004609569 | Organic nitrogen compounds | 203223726.1 | 88836129.57 | 146961573.6 | 125746189.7 | 155934958.8 | 130926302.6 | 102532802.9 | 72303963.08 | 351998791 | 299878017.5 | 439751598.6 | 224794460.5 |
| [M-H]- | Inosine 5'-monophosphate | 1.340229234 | 2.53816968 | 0.005216999 | Nucleosides, nucleotides, and analogues | 97546369.78 | 121949179.2 | 125797240.9 | 83609739.52 | 44597246.51 | 66570627.7 | 37958286.92 | 14215064.28 | 92349264.02 | 133239424.7 | 91122071.37 | 97876985.63 |
| [M-H]- | Pravastatin | 4.104446157 | 0.051774249 | 0.005264263 | Organic acids and derivatives | 1321025522 | 1719849607 | 1488662705 | 1562142390 | 742756798.6 | 731005498.3 | 518700753.1 | 221747273.4 | 14598416.61 | 24043346.73 | 3679309.286 | 72318003.37 |
| [M+H]+ | Trigonelline | 3.30509473 | 2.409525739 | 0.005301308 | Alkaloids and derivatives | 367900573.8 | 296845137.5 | 159173522.2 | 264720159.8 | 263151786.3 | 348312973.5 | 293875564.7 | 196741259.5 | 564800142.3 | 511011366.4 | 683410177.7 | 896272256.4 |
| [M-H]- | 1,3,5(10)-estratrien-3,17.beta.-diol 17-glucosiduronate | 1.081744928 | 2.448868371 | 0.005398306 | Lipids and lipid-like molecules | 25089014.48 | 25723545.81 | 16657896.45 | 38155168.77 | 38068075.63 | 24864886.09 | 32520428.39 | 21127002.22 | 88089125.52 | 80441303.98 | 45858210.52 | 71101395.39 |
| [2M-H]- | 3.alpha.-hydroxy-7-oxo-5.beta.-cholanic acid | 4.659374971 | 0.408570232 | 0.005477229 | Lipids and lipid-like molecules | 87352538.08 | 331532878 | 153378432.8 | 68366666.56 | 1031474544 | 1259850908 | 1653506788 | 1009290592 | 605670493.2 | 690085179.1 | 304703069.5 | 423648372.6 |
| [M+H-H2O]+ | (-)-.alpha.-kainic acid | 2.42535337 | 1.737946533 | 0.005890359 | Organic acids and derivatives | 438430353.3 | 255269826.5 | 385026545 | 337780636.4 | 197553629.4 | 377924207.5 | 181791967.2 | 293596250.6 | 462838922.9 | 457581556.2 | 473852184.3 | 432076353.2 |
| [M-H]- | Phenylbenzimidazolesulfonic acid | 1.01658564 | 12.47162546 | 0.005934237 | Organoheterocyclic compounds | 1393134.946 | 1816230.612 | 3134960.053 | 5162800.739 | 3349505.819 | 7033790.732 | 1432042.227 | 753149.8643 | 36160703.33 | 57279345.16 | 17051925.8 | 46257508.66 |
| [M-H]- | Neoabietic acid | 3.242078758 | 1.880386437 | 0.006184754 | Lipids and lipid-like molecules | 1094822209 | 561695079 | 583294056.5 | 1118085517 | 323735862.6 | 569806884.1 | 351894129.2 | 395783924.6 | 770871492.3 | 687619439.4 | 664040265.9 | 963598136.6 |
| [M+H]+ | Arcaine | 1.686271094 | 2.9334913 | 0.006189277 | Organic nitrogen compounds | 32986114.83 | 77573601.07 | 103716839.8 | 48905068.54 | 14598176.67 | 52865022.49 | 65406502.77 | 72342278.63 | 124463179.2 | 193697376.5 | 175530783.4 | 108296220.6 |
| [M-H]- | Pyridoxamine 5-phosphate | 1.843315266 | 3.026923168 | 0.006258197 | Organoheterocyclic compounds | 236901329 | 238205243.7 | 220618249.2 | 143270203.3 | 58991380.18 | 68888032.31 | 58331252.34 | 56236119.25 | 167190867.2 | 154972487.8 | 271147179.7 | 140557253.1 |
| [M+H-CH2O]+ | Apiole | 1.146843645 | 1.900402913 | 0.006880809 |  | 109861571.2 | 54536717.79 | 105645186 | 132317556.7 | 50197263.08 | 71621466.36 | 42550253.01 | 44975212.2 | 127901850.1 | 90260083.5 | 94059578.74 | 85616804.94 |
| [M-2H]2- | Plantamajoside | 3.434412887 | 3.391200134 | 0.007171632 | Phenylpropanoids and polyketides | 1163585014 | 271969784.2 | 298055254 | 1067766642 | 219232837.3 | 193704281.8 | 184287002.9 | 100903301.1 | 585018390.5 | 310061669.1 | 768167232.9 | 704242518.4 |
| [M+H]+ | L-histidinol | 2.711757776 | 2.101843297 | 0.007381336 | Organic nitrogen compounds | 497540675.9 | 211038412.9 | 347754778.9 | 408874807.7 | 135200568 | 297239030.6 | 191586671.3 | 271942468 | 470749098.8 | 384435987.6 | 610508088.3 | 417492711.6 |
| [M-H]- | Trans-traumatic acid | 2.51169028 | 2.036203708 | 0.008090899 | Lipids and lipid-like molecules | 731668420.8 | 623438025.5 | 417124457.1 | 542561363.3 | 208996467.3 | 329224541.5 | 185954400 | 171393595.9 | 349473792.6 | 509677505.7 | 558366605 | 406043025 |
| [M+H]+ | 2-ethyl-2-phenylmalonamide | 1.062438074 | 2.265511356 | 0.008323653 | Benzenoids | 36227623.66 | 34048659.36 | 55201953.83 | 34053441.62 | 26996744.52 | 32704245.87 | 18022590.15 | 45401775.29 | 94389743.42 | 58916301.43 | 64307777.61 | 61328069.34 |
| [M-H]- | 4'-hydroxychalcone | 3.281140309 | 1.638995814 | 0.008335881 | Phenylpropanoids and polyketides | 1074136797 | 598732120 | 934171551.9 | 912846387.8 | 426027144.6 | 831451472.2 | 611283606.8 | 481486169 | 1021444751 | 881216918.9 | 1027615175 | 921770433.3 |
| [M+Na]+ | N-.alpha.-(tert-butoxycarbonyl)-l-valine | 2.34017692 | 1.826078686 | 0.008483521 | Organic acids and derivatives | 459010903.1 | 284276144.2 | 377028905 | 289905091.2 | 205831686 | 356446897.7 | 179641975.8 | 191117585.4 | 445551872.6 | 420040037.3 | 486208140.7 | 352001019.1 |
| [M-H]- | Xanthosine | 1.406930762 | 16.35725397 | 0.008516295 | Nucleosides, nucleotides, and analogues | 71225322.5 | 12033355.29 | 12954972.1 | 24382237.64 | 1567159.531 | 4948036.304 | 6773183.5 | 4643718.553 | 97812131.47 | 31488064.03 | 56184056.44 | 107835627.4 |
| [M+Na]+ | Propentofylline | 1.353215578 | 2.567839477 | 0.008677999 | Organoheterocyclic compounds | 40077170.72 | 38325195.06 | 66876359.27 | 33983930.42 | 30444193.03 | 52168450.26 | 17397562.52 | 58992811.2 | 139939520.8 | 81677742.96 | 99142876.43 | 87534083.79 |
| [M+H]+ | Ser-Gly-Ser | 1.580060097 | 1.791735746 | 0.008760216 | Organic acids and derivatives | 217839881.1 | 133653018.1 | 179255248 | 210020079.3 | 66469271.47 | 164726162.2 | 91576025.93 | 103506786 | 208615618.5 | 182086675 | 196544394.5 | 176531282.7 |
| [M+H]+ | Vincamine | 1.14164526 | 2.644052506 | 0.008882867 |  | 21374510.45 | 28020442.76 | 41452164.75 | 19129442.46 | 30291797.34 | 33162181.76 | 12914695.1 | 37272263.68 | 107857583 | 66400277.2 | 66577871.06 | 59636875.24 |
| [M-H]- | D-sorbitol | 1.274745923 | 3.511653264 | 0.008891279 | Organic oxygen compounds | 77986887.06 | 38879953.92 | 36806719.73 | 43802818.5 | 17767586.44 | 34248244.96 | 18013069.48 | 24807667.15 | 47494062.22 | 73942332.47 | 119465085.7 | 92131663.23 |
| [M-H]- | Lapachol | 5.450200975 | 3.303173504 | 0.00933198 | Lipids and lipid-like molecules | 1522123835 | 756793818.8 | 712958752.2 | 844543416.1 | 371935029 | 666675360.1 | 359310258.4 | 503916876.1 | 921030383.3 | 1429183614 | 2280964300 | 1650921019 |
| [M+H]+ | Pyroglu-Arg-Arg | 2.028207927 | 0.447269213 | 0.009828544 | Organic acids and derivatives | 218883656.4 | 1317968114 | 218200099 | 190989477.4 | 178075071.6 | 230595069 | 206013580.2 | 326075388.3 | 67030940.36 | 124904257.7 | 103736030.1 | 125101358.3 |
| [M-H-H2O]- | 3-cyclopentene-1-octanoic acid, 2-(3-hydroxy-1-penten-1-yl)-5-oxo- | 2.782256283 | 2.114512217 | 0.010052954 | Lipids and lipid-like molecules | 472687000.2 | 319079491 | 373781432.4 | 385646419.3 | 184978342.1 | 414384473.1 | 249189095.5 | 108391140.9 | 575823688 | 427793919.6 | 496629722.8 | 523220443 |
| [M+H]+ | Securinine | 2.251176151 | 1.634763804 | 0.010089976 |  | 454682256.4 | 264304234.8 | 373522799.9 | 349556230.3 | 205613368 | 348297533.3 | 167158537.9 | 327469663.5 | 419546028.9 | 427364150.7 | 444311234.4 | 422892357.9 |
| [M+H]+ | Linolenic acid | 2.103044695 | 2.527904162 | 0.010541787 | Lipids and lipid-like molecules | 201151066.1 | 103075798.6 | 257411402.7 | 49849512.53 | 59574181.5 | 86571589.71 | 75875961.26 | 174626826.4 | 170798108 | 310009279.1 | 225900912.6 | 295981243 |
| [M+Na]+ | Silodosin | 1.073985099 | 2.705870865 | 0.010891089 | Organoheterocyclic compounds | 81842989.95 | 26162369.8 | 37388708.85 | 90259089.77 | 18986286.71 | 28768694.22 | 20477056.42 | 28286916.95 | 45813230.01 | 96995410.2 | 59591316.74 | 58767869.46 |
| [M-H]- | (4e,8e)-10-(4-hydroxy-6-methoxy-7-methyl-3-oxo-1h-2-benzofuran-5-yl)-4,8-dimethyldeca-4,8-dienoic acid | 2.964638268 | 1.93183526 | 0.011157267 | Lipids and lipid-like molecules | 414093165.2 | 407535704 | 533288127.7 | 240567581 | 438883142.9 | 507600802 | 302125131 | 135789657.5 | 607705647.9 | 640114970.5 | 768582596.7 | 658027072.4 |
| [M+H]+ | Vincanidine | 2.226925302 | 1.746660894 | 0.011331522 |  | 347554540 | 226974354.1 | 329813595.9 | 280669356.6 | 145204739 | 290678853.3 | 136355855.1 | 285085917.5 | 393706341.9 | 346638815.5 | 398653848.2 | 358457682.4 |
| [M-H]- | Bilirubin | 1.591808981 | 0.408300834 | 0.011446394 | Organoheterocyclic compounds | 46094824.46 | 24297495.94 | 57044757.86 | 43633705.7 | 137658362.5 | 205720955.5 | 104986038.2 | 130284295.8 | 50510334.32 | 89498200.6 | 50285281.42 | 45969319.18 |
| [M-H-H2O]- | (+)-abscisic acid | 2.692863203 | 2.231166541 | 0.011977203 | Lipids and lipid-like molecules | 332992921.6 | 232286130.5 | 338887201.8 | 238036470.9 | 266720543.5 | 221989430.7 | 306056252.8 | 134231779.7 | 559896907 | 457018579.2 | 694570580.1 | 361263203.2 |
| [M+H-H2O]+ | Betulin | 1.74985801 | 1.783951617 | 0.012889486 | Lipids and lipid-like molecules | 496418917.4 | 184410060.1 | 313954885.6 | 225427192.3 | 146225913 | 156250126.3 | 155017670.7 | 146629205.6 | 211752072.6 | 332448451.3 | 323183039.9 | 210342488.3 |
| [M-H]- | Arachidonic acid (peroxide free) | 2.038725819 | 1.989028924 | 0.012909159 | Lipids and lipid-like molecules | 449769122.1 | 188982347.3 | 331426684 | 300537725.8 | 56250057.14 | 219150026.5 | 129469702.3 | 168061700.4 | 244506119.8 | 265156824.5 | 285340081.7 | 344574271.9 |
| [M-H]- | Anacardic acid | 2.65614031 | 2.87319073 | 0.013758575 | Benzenoids | 247548849.1 | 137894983 | 283565580.8 | 173269382.6 | 23304199.81 | 325699681.8 | 48027046.64 | 120534544.1 | 374871363.5 | 373727454.8 | 407757805.9 | 330707693.3 |
| [M+H-C4H9ON]+ | 1-phenyl-2-decanoylamino-3-morpholino-1-propanol | 1.215113316 | 1.697939006 | 0.01393692 | Organic nitrogen compounds | 110919914.1 | 61118264.42 | 116598355.1 | 92679942.07 | 75182188.8 | 104848266.7 | 53026630.96 | 82010566.88 | 164036250.9 | 138820434.3 | 107581149.5 | 124527823.3 |
| [M-H]- | Acetoxystachybotrydial acetate | 1.112292872 | 0.337595876 | 0.014169026 |  | 26329431.37 | 471378125.1 | 41934348.4 | 34723388.88 | 82578825.69 | 77060207.61 | 49600270.19 | 34184094.31 | 25622671.7 | 24911333.51 | 17638214.71 | 14006515.42 |
| [M+H-NH3]+ | Pentamidine | 2.671990457 | 1.832979643 | 0.015048291 |  | 417255972.2 | 284084098.8 | 424414623.4 | 295544734.5 | 180304491.5 | 425986863.1 | 157208154.8 | 363285046.1 | 553124274.4 | 469107362.3 | 547018004.1 | 496123510.9 |
| [M-H]- | Lpe 18:2 | 1.185612399 | 2.137863162 | 0.015285533 | Lipids and lipid-like molecules | 92757295.94 | 80051708.52 | 59124965.61 | 48172862.12 | 66071099.04 | 54705011.21 | 43044814.48 | 33368453.54 | 103982318.2 | 87385156.71 | 81689307.31 | 148507125.5 |
| [M+H]+ | Pilocarpine | 2.300374059 | 1.790501619 | 0.015907941 |  | 518034088.9 | 228357677 | 395264768.9 | 378544408.8 | 171989531.2 | 375417518.4 | 189738669.7 | 237327891.3 | 496850323.4 | 487624422 | 346913881.7 | 413407950 |
| [M-H]- | (2s,3s)-2-(3,4-dihydroxyphenyl)-3,5,7-trihydroxy-6-methyl-2,3-dihydrochromen-4-one | 1.311541698 | 1.737480793 | 0.016158661 | Phenylpropanoids and polyketides | 197405818.6 | 175941804.7 | 119576946.2 | 217572833.2 | 82442222.83 | 122161155.7 | 105708469.4 | 54183146.08 | 134253597.7 | 168153147.8 | 193251586 | 137644720 |
| [M+H]+ | 4,4'-diaminodiphenylmethane | 1.552365118 | 1.646330675 | 0.017087808 | Benzenoids | 268062774.5 | 119295248.1 | 199466666.7 | 172647649.3 | 96087104.71 | 189479008.5 | 125223476.9 | 138622913.1 | 219950955.4 | 277834906.6 | 186914206.9 | 219814588.3 |
| [M-H]- | Lumichrome | 2.196529454 | 1.784816989 | 0.017175254 | Organoheterocyclic compounds | 707392451.6 | 341191919.6 | 640156153.5 | 733123775.5 | 152037124.1 | 245631596.5 | 239001616 | 291174986.1 | 325543025 | 446405450 | 533597552.6 | 350488067.1 |
| [M+H-H2O]+ | Terbutaline | 1.453364005 | 1.511752203 | 0.017503128 | Benzenoids | 350218424.6 | 149725860.6 | 230081545.2 | 209738245.5 | 120345002.4 | 204063770.6 | 146979331.3 | 148770005.8 | 227195714.7 | 269180699.4 | 190541183.2 | 250607792.3 |
| [M+H]+ | Estra-1,3,5(10),7-tetraene-3,17.beta.-diol | 13.50994313 | 1.726026209 | 0.017861676 | Lipids and lipid-like molecules | 13299604500 | 8493129740 | 12134115115 | 9370962791 | 5943339230 | 12057781506 | 4755160759 | 10792898411 | 15490365759 | 13442432536 | 15504604188 | 13469361331 |
| [M+H]+ | Quinine | 3.566812411 | 1.689824266 | 0.018007497 | Alkaloids and derivatives | 975077617.5 | 663158254.1 | 925812846.8 | 742070105.4 | 430454626.1 | 876480161.6 | 356510870.5 | 782091952.8 | 1067439167 | 975971752.3 | 1085275932 | 1003841948 |
| [M+H]+ | Lenalidomide | 3.585423478 | 1.654937624 | 0.018233144 | Organoheterocyclic compounds | 1141816673 | 574647927.4 | 1073926501 | 811197278.9 | 483004180.6 | 1042295652 | 476203527.8 | 691400167.8 | 1208250844 | 1051632660 | 1078554284 | 1118149580 |
| [M+H-C18H36]+ | Perifosine | 3.014193977 | 1.655879213 | 0.018252897 | Organic acids and derivatives | 1029051078 | 543826140.4 | 685365838.6 | 602336714.4 | 353902936.6 | 738362212.2 | 434070534.7 | 512335339.4 | 739936574.3 | 956150983 | 730291952.6 | 949413458.4 |
| [M-H]- | Cochlioquinone a | 5.16510192 | 0.297223902 | 0.018500767 |  | 408005760.9 | 637246231.3 | 874374413.7 | 281497294.6 | 633748848.4 | 1996115753 | 1505402858 | 1386715722 | 501220543.5 | 286018517.3 | 675583793.3 | 178442534.7 |
| [M+H]+ | D-xylose | 1.052907777 | 2.09122453 | 0.018896572 | Organic oxygen compounds | 43476189.53 | 29082008.31 | 33937796.2 | 62737124.87 | 39992948.89 | 36129645.31 | 40933461.49 | 45793304.85 | 62224418.07 | 69588490.6 | 84658711.03 | 124082957.8 |
| [M+H]+ | Gln-pro | 2.045622855 | 1.539797651 | 0.019411632 | Organic acids and derivatives | 501931398.4 | 278324272.1 | 433420735 | 332095439.4 | 218919939.9 | 421115926.1 | 247467416.8 | 246320641.7 | 426794050 | 477121611.1 | 418929872.6 | 423013882.2 |
| [M+H-C6H12O]+ | Isopropalin | 3.981354432 | 1.618241771 | 0.019455784 | Benzenoids | 1643203620 | 878399242.8 | 1227454733 | 1250711029 | 588693233.9 | 1310585591 | 663539060.6 | 1022351962 | 1412228962 | 1602100662 | 1374438078 | 1412903901 |
| [M+H]+ | Anisomycin | 1.695882168 | 1.738436421 | 0.019660748 | Benzenoids | 243956090.2 | 119652591.1 | 203706069.6 | 173609045.5 | 107161958.6 | 186440631.6 | 78027987.26 | 178199674.4 | 287001665.3 | 238227316.9 | 230724109.1 | 199891843.8 |
| [M-H]- | Gln-met | 2.793591415 | 7.457426944 | 0.019765479 | Organic acids and derivatives | 159814424.9 | 102756340.1 | 259504242.8 | 126637385.6 | 43641254.59 | 84292718.97 | 33090041.29 | 14906156.12 | 558377815.8 | 125223995.5 | 325789561 | 302595025 |
| [M-H]- | 3,5-dinitrosalicylate | 2.816814978 | 1.636197635 | 0.019930027 | Benzenoids | 628274105.4 | 372468633.9 | 437863033.2 | 384426228.2 | 348607172.4 | 624437216.4 | 341246137 | 431264775.9 | 818246807.2 | 586480198.3 | 809159579.3 | 642186870.8 |
| [M+H]+ | 1-butanone, 1-(2,3-dihydro-1h-inden-5-yl)-2-(ethylamino)- | 1.18150523 | 2.201466575 | 0.020501789 | Benzenoids | 64881733.77 | 42022394.05 | 63356829.43 | 76189238.66 | 23776764.63 | 73628575.79 | 26248152.85 | 31573079.27 | 88077618.68 | 58993749.59 | 93032244.84 | 101622497.8 |
| [M+H]+ | Trachelanthine | 3.111794507 | 1.946381344 | 0.0205726 |  | 782325392 | 568926846.4 | 634626237.6 | 798430123.4 | 221501578 | 605686470.5 | 326501068.4 | 268198054.8 | 609705591.1 | 553306642.7 | 794004406.9 | 810518023.7 |
| [M-H]- | Limaprost | 1.447465713 | 0.488331297 | 0.020774864 | Lipids and lipid-like molecules | 1735960580 | 25945690.26 | 9303807.306 | 45874399.22 | 151480490.4 | 162147159.9 | 130930849.5 | 95928516.81 | 55061875.85 | 88526045.42 | 23614531.94 | 96734272.9 |
| [M+H-H2O]+ | Muramic acid | 1.742763105 | 1.970533081 | 0.021378017 | Organic oxygen compounds | 335724888.4 | 103623201.4 | 241184334.2 | 293943174.1 | 89815894.28 | 185354897.2 | 172422261.3 | 55033892.7 | 179865582.7 | 274551233.7 | 284090004.5 | 251936202.7 |
| [M-H]- | Zanamivir | 2.138911998 | 1.770257581 | 0.02140546 | Organic acids and derivatives | 641278475.4 | 583424571 | 687248807.7 | 408114513.1 | 287892391.1 | 304260227.2 | 253721776.6 | 117432270 | 317820847.8 | 402314824.1 | 476728009.9 | 508437244.7 |
| [M-H]- | Glabridin | 1.496971565 | 1.973986701 | 0.02267482 | Phenylpropanoids and polyketides | 123263848.4 | 107751871.2 | 135405506.4 | 168310606.6 | 57830403.35 | 121219897.5 | 116934174.1 | 64811471.13 | 148861524.8 | 248214754.9 | 155413243.8 | 159716875.7 |
| [M-H]- | 1-myristoyl-2-hydroxy-sn-glycero-3-phosphoethanolamine | 2.140468408 | 2.388724411 | 0.022801787 | Lipids and lipid-like molecules | 247322547.1 | 166967210.2 | 146503696.6 | 178333401.7 | 121151868.2 | 162460919 | 177021678.8 | 60722380.1 | 246585570.6 | 211673353.7 | 335455263.9 | 451663637 |
| [M-H]- | Glimepiride | 4.205933276 | 4.685883455 | 0.02307729 | Benzenoids | 292134277.7 | 176157284.1 | 908907447.8 | 451860058 | 165332691.6 | 303213709.2 | 189581639.5 | 143830930.2 | 220702698.5 | 1103584048 | 1238600304 | 1194999220 |
| [M+Na]+ | 2'-deoxyinosine | 2.094615946 | 2.513533323 | 0.023285997 | Nucleosides, nucleotides, and analogues | 195975906 | 78063172.38 | 123746785.8 | 304746568.2 | 60657815.82 | 52944858.19 | 117687320.5 | 176780395.5 | 213643566.9 | 357219547.7 | 165506600.9 | 289328807.8 |
| [M-H]- | Ascorbyl stearate | 5.662857948 | 0.189592415 | 0.024042278 | Lipids and lipid-like molecules | 1308849408 | 985791676.3 | 1064884784 | 699567834.5 | 1586565824 | 2234702887 | 853200836.6 | 675514609.4 | 272729310.5 | 295917306.2 | 113695272 | 331974525.4 |
| [M-H]- | Tetrahydroalstonine | 1.993734295 | 3.14365508 | 0.024070514 |  | 82516335.79 | 122670871.8 | 86332874.8 | 276413522.1 | 86687405.01 | 69008594.96 | 80344867.33 | 37981106.3 | 227016409.5 | 337072060.9 | 190774121.5 | 106567977.4 |
| [M+H]+ | Leu-Phe | 1.595681215 | 1.88609582 | 0.024831501 | Organic acids and derivatives | 129347257.4 | 38408806.88 | 176663529 | 138837913.3 | 54539508.66 | 111122659.2 | 114103142.1 | 142028885.3 | 212636603.4 | 162445778.5 | 155301142.8 | 265160744 |
| [M-H]- | Pyrrole-2-carboxylic acid | 1.76809639 | 1.931785722 | 0.025298442 | Organoheterocyclic compounds | 171148103 | 111024705 | 96020909.77 | 120498708.4 | 52358019.97 | 116985310 | 129725977.4 | 183807643.2 | 313114247.8 | 207325496.3 | 212159198 | 200215856.6 |
| [M-H]- | Hypoxanthine | 6.970173974 | 2.242278608 | 0.025623153 | Organoheterocyclic compounds | 3244392870 | 2107205571 | 2132799194 | 3463536872 | 1782981217 | 1953285416 | 1949176385 | 750622649.8 | 3646286131 | 3187482159 | 2339738504 | 5257945577 |
| [M-H]- | 1-hydroxy-4-[(2s,3r,4s,5s,6r)-3,4,5-trihydroxy-6-(hydroxymethyl)oxan-2-yl]oxynaphthalene-2-carboxylic acid | 1.643165398 | 2.039017856 | 0.026047658 | Organic oxygen compounds | 36517570.46 | 83250022.72 | 55054907.42 | 42198294.75 | 140033820.6 | 155328356.7 | 84296586.44 | 19009739.3 | 207815351.2 | 184010954.4 | 172117680.5 | 248948210.5 |
| [M-H]- | 11.alpha.-hydroxyprogesterone .beta.-d-glucuronide | 1.883632132 | 1.6296045 | 0.026590333 | Lipids and lipid-like molecules | 385135538 | 397731037 | 415336156 | 339150890.1 | 195027628.5 | 319138186.9 | 250998297.9 | 127485531.9 | 302626077 | 416647980 | 392836980.7 | 342554841.3 |
| [M-H]- | Uridine | 7.653898453 | 2.115111566 | 0.026604506 | Nucleosides, nucleotides, and analogues | 3080993871 | 984758283.2 | 1492073514 | 2225813674 | 659939015.6 | 1740756242 | 1598963023 | 3203769836 | 5119360320 | 3261159580 | 3586665851 | 3268868369 |
| [M+H-H2O]+ | Androstan-3-ol-17-one 3-glucuronide | 2.308554951 | 3.270459158 | 0.026847641 | Lipids and lipid-like molecules | 538008592 | 34374531.06 | 89334465.6 | 333655383 | 54263316.86 | 103821765.7 | 159460590.2 | 55203150.18 | 255500443.4 | 181784824.5 | 499106543.5 | 282667990.3 |
| [M-H]- | 1-o-(9z-octadecenyl)-sn-glycero-2,3-cyclic-phosphate | 2.303068302 | 0.527842361 | 0.026871192 |  | 177862629 | 138943469 | 423037866.2 | 320482752.6 | 374816197 | 440307878.4 | 269039244.5 | 495327207.1 | 142114308.1 | 146355706.7 | 318823848.5 | 226428146.4 |
| [M+Na]+ | Ethyl 3-indoleacetate | 1.003968699 | 0.528223589 | 0.027018957 | Organoheterocyclic compounds | 2925073.353 | 44388280.91 | 19272236.19 | 8834110.484 | 69331507.41 | 99271853.5 | 59030199.75 | 47842017.24 | 32536646.17 | 36066878.52 | 36206762.96 | 40702410.73 |
| [M+H]+ | D-glucosaminic acid | 11.88815798 | 1.614666534 | 0.027330738 | Organic acids and derivatives | 14336431372 | 7376104060 | 10393937277 | 10412386641 | 5201296845 | 12082509055 | 7679366355 | 9508234838 | 12644962047 | 15835999413 | 11448319778 | 15730546177 |
| [M-H]- | Trans-3'-hydroxycotinine o-.beta.-d-glucuronide | 1.821052817 | 4.964370087 | 0.027879367 | Organic oxygen compounds | 108889991.5 | 259957312.7 | 114450942 | 192172142.6 | 20710501.75 | 40350496.29 | 26669341.48 | 34915580.02 | 126431256.9 | 49945928.73 | 245859942.6 | 186622606 |
| [M+H-2H2O]+ | Erythrodiol | 1.037587038 | 1.527688319 | 0.027974291 | Lipids and lipid-like molecules | 157588460.9 | 67734533.19 | 123798720 | 103960995.6 | 76290795.45 | 85916197.24 | 95804725.25 | 82312791.58 | 92312561.3 | 165005623.8 | 136857882 | 125733710.8 |
| [2M-H]- | 3-phosphonoalanine | 7.040264384 | 8.718070582 | 0.028732237 | Organic acids and derivatives | 1698840495 | 1622339266 | 1246390226 | 1208218924 | 207496191.4 | 273000049.4 | 443068866.5 | 82733277.97 | 1192817560 | 891883330.4 | 3581562553 | 3106716905 |
| [M+H]+ | Lsd | 1.041658539 | 1.84147191 | 0.028779909 | Alkaloids and derivatives | 80019737.16 | 64705532.39 | 65006480.61 | 89271353.36 | 39991163.16 | 74223509.94 | 47703540.27 | 26643336.51 | 86258448.14 | 65157482.67 | 83997143.62 | 111817722.9 |
| [M-H-C4H8]- | Neobavaisoflavone | 1.271823777 | 1.686608266 | 0.029840117 | Phenylpropanoids and polyketides | 185052085.5 | 126612054.8 | 109182413.7 | 181178985.9 | 85601482.54 | 129393458.3 | 62259987.05 | 81182002.17 | 121448043.7 | 129017853.2 | 160764889.8 | 193311902.3 |
| [M-H]- | Sarcosine | 1.752071858 | 1.695958482 | 0.030564412 | Organic acids and derivatives | 367583216.2 | 206776334.5 | 164893264.4 | 386832933.7 | 114722749.9 | 142292167.6 | 150082882.7 | 238131386.6 | 216935840.2 | 257290380 | 263133085.7 | 356922606 |
| [M+H]+ | Coenzyme q2 | 1.487152111 | 2.06107339 | 0.030614222 | Lipids and lipid-like molecules | 86325797.67 | 72677682.57 | 129978677.7 | 72479637.79 | 92776126.97 | 133741037.5 | 49418327.47 | 49610100.53 | 236935222.8 | 143380321.5 | 148232604.3 | 142425209.3 |
| [M+H-2H2O]+ | .beta.-muricholic acid | 2.725383512 | 0.599805696 | 0.03113535 | Lipids and lipid-like molecules | 191015051.6 | 419478626.8 | 382137943.8 | 159258185.9 | 526486409.9 | 765383596.1 | 715582034.6 | 591188402.2 | 378238220 | 601125363.1 | 256244215.5 | 323071540.7 |
| [M-H-C2H3O]- | 7-hydroxy-2-acetylaminofluorene | 4.650645818 | 1.528391574 | 0.031265869 |  | 2141495306 | 1720272499 | 2696947083 | 2802192816 | 1321314919 | 2216924779 | 1575619697 | 923821153.4 | 2340713619 | 2054594252 | 2457318765 | 2375313444 |
| [2M+Na]+ | Chenodeoxycholic acid | 1.550760361 | 0.494957089 | 0.032098955 | Lipids and lipid-like molecules | 194403620 | 278472642.5 | 282502282.3 | 104307604.2 | 111158491.2 | 204871133.5 | 159986182.3 | 221297773.9 | 79923540.04 | 139213952.9 | 82946829.09 | 43055978.21 |
| [M-H]- | Glutaric acid | 1.970403351 | 1.619722891 | 0.032228397 | Organic acids and derivatives | 312593109.2 | 206878339.6 | 331367511.9 | 351326188.3 | 119149558.1 | 220960109.1 | 152914244 | 310565365.9 | 304362042.2 | 302396935.1 | 367499039.2 | 327333930.8 |
| [M+H]+ | Nordihydroguaiaretic acid | 1.176106975 | 2.006370659 | 0.03322137 |  | 55116153.86 | 38373957.93 | 77237004.59 | 45651677.41 | 47050827.35 | 66318498.32 | 27539240.72 | 72010060.47 | 156186511.1 | 97345463.63 | 86801824.43 | 86859886.4 |
| [M+H]+ | Sempervirine | 1.118349573 | 1.620322404 | 0.035187866 | Organoheterocyclic compounds | 79290550.33 | 56014043.76 | 98640272.21 | 121282358.5 | 66246373.17 | 101644219 | 38860528.92 | 95060136.73 | 149236846.9 | 115962660.1 | 104547620.6 | 119284415.2 |
| [M-H]- | Phe(benzoyl)-leu-arg | 2.772226715 | 0.315400513 | 0.035610803 | Organic acids and derivatives | 45633928.48 | 608972529 | 436885801.5 | 80183255.1 | 501702991.6 | 585347616 | 209764528 | 398577621.5 | 33861095.84 | 326005040.6 | 153860835.8 | 21000772.36 |
| [M-H-CO2]- | 2,5-furandicarboxylic acid | 1.77903513 | 2.180771797 | 0.036411455 | Organoheterocyclic compounds | 120941408.1 | 67177487.32 | 140367851.6 | 107481286.8 | 63519052.1 | 94894212.63 | 100992476.3 | 155036602.2 | 347309001.4 | 171576135.8 | 173803624.4 | 211115411.9 |
| [M+H]+ | Schweinfurthin e | 4.299112042 | 0.320083991 | 0.037090455 | Organoheterocyclic compounds | 185302421.1 | 3832134391 | 1016533572 | 258501915.5 | 1252570142 | 684725094.6 | 1108892222 | 362143593.2 | 125764487.1 | 488467005.7 | 256148064.3 | 220572648.3 |
| [M-H]- | 2-o-methylguanosine | 1.351186538 | 0.303024763 | 0.037148194 |  | 18602749.3 | 109222297.9 | 117367999.6 | 17075414.1 | 137105229.8 | 120422352.3 | 55659935.33 | 51767725.89 | 22516627.65 | 54213711.92 | 23193253.54 | 10666882.97 |
| [M+H]+ | Sipeimine | 1.001121179 | 1.716226503 | 0.037618664 | Lipids and lipid-like molecules | 59878228.3 | 53370633.63 | 77643672.19 | 62686076.14 | 55325346.18 | 62828530.31 | 30307563.07 | 65028431.32 | 116069522.9 | 59627909.06 | 89364803.32 | 101334739.1 |
| [M+H-C6H16O8]+ | Ginsenoside f1 | 1.729156815 | 1.768901734 | 0.037958118 | Lipids and lipid-like molecules | 472771039.6 | 258346354.8 | 305650015.8 | 232998777.6 | 147609283.9 | 195281642 | 181783486.3 | 170898718.9 | 194857130.2 | 342633358.1 | 425003556.4 | 267906473.2 |
| [M-H-H2O]- | 2.alpha.-mannobiose | 1.420929755 | 0.451364442 | 0.038216465 | Organic oxygen compounds | 80764838.87 | 56198507.86 | 145560084.1 | 36078467.18 | 65663915.55 | 150664389.4 | 153341986.6 | 188867869.3 | 46084629.25 | 94455365.95 | 71140188.1 | 40424081.87 |
| [M+H]+ | Prosulfocarb | 1.226110058 | 1.505994234 | 0.0387902 |  | 148610890.9 | 97030974.86 | 139217732.4 | 127976729.3 | 86139474.62 | 159807990.9 | 69286948.94 | 120334166.9 | 180760877 | 163135290 | 159778198.6 | 152289406.1 |
| [M-H]- | Nicotinate | 2.988100255 | 1.71860672 | 0.040135299 | Organoheterocyclic compounds | 1426145884 | 925712949.1 | 751403582.1 | 1205573573 | 569058720.4 | 600710137.1 | 534893818.9 | 470339667.4 | 1089388586 | 757154947.5 | 626365846.3 | 1265064265 |
| [M+H-H2O]+ | 19-hydroxyandrost-4-ene-3,17-dione | 1.33861729 | 1.624614576 | 0.040613677 | Lipids and lipid-like molecules | 144641144.1 | 94289463.67 | 131881101.8 | 112608835.7 | 98103580.94 | 158557096.6 | 74894485.73 | 108987253.6 | 177583444.9 | 154320712 | 235020958.6 | 148786516.4 |
| [M+H]+ | 3,3'-dimethoxybenzidine | 1.933908974 | 1.593099046 | 0.042777817 | Benzenoids | 375922454.2 | 239205765.4 | 324276653.3 | 280271304.3 | 182511979.7 | 353432512.1 | 134074097 | 258937764.5 | 442499753.3 | 339477243.7 | 342037588.8 | 355904894.5 |
| [M+H-H2O]+ | Naloxone | 1.1330459 | 1.869520803 | 0.043241473 |  | 145444503.7 | 51922154.25 | 119999439.2 | 88605757.23 | 48079867.32 | 76086243 | 54848514.76 | 38622443.38 | 105614851.6 | 53369232.89 | 121083692.5 | 126809250.1 |
| [M+H]+ | Cis-4,10,13,16-docosatetraenoic acid methyl ester | 1.106234412 | 1.59092276 | 0.043294359 | Lipids and lipid-like molecules | 251268056.5 | 206194506.1 | 246692290.8 | 188382888.3 | 71889129.9 | 90682450.65 | 138904494.6 | 75940268.05 | 147643153.6 | 177320717.7 | 107511403 | 167964975.9 |
| [M+2H]2+ | Lopinavir | 1.050300789 | 0.358761756 | 0.043822029 | Organic acids and derivatives | 32118050.26 | 25897664.02 | 20231170.27 | 13848649.8 | 77749949.95 | 71700419.54 | 51259173.03 | 20032053.99 | 6373818.496 | 19193113.45 | 28945958.8 | 24680752.13 |
| [M-H-H2O]- | N-[tris(hydroxymethyl)methyl]-3-amino-2-hydroxypropanesulfonic acid | 2.681673326 | 0.425112182 | 0.043827003 | Organic acids and derivatives | 84303946.34 | 259631091.2 | 249452711.2 | 143207853.5 | 213232931.3 | 693682269.3 | 438367828.5 | 552430435 | 188715172.7 | 178829888.6 | 139331128.6 | 299864921.4 |
| [M+H]+ | 6,7-dimethylesculetin | 1.019373958 | 0.555197247 | 0.044016245 |  | 31701684.63 | 103486801.5 | 97798047.97 | 30225755.22 | 91416376.7 | 66457038.71 | 106900334.6 | 53807211.82 | 55346864.68 | 53368990.93 | 43985998.29 | 24173418.97 |
| [M+H]+ | Pymetrozin | 1.057654565 | 0.457561845 | 0.044304216 | Organoheterocyclic compounds | 49832155.05 | 29472247.62 | 45053115.09 | 52675639.31 | 73699142.55 | 65686828.58 | 54280791.11 | 108462527.9 | 1656378.016 | 44512678.27 | 51042184.18 | 41031594.79 |
| [M+H]+ | N,n-dimethylformamide | 1.284172991 | 2.06551963 | 0.044464274 | Organic acids and derivatives | 55459285.59 | 300125267.9 | 104371690.8 | 53203496.06 | 74329147.81 | 84440137.76 | 89354779.17 | 12678894.86 | 104335076.4 | 97003723.23 | 151716374.6 | 185638458.5 |
| [M-H]- | Indole-3-pyruvic acid | 1.310371373 | 1.507818033 | 0.044638771 | Organoheterocyclic compounds | 474175866.5 | 259109974.2 | 274539892.9 | 204264674.3 | 126038177.7 | 173329550.5 | 134636367.3 | 148048596.8 | 243587350.5 | 211437965.8 | 275231376 | 147372853.6 |
| [M-H]- | Genistein | 1.626033389 | 2.324900744 | 0.045774315 | Phenylpropanoids and polyketides | 83400832.48 | 58570851.8 | 46811085.79 | 255086290.2 | 33633314.51 | 59658349.85 | 50175304.76 | 152055630.6 | 102326891.1 | 160070784.7 | 239485327.6 | 185177708.4 |
| [M-H]- | 4-hydroxyquinoline | 1.570007256 | 0.242620652 | 0.045888694 | Organoheterocyclic compounds | 89889207.91 | 23590779.25 | 89270202.3 | 83185009.13 | 128710341.5 | 76983382.53 | 58203694.21 | 221194959.8 | 26788283.74 | 24696228.81 | 29191697.32 | 37017219.11 |
| [M-H]- | His-Met | 2.400152388 | 1.648513601 | 0.046304772 | Organic acids and derivatives | 467036831.4 | 287224573.2 | 462518866.1 | 662819326.8 | 368077852.3 | 496898531.8 | 330280385.1 | 152992869.3 | 592814109.2 | 422117043.7 | 663780166.2 | 543896547.1 |
| [M-H-C12H16O2N]- | Dauricine | 4.021660569 | 1.663230631 | 0.048628338 | Organoheterocyclic compounds | 1670280989 | 1030534145 | 1098813483 | 1151446915 | 928408240 | 1456331212 | 1164478570 | 326680766.3 | 1528239417 | 1610017629 | 1892414031 | 1415842509 |
| [M-H]- | 1h-pyrrole-3-propanoic acid, 5-[(1,2-dihydro-2-oxo-3h-indol-3-ylidene)methyl]-2,4-dimethyl- | 4.949760308 | 0.163449228 | 0.049444601 | Organoheterocyclic compounds | 20232875.93 | 877997751.2 | 188797794.7 | 20847321.24 | 1871775758 | 1745983667 | 164122703.5 | 1055069170 | 112546171.4 | 576704396.4 | 51361499.8 | 49983886.45 |
|  |  | 2.478193283 | 12.97981912 | 4.12864E-05 |  | 197320526.9 | 31145583.42 | 55297653.65 | 18640855.4 | 10239755.02 | 29294489.75 | 11204077.76 | 13935925.54 | 181666246.5 | 246039707.6 | 234397535.2 | 177356552.8 |
|  |  | 1.353541175 | 2.534637855 | 7.07686E-05 |  | 64981735.51 | 85668512.46 | 56933048.46 | 57666353.5 | 33139401.23 | 39471041.63 | 53653123.98 | 30513325.91 | 95021669.67 | 100856170 | 93009601.32 | 108485206.2 |
|  |  | 2.036258314 | 3.419588125 | 7.12154E-05 |  | 157910704.9 | 54263060.56 | 53726135.45 | 132428680.5 | 68002487.49 | 65679548.16 | 34907728.06 | 52505882.22 | 158487734 | 191132055.8 | 191482996.5 | 214953259 |
|  |  | 2.352868768 | 2.944091211 | 7.251E-05 |  | 113280835.2 | 187701824.3 | 68653824.67 | 311447840.3 | 115833845.7 | 112342796.1 | 71707683.62 | 68285559.9 | 290692289.8 | 271445912.1 | 232524084.9 | 289263436.4 |
|  |  | 1.211217595 | 3.841739506 | 0.000111996 |  | 39738051.18 | 27852502.79 | 45901990.53 | 32372362.71 | 10519242.3 | 27419745.47 | 10354965.36 | 16847996.02 | 57411161.69 | 69432846.51 | 56394946.12 | 67019445.19 |
|  |  | 1.41554663 | 2.369455868 | 0.000126739 |  | 166028164.8 | 113993032.6 | 99465578.63 | 101258248.3 | 52988023.58 | 56363170.21 | 39387165.46 | 41909009.75 | 104437016.2 | 127211739.4 | 119415277.1 | 100666494.5 |
|  |  | 4.198817334 | 8.326127941 | 0.000131774 |  | 603592110 | 105161173.9 | 165460952.9 | 67205282.08 | 31451004.8 | 97463850.84 | 33340751.07 | 141851542.1 | 531431454.8 | 761855633.6 | 702704406.7 | 536043533.4 |
|  |  | 1.630968332 | 2.165598237 | 0.000133642 |  | 63792748.55 | 66628616.97 | 56084320.69 | 70251135.95 | 102256928.8 | 64297202.1 | 84446848.01 | 61186832.42 | 182201403.3 | 165922016.2 | 164006041.2 | 163943913.3 |
|  |  | 1.372174662 | 2.486389602 | 0.000143345 |  | 39477883.6 | 41911852.23 | 33075354.92 | 39008940.47 | 51480223.03 | 36404146.37 | 47304143.27 | 33307602.66 | 116487886.7 | 106484930.3 | 88210013.23 | 107764158.8 |
|  |  | 1.863380681 | 0.182245499 | 0.000192565 |  | 33626959.23 | 31458989.26 | 19700393.58 | 22364926.36 | 157744205.5 | 132342452.2 | 96895554.52 | 150264223.4 | 24755726.95 | 26784763.76 | 24003316.26 | 22366937.97 |
|  |  | 3.529815125 | 0.014369098 | 0.000214086 |  | 11901994.9 | 497135398.1 | 3113001.289 | 11611314.22 | 322316858.6 | 532285108 | 331298087.5 | 379047440.4 | 5897014.068 | 4867053.734 | 3522760.957 | 8200054.863 |
|  |  | 7.133276674 | 18.74415724 | 0.000215962 |  | 24383094.96 | 216789641.9 | 137843022.7 | 37202642.99 | 84239360.72 | 162011638.4 | 69454219.42 | 54556120.73 | 1266379898 | 2191696649 | 1943204340 | 1538955876 |
|  |  | 1.534509412 | 1.952056031 | 0.000231191 |  | 150713074.9 | 84821183.36 | 125722075.2 | 120077858.5 | 73295595.13 | 107248632.6 | 64824625.91 | 73194488.94 | 153762779.6 | 161421011 | 156323041.8 | 150346661.8 |
|  |  | 1.647525365 | 2.112207143 | 0.000269677 |  | 73956472.73 | 71024908.56 | 52367462.46 | 69650925.63 | 103488946.3 | 69139893.37 | 96700165.44 | 67034596.62 | 195483471.6 | 176613781 | 156857090.8 | 181515259 |
|  |  | 1.204145158 | 1.964366886 | 0.000358479 |  | 103129874.4 | 38157209.95 | 56133738.35 | 64556581.85 | 52415808.31 | 54347373.91 | 41483983.55 | 49859317.49 | 105015919 | 109870402 | 88687618.79 | 85579875.85 |
|  |  | 2.764856627 | 6.432602649 | 0.000379659 |  | 280861269 | 57688120.36 | 101476079.9 | 45760457.71 | 34410810.95 | 67181152.52 | 37080321.37 | 45666579.29 | 211843184 | 349304245.9 | 354904220.3 | 269727015.5 |
|  |  | 1.649043176 | 0.095258758 | 0.000384835 |  | 18362462.76 | 7530343.45 | 3764914.183 | 7166527.903 | 119671866.2 | 103806083.2 | 83496898.89 | 66082323.43 | 11836506.43 | 4292132.54 | 11875819.12 | 7532504.606 |
|  |  | 2.012824865 | 2.696217722 | 0.000389649 |  | 56883475.73 | 203282447.6 | 73493333.45 | 263670854.8 | 37564200.04 | 92406391.93 | 114227230.2 | 72302825.28 | 203547447.3 | 210180307.7 | 241099134.1 | 198527765.8 |
|  |  | 1.969458924 | 2.392965999 | 0.000417607 |  | 218639389.5 | 120307000.9 | 159012053.1 | 155631569 | 85774180.01 | 82072980.04 | 115564396.3 | 92746174.74 | 181795744.7 | 243761121.5 | 259464848.4 | 215110946.3 |
|  |  | 2.966051919 | 0.32622502 | 0.000419298 |  | 90071826.26 | 656088021.5 | 284592284.8 | 89115287.3 | 400786181.9 | 468856535.8 | 422866621.2 | 305519814.2 | 140674051.5 | 169661692.7 | 88149111.96 | 122832236.7 |
|  |  | 1.986219345 | 1.965011717 | 0.00043044 |  | 273001450.6 | 192817277.2 | 241056743.7 | 219583497.1 | 111638920.9 | 177864143.1 | 104271578.1 | 129897178.8 | 275907385.5 | 248760486.6 | 242847711.7 | 261505679.8 |
|  |  | 1.37421662 | 5.776156868 | 0.000442468 |  | 16486899.17 | 13137128.39 | 12741806.74 | 10465873.54 | 16840383.12 | 22777358.56 | 10297714.75 | 2178851.841 | 61502819.65 | 87229937.62 | 90128218.57 | 62043920.66 |
|  |  | 1.326172458 | 0.216542733 | 0.000446441 |  | 4302197.246 | 16304311.08 | 4589304.333 | 8939440.622 | 71156937.7 | 60997904.9 | 70849273.49 | 73328503.01 | 16231044.63 | 35118534.59 | 3008680.733 | 5479560.504 |
|  |  | 1.067890786 | 1.686709866 | 0.000468747 |  | 90733584.56 | 53659550.1 | 62844423.41 | 62804127.12 | 46182974.58 | 64799813.45 | 43880775.27 | 57599700.38 | 89066962.07 | 95345666.74 | 87759657.2 | 86191596.94 |
|  |  | 2.095316754 | 1.94130743 | 0.000471538 |  | 508440189.8 | 192062584.5 | 357239051.8 | 270282793.5 | 145843453 | 118679925.1 | 176494610.9 | 189233820.7 | 301278880 | 282051134.9 | 348390363.9 | 291792142.4 |
|  |  | 1.000943816 | 2.803323513 | 0.000498786 |  | 21582079.41 | 35229235.95 | 14147713.28 | 58890074.14 | 22611356.29 | 23194570.65 | 16430546.4 | 11732377.61 | 60331170.06 | 47688157.4 | 42544262.38 | 56795029.24 |
|  |  | 1.565776549 | 3.223638522 | 0.00054993 |  | 44945114.48 | 26746605.19 | 37930936.93 | 45613710.8 | 28599465.25 | 39492998.03 | 18580655.03 | 53196004.33 | 99142531.32 | 95612840.21 | 124811178.3 | 131320942 |
|  |  | 1.365030588 | 2.137047094 | 0.00056548 |  | 97007730.82 | 58395453.49 | 85470889.24 | 80410333.7 | 42498278.29 | 79676709.52 | 44409322.4 | 44213536.15 | 113982535.3 | 111199826.2 | 113733022.9 | 111569540.6 |
|  |  | 1.789475532 | 11.50729916 | 0.00057572 |  | 105494323.8 | 20154561.37 | 27433324.03 | 8732210.095 | 3251945.204 | 17717996.12 | 8119737.895 | 10589803.71 | 83142722.8 | 156821557.8 | 111646205 | 104993195.1 |
|  |  | 1.361906398 | 2.38896015 | 0.000621838 |  | 72544044.32 | 67431336.54 | 80247025.3 | 81243140.69 | 43285076.96 | 61289299.38 | 52113649.72 | 20294369.16 | 112044705.6 | 96515352.83 | 104770334.1 | 109473497 |
|  |  | 1.367795394 | 1.847474175 | 0.000644214 |  | 145116716.9 | 92810542.07 | 103801143.1 | 113977713.9 | 74397336.88 | 98360940.39 | 69202305.67 | 53298287.5 | 134798681.9 | 136486267.2 | 143072598 | 131125590.9 |
|  |  | 1.038892868 | 1.891217388 | 0.000693386 |  | 46567942.11 | 29586908.6 | 27703557.99 | 37478699.69 | 38925948.14 | 41007482.34 | 39800566.22 | 42300165.55 | 68049601.53 | 86585422.44 | 65823810.16 | 85982990.96 |
|  |  | 1.012560295 | 3.530962485 | 0.000736182 |  | 12602004.95 | 17689586.95 | 42269433.03 | 18912643.9 | 19859382.9 | 17689771.32 | 6268521.531 | 9812540.756 | 49506301.62 | 57245329.83 | 36457103.46 | 46157547.65 |
|  |  | 2.601425797 | 4.281365222 | 0.000788734 |  | 262314581.1 | 215742088.8 | 327259352.6 | 368793541.7 | 60459170.65 | 85924642.43 | 51340413.46 | 77428303.25 | 191901285.2 | 347854985.4 | 304897142.8 | 333375058.5 |
|  |  | 2.338203818 | 0.46976336 | 0.000847168 |  | 351347122.1 | 587096877.1 | 340907393.8 | 172077816.9 | 297801771.7 | 314220058.4 | 294984782.8 | 376434787.6 | 124999623.6 | 122316764.9 | 208745853.4 | 146851503.4 |
|  |  | 1.127475962 | 0.242358048 | 0.00085491 |  | 19829428.34 | 22083591.82 | 13477101.59 | 13241985.32 | 62733543.06 | 51776500.05 | 49712534.32 | 43050171.96 | 11381660.46 | 25427317.67 | 1471189.37 | 11954051.41 |
|  |  | 1.021199935 | 2.098044811 | 0.000903935 |  | 68087857.82 | 60667680.54 | 71692095.13 | 44297152.22 | 38105141.56 | 36900241.95 | 40493121.66 | 16357971.45 | 63521254.17 | 70490210.6 | 69212008.75 | 73417323.07 |
|  |  | 1.78419088 | 0.271268032 | 0.000910006 |  | 436854.9072 | 89183349.54 | 4530961.297 | 32648988.02 | 151566576.9 | 143467209.6 | 106272708.9 | 150125992.7 | 34242665.49 | 72437448.81 | 12052286.02 | 30853605.3 |
|  |  | 1.732960443 | 1.981149482 | 0.000993393 |  | 178939501.9 | 117972040.7 | 125975927.6 | 141267133.1 | 96812725.25 | 132232544.6 | 77024308.6 | 99478368.74 | 216246341.7 | 226421833.6 | 176023817.5 | 184759112.5 |
|  |  | 1.32312119 | 2.726083481 | 0.00102914 |  | 71160805.63 | 47671895.37 | 59153610.54 | 96799148.75 | 11328122.12 | 38294224.25 | 36971853.15 | 42788295.8 | 99431192.84 | 70709153.75 | 93075685.6 | 89491451.11 |
|  |  | 1.07450302 | 1.895801255 | 0.001040808 |  | 74218607.48 | 59592228.66 | 66152086.05 | 74471158.08 | 42153686.89 | 59780551.57 | 35098312.88 | 34577445.4 | 89557547.3 | 78410646.11 | 79397026.23 | 77973227.53 |
|  |  | 1.818430317 | 2.041727579 | 0.001120605 |  | 327096803.7 | 102569659.8 | 245849658.3 | 127928113.2 | 140694302.9 | 93822758.25 | 141015636.6 | 89259572.25 | 218426875.5 | 205978619.7 | 270780987.4 | 253792713.6 |
|  |  | 1.759571046 | 3.435007828 | 0.001127789 |  | 62923197.87 | 77607704.85 | 61906931.67 | 80060245.87 | 60909685.29 | 60876074.61 | 21421750.5 | 28058249.02 | 159921564.3 | 160572088.9 | 103708593.1 | 164096978.1 |
|  |  | 1.145192222 | 2.842036256 | 0.001184938 |  | 41807079.51 | 19137610.38 | 27996530.9 | 30497187.52 | 32470477.06 | 9999776.992 | 31387846.32 | 22863231.43 | 67578404.56 | 80009417.57 | 73941096.78 | 53356612.83 |
|  |  | 1.298690736 | 6.585616432 | 0.001191908 |  | 67199212.34 | 66861221.69 | 60375208.25 | 63013130.85 | 4240576.084 | 11149176.6 | 18755937.94 | 6890721.11 | 45701746.63 | 59877156.15 | 89155351.01 | 75515813.66 |
|  |  | 1.888479034 | 2.032153806 | 0.00124972 |  | 265054926.8 | 125843209.2 | 261700393.3 | 253734149.4 | 84072470.34 | 151432414 | 110405492.9 | 105267965.6 | 211126165.5 | 197268023.5 | 256463603.2 | 252005994.2 |
|  |  | 1.254878932 | 2.358042233 | 0.001317215 |  | 73787216.7 | 58734215.96 | 83375255.99 | 55954167.67 | 28013451.34 | 62858876.31 | 24900430.82 | 33168248.86 | 89080904.58 | 82056617.69 | 88333973.21 | 91737690.07 |
|  |  | 2.016187768 | 0.627398606 | 0.001486798 |  | 188959954.7 | 200092554.6 | 215549430.3 | 142624579.5 | 359990755.8 | 296979337.5 | 387806637.5 | 362533212 | 222828138.2 | 245828503.3 | 232442707.5 | 181844947.4 |
|  |  | 2.087725181 | 4.762612829 | 0.001551371 |  | 99019907.84 | 57022472.48 | 150503837.1 | 162958667 | 31120795.06 | 86943851.02 | 31286231.37 | 7155837.992 | 126704727.1 | 191203257.7 | 222753764.7 | 204719141.1 |
|  |  | 1.950085182 | 2.166614681 | 0.001570328 |  | 258717517.6 | 108652834.6 | 189880073.1 | 166200857.4 | 64412975.82 | 159462529.4 | 114399526.4 | 96630984.2 | 235407081.7 | 261174726.8 | 203137373.9 | 242554576.4 |
|  |  | 2.00951391 | 2.323394161 | 0.001636364 |  | 195216063 | 117889358.4 | 217311584.3 | 112880799.1 | 79855163.48 | 153524623.6 | 88988635.45 | 85373370.24 | 222598681.5 | 235352364.2 | 286653929.1 | 202739925.8 |
|  |  | 1.128412904 | 2.559656962 | 0.001670163 |  | 66282610.78 | 26875006.99 | 63436528.23 | 64994177.98 | 29540375.91 | 29535689.15 | 34186708.48 | 17554714.2 | 76056494.83 | 86054406.38 | 51931525.63 | 69612327.14 |
|  |  | 1.944946092 | 2.261514135 | 0.001695253 |  | 233746975.6 | 81826308.25 | 248086483.4 | 320696730.5 | 73288440.65 | 105267911.7 | 134776032.3 | 106526159.8 | 177908286.9 | 236887994.9 | 271786226.1 | 262933525.2 |
|  |  | 1.07365515 | 2.850177943 | 0.001712503 |  | 21199941.42 | 53795863.93 | 53653576.54 | 17330359.86 | 18281996.24 | 24006797.04 | 22779000.35 | 18850415.45 | 74522280.26 | 69263143.76 | 46344799.82 | 49051604.63 |
|  |  | 1.296612951 | 2.06599548 | 0.001771757 |  | 104518966.2 | 80874620.5 | 69771303.92 | 72946614.67 | 49122856.78 | 79256119.52 | 48838902.11 | 34893447.89 | 98260693.93 | 108856227.9 | 108542732 | 122561387.7 |
|  |  | 1.032364109 | 2.473720849 | 0.001876927 |  | 69420368.59 | 17173230.92 | 42597067.95 | 68681334.75 | 20039149.13 | 23352593.35 | 22467530.22 | 31153276.06 | 57054854.35 | 56921696.73 | 48074484.02 | 77930929.36 |
|  |  | 1.294203823 | 2.605760457 | 0.001953477 |  | 119579358.5 | 182363113.8 | 84338646.89 | 109215788.2 | 29587552.28 | 39050192.25 | 23499035.73 | 42944430.29 | 93281057.4 | 60282964.28 | 101013102.5 | 97412152.7 |
|  |  | 1.47036953 | 1.727847673 | 0.002055061 |  | 192868555.7 | 88199505.28 | 123652444.2 | 127963769.3 | 84773715.89 | 107124789.2 | 80205053.47 | 119201478.1 | 186280119.4 | 150964257.2 | 187030094.7 | 151841025.6 |
|  |  | 1.328566543 | 1.857255602 | 0.002135025 |  | 121356288.7 | 78567474.88 | 141679413 | 117718325.3 | 59349782.98 | 55278535.47 | 67342435.63 | 92466060.38 | 116940192 | 111853120.9 | 135716332.3 | 145189666 |
|  |  | 1.55237198 | 1.61651744 | 0.002151903 |  | 201915250 | 115730509.4 | 148891617.7 | 181429561.7 | 117171525.6 | 175742556.7 | 116881089.6 | 115586675.3 | 213887780.1 | 206748205.3 | 201755901.5 | 226897031.9 |
|  |  | 1.270196714 | 2.135966797 | 0.002168119 |  | 114740972.8 | 65118166.58 | 102885792.8 | 77750237.48 | 34690393.95 | 77521590.62 | 38567099.09 | 36823589.75 | 95063279.69 | 103304832.2 | 100362525.3 | 101982444.3 |
|  |  | 3.718511542 | 3.410511666 | 0.002172704 |  | 391214560.1 | 209250700.4 | 566621481.2 | 545747687.3 | 198874333.6 | 349280059.3 | 142625408.3 | 93285666.78 | 479196787.7 | 637626596 | 824177829.1 | 733063213.1 |
|  |  | 1.391527826 | 0.503037252 | 0.00225331 |  | 118429846.3 | 98981146.48 | 112976363.7 | 42401427.48 | 121699722.1 | 127300930.6 | 133950120.6 | 120869767.1 | 42093152.15 | 58371665.96 | 97540824.35 | 55434857.88 |
|  |  | 1.15093292 | 0.318485785 | 0.002273944 |  | 3304381.57 | 10009506.89 | 2588659.788 | 11102049.37 | 74641462.55 | 62247228.41 | 52323487.59 | 54106736.91 | 10498825.47 | 37849858.04 | 11124077.73 | 18020854.6 |
|  |  | 1.121135486 | 2.804324312 | 0.002473708 |  | 109681287.3 | 67924673.23 | 61679523.35 | 127769727.8 | 18762492.05 | 20417405.31 | 32924866.66 | 24606226.7 | 71461932.23 | 75475483.18 | 80302045.77 | 43969521.37 |
|  |  | 1.342555937 | 1.565264977 | 0.002555392 |  | 188995001.2 | 133110795.3 | 179907787.8 | 177746568.9 | 113623732.4 | 130520731.7 | 101409811.5 | 104797499.3 | 191751061 | 143627748.8 | 187821680.3 | 181719370.3 |
|  |  | 1.621482376 | 0.372303373 | 0.002740755 |  | 63266259.72 | 96678494.26 | 90297362.62 | 43924193.13 | 130603498.4 | 155334044.8 | 158938331.4 | 102765411.9 | 80026103.46 | 54525574.97 | 46686262.76 | 22650757.2 |
|  |  | 2.317135506 | 1.979576233 | 0.002746983 |  | 615964880.1 | 454326359.6 | 303110635.4 | 287874644.1 | 145693797.5 | 230153731.7 | 179928405.7 | 198621865.3 | 285341484.2 | 428953010.3 | 357569211.9 | 421524249.3 |
|  |  | 1.077562661 | 2.300892421 | 0.002772683 |  | 83666731.4 | 90716161.49 | 56112777.56 | 88081748.51 | 25889543.45 | 33395788.96 | 22045039.65 | 37299594.18 | 58760707.62 | 53834001.81 | 76736106.64 | 83623974.2 |
|  |  | 1.255298188 | 2.328386917 | 0.002856328 |  | 82063836.45 | 69493085.31 | 52252342.96 | 76014905.76 | 40135052.55 | 59162264.75 | 27056206.92 | 36675896.02 | 93084255.18 | 112088936.9 | 103580376.4 | 70842000.72 |
|  |  | 4.134491619 | 0.288425253 | 0.002889164 |  | 302847784.7 | 246213515 | 290792909.2 | 215477218.3 | 633078437 | 1071935854 | 665724375.9 | 752028982.9 | 232803359.1 | 380052529.6 | 157480704.6 | 130348454.8 |
|  |  | 1.784055574 | 0.17297398 | 0.002897254 |  | 15897082.18 | 26827670.18 | 37562348.52 | 9979566.428 | 142294527.6 | 155718366.1 | 112893315.7 | 64528365.24 | 18861320.76 | 25411947.67 | 23145117.14 | 14819425.25 |
|  |  | 1.73214079 | 3.067143918 | 0.002929668 |  | 201839574.8 | 79750154.99 | 72832148.51 | 25402698.33 | 43090455.2 | 61266390.08 | 43879069.09 | 45964334.27 | 138738886.3 | 96607637.34 | 189750839.2 | 170542748.6 |
|  |  | 1.222688877 | 2.99174004 | 0.003044557 |  | 28045917.61 | 88199236.34 | 85803767.31 | 87437978.04 | 21352266.46 | 31671715.07 | 31982744.71 | 20419591.46 | 75038341.81 | 87262102.61 | 101330565.9 | 51777125.69 |
|  |  | 1.365622198 | 1.989680986 | 0.003083387 |  | 77269617.66 | 73690162.07 | 76206873.42 | 154555154 | 62593249.67 | 73291954.99 | 53131465.71 | 71143040.63 | 126333995.3 | 151539915.4 | 144861168.7 | 94899750.88 |
|  |  | 4.646501816 | 1.639591296 | 0.003093748 |  | 1770479622 | 1700031786 | 1493796835 | 1718902982 | 1052925350 | 1560133877 | 995708792.5 | 965915195.9 | 1793060472 | 1765532113 | 2041267289 | 1900750907 |
|  |  | 4.273544149 | 1.81423792 | 0.003171315 |  | 2710089315 | 1449368691 | 1133464601 | 1334185803 | 532195837.1 | 973716245.8 | 838247626.1 | 760962389.6 | 1360129642 | 1491956377 | 1166773760 | 1614570477 |
|  |  | 1.303764643 | 0.318847881 | 0.003234545 |  | 42876187.05 | 368414169.4 | 36645021.68 | 19461570.73 | 63418720.88 | 85702731.52 | 95109711.51 | 89302058.54 | 19948275.24 | 17634603.82 | 55714788.38 | 13048693.64 |
|  |  | 1.025603402 | 1.85359969 | 0.003247151 |  | 55300064.7 | 74435141.22 | 49004237.53 | 59458202.44 | 47285393.6 | 59317576.38 | 38194283.73 | 28799985.88 | 76008832.28 | 85144546.5 | 90000306.19 | 70626104.44 |
|  |  | 1.994915167 | 3.676551202 | 0.003310911 |  | 108703368.1 | 58373981.45 | 181347281.9 | 143171097.5 | 41377644.78 | 102194285.7 | 48588595.28 | 13872041.12 | 126790878.9 | 184961382.1 | 226670319.4 | 219066700.9 |
|  |  | 1.061363085 | 1.862542797 | 0.003464823 |  | 65486369.64 | 52978813.85 | 61992830.89 | 56956488.03 | 36648211.12 | 57204784.14 | 35159024.72 | 48228280.96 | 101361108.6 | 78281702.54 | 73849417.67 | 76625417.07 |
|  |  | 4.127821495 | 4.418808463 | 0.003521845 |  | 259141924.5 | 155634809 | 136686466.3 | 104825373.4 | 37394940.87 | 128646055.2 | 58132469.23 | 412682020.3 | 877111913.6 | 796948519.1 | 574963979.9 | 565117996.9 |
|  |  | 3.502915047 | 2.9966161 | 0.003528964 |  | 506452523 | 342617082.6 | 202583110.1 | 186320925.8 | 142954625.4 | 279146397.3 | 301454607.9 | 124189844.8 | 563273914.1 | 451453517.3 | 723002773.2 | 802637535.4 |
|  |  | 1.786677407 | 1.905882457 | 0.003822641 |  | 182619189 | 182039774.9 | 236961613.5 | 185990325 | 117279907.5 | 160765977.6 | 102727434.7 | 114849031 | 221374787 | 271991874.1 | 267985973.7 | 183245309.1 |
|  |  | 1.879532614 | 2.567583905 | 0.003874857 |  | 184736015.8 | 266592709.8 | 298139346.2 | 189579118.9 | 66263801.26 | 137254725.5 | 82609607.68 | 22569770.84 | 166179598.4 | 195056320.9 | 222064170.4 | 209307683.6 |
|  |  | 1.042734145 | 0.26158977 | 0.003900842 |  | 6205072.13 | 58791451.26 | 2798603.601 | 10915958.78 | 62736910.22 | 57269659.35 | 31919408.5 | 49617682.39 | 21980165.35 | 20521845.89 | 5140878.262 | 5078870.368 |
|  |  | 1.404467252 | 2.342459259 | 0.003999326 |  | 69723273.71 | 61121805.06 | 61561105.65 | 61141344.5 | 50298243.34 | 58322257.72 | 44001782.05 | 56660970.04 | 103929872.2 | 126165887.8 | 163917316.1 | 96224418 |
|  |  | 4.508161484 | 3.85586256 | 0.004004581 |  | 677060894.3 | 256553335.1 | 232096369.4 | 624348359.1 | 63428045.24 | 321795027.5 | 360439309.4 | 260187989.7 | 672139442.2 | 1120243329 | 789742937.1 | 1296295082 |
|  |  | 1.07896818 | 3.776609284 | 0.004102204 |  | 56235533.24 | 26066080.29 | 32060668.63 | 24756582.37 | 16603241.31 | 16655667.5 | 24560275.74 | 1687506.404 | 64275141.97 | 60024985.67 | 67328394.62 | 33104999.25 |
|  |  | 1.480896826 | 2.04404089 | 0.004107706 |  | 121605009.3 | 129975938.5 | 111974853.1 | 130809583.2 | 56376768.39 | 95395232.1 | 95834978.45 | 58822201.68 | 115102466.3 | 172605149.4 | 164739290.1 | 173906869.4 |
|  |  | 1.641601453 | 2.409213778 | 0.004113401 |  | 134120749.6 | 54125340.43 | 99781314.58 | 65873507.45 | 37404389.55 | 100738393.1 | 35760704.07 | 80296559.66 | 148962740.1 | 169528398.8 | 121709378.5 | 172221736.9 |
|  |  | 2.250024688 | 0.563285794 | 0.004284136 |  | 327116987.1 | 421281575.8 | 687784326.9 | 328728111 | 389415216.7 | 311958977.1 | 334848054.7 | 461675368.4 | 169492284 | 209126324.5 | 241893472.2 | 223232367.6 |
|  |  | 1.87362011 | 0.221674891 | 0.00430183 |  | 9696634.835 | 397456231.1 | 96506651.78 | 20715571.07 | 183237153.6 | 174815149.4 | 102945175.3 | 130949966.5 | 30907875.4 | 83309022.61 | 9303151.777 | 7699835.284 |
|  |  | 1.244120296 | 1.523102169 | 0.00450055 |  | 165512497.9 | 94082094.05 | 109643847.9 | 154731955.6 | 70304475.84 | 122218825.2 | 86046537.15 | 105744779.2 | 143555013 | 145667840.1 | 148131937.7 | 147995636.4 |
|  |  | 1.856964378 | 0.211284188 | 0.004526233 |  | 65031813.2 | 36549308.24 | 25952567.53 | 44243856.13 | 140305325.9 | 209775079.1 | 106655685.2 | 102059186.1 | 31872929.48 | 22645283.53 | 29219752.8 | 34326640.36 |
|  |  | 2.474950249 | 2.070222667 | 0.004693141 |  | 794234634.1 | 549054633.4 | 394841707.3 | 655843640.1 | 164561642.4 | 282089445.6 | 204239970.9 | 158795562 | 342943303.5 | 399212331 | 533909645.6 | 400166315.7 |
|  |  | 1.334384166 | 0.491621697 | 0.004776046 |  | 135757423.7 | 1623044446 | 791535511.2 | 233795167.6 | 113683091.6 | 95271840.51 | 145966589.3 | 109561446 | 58148474.28 | 71849055.29 | 64706042.63 | 33646332.24 |
|  |  | 1.990415969 | 0.307862017 | 0.004776142 |  | 99911502.72 | 404031323.3 | 173242358.6 | 142639115.2 | 221076781.7 | 118376712 | 167579232.6 | 239357105 | 61819367.51 | 85994427.58 | 31752146.81 | 50219136.81 |
|  |  | 1.925694206 | 0.437406148 | 0.00481612 |  | 148569180.3 | 105825920.9 | 209097276.4 | 108399978.8 | 145849663 | 235287753.5 | 204848915.2 | 259111772.8 | 72879549.66 | 119059572.6 | 70816442.09 | 106895542.1 |
|  |  | 1.16816559 | 1.58042593 | 0.004832282 |  | 209911154.3 | 110768001.4 | 130471719.9 | 129143051.6 | 86186708.84 | 103043739.2 | 83361746.56 | 74738730.91 | 118689147.3 | 126647705 | 164258572.2 | 139335376.5 |
|  |  | 1.016175793 | 0.374228143 | 0.004970068 |  | 905935.9319 | 12605565.57 | 3980925.126 | 662218.0806 | 45922397.84 | 58846033.14 | 71469428 | 43429710.52 | 25010906.83 | 30914884.17 | 10047149.6 | 16232845.94 |
|  |  | 1.876790829 | 0.209337457 | 0.004996068 |  | 96709308.64 | 154168824.6 | 237889265.1 | 100165941.7 | 143351503.7 | 186191614.8 | 151601463.8 | 72653998.15 | 19879340.04 | 52762628.12 | 30375459.28 | 12913359.34 |
|  |  | 2.674594655 | 2.172585971 | 0.005157391 |  | 470254936.3 | 159319409.2 | 292892338.9 | 340869518.4 | 78375109.26 | 242365253.5 | 201211726.4 | 273875923 | 419134885 | 369474326 | 413288246 | 527107317.1 |
|  |  | 1.512843512 | 1.512276091 | 0.005225199 |  | 290043217.5 | 170967772.5 | 230408844.2 | 277073939.8 | 151333999.2 | 205513694.9 | 137509553.3 | 130394578.5 | 234521529.8 | 217625214 | 236924200.7 | 255726304.8 |
|  |  | 1.91463244 | 2.040656485 | 0.005421458 |  | 270223520.5 | 177464510.2 | 214628657 | 349294507.5 | 135761216.9 | 170892855.4 | 153992829.1 | 48514185.52 | 217011059.5 | 271761514.1 | 288549761 | 261700539.3 |
|  |  | 1.377169947 | 1.858441308 | 0.005548256 |  | 145874568.1 | 97170202.86 | 116997189.2 | 85435877.24 | 57514388.18 | 89683605.31 | 52688416.34 | 96006635.49 | 111440999.7 | 148904232.6 | 131676895.9 | 157877730 |
|  |  | 2.115698506 | 0.506219465 | 0.005554009 |  | 37544513.6 | 217209683.2 | 108119682.6 | 205683135.2 | 271637572.5 | 295838364.6 | 355469729.8 | 260053015.8 | 170653168.7 | 217367358.5 | 106009915.5 | 104826517.1 |
|  |  | 1.861153119 | 0.272110144 | 0.00557282 |  | 24109861.2 | 88387724.69 | 54465261.27 | 28847169.03 | 156110861 | 209224049.9 | 101472376.8 | 174968379.5 | 35726784.17 | 90089652.38 | 33121164.94 | 15696067.92 |
|  |  | 1.0207951 | 1.697733455 | 0.005592869 |  | 96342358.56 | 76091446.51 | 66677485.12 | 168899377.8 | 48200000.15 | 69085873.79 | 42558369.89 | 44424062.64 | 90290214.65 | 71249508.06 | 99195258.14 | 86058156.74 |
|  |  | 1.619786789 | 3.236209421 | 0.005684189 |  | 33957954.41 | 122066002.7 | 117853919.6 | 78712722.51 | 56948594.66 | 62580286.8 | 34905211.27 | 9995736.953 | 156846242 | 168654051.2 | 88703132.64 | 117925938 |
|  |  | 2.66873958 | 3.149730793 | 0.005697931 |  | 103906163.9 | 109690901.9 | 77667662.33 | 113774486.7 | 134324464.2 | 104845855.2 | 180514943.8 | 58613798.63 | 423120681.1 | 225363847.9 | 371587758.3 | 486440995.9 |
|  |  | 1.485814638 | 3.013783797 | 0.005795555 |  | 137254114.7 | 89454575.6 | 59890189.39 | 138012300 | 20319429.98 | 74606053.52 | 55837639.7 | 1838557.629 | 104999490 | 106776493.9 | 109268397.9 | 138864091.3 |
|  |  | 1.535401044 | 1.87668154 | 0.006023188 |  | 133274370.2 | 87691128.2 | 137210291 | 101436323.5 | 64827110.35 | 124715044.1 | 50487864.59 | 109355715.1 | 165304939.7 | 155335693.7 | 178502495.9 | 156542628.3 |
|  |  | 3.094399991 | 3.041935916 | 0.006030727 |  | 580048302.5 | 248315738.9 | 247128033.7 | 277728072.6 | 212959760.4 | 231553961.4 | 202609058.9 | 21802576.4 | 689059970.5 | 397210389.6 | 422307255.2 | 526250453.6 |
|  |  | 1.044897794 | 2.308747669 | 0.006033429 |  | 81199296.89 | 44226843.89 | 55521793.37 | 73542881.87 | 24058983.11 | 22595380.82 | 28454191.93 | 36864864.49 | 56283122.74 | 88750328.38 | 61060059.21 | 52424862.93 |
|  |  | 2.293981122 | 3.012319338 | 0.006199802 |  | 54517480.32 | 136726709.8 | 259047062.8 | 205986536.1 | 163421861.4 | 92842078.97 | 127455321.3 | 11263842.49 | 218445830.1 | 265947908 | 322338037.4 | 383083467.2 |
|  |  | 2.310038431 | 1.668216689 | 0.006267303 |  | 400306706 | 314612258.9 | 406685058.5 | 385832351.2 | 242604125.7 | 360340510.6 | 241610517.6 | 252214337.5 | 505374153.6 | 371185846.5 | 435405922.9 | 517683246.5 |
|  |  | 4.095647099 | 2.100652962 | 0.006300497 |  | 965695111.6 | 419681578.9 | 491565618.8 | 455383517.2 | 250916275 | 585134614.4 | 329877605.5 | 769461226.6 | 1140773198 | 1014962258 | 888436136.9 | 1021410559 |
|  |  | 4.387323048 | 2.336739408 | 0.006429471 |  | 866787033.8 | 497419654.5 | 533536434 | 964864937.4 | 577474813 | 531187223.9 | 460505749.4 | 517058290.5 | 1290888521 | 1366977972 | 727592594.2 | 1489507601 |
|  |  | 1.382941057 | 1.860995768 | 0.006536135 |  | 156855605.9 | 87425999.01 | 146837323 | 139161651.3 | 43506643.48 | 109604071.9 | 63490022.12 | 75522542.32 | 140186880.2 | 120661873.2 | 152331566.2 | 130459867.8 |
|  |  | 1.01659374 | 0.102424089 | 0.006607401 |  | 1676597.712 | 5475715.508 | 5865165.099 | 49648578.37 | 39356742.73 | 43209986.97 | 48507497.73 | 12939347.47 | 3504201.479 | 5575209.326 | 1949311.072 | 3721737.333 |
|  |  | 1.32466258 | 1.592820859 | 0.00663158 |  | 199439875 | 94615479.98 | 133354732.2 | 187462571.9 | 101450151.3 | 137655459.8 | 99741496.22 | 86963761.6 | 157394608.8 | 201738895 | 152903584.1 | 166203346.3 |
|  |  | 1.887993098 | 4.15594643 | 0.006702574 |  | 43458399.53 | 43427033.34 | 79035994.03 | 31228052.32 | 25116025.3 | 48828079.18 | 14795059.49 | 63937332.22 | 232717849.2 | 103122602.3 | 162800078 | 135874809.7 |
|  |  | 9.393499644 | 1.571603884 | 0.006758551 |  | 9197169371 | 7294737841 | 6638017864 | 10192089244 | 4804967970 | 7080735911 | 3876239953 | 5516222365 | 7711708994 | 8405468258 | 9247224944 | 8076446456 |
|  |  | 1.167759509 | 0.132755104 | 0.006931219 |  | 10730332.96 | 28385743.28 | 14065178.47 | 11172877.36 | 84320035.26 | 59113189.45 | 27036951.4 | 50765894.76 | 6163046.122 | 11523514.89 | 7581064.136 | 4102592.523 |
|  |  | 1.769584016 | 2.189025867 | 0.006935481 |  | 139427380.1 | 127044761.7 | 172567445.7 | 218948204.4 | 105399527.6 | 112984752.2 | 126505436.7 | 46995793.92 | 192314928.2 | 170506965.8 | 217838289.2 | 277187336 |
|  |  | 2.732550314 | 0.214740584 | 0.006999219 |  | 162488658.5 | 279613786.4 | 295152512.6 | 197864964.1 | 451274113 | 360632737.7 | 242445052.7 | 188112200 | 75116082.16 | 73945151.15 | 26445227.95 | 91301006.48 |
|  |  | 1.181837423 | 0.2618438 | 0.007073367 |  | 22429875.57 | 100025350 | 31775212.52 | 28629534.26 | 84685438.92 | 61561870.85 | 60471041.21 | 31636143.84 | 15119194.53 | 18542215.65 | 17474973.41 | 11275263.14 |
|  |  | 1.435924369 | 1.782415414 | 0.00709356 |  | 94607007.22 | 86878222.19 | 82115138.82 | 68474419.51 | 95916819.41 | 97910532.87 | 66607801.26 | 114018041.3 | 195662849.1 | 131137492.6 | 189266011.2 | 151364793.3 |
|  |  | 1.063833958 | 1.815077691 | 0.007121949 |  | 51325721.93 | 108184500.9 | 48229683.15 | 84390394.3 | 45861848.34 | 56943013.62 | 38720083.52 | 50073245.89 | 93711351.09 | 61073627.17 | 102454696.3 | 90525928.18 |
|  |  | 5.569323734 | 1.609941445 | 0.007244268 |  | 3402654663 | 2477746612 | 2780953320 | 3334142404 | 1465737750 | 2514810798 | 1604986454 | 1501447973 | 2750632115 | 2863546898 | 3144062462 | 2651386135 |
|  |  | 1.358459862 | 2.109145314 | 0.007538461 |  | 137072313.5 | 93791966.46 | 101519662.7 | 113446235.1 | 35291604.18 | 94288505.43 | 47164928.84 | 46841506.43 | 104498904.1 | 102900970.1 | 125290001.9 | 138886637.3 |
|  |  | 4.66714189 | 2.070542624 | 0.007633322 |  | 1408310593 | 957779740.1 | 1042147850 | 1053839436 | 558754981.8 | 1166513976 | 746154935.4 | 327119617.4 | 1607167994 | 1283981744 | 1413907353 | 1489446533 |
|  |  | 2.72761092 | 2.509436795 | 0.007765764 |  | 264814925.1 | 551116496.2 | 476479263.3 | 225734569.2 | 156856510.9 | 256700651.1 | 239357071.8 | 53736652.65 | 470166250.8 | 560543491.1 | 419966122.6 | 322619871.6 |
|  |  | 1.150276025 | 0.404620028 | 0.007894804 |  | 122084875.3 | 29563215.95 | 10826527.56 | 11639323.27 | 76911072.87 | 48362091.92 | 67323517.38 | 92393592.88 | 28295297.06 | 45196724.26 | 21465817.61 | 20354934.14 |
|  |  | 1.21320658 | 6.246817777 | 0.007952348 |  | 17214774.68 | 42501699.79 | 1645498.711 | 57898787.7 | 25735100.4 | 8647617.635 | 737217.4416 | 5662640.362 | 98772135.62 | 64278910.46 | 49893181.68 | 41817091.98 |
|  |  | 1.854916663 | 1.538000194 | 0.008125942 |  | 342514180 | 324598598.8 | 474461283.6 | 263921079.2 | 335204170.3 | 240117515.1 | 288066030.8 | 189675483.1 | 399536415.4 | 413071510.2 | 448838172.8 | 358165306.8 |
|  |  | 1.196622079 | 4.779162133 | 0.00818102 |  | 27448851.61 | 6594789.724 | 27252928.93 | 61565347.02 | 12686499.67 | 22581765.98 | 13980028.04 | 5032935.968 | 80796136.87 | 81017594.88 | 27449635.68 | 70155429.84 |
|  |  | 1.273820597 | 1.625151028 | 0.008240052 |  | 144157219.9 | 90604759 | 125853440.8 | 109514813.7 | 69520565.52 | 121898504 | 61937428.71 | 97217388.73 | 145192427.5 | 150988758.4 | 137969130.4 | 135585196.6 |
|  |  | 2.301049881 | 2.272648583 | 0.008240843 |  | 385784414.7 | 138642961.4 | 251026450.2 | 271131574.8 | 75307785.07 | 185856508.1 | 152054841.6 | 164054127.3 | 298827961.9 | 261566644.2 | 447276807.4 | 304267847.7 |
|  |  | 1.170741584 | 0.221624173 | 0.008261082 |  | 19777352.12 | 8552673.015 | 30717672.59 | 17088586 | 55748039.52 | 91267395.05 | 32754129.96 | 62794822.03 | 16024228.4 | 15983262.21 | 13534455.59 | 8216185.336 |
|  |  | 1.194841982 | 1.86121703 | 0.008266933 |  | 186490381.3 | 90561353.72 | 67125558.81 | 59975570.89 | 38279199.42 | 67865963.95 | 48802246.83 | 64793965.69 | 117539824.3 | 73507110.21 | 114620851.3 | 103318605.2 |
|  |  | 1.375194922 | 5.757877176 | 0.00836256 |  | 30683115.42 | 14883580.07 | 4628783.867 | 7904139.741 | 992440.9747 | 6808051.553 | 4186576.276 | 42592008.09 | 50908182.82 | 83611649.91 | 65229735.63 | 114510052.8 |
|  |  | 5.116983589 | 2.893626759 | 0.008448711 |  | 1030011885 | 829496094.9 | 761952020.1 | 661115222.1 | 500457646.2 | 627958296.6 | 639022167.1 | 206044432.7 | 1597611426 | 841893169.2 | 1384639018 | 1886378280 |
|  |  | 4.092766641 | 0.250104015 | 0.008754229 |  | 207940999 | 1823106810 | 395046231.3 | 262645580.4 | 561007156.7 | 463956036.1 | 828333417.7 | 1069999556 | 150216214.7 | 309050780.3 | 157270510.4 | 114590602.4 |
|  |  | 1.940198663 | 0.40044684 | 0.008946437 |  | 186589387.3 | 202984086.9 | 94682285.36 | 83519723.84 | 250642833.8 | 252128470.7 | 159494680.9 | 178999663.6 | 66831113.11 | 124543633 | 117864078 | 27643346.68 |
|  |  | 1.660616224 | 0.264586229 | 0.009026151 |  | 50676472.41 | 33152855.89 | 37634176.09 | 64606135.81 | 170235772 | 113390924.1 | 70898720.94 | 181462761.6 | 40082009.59 | 40634245.99 | 34076189.95 | 27022645.54 |
|  |  | 1.452689099 | 1.646205764 | 0.009032549 |  | 183416684.4 | 114249136.6 | 153516032.1 | 163272032.8 | 82082224.11 | 156335376.6 | 81965577.68 | 122463520.8 | 184408085.3 | 197659483.2 | 171812932.9 | 175136287.5 |
|  |  | 1.020458894 | 2.261075545 | 0.009090137 |  | 55105585.39 | 93166955.65 | 83599194.36 | 47135290.09 | 38964077.54 | 40293749.68 | 33657880.94 | 5957146.981 | 73929727.24 | 78072103.35 | 52138385.08 | 64640290.04 |
|  |  | 1.954374501 | 0.234106103 | 0.009162463 |  | 23824061.24 | 146954110.4 | 111983962.1 | 26636993.69 | 201771723.7 | 237016260.6 | 83903946.66 | 198950533.6 | 72860390.36 | 62036014.73 | 24142914.56 | 9901585.429 |
|  |  | 1.459343939 | 1.516273789 | 0.009178585 |  | 367014771.3 | 184681490.3 | 200482816.6 | 305570943.2 | 121949382.4 | 179038664.4 | 108458213.3 | 146440314.9 | 213096285.7 | 181392384 | 234490797.9 | 213896775.5 |
|  |  | 1.281072389 | 2.897109051 | 0.009425881 |  | 71795778.98 | 99072421.9 | 55637743.46 | 79217729.08 | 70554013.73 | 14188712.81 | 41767510.77 | 10927606.22 | 103085350.9 | 73013945.45 | 98589859.9 | 123483264.2 |
|  |  | 1.95191333 | 2.395612416 | 0.009429337 |  | 186500611.7 | 115299699.9 | 145709277.9 | 186895859.5 | 96202131.69 | 128370919.9 | 81803882.46 | 88891556.87 | 296387298.3 | 182340658.8 | 169406685.7 | 298775461.6 |
|  |  | 1.296356532 | 0.559995936 | 0.009454365 |  | 12977360.17 | 47386277.68 | 41073533.26 | 15103071.29 | 113265408.4 | 107391899.7 | 144535923.1 | 133359523.2 | 70705509.95 | 99835140.08 | 42513609.42 | 66133256.61 |
|  |  | 2.768272495 | 0.554666524 | 0.009534732 |  | 159052657.7 | 437536297.4 | 200372735.4 | 343808694.4 | 590242521.4 | 533506866.2 | 505846132.4 | 544481337.5 | 287309525.3 | 481271115.7 | 211443843.9 | 225863169.2 |
|  |  | 2.489931187 | 5.416204263 | 0.009581318 |  | 41662866.13 | 55453462.71 | 107338722.1 | 32321438.44 | 34825922.57 | 56311935.88 | 24271565.56 | 81893896.07 | 426161969.2 | 176511318.3 | 270708698.7 | 195253097.2 |
|  |  | 1.353857623 | 0.449374007 | 0.009736709 |  | 64926862.94 | 52096783.23 | 50471397.17 | 106957280.4 | 86205421.25 | 147873496.6 | 118443615.3 | 86209789.18 | 41634221.16 | 65882313.34 | 53024217.77 | 36614149.17 |
|  |  | 3.441124352 | 0.388936689 | 0.009738253 |  | 48282464.79 | 165673490.2 | 80306341.68 | 32624198.2 | 546054613.1 | 661355979.9 | 961546494.9 | 531849834.9 | 321192802.5 | 359520105.7 | 149431726.5 | 220298268.4 |
|  |  | 1.625553095 | 1.876503611 | 0.009767361 |  | 164361899.6 | 132358215.9 | 139465588.9 | 119898940.9 | 86949434.28 | 142350669 | 59384493.42 | 119348807.6 | 201567586 | 159775652.2 | 230432393 | 173900525.2 |
|  |  | 1.024699177 | 3.850307331 | 0.00977527 |  | 24781843.84 | 20513341.53 | 18760049.99 | 26437196.88 | 9123558.196 | 13844207.16 | 6878132.295 | 22157535.4 | 64705345.96 | 56971116.32 | 55928852.86 | 22623884.37 |
|  |  | 1.349312973 | 1.855413265 | 0.009881886 |  | 130936051.2 | 98560174.01 | 120163343.8 | 142494953.6 | 57671697.33 | 117383731.3 | 60146677.29 | 60996874.53 | 114583299.7 | 131332857.6 | 152367731.2 | 151287628.9 |
|  |  | 1.264064743 | 0.490242172 | 0.010135952 |  | 22833648.06 | 40816493.92 | 29630403.08 | 20850547.15 | 79261319.9 | 95147656.88 | 128183598.9 | 131333631.8 | 63386137.47 | 68780162.28 | 34625181.99 | 45937444.76 |
|  |  | 3.542065378 | 3.424226273 | 0.010169463 |  | 236744579 | 218467109.1 | 191030698.5 | 144103219.6 | 94736265.92 | 190775402.1 | 78030619.66 | 342306041 | 741005590.3 | 758061649.5 | 331799054.1 | 586118097.4 |
|  |  | 1.007785067 | 2.115864752 | 0.010222316 |  | 74961477.9 | 52778839.3 | 66512687.64 | 49215290.66 | 37361343.81 | 53037374.24 | 31166293.6 | 6658532.133 | 66757753.89 | 65438954.96 | 70366331.61 | 68740636.24 |
|  |  | 1.263523505 | 1.733445046 | 0.010264759 |  | 152161025.5 | 70102233.83 | 92346469.96 | 96704909.96 | 49236759.88 | 105295028.7 | 62655725.68 | 85064743.98 | 117560885.2 | 152625028.3 | 116937802.4 | 136813963.9 |
|  |  | 1.566656616 | 0.457092423 | 0.010492577 |  | 230254251.7 | 692037542.5 | 304093651.3 | 188880390.5 | 114792331.5 | 113068273.8 | 169795537.1 | 199791832.3 | 64012275.1 | 84115736.53 | 57048486.44 | 67912444.16 |
|  |  | 1.072945284 | 0.262777563 | 0.010543416 |  | 14329227.11 | 33750525.16 | 46931632.03 | 9101817.027 | 58228366.71 | 58225888.38 | 37675318.53 | 53869763.04 | 3355517.365 | 41066236.8 | 8651561.352 | 1584243.209 |
|  |  | 1.796777076 | 2.032503264 | 0.010901708 |  | 173043746.5 | 69882327.35 | 112850990.2 | 128365493.4 | 53342841.69 | 116815328.2 | 89353798.39 | 157917092.2 | 270854246 | 188064997.8 | 200309512.6 | 189197171.4 |
|  |  | 1.859164728 | 1.615423499 | 0.011289069 |  | 442688183.3 | 212572350.1 | 268147472 | 287888059.4 | 138026022.6 | 239069493 | 176689456.5 | 218943333.7 | 284513423.1 | 277601850.8 | 382400521.5 | 303767668.4 |
|  |  | 1.318827686 | 2.59592615 | 0.011295298 |  | 57650437.28 | 28696708.38 | 50472797.29 | 39244918.7 | 37787233.99 | 50176249.34 | 20909686.21 | 44906945.67 | 104927613.5 | 142416543.5 | 77674282.03 | 74183383.38 |
|  |  | 1.101186767 | 2.370241391 | 0.011419432 |  | 56504949.65 | 28941228.84 | 57536195.19 | 35579074.71 | 7313405.269 | 37307006.77 | 25227014.22 | 50254292.03 | 52424821.94 | 78263091.41 | 84066006.58 | 69916143.89 |
|  |  | 1.687583814 | 2.113253784 | 0.011656029 |  | 129226608.6 | 178442955 | 95654063.97 | 133974310.5 | 84060869.85 | 139606454.8 | 52664111.65 | 73602118.55 | 188593815.5 | 131559320.2 | 191429910.3 | 227915362.8 |
|  |  | 2.566701957 | 3.095441073 | 0.011662447 |  | 169833012.9 | 139408655.1 | 76017554.49 | 364436358.6 | 101235062.7 | 141665491.4 | 130454273.6 | 85086455.5 | 235087449.2 | 541780990.9 | 306158079.9 | 336051457.3 |
|  |  | 1.013878092 | 1.910034294 | 0.011781029 |  | 102156997 | 50871917.69 | 58703375.33 | 84261255.81 | 30853108.06 | 39726072.64 | 42364252.35 | 50360823.3 | 97169788.23 | 57582176.6 | 91241819.93 | 65922945.19 |
|  |  | 1.534576157 | 0.472606462 | 0.012037105 |  | 101359854 | 102506740.2 | 86982961.27 | 102464236.5 | 205681699.9 | 163195345.2 | 116967160.7 | 121024407.8 | 78306167.29 | 86563391.81 | 75106732.4 | 46833737.06 |
|  |  | 1.059528057 | 1.679913552 | 0.012088851 |  | 88041029.92 | 52652958.31 | 81940626.79 | 80118989.48 | 36564196.84 | 80650132.52 | 43112682.08 | 58196640.86 | 95473045.85 | 82285197.1 | 89912563.41 | 99430038.5 |
|  |  | 1.327647718 | 0.412949496 | 0.012096138 |  | 112193837.5 | 159914824.2 | 102684390.6 | 79210710.49 | 91634122.64 | 73135023.82 | 91844457.75 | 136220182.2 | 42736897.29 | 61102727.97 | 41945285.23 | 16435603.57 |
|  |  | 1.499435863 | 1.884115522 | 0.012101135 |  | 152199035.5 | 87656640.88 | 137734529 | 97284090.95 | 69915868.38 | 126973943 | 49576897.71 | 101048671 | 165757347.4 | 144176481 | 144133996.6 | 200691296.8 |
|  |  | 1.290111502 | 1.81469074 | 0.012127655 |  | 10030623.34 | 10493106.37 | 80976525.73 | 9179188.014 | 47351240.72 | 96577360.7 | 42419542.15 | 80649348.37 | 116318919.9 | 101801610.1 | 126769318.8 | 139628027.5 |
|  |  | 1.168107944 | 1.60545867 | 0.012143583 |  | 101749122.6 | 115062017.2 | 114634429.6 | 124193543.3 | 75693747.27 | 108528103.7 | 70620541.96 | 80395038.83 | 139683663 | 143681723.3 | 101109744.5 | 153734710.4 |
|  |  | 1.570255428 | 2.379503341 | 0.012189275 |  | 143066718.9 | 145473354.1 | 136335066.2 | 1010012129 | 16120516.23 | 100876446.9 | 85480113.02 | 55302333.1 | 125189713.6 | 129803109.4 | 160656197.2 | 197737945.2 |
|  |  | 1.184515536 | 1.775325052 | 0.01243661 |  | 94910241.35 | 61080179.29 | 86898644.86 | 75298152.95 | 48831671.33 | 89224910.23 | 35477162.73 | 72257429.98 | 126176316.5 | 98728609.22 | 110601641.3 | 100852662.3 |
|  |  | 1.161097803 | 2.305524262 | 0.012725269 |  | 68117540.36 | 45247739.14 | 59253614.13 | 45220489.32 | 25940198.93 | 52095458.23 | 29398718.51 | 42446294.81 | 104431585 | 83678625.8 | 51812045.56 | 105631265.8 |
|  |  | 1.065132043 | 4.305667805 | 0.012845657 |  | 10316233.95 | 34793173.78 | 40088786.81 | 12400512.3 | 6386692.214 | 11056377.18 | 27716496.18 | 7130801.259 | 37898196.18 | 35570865.61 | 76226949.32 | 75448937.9 |
|  |  | 1.614065693 | 1.748149064 | 0.01286794 |  | 333415280.1 | 127228692.1 | 137350281.6 | 256537852 | 139595202.9 | 99246510.27 | 139842353.8 | 169942409.5 | 184642029.2 | 247471837.9 | 305804041.3 | 221162953.1 |
|  |  | 2.183289618 | 1.715088536 | 0.012903848 |  | 301946337.5 | 286831541.8 | 411090883 | 505033143.5 | 199610888.1 | 372228808.8 | 209645006.1 | 190494016.8 | 380888443.2 | 480377017 | 430176327.6 | 375587771.9 |
|  |  | 1.912057829 | 0.383885972 | 0.012906685 |  | 68753369.65 | 95837371.18 | 65745578.17 | 58202469.04 | 193276998.5 | 283472994.9 | 115119669.6 | 238901035 | 68371364.41 | 99941531.45 | 72435031.24 | 78173289.91 |
|  |  | 1.13819413 | 0.173968055 | 0.012928055 |  | 14952204.81 | 68021818.65 | 29064040.05 | 11447335.61 | 33209934.55 | 90985235.1 | 49458197.99 | 45437069.6 | 17240427.33 | 11215389.42 | 7523728.6 | 2135191.806 |
|  |  | 1.308429367 | 2.744467239 | 0.013175656 |  | 49938914.3 | 41810424.24 | 99689590.18 | 37618640.93 | 36450988.71 | 71615021.03 | 30668274.87 | 9069114.275 | 64083422.43 | 116275271 | 123522444.7 | 101760447.9 |
|  |  | 1.652547742 | 0.275511094 | 0.013188985 |  | 24517158.08 | 218538896.8 | 25398443.86 | 100381113.1 | 135755082 | 135887299.1 | 144349687.2 | 55794147.9 | 29702162.03 | 66626187.52 | 2959995.128 | 30693991.87 |
|  |  | 1.404356778 | 2.708892364 | 0.013205841 |  | 156777343.6 | 76079058.06 | 27149828.91 | 36562686.6 | 40264900.71 | 82419787.87 | 45385678.48 | 4533338.891 | 80449995.81 | 141536740.3 | 135992695.4 | 109585429.6 |
|  |  | 1.398767719 | 2.896152913 | 0.013250238 |  | 358112748 | 310229292.9 | 292539671.2 | 161440636.4 | 36700895.65 | 45512475.47 | 61446191.34 | 11368982.53 | 79098415.58 | 160884800.3 | 119878513.8 | 89124642.49 |
|  |  | 2.259275887 | 0.113619519 | 0.013458355 |  | 21509580.17 | 588138055.4 | 131445221.9 | 6375190.404 | 327965066.4 | 149362174 | 98919592.47 | 262669886.8 | 7230572.883 | 60053549.18 | 22507595.2 | 5525596.813 |
|  |  | 1.180679447 | 1.891901434 | 0.01352498 |  | 136383834.4 | 72091949.36 | 92218793.62 | 124603580.4 | 30903702.94 | 72945347.06 | 32520985.4 | 73938141.69 | 98848366.73 | 108850480.2 | 108107540.6 | 82075954.26 |
|  |  | 2.699826994 | 1.527569296 | 0.013596845 |  | 853020212.1 | 626134363.1 | 796993914.4 | 775479861.4 | 416336478.2 | 685855224 | 438220001.6 | 399224993.8 | 721678894.2 | 676559210.9 | 823278158 | 741413201.3 |
|  |  | 1.77353387 | 1.573342319 | 0.013617635 |  | 390863367.5 | 275699622.3 | 387545394 | 300339988.7 | 198495368.1 | 200159443.8 | 217547065.5 | 235691122.9 | 380080241 | 248297129.6 | 313051557.7 | 398890380 |
|  |  | 2.298751204 | 0.403647465 | 0.013966608 |  | 151373451.1 | 941966369.9 | 160890364.2 | 131612799.2 | 349360078.8 | 330946734.2 | 177556858.8 | 404361942.5 | 92115567.78 | 176126244.4 | 165869823.7 | 75382533.83 |
|  |  | 1.117391068 | 2.061092065 | 0.014003879 |  | 91720118.55 | 47801839.5 | 64715520.85 | 82055055.98 | 52876090.7 | 66083985.67 | 43820734.6 | 8150700.873 | 90391098.7 | 97724118.96 | 75606852.57 | 88583512.48 |
|  |  | 1.561557232 | 0.490561816 | 0.014035465 |  | 107421339.2 | 82870168.04 | 73201285.81 | 88065921.53 | 152115792.8 | 222243281.7 | 119133009 | 131749204.2 | 73867132.51 | 68765195.29 | 80699912.11 | 83387261.42 |
|  |  | 2.289660746 | 9.981675548 | 0.014128165 |  | 149125098.4 | 145225339.1 | 155096837.8 | 128876090 | 18890646.09 | 21536832.41 | 45041363.98 | 3055252.794 | 114416203.2 | 132040748.4 | 344054293.1 | 293107552.5 |
|  |  | 1.185877472 | 0.656640574 | 0.014428309 |  | 153730268.9 | 125779407.8 | 126792003.4 | 121323550.6 | 131478067 | 149633405.4 | 131035927.6 | 121885173.9 | 56416308.32 | 80645986.41 | 109347235.9 | 104257925.2 |
|  |  | 1.350855899 | 0.430354698 | 0.014477908 |  | 89698236.7 | 146687466.9 | 67638955.37 | 34594322.49 | 116187250 | 108560622 | 71475636.6 | 142306609.8 | 26720573.43 | 71326841.49 | 30005817.54 | 60670264 |
|  |  | 1.055918984 | 1.710403936 | 0.014652199 |  | 140148229.3 | 71405531.95 | 90994898.03 | 119978450.9 | 53526579.88 | 86855569.49 | 53719920.89 | 35571447.42 | 92008991.93 | 99870098 | 112965607.5 | 87989791.22 |
|  |  | 1.346315292 | 0.05435408 | 0.014664632 |  | 2046664.781 | 3720390.171 | 75895395.72 | 2623015.295 | 64787798.73 | 86785303.79 | 78177093.62 | 11743154.47 | 4802861.271 | 3039256.969 | 2931582.327 | 2352448.287 |
|  |  | 1.299946884 | 1.958629036 | 0.014752078 |  | 84315135.57 | 89818917.19 | 86707596.26 | 63937453.51 | 40738156.43 | 77113361.94 | 28000568.08 | 84380063.14 | 131553736.6 | 89040231.85 | 115911902.7 | 114433502.1 |
|  |  | 1.037684942 | 1.975339601 | 0.014871034 |  | 117991432.1 | 93507615.59 | 113187651 | 91247651.94 | 60033232.34 | 47545190.8 | 54644177.51 | 7376308.011 | 82442162.29 | 77014828.63 | 89115300.92 | 86443148.71 |
|  |  | 1.043007019 | 0.308669855 | 0.014937154 |  | 31823546.82 | 33073407.74 | 21720515.92 | 11933990.43 | 23998101.78 | 63013419.95 | 56155901.25 | 77292249.58 | 17444569 | 18637302.13 | 15564126.97 | 16403257.08 |
|  |  | 4.254139567 | 2.229434472 | 0.014945323 |  | 623059305.4 | 510818130.5 | 432564384.9 | 717837359.6 | 870748251.1 | 491009127 | 610952647.4 | 411411063.6 | 1654394946 | 1109866284 | 897880882 | 1653099629 |
|  |  | 1.208402089 | 8.50791851 | 0.015051052 |  | 3300965.967 | 22460569.58 | 13362473.5 | 2650106.416 | 3578655.409 | 6525047.13 | 16025537.1 | 1526411.492 | 71145820.63 | 15079196.11 | 83632523.24 | 65434486.22 |
|  |  | 1.637953629 | 2.498808956 | 0.015382027 |  | 92194530.64 | 139884116.9 | 90608003.71 | 90749461.64 | 52214923.27 | 86989375.11 | 64708185.91 | 73310773 | 95893394.71 | 223268224.2 | 154922416.9 | 218643922.3 |
|  |  | 2.132691474 | 0.318680474 | 0.015527746 |  | 70819010.09 | 446588743.5 | 358493742.1 | 81304754.13 | 128442346.6 | 242329047.4 | 203091120.3 | 332925257.3 | 60984993.72 | 125455728 | 54135852.89 | 48398982.41 |
|  |  | 1.69045141 | 0.135228423 | 0.015609212 |  | 68974860.35 | 37403652.13 | 75692486.97 | 59172713.99 | 131735051.6 | 173180679.7 | 75698336.93 | 50510297.58 | 14749428.84 | 4959710.965 | 24143971.87 | 14447156.54 |
|  |  | 1.039080167 | 2.677685545 | 0.015722217 |  | 40933140.9 | 81504290.36 | 133698877 | 42120984.73 | 31896701.94 | 22271412.01 | 20957512.15 | 23260076.43 | 66018168.73 | 83030042.99 | 82883056.49 | 31514705.28 |
|  |  | 2.426353967 | 1.892170322 | 0.015772681 |  | 666384249 | 456821971.6 | 426423191 | 762542694.4 | 306345241.7 | 304977173.5 | 181913182.8 | 242747800.6 | 663065152.2 | 418429564 | 380393935 | 498368389.6 |
|  |  | 1.490976115 | 2.642977022 | 0.015797824 |  | 220760286.7 | 45469615.1 | 63133248.52 | 170011417.8 | 41802552.67 | 70186331.6 | 62177666.71 | 38415996.26 | 102447918.9 | 150538024.4 | 101546081.7 | 207318762.5 |
|  |  | 1.095069234 | 3.123073698 | 0.015858866 |  | 90645761.26 | 34034787.96 | 45746949.12 | 116968264.7 | 20054879.82 | 33423554.61 | 21095225.24 | 15457879.65 | 32123724.26 | 77004033.03 | 98298552.11 | 73748823.02 |
|  |  | 2.980777757 | 2.00907496 | 0.015940985 |  | 669156030.8 | 518213484.2 | 989085574.6 | 927546994.5 | 330402254.4 | 542286793.3 | 297679900.4 | 147850924.4 | 541762798.1 | 808679661.1 | 696754568.8 | 601205509.1 |
|  |  | 1.268202369 | 5.931817167 | 0.015970703 |  | 36713727.78 | 12086886.87 | 15246325.95 | 13369799.54 | 3115815.612 | 13657899.05 | 8140814.194 | 22730387.55 | 49918487.93 | 111593572 | 85367738.77 | 35741134.33 |
|  |  | 1.21914754 | 1.791233628 | 0.016050068 |  | 98722415.53 | 84942195.18 | 93461592.45 | 188931855.7 | 63228164.32 | 106859998.9 | 70932180.08 | 35570733.08 | 128042000.6 | 128647550 | 136621680.8 | 102128005.7 |
|  |  | 1.589067528 | 1.574410131 | 0.016366944 |  | 173525509.2 | 179105783.2 | 191312310.8 | 220677381.2 | 127251549.7 | 228680843.8 | 128304647.6 | 128280820.9 | 260029162.9 | 218164245.2 | 245473251.2 | 240687667.9 |
|  |  | 1.198542719 | 1.705291818 | 0.01665595 |  | 160919861.9 | 205462399.3 | 194141274.5 | 169745050.6 | 42092268.9 | 111440592.6 | 68333957.62 | 65724840.18 | 134271708.9 | 117035949.1 | 110455196.3 | 128664849.1 |
|  |  | 1.022593294 | 1.592487332 | 0.016958888 |  | 173216672.5 | 72353567.13 | 135155323.8 | 168962896.4 | 55338987.06 | 90770273.36 | 87107056.6 | 42745382.16 | 112274212.7 | 119870159.7 | 105940247.8 | 101380889.8 |
|  |  | 5.406647886 | 2.523041987 | 0.017103252 |  | 1101600830 | 2514033455 | 1570139015 | 1161656935 | 703038662.9 | 1094288437 | 816249241.9 | 299366484.3 | 2155611265 | 2250335300 | 1980602492 | 962928002.2 |
|  |  | 1.435795111 | 1.55279418 | 0.017214772 |  | 306023398.2 | 199149012.7 | 259953382.2 | 375930317.4 | 141137794.1 | 181705178.2 | 112143183.5 | 140384463 | 176832833.2 | 214848634.5 | 229390838.3 | 272359842 |
|  |  | 1.670172353 | 12.5788735 | 0.017274364 |  | 152324677.3 | 15808217.08 | 20692549.8 | 19232836.24 | 5580582.351 | 5449812.533 | 13664608.85 | 9316769.662 | 120890024.6 | 18599105.68 | 135896848.1 | 152443816.7 |
|  |  | 1.957761397 | 3.113088705 | 0.017364624 |  | 94469140.41 | 145519419.2 | 88930225.54 | 77922910.43 | 106616951.7 | 55869136.67 | 91490361.46 | 32326639.13 | 275400373.6 | 221631462.4 | 101168637.6 | 293086438.7 |
|  |  | 1.912533649 | 2.622362427 | 0.01760414 |  | 189621973.6 | 158342611.2 | 131474597.7 | 124563724.7 | 66141720.83 | 108201853.3 | 89977435.43 | 68733448.25 | 164658097.6 | 133812803 | 282058004.5 | 292860591.2 |
|  |  | 1.952511514 | 1.74607015 | 0.017696081 |  | 315632223.5 | 199222396.7 | 255631352.9 | 245899142.9 | 116205191.5 | 248989839.7 | 115184040.8 | 201377677.6 | 266345734 | 257254402.7 | 343374372.9 | 323420600.4 |
|  |  | 1.879350044 | 0.193365735 | 0.017711277 |  | 41467332.4 | 38403257.96 | 44043104.47 | 38835186.78 | 164063387.5 | 192440916.4 | 150891447.3 | 38100976.26 | 26372794.15 | 31955512.7 | 20679414.34 | 26472654.53 |
|  |  | 1.101096616 | 0.448130073 | 0.017929492 |  | 48749341.08 | 61759394.06 | 55580856.72 | 43501203.42 | 58070232.93 | 70690279.73 | 55147322.41 | 102749400.9 | 23777810.99 | 48422959 | 30126030.42 | 26132927.73 |
|  |  | 2.643382663 | 4.693547105 | 0.017949578 |  | 50913054.1 | 26422090.38 | 23160012.6 | 195467592.9 | 62903740.17 | 49489524.87 | 111342514.1 | 62285216.01 | 528360059.8 | 383546938.5 | 284819652.4 | 145726362.9 |
|  |  | 1.265142132 | 1.52045536 | 0.018362037 |  | 215336609 | 138069743.5 | 153582710.5 | 171832404.6 | 115193783.5 | 154542964.8 | 103408571.1 | 93189371.47 | 162287480.6 | 160331467.4 | 216702305.5 | 169719827 |
|  |  | 1.356912973 | 2.073640333 | 0.018580516 |  | 75882066.14 | 73973750.92 | 45747096.73 | 58216272.82 | 58876111.46 | 56600054.36 | 87236812.29 | 65535000.15 | 153856974.4 | 161172700.5 | 75347631.59 | 165872520.4 |
|  |  | 1.161220673 | 2.640266775 | 0.018677522 |  | 34277184.18 | 45188247.72 | 33073720.74 | 22393183.04 | 13046623.65 | 34291203.69 | 21332691.43 | 49665293.2 | 61177491.35 | 100960689.8 | 50944925.14 | 99355006.38 |
|  |  | 2.030109539 | 2.013158785 | 0.018767408 |  | 295008536.5 | 313650591.9 | 359039474.1 | 216053633.7 | 176595691.5 | 234142565.7 | 151650633.7 | 43593112.64 | 372433714.8 | 294911685 | 310432312.2 | 242160282.1 |
|  |  | 1.243648624 | 3.898852982 | 0.018879457 |  | 49623909.14 | 39135252.66 | 20696351.8 | 54115762.16 | 22359567.91 | 20420181.27 | 26218019.53 | 12248009.17 | 129230172.6 | 43730495.1 | 79684782.25 | 64119893.4 |
|  |  | 5.310820824 | 0.15196287 | 0.018888383 |  | 38346050.81 | 1077413723 | 465334226.5 | 133275721.9 | 1119664443 | 1343345101 | 410143084.7 | 1919910751 | 67123704.56 | 232869047.6 | 86974454.12 | 341400462.7 |
|  |  | 1.360786232 | 1.694671633 | 0.018932119 |  | 123007325 | 85438170.78 | 124717191.9 | 90821928.74 | 65711625.33 | 120361429.5 | 53540839.82 | 117923201 | 176102433.1 | 134841644.7 | 150927343.5 | 144036552.6 |
|  |  | 1.003196913 | 1.830793797 | 0.019016535 |  | 73444847.13 | 56064095.65 | 44081353.94 | 45498503.89 | 31431900.56 | 38002115.37 | 36205171.58 | 63702767.38 | 74233616.34 | 62842326.62 | 101430623.1 | 71523634.49 |
|  |  | 1.026179623 | 1.870832575 | 0.01928392 |  | 138339477.7 | 84849790.16 | 106748704.6 | 162128853.9 | 25844909.37 | 31341380.12 | 32567639.28 | 68741296.16 | 60628352.72 | 78116843.44 | 82129130.14 | 75643703.45 |
|  |  | 1.54239259 | 1.724548368 | 0.019417282 |  | 177934026.6 | 108109408.4 | 159589608.3 | 132685689.8 | 76422888.31 | 160573936.6 | 62950240.86 | 139919954.1 | 197314118.9 | 176275038.1 | 209109799.8 | 175872994.3 |
|  |  | 1.343756798 | 3.335069654 | 0.019477931 |  | 29248801.52 | 22043835.09 | 46617366.1 | 25675890.81 | 18096745.83 | 38969125.99 | 13113226.13 | 41487968 | 147734468.9 | 61771696.14 | 92314277.32 | 70597000.62 |
|  |  | 2.071597432 | 1.800643632 | 0.019559238 |  | 322918653.2 | 237770200.6 | 345607914.2 | 312228901.3 | 191167721.6 | 275738123 | 298145628.3 | 71227839.25 | 351662146 | 360443743.9 | 408324606.9 | 385410521.1 |
|  |  | 1.830729785 | 0.502810042 | 0.019626173 |  | 96650014.47 | 381543453.2 | 102373382.5 | 105644509.8 | 154272016.4 | 292428461.6 | 259402369.4 | 254710174.8 | 76855910.88 | 178155596.8 | 138172560 | 89922368.84 |
|  |  | 1.619156005 | 2.398791379 | 0.019793444 |  | 42351292.92 | 67833833.89 | 52690846.27 | 30496813.85 | 60947298.15 | 113023704.2 | 61876399.03 | 41129353.89 | 243007445.8 | 120078347.8 | 150707591 | 150616068.2 |
|  |  | 1.091677689 | 1.667665433 | 0.019812675 |  | 88242601.84 | 56784590.72 | 113319487.7 | 82056027.88 | 49750513.67 | 79320963.07 | 51237455.17 | 68883991.83 | 107908095 | 77592545.57 | 98861337.28 | 131208447.2 |
|  |  | 1.046246592 | 6.782860097 | 0.019871674 |  | 16062708.21 | 5229058.404 | 9526102.016 | 52798859.81 | 3398164.979 | 2147253.093 | 9364420.282 | 13650191.7 | 36863640.67 | 67892088.64 | 17697608.12 | 71265350.77 |
|  |  | 1.149898082 | 2.531837667 | 0.019937502 |  | 49265034.73 | 43874245.37 | 60914418.27 | 47290520.95 | 34542711.57 | 64452248.79 | 9916954.562 | 16290897.43 | 66653763.39 | 65213539.59 | 104535288.7 | 80590604.71 |
|  |  | 2.080246367 | 1.514445794 | 0.019941349 |  | 565053593.5 | 359262805.6 | 501480664.2 | 438745866.4 | 259649560.5 | 443259850.7 | 262381108.7 | 241841802.5 | 481260680.9 | 434648542.9 | 480774918.7 | 431452325.6 |
|  |  | 1.142388528 | 0.361752185 | 0.020029696 |  | 29612103.18 | 60498286.37 | 85930892.02 | 32509893.36 | 46875941.56 | 96840040.07 | 51904966.94 | 99185431.37 | 37599377.58 | 32986992.49 | 19119241.96 | 16941239.91 |
|  |  | 1.008799187 | 0.476298949 | 0.020174985 |  | 42910525.25 | 41043623.81 | 33050994.47 | 27120588.14 | 45402613.02 | 77867542.82 | 106109630.7 | 71888713.44 | 35849713.65 | 37178287.22 | 39402506.41 | 31063362.65 |
|  |  | 1.223387309 | 1.786164901 | 0.020462178 |  | 25886600.66 | 238219227.4 | 72326750.34 | 24613158.53 | 107759968.6 | 73517353.86 | 60144716.29 | 62012341.47 | 142427104.1 | 166850297.1 | 140284017.1 | 92422421.54 |
|  |  | 1.757147929 | 1.557276168 | 0.020487583 |  | 281873365 | 175098778.7 | 229988085.9 | 282841074.1 | 161926679.8 | 250496141.4 | 116698662.4 | 243371220.7 | 300835347.7 | 320719200.7 | 268200203.5 | 313229726.2 |
|  |  | 2.673267535 | 2.559158554 | 0.020498997 |  | 270853713.6 | 308279043.8 | 295796354 | 230784182.6 | 2866662.032 | 214986078.7 | 217945096.6 | 248249831.5 | 354381511.1 | 599097373.1 | 479255184 | 317852374.8 |
|  |  | 1.937689515 | 0.499566796 | 0.020616958 |  | 106688601.9 | 208129065.7 | 90215883.02 | 115755905.6 | 306421220.3 | 278386908.2 | 205615213.1 | 229732976.3 | 148549652 | 214206782.1 | 76749747.14 | 70130042.13 |
|  |  | 1.545988819 | 0.545662276 | 0.020664015 |  | 224391119.6 | 231308192.9 | 76854301.1 | 182357922.5 | 181389839.8 | 170660627.2 | 166436990.9 | 230074177.9 | 79343472.91 | 81347404.5 | 76690774.81 | 171080194 |
|  |  | 3.452005375 | 1.936605027 | 0.020722367 |  | 1260645590 | 544514915.3 | 907584895.7 | 932925248.1 | 517613285.5 | 675542371.7 | 312722629.3 | 368757777.2 | 921967406.2 | 643205012.1 | 1202109913 | 863147294.1 |
|  |  | 1.144627838 | 1.522680873 | 0.021018 |  | 174845544.6 | 166707511.8 | 236573863.2 | 198545632.5 | 106914573.9 | 127510298.6 | 97620186.41 | 62393639.81 | 127465308.3 | 169021967.7 | 162415455.3 | 141701530.9 |
|  |  | 1.059027834 | 2.124268388 | 0.021089762 |  | 18113592.46 | 94810878.79 | 75981246.36 | 18220872.7 | 51989878.29 | 42684679.51 | 51648681.51 | 16178454.85 | 70612546.97 | 99504577.48 | 113300082.4 | 61780005.03 |
|  |  | 1.462100859 | 4.422924021 | 0.021094581 |  | 28557908.34 | 18433937.55 | 109266895.9 | 24402629.98 | 19369968.98 | 30185758.68 | 17917971.41 | 20797483.03 | 135546884 | 27029565.52 | 120091256.5 | 107749025.7 |
|  |  | 2.958977136 | 0.374086166 | 0.021360912 |  | 69044836.44 | 236616826.5 | 329757633.9 | 103788933.9 | 453160980 | 711907108 | 356189516.7 | 399660818.1 | 158160464.6 | 135329578.4 | 340227426.8 | 84871537.36 |
|  |  | 1.640205528 | 0.167133825 | 0.021433295 |  | 15546130.77 | 30227281.59 | 22607967.59 | 14866822.24 | 66670084.31 | 143734448.3 | 200741241 | 68786786.1 | 18526278.93 | 20668591.05 | 18125104.92 | 22892989.69 |
|  |  | 2.088014865 | 1.797515127 | 0.021601209 |  | 183309669.5 | 154702660.4 | 264241694.1 | 170584311.7 | 190271613.6 | 277542530.2 | 99448251.75 | 248216519.1 | 466999430.8 | 360302900.8 | 306862662 | 331670691.4 |
|  |  | 1.328000158 | 1.635786893 | 0.02166428 |  | 138541007.3 | 108451315 | 125922653.1 | 114183583.9 | 72989607.19 | 121817725.4 | 51198160.77 | 124676090.2 | 162760566.2 | 137189909.6 | 159235623.8 | 147169976.4 |
|  |  | 1.585469489 | 2.911167907 | 0.021706464 |  | 187718232.1 | 63302463.04 | 120161762.8 | 51618334.29 | 42229434.86 | 59820871.57 | 63508411.14 | 39596836.12 | 227991688.1 | 162075769.9 | 127987512.7 | 79187293.09 |
|  |  | 4.658586086 | 3.041839545 | 0.021766619 |  | 864006569.3 | 127026164.3 | 327571660.6 | 682751794.1 | 513462365.1 | 100756930 | 818882628.1 | 154876848.5 | 1420316016 | 1662286094 | 975236291.6 | 772538223.9 |
|  |  | 1.753819853 | 0.500307169 | 0.021825161 |  | 39170777.45 | 631575263.8 | 63405961.64 | 76845474.49 | 312152298.6 | 165620929.7 | 167710065.7 | 263369985.1 | 95636909.99 | 126671428.7 | 114633278.8 | 117764193.2 |
|  |  | 4.616354088 | 2.272827662 | 0.021831099 |  | 2698101701 | 1180284421 | 1152050004 | 888865407 | 432510656.8 | 1074527902 | 747837448.6 | 233166830.7 | 1144450915 | 1153867349 | 1910964552 | 1445609772 |
|  |  | 3.630236828 | 0.378316173 | 0.021913338 |  | 248972215.6 | 385186055.6 | 208203491.3 | 317068058.8 | 998643295.1 | 753513929.5 | 439030293.6 | 894744891.6 | 283829578.6 | 557473003.5 | 238117918.7 | 88037637.53 |
|  |  | 1.193415516 | 1.573215168 | 0.021925987 |  | 135386463.2 | 83478446.67 | 112439004.6 | 109290583.9 | 60194312.61 | 119152952.7 | 54961193.25 | 100196902.3 | 132089401.8 | 128607529.6 | 131170424.7 | 134381551.4 |
|  |  | 2.614239028 | 2.916007058 | 0.021955542 |  | 147356373 | 159781794 | 209011707.3 | 152113550.2 | 126658430.3 | 150235262.1 | 148123737 | 137638061.1 | 387452867.9 | 321226226.6 | 662858699.6 | 269169587.6 |
|  |  | 1.228238295 | 2.365349597 | 0.022168422 |  | 78730086.32 | 93640246.81 | 74076454.66 | 83971632.66 | 20974382.24 | 35965998.32 | 20754659.87 | 70159842.41 | 116494919.2 | 59412403.99 | 84873223.44 | 88947940.88 |
|  |  | 2.32204623 | 2.100988338 | 0.02231925 |  | 375158320 | 299848917.1 | 357941879.5 | 230253094.1 | 176971381.5 | 286698910.4 | 202451991.6 | 126095823.8 | 246602379.1 | 409741942.2 | 544841496.1 | 463255186.8 |
|  |  | 1.041054286 | 0.296093187 | 0.022923024 |  | 17627490.14 | 7037197.768 | 27832721.72 | 68709951.92 | 56084870.92 | 67205124.19 | 53986348.8 | 17137549.83 | 16173864.9 | 16598311.3 | 5806099.963 | 18986353.19 |
|  |  | 1.384011453 | 2.246800494 | 0.023032367 |  | 233423260.5 | 65777359.52 | 80472490.91 | 99883153.82 | 66946918.3 | 79933475.78 | 45369254.9 | 56800246.91 | 110722236.2 | 168818973.7 | 192586321.9 | 87437897.46 |
|  |  | 1.732960443 | 1.51497675 | 0.023268644 |  | 18280644.43 | 30583493.39 | 24415609.01 | 23566555.69 | 11425717.74 | 21130809.03 | 15873376.1 | 20889602.95 | 21868083.44 | 28065350.55 | 30240405.57 | 24843600.06 |
|  |  | 1.052004064 | 1.789082701 | 0.023289721 |  | 114333595.6 | 48829703.99 | 77008414.18 | 79843986.01 | 53761614.67 | 76825762.9 | 37573369.04 | 48548387.8 | 130946932.5 | 86329863.42 | 89372147.04 | 81061620.5 |
|  |  | 1.11964673 | 0.557600665 | 0.023293805 |  | 44782388.3 | 82657813.04 | 54645711.86 | 52825323.19 | 99812096.15 | 127115056.7 | 75605827.92 | 84351432.3 | 49415510.79 | 77258738.84 | 36468468.66 | 52584287.83 |
|  |  | 1.412055841 | 1.873789712 | 0.023313216 |  | 144295321.5 | 159928419.9 | 75958416.65 | 141570226.8 | 39102309.8 | 145321144.1 | 69378459.25 | 69009008.62 | 167284375.7 | 144010198.4 | 144526280.9 | 149058929.2 |
|  |  | 1.505565409 | 2.892961403 | 0.023385342 |  | 119064088.6 | 46404201.89 | 108937930.7 | 66242741.57 | 34626871.39 | 24659508.69 | 86397273.49 | 45840242.49 | 212214710.4 | 136961870.5 | 85601015.1 | 119293643.1 |
|  |  | 1.501166517 | 2.042622553 | 0.023441107 |  | 158193034.4 | 155850620.5 | 115850359.1 | 165495161.3 | 68914240.41 | 126397122.1 | 83411033.28 | 60067064.57 | 130112790.4 | 158450475.1 | 157161137.7 | 246294589.2 |
|  |  | 1.141728981 | 3.147810316 | 0.023462126 |  | 416252719.9 | 199588744.5 | 293786541.2 | 230989058.5 | 33276284.48 | 23443294.16 | 27614404.57 | 15780641.98 | 41898182.6 | 104921687.4 | 111939672.9 | 56382307.12 |
|  |  | 1.908189246 | 1.706594735 | 0.023700044 |  | 337508794.1 | 251632191.2 | 309618147.7 | 292899024.9 | 194521244.3 | 284778950.4 | 223585764.3 | 82411986.99 | 320427683.1 | 350955181.8 | 376075941.2 | 292726534.4 |
|  |  | 1.163297659 | 1.543202161 | 0.023754062 |  | 100775188.4 | 106386580.3 | 138533965 | 129735452.8 | 71597806.26 | 129902183.6 | 86761211.52 | 93242186.21 | 167215517.3 | 168277459.1 | 122673057.4 | 130570818.2 |
|  |  | 1.274338024 | 1.98051484 | 0.023807568 |  | 63639131.97 | 52221887.34 | 86567550.09 | 64112383.82 | 46177357.41 | 69565476.43 | 29879047.09 | 89537665.3 | 158177130.8 | 98381933.18 | 102868927 | 106308980 |
|  |  | 1.38479077 | 2.35005026 | 0.024194005 |  | 131524734.6 | 97074096.24 | 102567685.6 | 43082416.57 | 77722304.93 | 110299553.2 | 2088949.826 | 28029068.75 | 123311609.9 | 121681197.6 | 130200457.1 | 137446409.3 |
|  |  | 1.319370504 | 2.110245527 | 0.024240242 |  | 203489854.6 | 98481407.65 | 100047652.6 | 54362110.21 | 53341064.4 | 61252735.11 | 76403961.21 | 65117276.83 | 150919841.6 | 132462340.1 | 184003500.7 | 73079929.97 |
|  |  | 1.179742213 | 0.422532253 | 0.024328752 |  | 60067540.75 | 77115153.56 | 98532533.73 | 85558924.53 | 92465983.65 | 79847078.98 | 91513891.62 | 65420917.25 | 9791522.723 | 54799773.34 | 64799395.48 | 9727153.262 |
|  |  | 4.951233838 | 1.528192657 | 0.024356114 |  | 2250917398 | 2150216118 | 1701731064 | 2562057593 | 1616752560 | 2546603647 | 1392386316 | 1407086405 | 2260550325 | 2666702320 | 2832737153 | 2880554246 |
|  |  | 1.023721034 | 0.343825399 | 0.024738096 |  | 17948540.33 | 75162163.11 | 21729778.12 | 19136608.7 | 42060162.49 | 35099775.23 | 54935058.06 | 77544847.26 | 8748164.238 | 38266076.42 | 13062779.93 | 12002482.06 |
|  |  | 3.860073479 | 0.048398635 | 0.024809661 |  | 37603637.06 | 19478630.62 | 31281442.76 | 28089050.09 | 806182141.6 | 28131751.45 | 683547748.6 | 719425763.5 | 23594567.69 | 29741414 | 24730783.81 | 30214891 |
|  |  | 1.278004293 | 1.699162662 | 0.025020668 |  | 187189837.8 | 121156054.3 | 123477686.8 | 129795171.5 | 92343435.98 | 111159918.7 | 85652273.48 | 69784503.34 | 149283502.9 | 105184778.1 | 199632218.7 | 155797169.5 |
|  |  | 1.253326485 | 1.956778458 | 0.025033769 |  | 186879441 | 99283501.71 | 83097430.12 | 123529425.7 | 36658356.4 | 58899474.95 | 36955866.38 | 93501688 | 100764318.3 | 133656179.4 | 80317508.97 | 127524031.3 |
|  |  | 3.115511893 | 2.240358322 | 0.025406929 |  | 486247681.7 | 416082577.6 | 412634846.3 | 214360699.3 | 238914791.6 | 487165209.7 | 208192510.9 | 232143962.6 | 957409060.8 | 496094515.9 | 571698749.5 | 587988530.4 |
|  |  | 6.186132301 | 1.823995312 | 0.025426759 |  | 3036605176 | 2699469440 | 2752712837 | 3180834250 | 1470342570 | 2721060637 | 1554485043 | 1066532410 | 2566258797 | 2605173827 | 3853178848 | 3401211876 |
|  |  | 12.31147768 | 2.435827629 | 0.025507892 |  | 10895516785 | 6373157610 | 5667014398 | 7728710890 | 4902526721 | 6527540769 | 3970547736 | 879724036.9 | 11212395836 | 7579730518 | 7085380380 | 13778593450 |
|  |  | 1.245554039 | 0.389882834 | 0.025729968 |  | 9411534.544 | 8720478.695 | 11433475.65 | 14946156.99 | 116342238.1 | 120017461.6 | 56858895.59 | 70455735.11 | 43448320.34 | 59376979.49 | 15311759.24 | 23653319.42 |
|  |  | 1.032942264 | 2.71805831 | 0.025781029 |  | 78699644.23 | 25179569.81 | 97698413.79 | 57189940.37 | 18549352.62 | 31921530.92 | 19778348.74 | 22525403.28 | 46267022.45 | 36775046.66 | 94298121.46 | 74826678.61 |
|  |  | 1.771710647 | 0.099636729 | 0.0259524 |  | 8698090.987 | 12849769.54 | 13330538.71 | 9213958.109 | 148734716.7 | 131812239.9 | 145799395.8 | 9552551.022 | 13776704.52 | 11446240.36 | 11126318.98 | 7082276.913 |
|  |  | 1.744917403 | 0.423898213 | 0.026110937 |  | 93244639.63 | 366517172.6 | 268509640.8 | 48698705.39 | 87809187.97 | 234111986.3 | 225006659.8 | 247950630.6 | 62063256.53 | 115225264.1 | 86767576.5 | 72891463.18 |
|  |  | 1.145764524 | 0.304312153 | 0.026306619 |  | 5475157.346 | 147103159.4 | 11967123.68 | 2225998.715 | 70359171.75 | 54307259.38 | 41878824.48 | 92908235.54 | 10901153.53 | 51852015.84 | 10411723.82 | 5789957.214 |
|  |  | 2.04201798 | 0.305487447 | 0.026338459 |  | 55474385.03 | 383825784.4 | 110793464 | 42057260.19 | 174384076.1 | 325166347.4 | 190370290.6 | 110754866.5 | 24678098.36 | 94818330.71 | 72413731.78 | 52686178.3 |
|  |  | 4.267863298 | 1.644067941 | 0.026366894 |  | 1589148337 | 858760969.1 | 1303213433 | 1262445097 | 533666029 | 1179703607 | 782307281.1 | 1364193852 | 1499876969 | 1834003535 | 1636747787 | 1375261494 |
|  |  | 1.337856988 | 1.719865857 | 0.026442257 |  | 119259370 | 340460595.2 | 132727547.8 | 102503294.7 | 87807363.88 | 89297124.85 | 87707464.44 | 108969256.9 | 185482667.1 | 206855649.6 | 144813644.3 | 105701580.3 |
|  |  | 3.625932098 | 0.519649496 | 0.026460439 |  | 543122524.2 | 643396976.6 | 511829487.9 | 505053611.1 | 763882826.1 | 1200485610 | 726168932.7 | 1280399657 | 649534722.2 | 643693005 | 409862951.1 | 360404747.2 |
|  |  | 1.28610769 | 1.528388451 | 0.026861546 |  | 204167736.5 | 158399992.4 | 184679203.6 | 178298310.2 | 127121278.2 | 164661514.6 | 122725612.1 | 63872191.36 | 176972827.6 | 200982327 | 177535077.2 | 175661146.9 |
|  |  | 1.247144499 | 2.398661149 | 0.026970018 |  | 64029355.83 | 105224772.8 | 108234850.8 | 53889863.44 | 40348197.95 | 62214400.53 | 63571013.62 | 9426632.337 | 142512633.8 | 122835630.2 | 67398714.7 | 88362558.94 |
|  |  | 1.012275084 | 0.279921221 | 0.027099788 |  | 21625005.74 | 50214968.33 | 19327326.24 | 16359084.64 | 44985842.65 | 44452537.69 | 87803776.38 | 28391861.53 | 14736203.28 | 15480125.19 | 13491043.34 | 13853953.7 |
|  |  | 1.107608286 | 0.160227275 | 0.02716716 |  | 3443364.856 | 97477451.98 | 5151230.155 | 3804594.967 | 51023549.41 | 71014051.27 | 53702772.28 | 8492835.526 | 6662172.72 | 6328699.784 | 6328125.579 | 10200186.94 |
|  |  | 1.967498781 | 4.657994826 | 0.027243104 |  | 66508359.52 | 60677509.15 | 146631991.6 | 64269310.18 | 27151992.72 | 57585936.9 | 81438643.25 | 9318875.809 | 149514972.2 | 224184891.3 | 100224087.5 | 343532941 |
|  |  | 1.018945756 | 1.999686723 | 0.027288792 |  | 77925016.21 | 45462058.72 | 54146730.62 | 60933901.12 | 38564854.63 | 57379150.96 | 38221701.92 | 37710761.66 | 58102705.61 | 103230693.6 | 66439865.76 | 115925828.5 |
|  |  | 1.068499983 | 1.771314707 | 0.02735943 |  | 85777730.08 | 74819278.49 | 101023570.1 | 50357794.22 | 68775297.63 | 77100506.1 | 68271827.89 | 41569638.31 | 74735539.46 | 116323457.4 | 148780616 | 113116148.3 |
|  |  | 2.867357505 | 1.80602845 | 0.027531793 |  | 368124343.7 | 393146492.8 | 435047377.9 | 474100923.2 | 339221460.2 | 630350585 | 343012709 | 253849382.8 | 690777311.6 | 713482407.8 | 887399649.6 | 537365248.1 |
|  |  | 1.365747266 | 2.969567542 | 0.0276429 |  | 332431624.5 | 112620259.9 | 256240583.3 | 154989373.3 | 31294971.39 | 59602869.61 | 51209072.71 | 8475522.411 | 71601720.12 | 81225806.39 | 172478434.3 | 121858753.9 |
|  |  | 1.318526106 | 0.480257666 | 0.027740291 |  | 4560264.792 | 1644359.309 | 4015358.441 | 2159393.985 | 1693712.243 | 1563154.39 | 1070018.418 | 1884343.174 | 1026868.463 | 1135354.318 | 653224.5977 | 167542.5898 |
|  |  | 1.289699564 | 1.546880811 | 0.027754293 |  | 141516485.5 | 195638626.2 | 131766045.2 | 102835148.8 | 69528757.76 | 129779290.5 | 81394449.22 | 139047716 | 134484499.1 | 178566896.9 | 172735566.4 | 163516588.5 |
|  |  | 1.68842638 | 1.925173815 | 0.027858227 |  | 92961845.85 | 83622683.34 | 158301725 | 90181074.59 | 130344220.9 | 156774428 | 66308537.68 | 137925183.5 | 335053160.8 | 229785371.9 | 191814942.6 | 189285241.3 |
|  |  | 1.194063312 | 0.561488204 | 0.027996341 |  | 123340970.5 | 96813082.58 | 98792634.54 | 75932356.86 | 132272837.3 | 134809241.4 | 100700978.3 | 100895496.5 | 63325376.52 | 63680153.16 | 104944665.3 | 31207284.29 |
|  |  | 1.350506606 | 2.037561101 | 0.028009954 |  | 125252289.1 | 98225585.14 | 104956820.3 | 102086959.3 | 51520673.52 | 91190519.6 | 43662067.71 | 76766015.55 | 195814546.2 | 102405107.8 | 117517586.8 | 120425112.8 |
|  |  | 1.568159859 | 1.858519284 | 0.028111431 |  | 214962727 | 154271843.8 | 148472291.2 | 216538221.2 | 43602441.32 | 169219121.6 | 78441737.34 | 114264313.6 | 161783076.3 | 181727114.5 | 182403209.2 | 227767490.6 |
|  |  | 2.065526507 | 2.114414342 | 0.028511485 |  | 233049849.8 | 258315099.8 | 334184476.9 | 165010940.2 | 76222092.96 | 209722232.3 | 157910477.5 | 155580351.5 | 261945568 | 455514405.5 | 329069725.5 | 220924588.5 |
|  |  | 4.400565033 | 0.441501659 | 0.028577581 |  | 340632381.7 | 1381494925 | 649625343.8 | 303282170.2 | 1838898430 | 1104851402 | 1103933072 | 797905573 | 766915136.6 | 483653711.7 | 380115148.1 | 508651354.2 |
|  |  | 3.578432934 | 2.178995094 | 0.028704526 |  | 1348680833 | 571405733.3 | 528524692 | 939819760.5 | 399031197.5 | 366677414 | 859110768.3 | 326130714.7 | 758191196.1 | 840870862 | 1418407728 | 1233640899 |
|  |  | 1.721817955 | 2.684634148 | 0.02894151 |  | 146555832.3 | 120990977.4 | 56902758.77 | 319773694.1 | 1285654.479 | 77350722.14 | 49320806.75 | 117862912.5 | 204226839.7 | 133142403.9 | 215972925.6 | 106594854.4 |
|  |  | 1.59439879 | 2.343427889 | 0.029476844 |  | 43790529.58 | 104532857.6 | 93617962.54 | 40900843.96 | 67124184.95 | 123396486.8 | 60773252.06 | 34965063.78 | 147011238.4 | 120683717.6 | 153425479.8 | 249706859.3 |
|  |  | 1.62514179 | 2.754415774 | 0.029633998 |  | 134867229.5 | 56959285.69 | 98837160.4 | 166016743.1 | 69968898.88 | 52283185.81 | 90646564.44 | 44181611.39 | 104665650.3 | 216256193 | 121690760.3 | 265493321.2 |
|  |  | 10.49175625 | 0.251586711 | 0.029664301 |  | 905835153.6 | 7838355030 | 2671887992 | 913190915.1 | 6381069644 | 8879290343 | 2224923782 | 4379714181 | 1230918484 | 2090722308 | 1284607025 | 894695114.4 |
|  |  | 2.650559892 | 1.50645071 | 0.029887225 |  | 934327838.6 | 882293573.6 | 564304678.8 | 682302377.1 | 523421685.4 | 680722389.9 | 506634800.3 | 417793163.5 | 859611281.8 | 598947754.7 | 779436137.6 | 968593686.2 |
|  |  | 1.126683119 | 1.605274548 | 0.029897567 |  | 120580298.4 | 99741146.99 | 120764477.7 | 125296541.9 | 71430523.47 | 116189552.3 | 89140347.63 | 46997776.2 | 109286061.3 | 119193822 | 143868805.9 | 147372108.3 |
|  |  | 1.827401516 | 2.478819802 | 0.030260283 |  | 239748213.5 | 88201418.24 | 132433098.5 | 122918390.2 | 68690066.8 | 60448216.11 | 47681079.25 | 150511375 | 278756607.6 | 244317290.3 | 119887052.2 | 168432963.2 |
|  |  | 1.105328756 | 0.293670292 | 0.030631386 |  | 6461099.108 | 23291013.87 | 12258853.17 | 19627842.18 | 72121318.04 | 103225359 | 24098954.42 | 61556405.76 | 15005978.49 | 20947327.8 | 19516550.26 | 21178687.94 |
|  |  | 1.457107747 | 2.369903669 | 0.030664461 |  | 140340296.1 | 86942040.07 | 75939958.29 | 133371854.5 | 56121797.17 | 66872965.64 | 84441911.78 | 44117177.9 | 113211003 | 88000973.2 | 215935575.3 | 179010846.5 |
|  |  | 1.256145894 | 1.568484195 | 0.0312438 |  | 107175229.2 | 494375152 | 460162533.9 | 119462586 | 116182059.1 | 135857187.9 | 116007538.3 | 95206846.93 | 139464391 | 228134618.8 | 148944784.8 | 210062205.8 |
|  |  | 6.464614531 | 3.036555524 | 0.031314825 |  | 1745952177 | 891146387.5 | 828202725.8 | 1565264047 | 857632782.1 | 676468847.6 | 1409418079 | 472822801.4 | 4249309718 | 2026113888 | 1548436920 | 2550053193 |
|  |  | 1.258324821 | 1.69176774 | 0.031357349 |  | 97601367.86 | 75641438.86 | 114630158.1 | 81108379.29 | 58221024.17 | 118533165.3 | 40716498.29 | 100303580.7 | 154758695 | 120179957.9 | 138019361.9 | 124642241.5 |
|  |  | 1.067967747 | 1.549832081 | 0.03145946 |  | 131384427 | 205543783 | 151168615.3 | 95955377.49 | 80092664.53 | 112339250.1 | 79421234.44 | 40807662.31 | 113516112.2 | 112288005.2 | 129903176.9 | 128864461.6 |
|  |  | 1.223393141 | 1.624386928 | 0.03160584 |  | 126865298.1 | 78936508.52 | 127222167.1 | 90998821.35 | 61550964.82 | 130862519.8 | 49112385.48 | 91995160.71 | 144195154.1 | 124695090.9 | 138271427.1 | 134605530.7 |
|  |  | 2.681368005 | 0.313851223 | 0.031719394 |  | 335909516.5 | 411885159.6 | 484915261.4 | 274944543.3 | 304042165.9 | 502656597.9 | 469980705.8 | 145908654.7 | 142333253.7 | 162987303.4 | 27351671.97 | 113808793.8 |
|  |  | 1.293903335 | 1.832373032 | 0.031933155 |  | 67764677.47 | 104522087.5 | 87300114.27 | 79657166.28 | 55327184.16 | 76506754.33 | 93364378.42 | 82575399.56 | 158710796.9 | 193678659.5 | 109255806.1 | 102310995.6 |
|  |  | 1.181981483 | 1.939833462 | 0.031972004 |  | 159517481.2 | 47957185.26 | 63679392.12 | 124175731.6 | 45979971.02 | 68494033.63 | 24864910.52 | 81248447.79 | 112865085 | 100185778.1 | 74052974.55 | 140798910.5 |
|  |  | 1.39890711 | 2.144951758 | 0.031981398 |  | 109923849.3 | 89761351.22 | 175672970.3 | 106231098.4 | 36680700.59 | 95668398.88 | 68402397.18 | 67670380.83 | 122202715.7 | 124362661.3 | 218031105.6 | 111155495.5 |
|  |  | 1.330626491 | 2.670696199 | 0.032275781 |  | 50007039.77 | 53754937.22 | 108526169.8 | 65041773.77 | 37948384.24 | 67095464.71 | 38355935.28 | 24224011.83 | 174758087.3 | 82894689.31 | 69430534.78 | 120588923.6 |
|  |  | 1.635974359 | 0.092545532 | 0.032349777 |  | 270121249.4 | 3570817.397 | 4106017.368 | 280349457 | 125526096.5 | 5232739.254 | 131364034.2 | 163182065.3 | 4943427.232 | 10502600.63 | 17455580.82 | 6458462.67 |
|  |  | 1.947846352 | 2.42864478 | 0.033076804 |  | 290028050.4 | 257064750 | 312641327 | 163106503.2 | 308412626.9 | 50835550.45 | 40174099.2 | 93363454.88 | 275431851 | 291807200 | 331376230.2 | 298186213.2 |
|  |  | 1.065133535 | 2.050103095 | 0.033207394 |  | 72629510.06 | 44380032.68 | 120505461.8 | 12256830.62 | 11943083.09 | 70070512.05 | 18122728.84 | 45474741.89 | 74250733.29 | 64422820.69 | 78064965.58 | 81779177.18 |
|  |  | 2.765294425 | 0.069682734 | 0.033287611 |  | 22051384.26 | 26784469.52 | 16787704.1 | 34012359.41 | 13491686.47 | 533253455.9 | 406918147.6 | 363535620.9 | 22119782.34 | 7518595.005 | 33638967.57 | 28508676.96 |
|  |  | 4.539165211 | 2.310787169 | 0.033289698 |  | 1813524716 | 864683570.1 | 1691817686 | 2628913037 | 164951438 | 903101289.3 | 749644686.4 | 550605572.4 | 1283578815 | 933028374.8 | 1229000464 | 2027036499 |
|  |  | 1.007146901 | 0.658405365 | 0.033580105 |  | 49284797.77 | 74560955.01 | 32777289.36 | 90311660.38 | 123838877.2 | 134905903.9 | 79623370.65 | 114245586.1 | 89099027.59 | 84698469.2 | 57379212.96 | 66826603.6 |
|  |  | 2.399849805 | 12.61726345 | 0.033663361 |  | 34664778.53 | 9951222.862 | 21028189.14 | 35399287.83 | 14487979.49 | 19347563.18 | 23294581.62 | 16291518.62 | 387381766.5 | 17460853.12 | 241735100.8 | 279802491.2 |
|  |  | 1.489588869 | 1.586654961 | 0.033694825 |  | 164861166.7 | 120313995.2 | 137180984.5 | 116281834.1 | 84654679.09 | 107587060.2 | 113219015.6 | 195042294.8 | 194915062.1 | 168059261.9 | 208203486.8 | 222947835.7 |
|  |  | 1.740383312 | 2.223963876 | 0.033718319 |  | 91647234.37 | 136146511.5 | 128545652.8 | 111745968.1 | 112816956 | 137769355 | 63520622.02 | 93275851.84 | 131698767.4 | 176887551.6 | 298842014 | 298576264.2 |
|  |  | 1.565018249 | 0.092592818 | 0.033777372 |  | 11235362.15 | 152132487.5 | 2351891.002 | 5737882.857 | 120098210.4 | 117337557.8 | 99106831.64 | 2052603.147 | 15542020.34 | 4438586.014 | 5392518.541 | 5978359.016 |
|  |  | 3.074750313 | 1.542382764 | 0.033789194 |  | 1429190940 | 864577361 | 1323139645 | 1250504304 | 513630037.8 | 961314538.5 | 595580940.4 | 460226188.8 | 1015530980 | 853349297.7 | 1104106625 | 930400908.6 |
|  |  | 1.042041584 | 1.709247277 | 0.033840145 |  | 90607277.13 | 46403004.86 | 90456900.59 | 53728795.16 | 39275215.55 | 50993170.65 | 28116412.16 | 85534936.08 | 78987135.98 | 85298748.41 | 99868968.91 | 84394397.52 |
|  |  | 1.011911098 | 3.033807262 | 0.033991353 |  | 34980642.1 | 43395267.68 | 30147618.63 | 26107496.16 | 10473568.83 | 15458714.12 | 36486718.98 | 19229305.32 | 71383741.88 | 79631815.36 | 19961380.27 | 76728289.99 |
|  |  | 1.042731297 | 0.199029761 | 0.034222931 |  | 11194959.13 | 23010570.61 | 16832720.56 | 26048570.25 | 49788653.47 | 91410314.26 | 19025465.98 | 45344608.63 | 15472154.77 | 9961630.456 | 8669613.032 | 6810959.027 |
|  |  | 1.538241816 | 0.62720486 | 0.034242931 |  | 277697279.9 | 4181840.648 | 282541691.5 | 146564009.2 | 200909988.8 | 249181573.5 | 192511230.4 | 263779644.2 | 115894387.1 | 130297522.4 | 105317346.7 | 216978213.5 |
|  |  | 1.336299737 | 7.296793326 | 0.034292599 |  | 3264960.97 | 5956042.119 | 6860996.713 | 3506128.92 | 6709532.9 | 8846956.649 | 8716291.326 | 24465255.39 | 6270610.768 | 116874415.7 | 106632169.3 | 125854181.9 |
|  |  | 6.844506028 | 1.731061694 | 0.034416159 |  | 4029717647 | 4871581039 | 2298418993 | 3823473475 | 1681833565 | 2808565015 | 2027059034 | 3148315556 | 3392786639 | 3627419784 | 5810915171 | 3900928079 |
|  |  | 1.00230761 | 7.546550867 | 0.034750438 |  | 4055165.789 | 5381957.291 | 5807350.121 | 4361183.951 | 3405527.845 | 12356243.9 | 3742646.849 | 5090205.415 | 71822716.86 | 52161342.25 | 57399607.78 | 4220914.225 |
|  |  | 1.182933379 | 1.620849208 | 0.035037493 |  | 165180757.8 | 103341557 | 72460714.26 | 170355266.5 | 79949586.71 | 122315137.4 | 57586258.35 | 67612354.1 | 125502287.3 | 101678444.5 | 147283605.7 | 156304352.4 |
|  |  | 1.829668191 | 1.662204007 | 0.035053233 |  | 402874679 | 275662987.1 | 238764632.5 | 345676552.2 | 232205796.4 | 244349914.7 | 234702079.4 | 210022929.6 | 242110061.3 | 355953693.9 | 503869930.2 | 429422819.2 |
|  |  | 2.787919281 | 0.470854303 | 0.035230597 |  | 126385438.6 | 401693989.6 | 329141638.7 | 300856296.2 | 498841447.6 | 753826005.1 | 449577859.6 | 394339337.3 | 290350131 | 391455093.9 | 221966222.6 | 83414456.82 |
|  |  | 1.569343469 | 1.832570568 | 0.03533065 |  | 33859856.1 | 474119448.2 | 289746507.3 | 408171451.7 | 195885809.3 | 116342364.1 | 127501855.6 | 37218680.96 | 249533789.8 | 245293417 | 197698724.7 | 181516236.7 |
|  |  | 3.008677775 | 0.342489946 | 0.035368098 |  | 52173405.99 | 151187430.5 | 83517590.09 | 204057465.7 | 640573721 | 425071001.6 | 469451456.9 | 179691617.4 | 98614481.25 | 269065962 | 79954733.13 | 139662403.3 |
|  |  | 1.797070144 | 2.958968745 | 0.035685078 |  | 135805303.7 | 88756482.14 | 74185798.91 | 77392974.15 | 53678346.3 | 86725447.84 | 81302150.84 | 43997326.16 | 147370272.3 | 118818910.1 | 331096007.9 | 188922484.4 |
|  |  | 1.137520845 | 1.972564228 | 0.03569702 |  | 112306005.8 | 49399943.86 | 74226521.8 | 72704028.19 | 59475264.24 | 96497148.95 | 31473078.27 | 22716614.87 | 92938196.01 | 116895262.2 | 121059858.7 | 83664936.2 |
|  |  | 1.208357457 | 0.298072721 | 0.035878929 |  | 12643991.85 | 177266375 | 23342210.21 | 8265247.723 | 61306601.47 | 40985613.88 | 57875230.09 | 119703446.8 | 10915504.76 | 37829057.8 | 21959709.26 | 12717606.45 |
|  |  | 1.196077626 | 1.896695282 | 0.036418148 |  | 164976411.3 | 106451596 | 154757110.1 | 103881552.2 | 38081740.01 | 97027030.49 | 80826071.39 | 28461693.05 | 104751148.4 | 90271182.58 | 145998990.4 | 122524433.3 |
|  |  | 1.889643232 | 0.518918459 | 0.036638426 |  | 399292511.4 | 850538779.4 | 413005264.6 | 494681451.2 | 148115641.6 | 323858505 | 252454705.3 | 358000452.1 | 168414467.9 | 120916979 | 162191515.2 | 110169584.4 |
|  |  | 1.37048841 | 2.117631585 | 0.03667274 |  | 75554934.52 | 141297381.7 | 72422172.87 | 83357230.22 | 53737264.09 | 84290372.88 | 40947171.63 | 77152769.13 | 120500345.8 | 94473932.23 | 207602134.4 | 119807435.8 |
|  |  | 1.046182014 | 0.332887272 | 0.036682327 |  | 22788159.81 | 16436006.92 | 85258057.98 | 19041463.83 | 20310799.93 | 88836107.17 | 63676723.23 | 81577536.38 | 31753413.55 | 14007971.77 | 17091534.34 | 21833990.71 |
|  |  | 1.043571514 | 1.514478442 | 0.036862612 |  | 224873361.2 | 86549149.65 | 142278934.5 | 193458018.1 | 64063204.99 | 94467358.28 | 48340703.4 | 91841727.35 | 121814183.4 | 91011518.19 | 106884302 | 132684386 |
|  |  | 3.725786264 | 1.69320668 | 0.037120994 |  | 7163165.517 | 8153573.104 | 10662032.29 | 9510004.051 | 5564829.431 | 8598438.939 | 6280712.671 | 10691226.46 | 11071353.01 | 12755979.63 | 17977443.36 | 10913565.34 |
|  |  | 1.170028728 | 2.734672875 | 0.037252612 |  | 21613676.99 | 41798740.3 | 25206683.14 | 17240774.9 | 10779829.26 | 18657007.17 | 24301380.02 | 56640324.07 | 72275186.79 | 34849079.06 | 101498458 | 93226476.87 |
|  |  | 1.488379871 | 1.930277268 | 0.037323706 |  | 163706545.5 | 132320187.4 | 203420355.5 | 145040530.8 | 92616206.79 | 154216292.1 | 100494475.1 | 25546399.26 | 226427719.5 | 188025719.4 | 134677734.7 | 170617822.6 |
|  |  | 3.029904371 | 1.931783028 | 0.037483441 |  | 450623002.6 | 597467539.8 | 365049440.4 | 332217008.7 | 216584300.6 | 302560811.8 | 323876471.5 | 597441267.5 | 913140084.1 | 679530401.3 | 451009423.8 | 738981779.6 |
|  |  | 1.733641807 | 2.885237145 | 0.037754927 |  | 69358982.72 | 113636901.5 | 63102753.91 | 58441155.39 | 71633336.73 | 98536933.17 | 63808310.4 | 28119458.6 | 250690313.6 | 140444998.7 | 89821844.01 | 275257841.3 |
|  |  | 1.382067514 | 1.721156227 | 0.037889742 |  | 141699964.5 | 192824646 | 116018527.6 | 155222357.4 | 105338826 | 135757405.5 | 162506395.2 | 36987658.43 | 212636825.5 | 162001952.3 | 173342862.9 | 210343072.2 |
|  |  | 2.403957193 | 2.000313365 | 0.037991745 |  | 462447736.2 | 243161149 | 346802056 | 992689966.1 | 206756551.9 | 336922469.4 | 302602517.1 | 174357066.3 | 281440110.5 | 507500880.4 | 542230840.2 | 710425210.4 |
|  |  | 1.686212995 | 0.161460579 | 0.038251722 |  | 23441541.58 | 52001731.54 | 42749091.12 | 17761770.42 | 83589441.71 | 91859854.32 | 68943765.72 | 227396135.1 | 8969385.215 | 37979889.89 | 18475628.04 | 10750453.83 |
|  |  | 3.095282189 | 1.613107604 | 0.038290387 |  | 472856606.1 | 362146904.7 | 284794331.9 | 969294375.8 | 407461320.5 | 405767021.9 | 404613821.7 | 835406633.3 | 856020434.5 | 941474282.5 | 694380795.9 | 820235734.1 |
|  |  | 1.001383409 | 2.105740965 | 0.038329551 |  | 17861859.18 | 60969402.59 | 22856409.83 | 32160945.32 | 22008786.46 | 37667383.16 | 62338540.83 | 32813032.8 | 103966829.4 | 57046910.53 | 106655729.5 | 58357652.09 |
|  |  | 2.318967704 | 0.306062658 | 0.038338544 |  | 200429155.5 | 112159985.4 | 47979745.87 | 170210828 | 475833090.6 | 166914380.1 | 182964996.2 | 344785830.4 | 32016108.57 | 143995688.6 | 77123061.65 | 105110961.4 |
|  |  | 2.44748985 | 0.206531356 | 0.038586025 |  | 80833909.91 | 103106403.5 | 56298821.25 | 65478836.53 | 78823941.49 | 491765845.6 | 253325673.4 | 324316333.2 | 47911053.54 | 38116966.15 | 91126974.19 | 59990875.29 |
|  |  | 1.56855459 | 2.976146567 | 0.038688136 |  | 154697980.5 | 80792233.76 | 109033219.5 | 133018936.8 | 19405350.29 | 49502990.61 | 20790523.8 | 89082526.92 | 108377141.8 | 75073102.5 | 136360554.1 | 212268826.5 |
|  |  | 1.114434326 | 0.618549757 | 0.038749403 |  | 92275337.33 | 87320711.45 | 93261227.23 | 80445036.67 | 76063363.87 | 155529628.6 | 106205984.5 | 121058407.8 | 72141124.26 | 67871786.26 | 75201211.02 | 68612002.28 |
|  |  | 1.12336318 | 2.832783696 | 0.038860794 |  | 90256646.67 | 44345869.96 | 43745353.2 | 76079719.95 | 40316723.69 | 39864360.54 | 12825078.38 | 24041441.82 | 93926141 | 80866850.77 | 32144477.02 | 124633076.7 |
|  |  | 1.259706341 | 1.504626976 | 0.038938071 |  | 256054271.3 | 140869070 | 217647421 | 178933925.1 | 94769607.77 | 178579112.2 | 100145391.5 | 88709317.39 | 191161542.5 | 160370911.1 | 167624975.6 | 176286318.4 |
|  |  | 6.276107865 | 0.543783007 | 0.039106294 |  | 1879820196 | 2325642297 | 1703649404 | 1685008911 | 2462627069 | 4049781985 | 2448679112 | 4237130556 | 2344667697 | 2268402748 | 1368085177 | 1195811445 |
|  |  | 5.127712083 | 2.227677254 | 0.039360529 |  | 1055043616 | 1697162564 | 947081664.5 | 1318333541 | 1192212338 | 631756802.7 | 915315216.6 | 843962955.9 | 2989416717 | 2222184288 | 1670882845 | 1099834683 |
|  |  | 2.174703056 | 3.925239905 | 0.039701344 |  | 62067370.4 | 46270904.18 | 41263524.53 | 76299055.4 | 67327613.29 | 41132582.38 | 114214080.9 | 45088052.13 | 191666956.6 | 100482827.9 | 328709446.9 | 430172146.4 |
|  |  | 1.525728703 | 2.98835019 | 0.03974366 |  | 261476098.2 | 161007467.5 | 135926427 | 84827196.32 | 44090587.87 | 54732987.16 | 45184332.64 | 41708234.15 | 108409245.3 | 64283050.26 | 153216123.5 | 229076448.7 |
|  |  | 2.138852634 | 3.165754609 | 0.039798391 |  | 202049123.7 | 81167441.78 | 419681855.1 | 124920603.3 | 84994364.4 | 113195474.3 | 85825160.31 | 62365249.98 | 455408285.6 | 318425948.5 | 169070330.8 | 153650304.5 |
|  |  | 1.085448096 | 0.652149046 | 0.039861859 |  | 113108706.1 | 117008464.7 | 105049208.7 | 139557903.5 | 103290167.5 | 113998922.4 | 131414166.8 | 98036429.25 | 54002794.62 | 109211933.6 | 73167001.84 | 54959129.71 |
|  |  | 1.337096719 | 2.812865807 | 0.040036187 |  | 70059135.53 | 138977639.8 | 106627026.8 | 70549481.75 | 1536601.773 | 959278.9679 | 128063055.9 | 49376991.95 | 101444688.1 | 131324340 | 143588382.2 | 129778210.6 |
|  |  | 2.346697776 | 0.408522239 | 0.040108318 |  | 78658781.03 | 793983274.3 | 107643435.2 | 96837076.22 | 399945732.8 | 301197221.8 | 180680508.4 | 495635625.9 | 63668589.37 | 250005911.4 | 128828810.1 | 120219359.6 |
|  |  | 1.049082371 | 0.556423042 | 0.040249592 |  | 38201107.04 | 23743261.6 | 44734401.15 | 77362640.04 | 75291585.6 | 96161752.23 | 111510243.1 | 51330900.78 | 43001323.71 | 62596260.97 | 43926374.62 | 36485193.31 |
|  |  | 2.564997664 | 0.610690558 | 0.040251081 |  | 179584160.5 | 1568101511 | 342006925 | 276742131.3 | 529975461.9 | 794814241.7 | 528893310 | 618492166.8 | 335058300.7 | 480741208.7 | 203969768.4 | 489964763 |
|  |  | 1.100805664 | 0.196014877 | 0.040265561 |  | 5342860.678 | 4735630.007 | 5595378.027 | 13188546.95 | 86363339.91 | 49817702.31 | 48810051.1 | 12529371.73 | 7617360.341 | 8819789.093 | 6305344.615 | 15974455.54 |
|  |  | 3.218493343 | 0.539040224 | 0.040394736 |  | 487197077.2 | 615293144.5 | 438275605.3 | 441295014 | 635099224.5 | 1036185029 | 630609012.3 | 1122267048 | 597401900.7 | 593200366.6 | 346509949.5 | 308647924.8 |
|  |  | 1.779910185 | 2.48420148 | 0.040472815 |  | 140522577.1 | 106173266.3 | 255679040.9 | 117631242.7 | 43870649.74 | 125984449.6 | 143187678 | 43718411.93 | 282413594 | 284966083.6 | 219410988 | 99476008.93 |
|  |  | 3.344281375 | 0.406587681 | 0.040532169 |  | 130323205.6 | 315563007.1 | 243752662.7 | 240890378.8 | 680351781.4 | 549360532.8 | 468844992.4 | 1047080194 | 45797842.48 | 265916084.3 | 322391665.1 | 482236791.9 |
|  |  | 1.567060749 | 0.262672328 | 0.040986112 |  | 77175664.06 | 72241991.85 | 93580330.46 | 92117765.09 | 97764115.86 | 86286394.11 | 70742722.39 | 215305077.9 | 31036671.7 | 24396539.44 | 23714883.4 | 44333723.12 |
|  |  | 1.05082074 | 0.336810569 | 0.041302782 |  | 135223039.3 | 67364744.45 | 104810944.5 | 118551490.3 | 50426222.19 | 94140706.81 | 53725917.84 | 26097123.57 | 26475999.48 | 16512524.1 | 13350842.48 | 19237547.61 |
|  |  | 1.74176446 | 1.616768103 | 0.041337541 |  | 436175617.8 | 186611971.9 | 200624630.2 | 425032061.4 | 131679006.5 | 228352285.6 | 135723132.7 | 203205795.4 | 244019779.9 | 208448446.3 | 353813334.2 | 323775028.8 |
|  |  | 6.775769616 | 1.507575323 | 0.041393728 |  | 8012291124 | 4403714889 | 6425504098 | 4538526644 | 2015046174 | 4959599124 | 3587225863 | 2893608928 | 5451659511 | 4429078208 | 5192281466 | 5212130550 |
|  |  | 5.036866521 | 0.120101738 | 0.041529796 |  | 290374146.2 | 1719633599 | 660072960 | 395773488.7 | 2072146461 | 836885711 | 675241880.1 | 558912830.8 | 99114091.87 | 245665329.3 | 48572376.51 | 104252148.3 |
|  |  | 1.207482779 | 2.89969934 | 0.041583317 |  | 45750336.31 | 99857361.62 | 107637089.4 | 28290934.27 | 30891649.91 | 47564118.99 | 34541356.29 | 10206536.79 | 122667811.8 | 126863032.8 | 39701591.36 | 68021141.4 |
|  |  | 1.13186869 | 4.413176141 | 0.041762947 |  | 16059830.1 | 5276408.201 | 9402357.242 | 12191771.49 | 3587090.873 | 16231289.57 | 26645722.03 | 12937333.65 | 92038185.89 | 100402483.3 | 51570331.2 | 18138000.19 |
|  |  | 2.075480718 | 0.4217591 | 0.04185217 |  | 392194061.4 | 334101348.3 | 408177288.5 | 501594280.2 | 199355511.2 | 431373632.9 | 221744532.1 | 186835703.1 | 100541190.7 | 112592209.4 | 93003294.79 | 132201493.6 |
|  |  | 2.251076353 | 1.93577042 | 0.042107368 |  | 417597049.7 | 187894979.9 | 243433359.3 | 401410736.8 | 320297194.4 | 271560714.5 | 229773962.2 | 110620364.4 | 425880198.7 | 529794568.2 | 588498482.5 | 260453052.4 |
|  |  | 1.165731016 | 0.632358488 | 0.04238845 |  | 112519408.9 | 66858987.06 | 43848923.65 | 115666523.1 | 101904644.5 | 173116748.9 | 178400056.9 | 169055592 | 133089462 | 97571136.08 | 93399888.95 | 69568154.39 |
|  |  | 1.15711032 | 2.683716616 | 0.04292621 |  | 41662679.27 | 109363353.9 | 95453212.39 | 26643439.38 | 27694848.84 | 52235785.46 | 38987942.15 | 11482356.83 | 128447083.6 | 112892697.5 | 47345749.27 | 61273621.07 |
|  |  | 1.107516382 | 0.521660633 | 0.043216238 |  | 126633295.8 | 45157173.91 | 55465560.91 | 64793784.15 | 61327542.38 | 90802270.67 | 64104594.74 | 130152453.5 | 37928125.27 | 50956524.68 | 47981182.86 | 43830556.65 |
|  |  | 1.261552072 | 0.538986322 | 0.043558448 |  | 81964509.41 | 121921939.8 | 321339316.6 | 65847309.6 | 102647484.1 | 119869886.1 | 98193714.84 | 187409907.9 | 95329426.16 | 65786625.86 | 66142156.18 | 46612057.1 |
|  |  | 1.063804663 | 1.765003364 | 0.043702947 |  | 208629582.6 | 146592749 | 192323851.2 | 159125638.6 | 53045573.4 | 80938976.11 | 62331621.26 | 64675227.53 | 73743415.01 | 116388855.4 | 164261144.3 | 106257281.2 |
|  |  | 1.104983162 | 1.767670219 | 0.043708771 |  | 122251487.5 | 95288568.69 | 117842231.4 | 102667748.5 | 56948580.27 | 98599634.65 | 56359581.73 | 37420488.51 | 88675416.26 | 94146202.87 | 149112795.4 | 108795770 |
|  |  | 2.02454629 | 1.921018911 | 0.043971958 |  | 465569999.2 | 232503467.5 | 189169671.4 | 362988941.7 | 145120790.6 | 193533626.6 | 167025263.1 | 245440035.6 | 200129240.5 | 320404416.1 | 495418025.6 | 426963496.8 |
|  |  | 1.812167507 | 3.448097029 | 0.043991319 |  | 110843356.6 | 68549341.76 | 93078485.37 | 305220714.4 | 41571150.01 | 67912304.68 | 35522105.58 | 82195257.48 | 70390137.96 | 156483110.5 | 235884037.4 | 320653178.8 |
|  |  | 1.081115558 | 1.572760803 | 0.044086201 |  | 150529400 | 14057203.14 | 95901909.34 | 25014986.95 | 44074294.76 | 100799171.4 | 65368154.28 | 98033669.81 | 103868341.6 | 143984331.1 | 101729866.5 | 135260754.1 |
|  |  | 1.093397943 | 0.492057648 | 0.044576834 |  | 60682201.6 | 181712483.8 | 102177818.8 | 104381649.9 | 94627736.7 | 65913678.88 | 63239683.28 | 116621597.6 | 8911353.108 | 59512251.48 | 44400525.96 | 54673619.59 |
|  |  | 1.336492191 | 0.629203764 | 0.044581646 |  | 258686465.6 | 50388901.04 | 4090336.015 | 29832394.96 | 71076315.2 | 72813878.06 | 69152365.05 | 48994302.85 | 27241162.94 | 35646819.85 | 38406328.76 | 63580267.9 |
|  |  | 8.281620606 | 25.01302012 | 0.044782064 |  | 4810572044 | 21490202.59 | 23806280.65 | 27509516.1 | 90786924.03 | 200828897.5 | 81633109.8 | 59722350.43 | 4987014392 | 26974491.46 | 3231274821 | 2584655679 |
|  |  | 1.577214534 | 1.759994165 | 0.045326389 |  | 303290253.1 | 176194339.6 | 234837767 | 187625067.1 | 107956481.7 | 196316358.4 | 134833034.8 | 50901793.29 | 215845603.5 | 166225585.4 | 270197231.5 | 210142216.3 |
|  |  | 1.383718057 | 0.528941014 | 0.045348365 |  | 29090613.21 | 57686332.26 | 78195851.47 | 45884331.91 | 186099832.4 | 144365360.3 | 109015551.2 | 124753015 | 65034841.01 | 128548248 | 75155038.47 | 29708249.03 |
|  |  | 1.231325264 | 1.729878601 | 0.045921213 |  | 72492943.47 | 62886892.49 | 87677006.04 | 52348773.68 | 53464929.21 | 84784616.16 | 50134090.94 | 117632574.5 | 170716018 | 144613734.5 | 111941627.2 | 102099514.7 |
|  |  | 4.072367376 | 0.596563101 | 0.046048635 |  | 427474341.3 | 792941157.2 | 1540042025 | 846216891.8 | 1255098218 | 1823662020 | 1340234895 | 804870056.2 | 760718240.3 | 751125118.7 | 850601791.1 | 753920067.9 |
|  |  | 1.094157427 | 0.333504668 | 0.046061555 |  | 48516569.52 | 19301418.38 | 43352391.04 | 23955802.66 | 43026915.91 | 60164447.69 | 41035901.93 | 112994453.3 | 30418486.81 | 20324575.31 | 15138336.68 | 19903245.21 |
|  |  | 1.483161525 | 2.568541011 | 0.046064502 |  | 75912215.34 | 92433180.13 | 211469974.8 | 149919922.7 | 80492936.02 | 79572591.57 | 26631831.85 | 33412185.84 | 191257112.6 | 55005808.27 | 136241082.8 | 182856390.5 |
|  |  | 2.12223898 | 0.320239311 | 0.046500645 |  | 27710692.33 | 53761384.92 | 82900270.12 | 200636621 | 272756116.3 | 114714615.7 | 164983337.1 | 377263976.8 | 45780418.44 | 142946720.7 | 37278558.44 | 71726568.89 |
|  |  | 1.02475901 | 0.489786018 | 0.047075431 |  | 140107360.3 | 100305390.8 | 161566799.3 | 67547772.11 | 52585460.04 | 90905434.08 | 90934544 | 84792993.15 | 11034918.42 | 32552091.24 | 75934003.43 | 36827711.09 |
|  |  | 1.03830842 | 0.377914872 | 0.047342147 |  | 15744674.3 | 118534919.4 | 45464265.17 | 9470988.646 | 91863825.8 | 94626422.76 | 27316246.17 | 66857097.94 | 24007916.42 | 45351055.84 | 30300075.67 | 6407897.865 |
|  |  | 1.703554343 | 1.765123497 | 0.047487761 |  | 234084331.6 | 442807601.8 | 245628446.4 | 239196802.5 | 130447171.9 | 190870170.7 | 204010858 | 97500331.45 | 298548288.9 | 379362152.5 | 222552993 | 198905842 |
|  |  | 3.296720581 | 0.096756656 | 0.047496045 |  | 45477409.62 | 20777009.58 | 65426415.18 | 123328313.7 | 904781257.9 | 278069280.6 | 122933749.1 | 715816993.1 | 6561463.981 | 77792711.39 | 72460551.83 | 38788652.05 |
|  |  | 1.1544393 | 1.584814577 | 0.047621727 |  | 145730400 | 103735780 | 148308247.5 | 173243832.8 | 38892059.49 | 87076537.37 | 128768250.8 | 122212124 | 163928087.8 | 129860356 | 162259182.1 | 141346599.1 |
|  |  | 1.84243501 | 0.627220357 | 0.047825948 |  | 73768667.09 | 204761186.5 | 394153743.1 | 73871881.55 | 294382772.8 | 312701882.6 | 336085184.2 | 274723370.4 | 203164194 | 310170324.5 | 118242465.1 | 132310430.8 |
|  |  | 1.5580493 | 0.479965395 | 0.048425167 |  | 42565937.65 | 170677466.3 | 177012726.4 | 28782514.75 | 147981325.1 | 174454365.2 | 181594693.4 | 195758135.3 | 34033639.7 | 140972229.2 | 12238521.62 | 148629882.6 |
|  |  | 1.319484272 | 2.240714418 | 0.04898365 |  | 84933179.9 | 109049615.8 | 72988426.9 | 121455245.6 | 49851354.41 | 79329289.54 | 55442198.71 | 38135088.58 | 194337094.7 | 65377504.2 | 127825689.4 | 111596619.8 |
|  |  | 1.673504088 | 0.340881959 | 0.049025419 |  | 11635087.62 | 25196993.58 | 20027451.67 | 34729976.37 | 179067063.4 | 46030921.81 | 198604779.5 | 146268775 | 32916489.62 | 100945007.7 | 28804132.39 | 31627385.6 |
|  |  | 1.031622266 | 1.618535914 | 0.049578131 |  | 61853953.92 | 55986682.99 | 58447184.4 | 50939740.01 | 42283934.87 | 53759756.9 | 44445350.89 | 97584467.87 | 74211205.2 | 99696998.75 | 108832893.7 | 102589429.4 |
|  |  | 1.114789724 | 0.470492867 | 0.049903916 |  | 81328587.02 | 45540280.71 | 52579080.75 | 54059640.16 | 51454291.22 | 121718904 | 92164495.82 | 121654548.6 | 61728702.33 | 54724324.03 | 7643384.713 | 57980677.36 |
|  |  | 1.001886492 | 2.202052833 | 0.049944318 |  | 220299199.1 | 20071135.83 | 139152667 | 160666557.9 | 20841149.69 | 19142316.02 | 47620473.69 | 50519142.38 | 109854546.6 | 45264133.25 | 90397940.2 | 58637703.47 |
