# Supplementary material for: To explore the mechanism of acupoint application in the treatment of primary dysmenorrhea by 16S rDNA sequencing and metabolomics
Source: Front Endocrinol (Lausanne). 2024 May 30;15:1397402. doi: 10.3389/fendo.2024.1397402 (PMC11169635; doi:10.3389/fendo.2024.1397402)
Supplement: Supplementary file 4 [file Table_4.docx]

Table S4: Screening of differential metabolites in feces of three groups of rats

| m/z | rt(s) | Name | adduct | SuperClass | Model | Treatment | Fold change | p-value | VIP |
| --- | --- | --- | --- | --- | --- | --- | --- | --- | --- |
| 317.06690 | 22.646100 | (2s,3s)-2-(3,4-dihydroxyphenyl)-3,5,7-trihydroxy-6-methyl-2,3-dihydrochromen-4-one | [M-H]- | Phenylpropanoids and polyketides | ↓ | ↑ | 0.575545931 | 0.016158661 | 1.097190793 |
| 373.27392 | 107.824000 | .beta.-muricholic acid | [M+H-2H2O]+ | Lipids and lipid-like molecules | ↑ | ↓ | 1.667206575 | 0.03113535 | 3.395958844 |
| 505.26300 | 21.259350 | 11.alpha.-hydroxyprogesterone .beta.-d-glucuronide | [M-H]- | Lipids and lipid-like molecules | ↓ | ↑ | 0.613645826 | 0.026590333 | 1.57810684 |
| 329.10324 | 343.443500 | 3,2'-dihydroxy-4,4',6'-trimethoxychalcone | [M-H]- | Phenylpropanoids and polyketides | ↓ | ↑ | 0.733404669 | 0.002024485 | 1.951628979 |
| 269.11346 | 220.189500 | 3,4-dimethoxychalcone | [M+H]+ | Phenylpropanoids and polyketides | ↑ | ↓ | 19.8438166 | 0.001780301 | 4.623807661 |
| 779.54657 | 107.834500 | 3.alpha.-hydroxy-7-oxo-5.beta.-cholanic acid | [2M-H]- | Lipids and lipid-like molecules | ↑ | ↓ | 2.447559616 | 0.005477229 | 3.750954123 |
| 107.05012 | 207.735500 | 3-hydroxyphenylacetic acid | [M-H-CO2]- | Benzenoids | ↓ | ↑ | 0.685245551 | 0.029337363 | 1.9941822 |
| 337.03882 | 27.818350 | 3-phosphonoalanine | [2M-H]- | Organic acids and derivatives | ↓ | ↑ | 0.114704279 | 0.028732237 | 5.964956439 |
| 329.10322 | 51.291200 | 4,2'-dihydroxy-3,4',6'-trimethoxychalcone | [M-H]- | Phenylpropanoids and polyketides | ↑ | ↓ | 1.375736299 | 0.009270405 | 3.285975739 |
| 315.19140 | 357.383000 | 6-oxirane boldenone | [M+H]+ | Lipids and lipid-like molecules | ↑ | ↓ | 7.100601145 | 1.16887E-06 | 2.620042928 |
| 256.09298 | 159.284000 | 7,8-dihydroneopterin | [M+H]+ | Organoheterocyclic compounds | ↓ | ↑ | 0.488142507 | 0.001590268 | 2.494686091 |
| 193.09766 | 100.585000 | Apiole | [M+H-CH2O]+ |  | ↓ | ↑ | 0.526204203 | 0.006880809 | 1.394150065 |
| 303.21784 | 217.019000 | Arachidonic acid (peroxide free) | [M-H]- | Lipids and lipid-like molecules | ↓ | ↑ | 0.502757898 | 0.012909159 | 1.689150736 |
| 463.35332 | 278.705000 | Arachidonoylserotonin | [M+H]+ | Organoheterocyclic compounds | ↑ | ↓ | 3.945948435 | 0.000649928 | 1.590181072 |
| 435.20550 | 27.404750 | Artocaprin | [M-H]- | Phenylpropanoids and polyketides | ↓ | ↑ | 0.383885284 | 0.004140065 | 1.509767386 |
| 367.33231 | 227.102000 | Batyl alcohol | [M+Na]+ | Lipids and lipid-like molecules | ↓ | ↑ | 0.726835033 | 0.044150209 | 1.716592052 |
| 583.25840 | 77.050300 | Bilirubin | [M-H]- | Organoheterocyclic compounds | ↑ | ↓ | 2.449174522 | 0.011446394 | 1.299838933 |
| 245.11328 | 390.478500 | Biotin | [M+H]+ |  | ↑ | ↓ | 4.079392829 | 5.72717E-05 | 3.670576827 |
| 365.31636 | 229.060500 | Cholesta-4,6-dien-3-one | [M+H-H2O]+ | Lipids and lipid-like molecules | ↓ | ↑ | 0.760641298 | 0.045312998 | 2.785487538 |
| 347.30586 | 219.002500 | Cis-4,10,13,16-docosatetraenoic acid methyl ester | [M+H]+ | Lipids and lipid-like molecules | ↓ | ↑ | 0.628566028 | 0.043294359 | 1.337780825 |
| 531.29981 | 88.860350 | Cochlioquinone a | [M-H]- |  | ↑ | ↓ | 3.364466965 | 0.018500767 | 4.143170185 |
| 241.11851 | 74.989200 | Cyanazine | [M+H]+ | Organoheterocyclic compounds | ↓ | ↑ | 0.750552527 | 0.038131478 | 1.089025116 |
| 173.09239 | 246.764000 | Deoxypeganine | [M+H]+ | Organoheterocyclic compounds | ↑ | ↓ | 10.02652807 | 8.36381E-05 | 3.399148131 |
| 128.08196 | 155.381000 | Dl-5-hydroxylysine | [M+H-H5ON]+ | Organic acids and derivatives | ↓ | ↑ | 0.601806894 | 0.018209597 | 1.16877105 |
| 297.12712 | 58.075100 | Enterolactone | [M-H]- | Lignans, neolignans and related compounds | ↑ | ↓ | 3.4515162 | 0.012235988 | 2.229726849 |
| 218.13866 | 387.004000 | Ethoxyquin | [M+H]+ | Organoheterocyclic compounds | ↓ | ↑ | 0.409760857 | 5.28412E-06 | 4.006834587 |
| 226.10882 | 705.252500 | Ethyl 3-indoleacetate | [M+Na]+ | Organoheterocyclic compounds | ↑ | ↓ | 1.893137719 | 0.027018957 | 1.276840393 |
| 423.36237 | 30.744650 | Ginsenoside f1 | [M+H-C6H16O8]+ | Lipids and lipid-like molecules | ↓ | ↑ | 0.565322528 | 0.037958118 | 2.06797774 |
| 276.08560 | 175.656000 | Gln-met | [M-H]- | Organic acids and derivatives | ↓ | ↑ | 0.134094508 | 0.019765479 | 2.30065207 |
| 257.16087 | 371.826000 | Huperzine b | [M+H]+ |  | ↓ | ↑ | 0.669888418 | 0.002648369 | 4.049697377 |
| 135.03125 | 185.843500 | Hypoxanthine | [M-H]- | Organoheterocyclic compounds | ↓ | ↑ | 0.44597491 | 0.025623153 | 5.766820161 |
| 202.02949 | 214.521500 | Indole-3-pyruvic acid | [M-H]- | Organoheterocyclic compounds | ↓ | ↑ | 0.663210001 | 0.044638771 | 1.111157068 |
| 347.02623 | 28.875550 | Inosine 5'-monophosphate | [M-H]- | Nucleosides, nucleotides, and analogues | ↓ | ↑ | 0.393984692 | 0.005216999 | 1.113796054 |
| 202.14307 | 366.666000 | Lobelanidine | [M+H-C8H10O2]+ | Organic nitrogen compounds | ↓ | ↑ | 0.722267216 | 0.034508423 | 1.430828873 |
| 218.13875 | 364.653000 | L-propionylcarnitine | [M+H]+ | Lipids and lipid-like molecules | ↓ | ↑ | 0.491483502 | 0.000368488 | 1.707818908 |
| 241.07311 | 54.877000 | Lumichrome | [M-H]- | Organoheterocyclic compounds | ↓ | ↑ | 0.560281534 | 0.017175254 | 1.8338798 |
| 215.10280 | 369.780000 | Metribuzin | [M+H]+ | Organosulfur compounds | ↑ | ↓ | 2.987030904 | 0.000194574 | 2.1563992 |
| 240.05693 | 94.234100 | N-[tris(hydroxymethyl)methyl]-3-amino-2-hydroxypropanesulfonic acid | [M-H-H2O]- | Organic acids and derivatives | ↑ | ↓ | 2.352320264 | 0.043827003 | 2.137767654 |
| 301.20218 | 320.451500 | Neoabietic acid | [M-H]- | Lipids and lipid-like molecules | ↓ | ↑ | 0.53180558 | 0.006184754 | 2.689336077 |
| 122.02478 | 239.380000 | Nicotinate | [M-H]- | Organoheterocyclic compounds | ↓ | ↑ | 0.581866688 | 0.040135299 | 2.494440817 |
| 146.11756 | 271.428000 | N-methyl-l-isoleucine | [M+H]+ | Organic acids and derivatives | ↓ | ↑ | 0.296058953 | 0.000298967 | 6.452406413 |
| 383.32692 | 229.119000 | Pinanethromboxane a2 | [M+Li]+ | Lipids and lipid-like molecules | ↓ | ↑ | 0.759538166 | 0.032487128 | 2.343115559 |
| 515.30490 | 46.207900 | Probucol | [M-H]- | Benzenoids | ↑ | ↓ | 2.98981298 | 0.000531648 | 4.334767879 |
| 218.11347 | 399.428500 | Pymetrozin | [M+H]+ | Organoheterocyclic compounds | ↑ | ↓ | 2.185496916 | 0.044304216 | 1.327878878 |
| 247.06462 | 28.072200 | Pyridoxamine 5-phosphate | [M-H]- | Organoheterocyclic compounds | ↓ | ↑ | 0.330368478 | 0.006258197 | 1.533819319 |
| 250.11106 | 365.980000 | Ser-Gly-Ser | [M+H]+ | Organic acids and derivatives | ↓ | ↑ | 0.558118016 | 0.008760216 | 1.913010257 |
| 302.19622 | 391.104000 | Trachelanthine | [M+H]+ |  | ↓ | ↑ | 0.513773934 | 0.0205726 | 3.696690285 |
| 367.10514 | 322.600500 | Trans-3'-hydroxycotinine o-.beta.-d-glucuronide | [M-H]- | Organic oxygen compounds | ↓ | ↑ | 0.201435425 | 0.027879367 | 1.538398489 |
| 227.12898 | 215.597500 | Trans-traumatic acid | [M-H]- | Lipids and lipid-like molecules | ↓ | ↑ | 0.491109998 | 0.008090899 | 2.092921151 |
| 151.02618 | 237.376500 | Xanthine | [M-H]- | Organoheterocyclic compounds | ↓ | ↑ | 0.400165862 | 0.000243027 | 4.972452727 |
| 264.10914 | 183.750500 | Zaleplon | [M+H-C2H2O]+ | Organoheterocyclic compounds | ↓ | ↑ | 0.527479817 | 0.004276449 | 3.449852833 |
| 331.12218 | 32.680700 | Zanamivir | [M-H]- | Organic acids and derivatives | ↓ | ↑ | 0.564889545 | 0.02140546 | 1.781361523 |
