# Supplementary material for: To explore the mechanism of acupoint application in the treatment of primary dysmenorrhea by 16S rDNA sequencing and metabolomics
Source: Front Endocrinol (Lausanne). 2024 May 30;15:1397402. doi: 10.3389/fendo.2024.1397402 (PMC11169635; doi:10.3389/fendo.2024.1397402)
Supplement: Supplementary file 5 [file Table_5.docx]

Table S5: Screening of differential flora and differential metabolites ( Model VS Control )

| ID | Aerococcus | Lachnospiraceae_NK4A136_group | Catabacter | gut_metagenome | Butyricicoccus | Kribbella | Lactobacillus | Dubosiella | Candidatus_Arthromitus | Lachnoanaerobaculum | Papillibacter | Peptococcus | uncultured_Acidimicrobidae_bacterium | Haemophilus | Enterococcus | Others |
| --- | --- | --- | --- | --- | --- | --- | --- | --- | --- | --- | --- | --- | --- | --- | --- | --- |
| 11_dehydrothromboxane b2 | -0.706599495 | 0.428571429 | 0.36147202 | 0.472805429 | 0.742528282 | -0.659828879 | 0.642857143 | -0.30952381 | 0.306892885 | 0.600099198 | -0.666666667 | 0.598813131 | -0.701068184 | 0.600099198 | -0.574860606 | -0.214285714 |
| 1,24_dihydroxyvitamin d3 | -0.898219696 | 0.904761905 | 0.614502433 | 0.666776887 | 0.970077272 | -0.866025404 | 0.69047619 | -0.857142857 | 0.527855762 | 0.872871561 | -0.738095238 | 0.718575757 | -0.783546794 | 0.872871561 | -0.670670707 | 0 |
| 2,3_dimercaptosuccinic acid | -0.634741919 | 0.523809524 | 0.530158962 | 0.509175077 | 0.706599495 | -0.783546794 | 0.833333333 | -0.357142857 | 0.503304332 | 0.463713017 | -0.714285714 | 0.670670707 | -0.824786099 | 0.463713017 | -0.419169192 | -0.238095238 |
| 2_amino_1_phenylethanol | -0.802409595 | 0.714285714 | 0.80728751 | 0.666776887 | 0.682646969 | -0.536110964 | 0.69047619 | -0.619047619 | 0.650612916 | 0.545544726 | -0.595238095 | 0.50300303 | -0.453632354 | 0.545544726 | -0.622765656 | 0.285714286 |
| 2_amino_9h_pyrido[2,3_b]indole | -0.742528282 | 0.642857143 | 0.49401176 | 0.460682213 | 0.790433333 | -0.824786099 | 0.738095238 | -0.523809524 | 0.380547178 | 0.600099198 | -0.69047619 | 0.610789394 | -0.783546794 | 0.600099198 | -0.45509798 | -0.071428571 |
| 2_aminophenol | 0.55090808 | -0.523809524 | -0.722944039 | -0.703146535 | -0.479050505 | 0.412393049 | -0.666666667 | 0.380952381 | -0.724267209 | -0.545544726 | 0.261904762 | -0.50300303 | 0.577350269 | -0.545544726 | 0.55090808 | 0.023809524 |
| 2_arachidonoylglycerol | -0.814385858 | 0.80952381 | 0.626551501 | 0.569791158 | 0.874267171 | -0.866025404 | 0.761904762 | -0.714285714 | 0.527855762 | 0.681930907 | -0.738095238 | 0.646718181 | -0.783546794 | 0.681930907 | -0.514979293 | 0 |
| 2'_deoxycytidine 5'_monophosphate | -0.682646969 | 0.714285714 | 0.686796837 | 0.691023319 | 0.754504545 | -0.783546794 | 0.833333333 | -0.571428571 | 0.699715778 | 0.654653671 | -0.595238095 | 0.718575757 | -0.866025404 | 0.654653671 | -0.526955555 | -0.19047619 |
| 2'_o_methyladenosine | -0.862290909 | 0.714285714 | 0.819336578 | 0.957734074 | 0.77845707 | -0.536110964 | 0.761904762 | -0.619047619 | 0.77337007 | 0.791039852 | -0.642857143 | 0.838338383 | -0.618589574 | 0.791039852 | -0.946124747 | 0 |
| 2_pyrrolidinone, 1_methyl_ | -0.814385858 | 0.666666667 | 0.602453366 | 0.533421509 | 0.766480808 | -0.577350269 | 0.571428571 | -0.619047619 | 0.45420147 | 0.545544726 | -0.738095238 | 0.479050505 | -0.412393049 | 0.545544726 | -0.598813131 | 0.214285714 |
| 3h_imidazo(4,5_f)quinoline, 2_amino_3_methyl_ | 0.850314646 | -0.69047619 | -0.626551501 | -0.739516184 | -0.898219696 | 0.783546794 | -0.80952381 | 0.571428571 | -0.601510055 | -0.763762616 | 0.785714286 | -0.802409595 | 0.824786099 | -0.763762616 | 0.742528282 | 0.19047619 |
| 3_hydroxyphenylacetic acid | -0.598813131 | 0.833333333 | 0.566306164 | 0.521298293 | 0.754504545 | -0.866025404 | 0.69047619 | -0.761904762 | 0.564682909 | 0.627376434 | -0.571428571 | 0.634741919 | -0.824786099 | 0.627376434 | -0.323359091 | -0.119047619 |
| 4,6_dinitro_o_cresol | -0.622765656 | 0.857142857 | 0.481962693 | 0.521298293 | 0.77845707 | -0.866025404 | 0.619047619 | -0.833333333 | 0.441925755 | 0.681930907 | -0.619047619 | 0.73055202 | -0.783546794 | 0.681930907 | -0.395216666 | -0.095238095 |
| 5,7,3',4',5'_pentahydroxyflavone | -0.622765656 | 0.666666667 | 0.614502433 | 0.557667942 | 0.658694444 | -0.783546794 | 0.80952381 | -0.523809524 | 0.540131478 | 0.545544726 | -0.547619048 | 0.682646969 | -0.824786099 | 0.545544726 | -0.407192929 | -0.095238095 |
| 5(s),14(r)_lipoxin b4 | -0.77845707 | 0.80952381 | 0.578355231 | 0.715269751 | 0.934148484 | -0.824786099 | 0.69047619 | -0.738095238 | 0.650612916 | 0.872871561 | -0.666666667 | 0.718575757 | -0.866025404 | 0.872871561 | -0.646718181 | -0.261904762 |
| 7,8_dihydroneopterin | -0.574860606 | 0.761904762 | 0.542208029 | 0.460682213 | 0.73055202 | -0.866025404 | 0.714285714 | -0.666666667 | 0.540131478 | 0.545544726 | -0.595238095 | 0.586836868 | -0.824786099 | 0.545544726 | -0.263477778 | -0.142857143 |
| Ala_Ala | -0.670670707 | 0.880952381 | 0.566306164 | 0.424312564 | 0.658694444 | -0.618589574 | 0.404761905 | -0.904761905 | 0.380547178 | 0.627376434 | -0.404761905 | 0.371264141 | -0.412393049 | 0.627376434 | -0.371264141 | 0.380952381 |
| Apiole | -0.467074242 | 0.642857143 | 0.578355231 | 0.509175077 | 0.646718181 | -0.824786099 | 0.80952381 | -0.5 | 0.662888632 | 0.409158544 | -0.619047619 | 0.646718181 | -0.866025404 | 0.409158544 | -0.22754899 | -0.30952381 |
| Arachidonic acid (peroxide free) | -0.670670707 | 0.857142857 | 0.602453366 | 0.521298293 | 0.754504545 | -0.866025404 | 0.714285714 | -0.785714286 | 0.503304332 | 0.627376434 | -0.595238095 | 0.658694444 | -0.783546794 | 0.627376434 | -0.371264141 | 0.023809524 |
| Azelaic acid | -0.479050505 | 0.69047619 | 0.542208029 | 0.327326835 | 0.562884343 | -0.783546794 | 0.69047619 | -0.595238095 | 0.441925755 | 0.300049599 | -0.571428571 | 0.50300303 | -0.659828879 | 0.300049599 | -0.107786364 | 0.047619048 |
| Batyl alcohol | -0.838338383 | 0.761904762 | 0.662698702 | 0.618284022 | 0.838338383 | -0.824786099 | 0.80952381 | -0.642857143 | 0.527855762 | 0.681930907 | -0.714285714 | 0.694623232 | -0.783546794 | 0.681930907 | -0.586836868 | 0.023809524 |
| .beta._muricholic acid | 0.646718181 | -0.666666667 | -0.831385645 | -0.739516184 | -0.598813131 | 0.494871659 | -0.714285714 | 0.547619048 | -0.822472932 | -0.518267489 | 0.476190476 | -0.526955555 | 0.536110964 | -0.518267489 | 0.562884343 | -0.071428571 |
| Bilirubin | 0.886243434 | -0.666666667 | -0.80728751 | -0.788009048 | -0.742528282 | 0.494871659 | -0.714285714 | 0.571428571 | -0.662888632 | -0.627376434 | 0.69047619 | -0.634741919 | 0.453632354 | -0.627376434 | 0.826362121 | -0.214285714 |
| Biotin | 0.77845707 | -0.904761905 | -0.915729116 | -0.763762616 | -0.694623232 | 0.659828879 | -0.785714286 | 0.833333333 | -0.74881864 | -0.545544726 | 0.666666667 | -0.718575757 | 0.536110964 | -0.545544726 | 0.598813131 | -0.30952381 |
| Camptothecin | -0.706599495 | 0.785714286 | 0.710894972 | 0.7516394 | 0.77845707 | -0.783546794 | 0.80952381 | -0.666666667 | 0.724267209 | 0.73648538 | -0.571428571 | 0.766480808 | -0.866025404 | 0.73648538 | -0.586836868 | -0.166666667 |
| Chrysin | -0.802409595 | 0.714285714 | 0.795238443 | 0.739516184 | 0.694623232 | -0.659828879 | 0.833333333 | -0.571428571 | 0.638337201 | 0.654653671 | -0.547619048 | 0.706599495 | -0.701068184 | 0.654653671 | -0.670670707 | 0.119047619 |
| Cyanazine | -0.862290909 | 0.952380952 | 0.67474777 | 0.691023319 | 0.922172222 | -0.866025404 | 0.714285714 | -0.904761905 | 0.576958624 | 0.845594325 | -0.69047619 | 0.742528282 | -0.783546794 | 0.845594325 | -0.634741919 | 0.047619048 |
| Cycloate | -0.718575757 | 0.761904762 | 0.915729116 | 0.727392967 | 0.50300303 | -0.494871659 | 0.761904762 | -0.666666667 | 0.650612916 | 0.43643578 | -0.5 | 0.670670707 | -0.412393049 | 0.43643578 | -0.598813131 | 0.452380952 |
| Dapsone | -0.718575757 | 0.80952381 | 0.722944039 | 0.59403759 | 0.77845707 | -0.866025404 | 0.857142857 | -0.69047619 | 0.638337201 | 0.518267489 | -0.761904762 | 0.706599495 | -0.783546794 | 0.518267489 | -0.419169192 | 0 |
| Deoxypeganine | 0.682646969 | -0.833333333 | -0.915729116 | -0.81225548 | -0.634741919 | 0.536110964 | -0.714285714 | 0.761904762 | -0.871575794 | -0.545544726 | 0.547619048 | -0.634741919 | 0.494871659 | -0.545544726 | 0.598813131 | -0.19047619 |
| Dl_a_hydroxybutyric acid | -0.73055202 | 0.928571429 | 0.843434712 | 0.715269751 | 0.754504545 | -0.783546794 | 0.80952381 | -0.857142857 | 0.74881864 | 0.545544726 | -0.738095238 | 0.754504545 | -0.659828879 | 0.545544726 | -0.50300303 | 0.142857143 |
| DL_isoleucine | -0.850314646 | 0.928571429 | 0.734993106 | 0.739516184 | 0.790433333 | -0.577350269 | 0.523809524 | -0.952380952 | 0.576958624 | 0.763762616 | -0.619047619 | 0.646718181 | -0.412393049 | 0.763762616 | -0.742528282 | 0.333333333 |
| Dodecanedioic acid | -0.514979293 | 0.714285714 | 0.578355231 | 0.460682213 | 0.682646969 | -0.866025404 | 0.785714286 | -0.595238095 | 0.601510055 | 0.409158544 | -0.666666667 | 0.622765656 | -0.824786099 | 0.409158544 | -0.203596465 | -0.19047619 |
| Emetine | -0.45509798 | 0.523809524 | 0.409668289 | 0.400066132 | 0.610789394 | -0.783546794 | 0.714285714 | -0.380952381 | 0.45420147 | 0.463713017 | -0.5 | 0.574860606 | -0.866025404 | 0.463713017 | -0.22754899 | -0.333333333 |
| Epinephrine | -0.77845707 | 0.857142857 | 0.686796837 | 0.59403759 | 0.826362121 | -0.866025404 | 0.785714286 | -0.761904762 | 0.576958624 | 0.654653671 | -0.69047619 | 0.670670707 | -0.783546794 | 0.654653671 | -0.479050505 | 0.047619048 |
| Ethyl 3_indoleacetate | 0.826362121 | -0.80952381 | -0.831385645 | -0.691023319 | -0.682646969 | 0.536110964 | -0.642857143 | 0.738095238 | -0.638337201 | -0.654653671 | 0.476190476 | -0.491026767 | 0.453632354 | -0.654653671 | 0.646718181 | -0.380952381 |
| Fenfluramine | -0.514979293 | 0.595238095 | 0.31327575 | 0.278833971 | 0.73055202 | -0.866025404 | 0.619047619 | -0.5 | 0.343720031 | 0.490990253 | -0.619047619 | 0.479050505 | -0.824786099 | 0.490990253 | -0.167667677 | -0.285714286 |
| Flutamide | -0.922172222 | 0.833333333 | 0.650649635 | 0.7516394 | 0.95810101 | -0.824786099 | 0.761904762 | -0.761904762 | 0.564682909 | 0.845594325 | -0.80952381 | 0.826362121 | -0.783546794 | 0.845594325 | -0.77845707 | -0.047619048 |
| Geranylgeranyl pyrophosphate | -0.431145454 | 0.547619048 | 0.771140308 | 0.848625129 | 0.538931818 | -0.536110964 | 0.833333333 | -0.404761905 | 0.969781517 | 0.381881308 | -0.619047619 | 0.826362121 | -0.701068184 | 0.381881308 | -0.526955555 | -0.380952381 |
| Gomisin c | -0.754504545 | 0.642857143 | 0.638600568 | 0.497051861 | 0.694623232 | -0.577350269 | 0.619047619 | -0.571428571 | 0.478752901 | 0.43643578 | -0.714285714 | 0.45509798 | -0.412393049 | 0.43643578 | -0.50300303 | 0.238095238 |
| Heliotrine | -0.55090808 | 0.642857143 | 0.578355231 | 0.557667942 | 0.658694444 | -0.783546794 | 0.785714286 | -0.5 | 0.601510055 | 0.545544726 | -0.523809524 | 0.658694444 | -0.866025404 | 0.545544726 | -0.359287879 | -0.238095238 |
| Hexadecanedioic acid | 0.766480808 | -0.880952381 | -0.747042174 | -0.569791158 | -0.646718181 | 0.577350269 | -0.547619048 | 0.857142857 | -0.503304332 | -0.627376434 | 0.428571429 | -0.45509798 | 0.412393049 | -0.627376434 | 0.514979293 | -0.476190476 |
| Homoplantaginin | -0.574860606 | 0.714285714 | 0.602453366 | 0.618284022 | 0.682646969 | -0.783546794 | 0.761904762 | -0.595238095 | 0.626061486 | 0.627376434 | -0.5 | 0.706599495 | -0.866025404 | 0.627376434 | -0.419169192 | -0.214285714 |
| Huperzine b | -0.670670707 | 0.738095238 | 0.614502433 | 0.509175077 | 0.694623232 | -0.824786099 | 0.785714286 | -0.619047619 | 0.478752901 | 0.545544726 | -0.595238095 | 0.658694444 | -0.783546794 | 0.545544726 | -0.383240404 | 0.023809524 |
| Hydroxyphenyllactic acid | -0.73055202 | 0.80952381 | 0.626551501 | 0.7516394 | 0.802409595 | -0.783546794 | 0.738095238 | -0.738095238 | 0.601510055 | 0.791039852 | -0.619047619 | 0.862290909 | -0.824786099 | 0.791039852 | -0.658694444 | -0.142857143 |
| Hypoxanthine | -0.694623232 | 0.69047619 | 0.759091241 | 0.691023319 | 0.706599495 | -0.783546794 | 0.928571429 | -0.523809524 | 0.699715778 | 0.518267489 | -0.69047619 | 0.77845707 | -0.824786099 | 0.518267489 | -0.514979293 | -0.095238095 |
| Indole_3_pyruvic acid | -0.862290909 | 0.952380952 | 0.67474777 | 0.691023319 | 0.922172222 | -0.866025404 | 0.714285714 | -0.904761905 | 0.576958624 | 0.845594325 | -0.69047619 | 0.742528282 | -0.783546794 | 0.845594325 | -0.634741919 | 0.047619048 |
| Inosine 5'_monophosphate | -0.73055202 | 0.80952381 | 0.734993106 | 0.884994777 | 0.850314646 | -0.783546794 | 0.833333333 | -0.714285714 | 0.822472932 | 0.763762616 | -0.738095238 | 0.934148484 | -0.866025404 | 0.763762616 | -0.718575757 | -0.285714286 |
| L_abrine | -0.742528282 | 0.642857143 | 0.903680049 | 0.788009048 | 0.562884343 | -0.453632354 | 0.785714286 | -0.5 | 0.761094355 | 0.518267489 | -0.476190476 | 0.598813131 | -0.494871659 | 0.518267489 | -0.682646969 | 0.238095238 |
| Levorphanol | -0.55090808 | 0.642857143 | 0.578355231 | 0.557667942 | 0.658694444 | -0.783546794 | 0.785714286 | -0.5 | 0.601510055 | 0.545544726 | -0.523809524 | 0.658694444 | -0.866025404 | 0.545544726 | -0.359287879 | -0.238095238 |
| Lithocholic acid | 0.670670707 | -0.619047619 | -0.626551501 | -0.739516184 | -0.826362121 | 0.701068184 | -0.761904762 | 0.5 | -0.785645786 | -0.627376434 | 0.761904762 | -0.706599495 | 0.783546794 | -0.627376434 | 0.610789394 | 0.380952381 |
| Lobelanidine | -0.526955555 | 0.69047619 | 0.542208029 | 0.509175077 | 0.694623232 | -0.824786099 | 0.738095238 | -0.571428571 | 0.601510055 | 0.545544726 | -0.547619048 | 0.610789394 | -0.866025404 | 0.545544726 | -0.287430303 | -0.261904762 |
| Lumichrome | -0.742528282 | 0.833333333 | 0.759091241 | 0.59403759 | 0.754504545 | -0.783546794 | 0.785714286 | -0.738095238 | 0.638337201 | 0.518267489 | -0.714285714 | 0.622765656 | -0.659828879 | 0.518267489 | -0.443121717 | 0.142857143 |
| Medroxyprogesterone 17_acetate | -0.610789394 | 0.69047619 | 0.650649635 | 0.509175077 | 0.646718181 | -0.824786099 | 0.857142857 | -0.547619048 | 0.540131478 | 0.409158544 | -0.666666667 | 0.694623232 | -0.783546794 | 0.409158544 | -0.323359091 | -0.023809524 |
| Metribuzin | 0.754504545 | -0.761904762 | -0.891630982 | -0.7516394 | -0.658694444 | 0.701068184 | -0.928571429 | 0.619047619 | -0.724267209 | -0.463713017 | 0.714285714 | -0.802409595 | 0.659828879 | -0.463713017 | 0.586836868 | -0.166666667 |
| N_acetyl_l_phenylalanine | -0.694623232 | 0.714285714 | 0.530158962 | 0.763762616 | 0.874267171 | -0.783546794 | 0.714285714 | -0.642857143 | 0.650612916 | 0.791039852 | -0.738095238 | 0.874267171 | -0.866025404 | 0.791039852 | -0.682646969 | -0.404761905 |
| Neoabietic acid | -0.586836868 | 0.738095238 | 0.614502433 | 0.460682213 | 0.682646969 | -0.866025404 | 0.80952381 | -0.619047619 | 0.540131478 | 0.409158544 | -0.69047619 | 0.646718181 | -0.783546794 | 0.409158544 | -0.251501515 | -0.047619048 |
| Neohesperidose | -0.682646969 | 0.833333333 | 0.67474777 | 0.703146535 | 0.814385858 | -0.824786099 | 0.761904762 | -0.738095238 | 0.724267209 | 0.73648538 | -0.595238095 | 0.718575757 | -0.866025404 | 0.73648538 | -0.514979293 | -0.19047619 |
| Nicotinate | -0.718575757 | 0.666666667 | 0.626551501 | 0.666776887 | 0.802409595 | -0.783546794 | 0.80952381 | -0.523809524 | 0.650612916 | 0.681930907 | -0.642857143 | 0.694623232 | -0.866025404 | 0.681930907 | -0.562884343 | -0.238095238 |
| N_methyltyramine | -0.694623232 | 0.666666667 | 0.49401176 | 0.484928645 | 0.826362121 | -0.701068184 | 0.571428571 | -0.619047619 | 0.515580047 | 0.545544726 | -0.785714286 | 0.491026767 | -0.577350269 | 0.545544726 | -0.45509798 | -0.095238095 |
| N,n'_diacetylchitobiose | -0.754504545 | 0.761904762 | 0.891630982 | 0.7516394 | 0.658694444 | -0.701068184 | 0.928571429 | -0.619047619 | 0.724267209 | 0.463713017 | -0.714285714 | 0.802409595 | -0.659828879 | 0.463713017 | -0.586836868 | 0.166666667 |
| Phloretin | -0.826362121 | 0.785714286 | 0.819336578 | 0.800132264 | 0.718575757 | -0.659828879 | 0.80952381 | -0.666666667 | 0.662888632 | 0.73648538 | -0.523809524 | 0.754504545 | -0.701068184 | 0.73648538 | -0.73055202 | 0.142857143 |
| Pravastatin | -0.658694444 | 0.595238095 | 0.722944039 | 0.848625129 | 0.766480808 | -0.659828879 | 0.857142857 | -0.452380952 | 0.871575794 | 0.572821962 | -0.785714286 | 0.838338383 | -0.783546794 | 0.572821962 | -0.682646969 | -0.380952381 |
| Probucol | 0.718575757 | -0.642857143 | -0.602453366 | -0.509175077 | -0.658694444 | 0.453632354 | -0.452380952 | 0.595238095 | -0.503304332 | -0.600099198 | 0.428571429 | -0.27545404 | 0.371153744 | -0.600099198 | 0.526955555 | -0.238095238 |
| Pyridoxamine 5_phosphate | -0.802409595 | 0.738095238 | 0.614502433 | 0.800132264 | 0.922172222 | -0.783546794 | 0.761904762 | -0.642857143 | 0.687440063 | 0.845594325 | -0.738095238 | 0.826362121 | -0.866025404 | 0.845594325 | -0.754504545 | -0.30952381 |
| Serotonin | -0.898219696 | 0.880952381 | 0.614502433 | 0.703146535 | 0.994029797 | -0.866025404 | 0.714285714 | -0.833333333 | 0.564682909 | 0.845594325 | -0.833333333 | 0.77845707 | -0.783546794 | 0.845594325 | -0.706599495 | -0.071428571 |
| Spermidine | -0.814385858 | 0.619047619 | 0.771140308 | 0.763762616 | 0.682646969 | -0.536110964 | 0.761904762 | -0.476190476 | 0.650612916 | 0.681930907 | -0.5 | 0.622765656 | -0.618589574 | 0.681930907 | -0.754504545 | 0.095238095 |
| Sulfathiazole | -0.407192929 | 0.476190476 | 0.349422952 | 0.327326835 | 0.646718181 | -0.824786099 | 0.714285714 | -0.333333333 | 0.466477185 | 0.354604072 | -0.642857143 | 0.538931818 | -0.866025404 | 0.354604072 | -0.131738889 | -0.452380952 |
| Swainsonine | -0.45509798 | 0.523809524 | 0.409668289 | 0.400066132 | 0.610789394 | -0.783546794 | 0.714285714 | -0.380952381 | 0.45420147 | 0.463713017 | -0.5 | 0.574860606 | -0.866025404 | 0.463713017 | -0.22754899 | -0.333333333 |
| Tartaric acid | -0.694623232 | 0.833333333 | 0.650649635 | 0.460682213 | 0.682646969 | -0.701068184 | 0.595238095 | -0.785714286 | 0.478752901 | 0.545544726 | -0.523809524 | 0.443121717 | -0.536110964 | 0.545544726 | -0.359287879 | 0.285714286 |
| Taurocholate | -0.934148484 | 0.904761905 | 0.783189376 | 0.81225548 | 0.886243434 | -0.659828879 | 0.666666667 | -0.880952381 | 0.650612916 | 0.791039852 | -0.761904762 | 0.742528282 | -0.536110964 | 0.791039852 | -0.826362121 | 0.214285714 |
| Taurolithocholic acid sulfate | -0.922172222 | 0.833333333 | 0.650649635 | 0.7516394 | 0.95810101 | -0.824786099 | 0.761904762 | -0.761904762 | 0.564682909 | 0.845594325 | -0.80952381 | 0.826362121 | -0.783546794 | 0.845594325 | -0.77845707 | -0.047619048 |
| Tenofovir | -0.754504545 | 0.928571429 | 0.771140308 | 0.800132264 | 0.814385858 | -0.659828879 | 0.619047619 | -0.904761905 | 0.785645786 | 0.791039852 | -0.571428571 | 0.658694444 | -0.618589574 | 0.791039852 | -0.658694444 | 0.047619048 |
| Thenylchlor | -0.934148484 | 0.880952381 | 0.783189376 | 0.788009048 | 0.826362121 | -0.701068184 | 0.738095238 | -0.80952381 | 0.576958624 | 0.845594325 | -0.595238095 | 0.754504545 | -0.659828879 | 0.845594325 | -0.802409595 | 0.238095238 |
| Thiouric acid | -0.898219696 | 0.761904762 | 0.626551501 | 0.691023319 | 0.934148484 | -0.824786099 | 0.785714286 | -0.666666667 | 0.540131478 | 0.763762616 | -0.833333333 | 0.77845707 | -0.783546794 | 0.763762616 | -0.718575757 | -0.071428571 |
| Trachelanthine | -0.610789394 | 0.69047619 | 0.650649635 | 0.509175077 | 0.646718181 | -0.824786099 | 0.857142857 | -0.547619048 | 0.540131478 | 0.409158544 | -0.666666667 | 0.694623232 | -0.783546794 | 0.409158544 | -0.323359091 | -0.023809524 |
| Trans_traumatic acid | -0.77845707 | 0.69047619 | 0.590404299 | 0.703146535 | 0.874267171 | -0.783546794 | 0.761904762 | -0.571428571 | 0.626061486 | 0.791039852 | -0.666666667 | 0.718575757 | -0.866025404 | 0.791039852 | -0.658694444 | -0.261904762 |
| Trans_zeatin | -0.574860606 | 0.619047619 | 0.445815491 | 0.351573268 | 0.646718181 | -0.824786099 | 0.714285714 | -0.5 | 0.331444316 | 0.463713017 | -0.571428571 | 0.574860606 | -0.783546794 | 0.463713017 | -0.251501515 | -0.071428571 |
| Trihydroxycoprostane | -0.898219696 | 0.785714286 | 0.626551501 | 0.654653671 | 0.910195959 | -0.824786099 | 0.761904762 | -0.69047619 | 0.503304332 | 0.791039852 | -0.738095238 | 0.718575757 | -0.783546794 | 0.791039852 | -0.682646969 | 0 |
| Tris(hydroxymethyl)aminomethane | -0.754504545 | 0.80952381 | 0.891630982 | 0.739516184 | 0.694623232 | -0.659828879 | 0.833333333 | -0.714285714 | 0.761094355 | 0.43643578 | -0.785714286 | 0.73055202 | -0.536110964 | 0.43643578 | -0.574860606 | 0.214285714 |
| Tyramine | -0.718575757 | 0.952380952 | 0.771140308 | 0.715269751 | 0.802409595 | -0.783546794 | 0.714285714 | -0.904761905 | 0.74881864 | 0.681930907 | -0.642857143 | 0.694623232 | -0.701068184 | 0.681930907 | -0.514979293 | 0.047619048 |
| Vitamin k1 | 0.646718181 | -0.571428571 | -0.626551501 | -0.545544726 | -0.586836868 | 0.371153744 | -0.476190476 | 0.547619048 | -0.540131478 | -0.354604072 | 0.666666667 | -0.419169192 | 0.206196525 | -0.354604072 | 0.538931818 | -0.238095238 |
| Wogonin | -0.742528282 | 0.833333333 | 0.759091241 | 0.59403759 | 0.754504545 | -0.783546794 | 0.785714286 | -0.738095238 | 0.638337201 | 0.518267489 | -0.714285714 | 0.622765656 | -0.659828879 | 0.518267489 | -0.443121717 | 0.142857143 |
| Xanthine | -0.754504545 | 0.666666667 | 0.638600568 | 0.460682213 | 0.670670707 | -0.577350269 | 0.595238095 | -0.595238095 | 0.441925755 | 0.463713017 | -0.619047619 | 0.395216666 | -0.412393049 | 0.463713017 | -0.467074242 | 0.30952381 |
| Zaleplon | -0.898219696 | 0.785714286 | 0.626551501 | 0.654653671 | 0.910195959 | -0.824786099 | 0.761904762 | -0.69047619 | 0.503304332 | 0.791039852 | -0.738095238 | 0.718575757 | -0.783546794 | 0.791039852 | -0.682646969 | 0 |
| Zanamivir | -0.73055202 | 0.833333333 | 0.734993106 | 0.848625129 | 0.826362121 | -0.783546794 | 0.80952381 | -0.738095238 | 0.785645786 | 0.791039852 | -0.642857143 | 0.874267171 | -0.866025404 | 0.791039852 | -0.682646969 | -0.214285714 |
