# Supplementary material for: To explore the mechanism of acupoint application in the treatment of primary dysmenorrhea by 16S rDNA sequencing and metabolomics
Source: Front Endocrinol (Lausanne). 2024 May 30;15:1397402. doi: 10.3389/fendo.2024.1397402 (PMC11169635; doi:10.3389/fendo.2024.1397402)
Supplement: Supplementary file 6 [file Table_6.docx]

Table S6: Screening of differential flora and differential metabolites ( Treatment VS Model )

| ID | Lachnospiraceae_NK4A136_group | Romboutsia | uncultured_proteobacterium | Parabacteroides | Anaeroplasma | Bacillus | Streptococcus | Corynebacterium_1 | Agathobacter | Others |
| --- | --- | --- | --- | --- | --- | --- | --- | --- | --- | --- |
| 11alpha_hydroxyprogesterone | 0.00385032 | 0.002008276 | 0.040372376 | 0.036552761 | 0.028004939 | 0.075689688 | 0.01017554 | 0.059897456 | 0.016540723 | 0.455644891 |
| 1,3,5(10)_estratrien_3,17.beta._diol 17_glucosiduronate | 3.31E-05 | 0.0002604 | 0.067055745 | 0.028004939 | 0.101733037 | 0.055884726 | 0.00385032 | 0.153988346 | 0.026520014 | 0.492726245 |
| 19_hydroxyandrost_4_ene_3,17_dione | 0.455644891 | 0.385120644 | 0.09250701 | 0.20703125 | 0.101733037 | 0.490015485 | 0.778885726 | 0.073305823 | 0.075689688 | 0.160025643 |
| 21_Hydroxypregnenolone | 0.085558891 | 0.046528232 | 0.170792318 | 0.20703125 | 0.036552761 | 0.136058262 | 0.070987654 | 0.018301668 | 0.000375312 | 0.778885726 |
| 2,5_furandicarboxylic acid | 0.119529806 | 0.014902668 | 0.00542395 | 0.036552761 | 0.028004939 | 0.271502387 | 0.160025643 | 0.015406042 | 0.039555628 | 0.866526271 |
| 2'_deoxyinosine | 0.101733037 | 0.036552761 | 0.327952835 | 0.232935535 | 0.119529806 | 0.083087725 | 0.020815127 | 0.053788372 | 0.022867334 | 0.570156321 |
| 2_ethyl_2_phenylmalonamide | 0.138959957 | 0.036552761 | 0.00542395 | 0.014902668 | 0.046528232 | 0.216608204 | 0.232935535 | 0.008503234 | 0.019544537 | 0.570156321 |
| 3,3'_dimethoxybenzidine | 0.232935535 | 0.160025643 | 0.025473928 | 0.182720751 | 0.070987654 | 0.777583044 | 0.492726245 | 0.088332088 | 0.039555628 | 0.182720751 |
| 3,5_dinitrosalicylate | 0.232935535 | 0.119529806 | 0.00542395 | 0.057990318 | 0.046528232 | 0.490015485 | 0.492726245 | 0.033176804 | 0.04462261 | 0.260404767 |
| 3_hydroxyphenylacetic acid | 0.232935535 | 0.101733037 | 0.011742396 | 0.101733037 | 0.014902668 | 0.452798757 | 0.351812553 | 0.015406042 | 0.030512759 | 0.419753086 |
| 4,4'_diaminodiphenylmethane | 0.101733037 | 0.119529806 | 0.275434755 | 0.455644891 | 0.046528232 | 0.332517108 | 0.138959957 | 0.073305823 | 0.000375312 | 0.385120644 |
| 4'_hydroxychalcone | 0.20703125 | 0.085558891 | 0.011742396 | 0.085558891 | 0.00385032 | 0.243269917 | 0.351812553 | 0.012816502 | 0.050063395 | 0.693238812 |
| 4_hydroxyisophthalic acid | 0.693238812 | 0.492726245 | 0.198046493 | 0.385120644 | 0.01017554 | 0.286195735 | 0.651401496 | 0.008503234 | 0.075689688 | 0.530922862 |
| 4_hydroxyquinoline | 0.020815127 | 0.028004939 | 0.170792318 | 0.182720751 | 0.036552761 | 0.075689688 | 0.046528232 | 0.105004638 | 0.019544537 | 0.651401496 |
| 4_hydroxytamoxifen | 0.01017554 | 0.01017554 | 0.025473928 | 0.01017554 | 0.085558891 | 0.075689688 | 0.070987654 | 0.096461186 | 0.039555628 | 0.260404767 |
| 4_nitrosodiphenylamine | 0.385120644 | 0.085558891 | 0.00542395 | 0.070987654 | 0.119529806 | 0.670344277 | 0.492726245 | 0.080614713 | 0.271502387 | 0.570156321 |
| 6,7_dimethylesculetin | 0.492726245 | 0.20703125 | 0.198046493 | 0.20703125 | 0.014902668 | 0.083087725 | 0.232935535 | 0.000114214 | 0.034855039 | 0.778885726 |
| 7,8_dihydroneopterin | 0.046528232 | 0.046528232 | 0.170792318 | 0.182720751 | 0.036552761 | 0.116753544 | 0.057990318 | 0.025059027 | 5.30E-07 | 0.351812553 |
| 7_hydroxy_2_acetylaminofluorene | 0.289403225 | 0.160025643 | 0.021415506 | 0.138959957 | 0.00385032 | 0.316709185 | 0.455644891 | 0.033176804 | 0.126198986 | 0.570156321 |
| 7_keto_8_aminopelargonic acid | 0.319888641 | 0.182720751 | 0.157975696 | 0.232935535 | 0.002008276 | 0.075689688 | 0.260404767 | 0.000891495 | 0.011442765 | 0.82250543 |
| (+)_abscisic acid | 0.046528232 | 0.020815127 | 0.040372376 | 0.028004939 | 0.046528232 | 0.050063395 | 0.101733037 | 0.080614713 | 0.116753544 | 0.82250543 |
| Ajmaline | 0.385120644 | 0.419753086 | 0.133974596 | 0.351812553 | 0.20703125 | 0.865730124 | 0.82250543 | 0.21339733 | 0.068692742 | 0.046528232 |
| (_)_.alpha._kainic acid | 0.182720751 | 0.101733037 | 0.040372376 | 0.057990318 | 0.028004939 | 0.136058262 | 0.289403225 | 0.008503234 | 0.013844257 | 0.385120644 |
| Anacardic acid | 0.232935535 | 0.119529806 | 0.040372376 | 0.101733037 | 0.014902668 | 0.191549313 | 0.351812553 | 0.008503234 | 0.019544537 | 0.570156321 |
| Androstan_3_ol_17_one 3_glucuronide | 0.232935535 | 0.070987654 | 0.021415506 | 0.101733037 | 0.000862968 | 0.157018422 | 0.260404767 | 0.008503234 | 0.090890436 | 0.82250543 |
| Anisomycin | 0.057990318 | 0.046528232 | 0.067055745 | 0.085558891 | 0.057990318 | 0.179628967 | 0.138959957 | 0.033176804 | 0.001110817 | 0.260404767 |
| Apiole | 0.046528232 | 0.036552761 | 0.025473928 | 0.028004939 | 0.057990318 | 0.146331533 | 0.160025643 | 0.042731523 | 0.011442765 | 0.182720751 |
| Arachidonic acid (peroxide free) | 0.289403225 | 0.119529806 | 0.083491718 | 0.20703125 | 3.31E-05 | 0.126198986 | 0.20703125 | 0.000114214 | 0.011442765 | 0.955374012 |
| Arcaine | 0.182720751 | 0.085558891 | 0.212476988 | 0.182720751 | 0.036552761 | 0.050063395 | 0.119529806 | 0.012816502 | 0.016540723 | 0.82250543 |
| Ascorbyl stearate | 0.160025643 | 0.036552761 | 0.040372376 | 0.020815127 | 0.070987654 | 0.062092692 | 0.160025643 | 0.018301668 | 0.083087725 | 0.778885726 |
| Batyl alcohol | 0.119529806 | 0.160025643 | 0.466584825 | 0.778885726 | 0.046528232 | 0.416791591 | 0.119529806 | 0.18839393 | 0.016540723 | 0.693238812 |
| .beta._muricholic acid | 0.289403225 | 0.070987654 | 0.021415506 | 0.002008276 | 0.20703125 | 0.116753544 | 0.260404767 | 0.066401565 | 0.399263874 | 0.82250543 |
| Betulin | 0.138959957 | 0.119529806 | 0.212476988 | 0.289403225 | 0.01017554 | 0.090890436 | 0.160025643 | 0.025059027 | 0.007471415 | 0.651401496 |
| Bilirubin | 0.138959957 | 0.014902668 | 0.021415506 | 0.036552761 | 0.01017554 | 0.075689688 | 0.085558891 | 0.010520131 | 0.116753544 | 0.455644891 |
| Biotin | 0.182720751 | 0.070987654 | 0.2589226 | 0.260404767 | 0.01017554 | 0.039555628 | 0.057990318 | 0.003980559 | 0.009323116 | 0.610344416 |
| Chenodeoxycholic acid | 0.020815127 | 0.00385032 | 0.011742396 | 0.006530017 | 0.085558891 | 0.116753544 | 0.046528232 | 0.133138586 | 0.191549313 | 0.73576486 |
| Cinchonine | 0.385120644 | 0.319888641 | 0.212476988 | 0.319888641 | 0.036552761 | 0.216608204 | 0.492726245 | 0.02894281 | 0.026520014 | 0.455644891 |
| Coenzyme q2 | 0.046528232 | 0.036552761 | 0.025473928 | 0.028004939 | 0.057990318 | 0.146331533 | 0.160025643 | 0.042731523 | 0.011442765 | 0.182720751 |
| Dauricine | 0.20703125 | 0.232935535 | 0.212476988 | 0.289403225 | 0.036552761 | 0.157018422 | 0.351812553 | 0.088332088 | 0.055884726 | 0.351812553 |
| Deoxypeganine | 0.036552761 | 0.002008276 | 0.040372376 | 0.020815127 | 0.057990318 | 0.068692742 | 0.014902668 | 0.018301668 | 0.022867334 | 0.866526271 |
| D_glucosaminic acid | 0.138959957 | 0.138959957 | 0.327952835 | 0.530922862 | 0.020815127 | 0.271502387 | 0.119529806 | 0.042731523 | 0.000375312 | 0.530922862 |
| DL_arginine | 0.01017554 | 0.014902668 | 0.212476988 | 0.101733037 | 0.046528232 | 0.016540723 | 0.00385032 | 0.03777021 | 0.001110817 | 0.419753086 |
| D_sorbitol | 0.351812553 | 0.160025643 | 0.083491718 | 0.182720751 | 0.0002604 | 0.107721237 | 0.289403225 | 0.000114214 | 0.019544537 | 0.910849169 |
| D_xylose | 0.20703125 | 0.036552761 | 0.083491718 | 0.070987654 | 0.01017554 | 0.030512759 | 0.070987654 | 0.001403174 | 0.062092692 | 0.419753086 |
| Erythrodiol | 0.289403225 | 0.20703125 | 0.445506322 | 0.530922862 | 0.01017554 | 0.062092692 | 0.160025643 | 0.04806854 | 0.083087725 | 0.610344416 |
| Estra_1,3,5(10),7_tetraene_3,17.beta._diol | 0.20703125 | 0.085558891 | 0.011742396 | 0.036552761 | 0.014902668 | 0.179628967 | 0.319888641 | 0.003980559 | 0.022867334 | 0.455644891 |
| Ethyl 3_indoleacetate | 0.046528232 | 0.01017554 | 0.067055745 | 0.036552761 | 0.119529806 | 0.090890436 | 0.057990318 | 0.053788372 | 0.030512759 | 0.955374012 |
| Genistein | 0.651401496 | 0.289403225 | 0.198046493 | 0.260404767 | 0.01017554 | 0.107721237 | 0.385120644 | 0.000114214 | 0.062092692 | 0.610344416 |
| Glabridin | 0.138959957 | 0.101733037 | 0.2589226 | 0.385120644 | 0.000862968 | 0.075689688 | 0.085558891 | 0.010520131 | 0.003373343 | 0.866526271 |
| Glimepiride | 0.351812553 | 0.260404767 | 0.198046493 | 0.351812553 | 0.000862968 | 0.116753544 | 0.319888641 | 0.018301668 | 0.075689688 | 0.73576486 |
| Glutaric acid | 0.866526271 | 0.351812553 | 0.083491718 | 0.182720751 | 0.046528232 | 0.348683741 | 0.693238812 | 0.002928681 | 0.179628967 | 0.570156321 |
| Huperzine b | 0.20703125 | 0.085558891 | 0.011742396 | 0.085558891 | 0.00385032 | 0.243269917 | 0.351812553 | 0.012816502 | 0.050063395 | 0.693238812 |
| Hydrocortisone | 0.119529806 | 0.046528232 | 0.00542395 | 0.028004939 | 0.028004939 | 0.257192085 | 0.260404767 | 0.012816502 | 0.013844257 | 0.351812553 |
| Hypoxanthine | 0.028004939 | 0.014902668 | 0.040372376 | 0.138959957 | 0.00385032 | 0.203876682 | 0.057990318 | 0.042731523 | 0.011442765 | 0.570156321 |
| Indole_3_pyruvic acid | 0.530922862 | 0.419753086 | 0.170792318 | 0.289403225 | 0.138959957 | 0.452798757 | 0.778885726 | 0.080614713 | 0.083087725 | 0.419753086 |
| Inosine 5'_monophosphate | 0.014902668 | 0.028004939 | 0.212476988 | 0.20703125 | 0.014902668 | 0.050063395 | 0.014902668 | 0.04806854 | 0.001110817 | 0.385120644 |
| Isopropalin | 0.101733037 | 0.070987654 | 0.212476988 | 0.319888641 | 0.006530017 | 0.126198986 | 0.070987654 | 0.010520131 | 6.55E-05 | 0.693238812 |
| Lapachol | 0.289403225 | 0.138959957 | 0.083491718 | 0.119529806 | 0.002008276 | 0.068692742 | 0.232935535 | 0.000114214 | 0.013844257 | 0.693238812 |
| L_histidinol | 0.260404767 | 0.101733037 | 0.011742396 | 0.070987654 | 0.006530017 | 0.243269917 | 0.385120644 | 0.003980559 | 0.030512759 | 0.651401496 |
| Linolenic acid | 0.419753086 | 0.232935535 | 0.445506322 | 0.455644891 | 0.020815127 | 0.075689688 | 0.160025643 | 0.002928681 | 0.011442765 | 0.693238812 |
| Lobelanidine | 0.057990318 | 0.101733037 | 0.133974596 | 0.138959957 | 0.138959957 | 0.243269917 | 0.20703125 | 0.133138586 | 0.009323116 | 0.070987654 |
| Lopinavir | 0.20703125 | 0.101733037 | 0.227437007 | 0.260404767 | 0.289403225 | 0.434639449 | 0.20703125 | 0.143354053 | 0.050063395 | 0.866526271 |
| Lsd | 0.138959957 | 0.070987654 | 0.011742396 | 0.138959957 | 0.006530017 | 0.452798757 | 0.289403225 | 0.059897456 | 0.075689688 | 0.492726245 |
| L_tryptophanamide | 0.046528232 | 0.036552761 | 0.025473928 | 0.070987654 | 0.028004939 | 0.203876682 | 0.182720751 | 0.073305823 | 0.030512759 | 0.351812553 |
| Lumichrome | 0.351812553 | 0.160025643 | 0.157975696 | 0.182720751 | 0.006530017 | 0.055884726 | 0.232935535 | 0.000268663 | 0.016540723 | 0.910849169 |
| Mefenamic acid | 0.070987654 | 0.070987654 | 0.170792318 | 0.160025643 | 0.046528232 | 0.099100808 | 0.101733037 | 0.025059027 | 0.000178157 | 0.319888641 |
| Metamitron | 0.101733037 | 0.028004939 | 0.021415506 | 0.085558891 | 0.000862968 | 0.126198986 | 0.119529806 | 0.021515531 | 0.083087725 | 0.955374012 |
| Methapyrilene | 0.070987654 | 0.028004939 | 0.067055745 | 0.119529806 | 0.046528232 | 0.229740545 | 0.101733037 | 0.025059027 | 0.001690861 | 0.693238812 |
| Metribuzin | 0.057990318 | 0.014902668 | 0.040372376 | 0.160025643 | 0.00385032 | 0.229740545 | 0.070987654 | 0.033176804 | 0.022867334 | 0.955374012 |
| Muramic acid | 0.319888641 | 0.289403225 | 0.309958736 | 0.419753086 | 0.00385032 | 0.083087725 | 0.289403225 | 0.02894281 | 0.055884726 | 0.651401496 |
| Naloxone | 0.351812553 | 0.182720751 | 0.021415506 | 0.20703125 | 0.01017554 | 0.547942604 | 0.530922862 | 0.088332088 | 0.257192085 | 0.778885726 |
| Neoabietic acid | 0.101733037 | 0.036552761 | 0.040372376 | 0.160025643 | 0.00385032 | 0.271502387 | 0.119529806 | 0.008503234 | 0.002438797 | 0.693238812 |
| Nicotinate | 0.014902668 | 0.01017554 | 0.040372376 | 0.085558891 | 0.01017554 | 0.146331533 | 0.036552761 | 0.042731523 | 0.007471415 | 0.385120644 |
| N_methyltryptamine | 0.260404767 | 0.085558891 | 0.021415506 | 0.119529806 | 0.0002604 | 0.216608204 | 0.260404767 | 0.000520392 | 0.019544537 | 0.866526271 |
| N,n_dimethylformamide | 0.101733037 | 0.028004939 | 0.021415506 | 0.085558891 | 0.000862968 | 0.126198986 | 0.119529806 | 0.021515531 | 0.083087725 | 0.955374012 |
| Nordihydroguaiaretic acid | 0.046528232 | 0.020815127 | 0.067055745 | 0.070987654 | 0.070987654 | 0.168118035 | 0.070987654 | 0.025059027 | 0.000679106 | 0.492726245 |
| Oxypurinol | 0.101733037 | 0.101733037 | 0.025473928 | 0.160025643 | 0.070987654 | 0.670344277 | 0.351812553 | 0.18839393 | 0.075689688 | 0.119529806 |
| Pentamidine | 0.119529806 | 0.046528232 | 0.00542395 | 0.028004939 | 0.028004939 | 0.257192085 | 0.260404767 | 0.012816502 | 0.013844257 | 0.351812553 |
| Pilocarpine | 0.085558891 | 0.085558891 | 0.133974596 | 0.319888641 | 0.057990318 | 0.471260529 | 0.182720751 | 0.088332088 | 0.001690861 | 0.319888641 |
| Pirimicarb | 0.036552761 | 0.028004939 | 0.040372376 | 0.014902668 | 0.057990318 | 0.039555628 | 0.101733037 | 0.059897456 | 0.055884726 | 0.351812553 |
| Plantamajoside | 0.070987654 | 0.028004939 | 0.021415506 | 0.014902668 | 0.014902668 | 0.039555628 | 0.101733037 | 0.021515531 | 0.075689688 | 0.570156321 |
| Pravastatin | 0.232935535 | 0.070987654 | 0.040372376 | 0.057990318 | 0.036552761 | 0.126198986 | 0.260404767 | 0.010520131 | 0.055884726 | 0.866526271 |
| Primaquine | 0.73576486 | 0.73576486 | 0.384743302 | 0.570156321 | 0.101733037 | 0.452798757 | 0.910849169 | 0.088332088 | 0.090890436 | 0.182720751 |
| Probucol | 0.182720751 | 0.028004939 | 0.021415506 | 0.006530017 | 0.036552761 | 0.034855039 | 0.119529806 | 0.002928681 | 0.090890436 | 0.73576486 |
| Propentofylline | 0.138959957 | 0.036552761 | 0.00542395 | 0.014902668 | 0.046528232 | 0.216608204 | 0.232935535 | 0.008503234 | 0.019544537 | 0.570156321 |
| Prosulfocarb | 0.160025643 | 0.232935535 | 0.227437007 | 0.351812553 | 0.182720751 | 0.528366471 | 0.385120644 | 0.17650983 | 0.009323116 | 0.070987654 |
| Pymetrozin | 0.01017554 | 0.00385032 | 0.025473928 | 0.085558891 | 0.020815127 | 0.216608204 | 0.046528232 | 0.096461186 | 0.030512759 | 0.693238812 |
| Pyridoxamine 5_phosphate | 0.070987654 | 0.057990318 | 0.040372376 | 0.046528232 | 0.028004939 | 0.090890436 | 0.182720751 | 0.042731523 | 0.034855039 | 0.289403225 |
| Pyrrole_2_carboxylic acid | 0.138959957 | 0.036552761 | 0.025473928 | 0.046528232 | 0.057990318 | 0.191549313 | 0.20703125 | 0.025059027 | 0.039555628 | 1 |
| Quinine | 0.20703125 | 0.085558891 | 0.011742396 | 0.036552761 | 0.014902668 | 0.179628967 | 0.319888641 | 0.003980559 | 0.022867334 | 0.455644891 |
| Sarcosine | 0.530922862 | 0.182720751 | 0.198046493 | 0.260404767 | 0.01017554 | 0.099100808 | 0.232935535 | 0.000891495 | 0.083087725 | 0.319888641 |
| Securinine | 0.260404767 | 0.160025643 | 0.157975696 | 0.160025643 | 0.006530017 | 0.04462261 | 0.20703125 | 0.000891495 | 0.007471415 | 0.610344416 |
| Sempervirine | 0.046528232 | 0.020815127 | 0.025473928 | 0.070987654 | 0.028004939 | 0.257192085 | 0.101733037 | 0.021515531 | 0.001110817 | 0.351812553 |
| Sipeimine | 0.385120644 | 0.138959957 | 0.00542395 | 0.057990318 | 0.119529806 | 0.691465352 | 0.570156321 | 0.042731523 | 0.107721237 | 0.610344416 |
| Telmisartan | 0.182720751 | 0.028004939 | 0.021415506 | 0.028004939 | 0.014902668 | 0.062092692 | 0.138959957 | 0.010520131 | 0.146331533 | 0.492726245 |
| Terbutaline | 0.138959957 | 0.138959957 | 0.327952835 | 0.530922862 | 0.020815127 | 0.271502387 | 0.119529806 | 0.042731523 | 0.000375312 | 0.530922862 |
| Tetrahydroalstonine | 0.002008276 | 0.006530017 | 0.170792318 | 0.057990318 | 0.101733037 | 0.013844257 | 0.006530017 | 0.133138586 | 0.026520014 | 0.530922862 |
| Trachelanthine | 0.351812553 | 0.160025643 | 0.021415506 | 0.20703125 | 0.000862968 | 0.434639449 | 0.455644891 | 0.015406042 | 0.083087725 | 0.778885726 |
| Trans_traumatic acid | 0.119529806 | 0.101733037 | 0.157975696 | 0.138959957 | 0.006530017 | 0.022867334 | 0.119529806 | 0.015406042 | 0.022867334 | 0.492726245 |
| Trigonelline | 0.119529806 | 0.046528232 | 0.021415506 | 0.101733037 | 0.0002604 | 0.157018422 | 0.160025643 | 0.012816502 | 0.04462261 | 0.73576486 |
| Undecanoic acid | 0.232935535 | 0.085558891 | 0.157975696 | 0.232935535 | 0.002008276 | 0.083087725 | 0.101733037 | 0.000268663 | 0.004512386 | 0.82250543 |
| Uracil | 0.260404767 | 0.160025643 | 0.157975696 | 0.260404767 | 0.000862968 | 0.075689688 | 0.232935535 | 0.005249136 | 0.022867334 | 0.866526271 |
| Uridine | 0.182720751 | 0.046528232 | 0.00542395 | 0.036552761 | 0.028004939 | 0.286195735 | 0.289403225 | 0.008503234 | 0.026520014 | 0.778885726 |
| Vincamine | 0.119529806 | 0.046528232 | 0.025473928 | 0.028004939 | 0.070987654 | 0.168118035 | 0.20703125 | 0.015406042 | 0.011442765 | 0.492726245 |
| Xanthine | 0.182720751 | 0.070987654 | 0.2589226 | 0.260404767 | 0.01017554 | 0.039555628 | 0.057990318 | 0.003980559 | 0.009323116 | 0.610344416 |
| Xanthosine | 0.101733037 | 0.020815127 | 0.011742396 | 0.101733037 | 0.002008276 | 0.257192085 | 0.138959957 | 0.021515531 | 0.050063395 | 0.910849169 |
| Zaleplon | 0.260404767 | 0.101733037 | 0.021415506 | 0.119529806 | 0.0002604 | 0.191549313 | 0.319888641 | 0.003980559 | 0.050063395 | 0.866526271 |
| Zanamivir | 0.101733037 | 0.057990318 | 0.083491718 | 0.119529806 | 0.000862968 | 0.050063395 | 0.085558891 | 0.008503234 | 0.022867334 | 0.610344416 |
